# Supplementary material for: YOD1 sustains NOD2-mediated protective signaling in colitis by stabilizing RIPK2
Source: EMBO Rep. 2024 Sep 27;25(11):4827–45. doi: 10.1038/s44319-024-00276-6 (PMC11549337; doi:10.1038/s44319-024-00276-6)
Supplement: Supplementary file 1 — Appendix [file 44319_2024_276_MOESM1_ESM.pdf]

Appendix for

YOD1 sustains NOD2-mediated protective signaling in colitis by stabilizing RIPK2

Table of contents

Appendix Figure S1 2

Appendix Figure S2 3

Appendix Figure S3 4

Appendix Figure S4 4

Appendix Figure S5 5

Appendix Figure S6 5

Appendix Figure S7 6

Appendix Figure S8 6

Appendix Figure S9 7

Appendix Figure S10 8

Appendix Figure S11 9

Appendix Figure S12 10

Appendix Figure S13 11

Appendix Figure S14 12

Appendix Figure S15 12

Appendix Figure S16 13

Appendix Table S1 14

Appendix Table S2 14

Appendix Figure S1

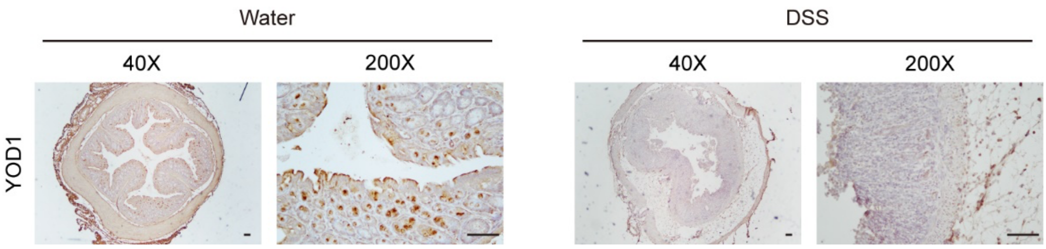

Appendix Figure S1. YOD1 is downregulated in murine colons during DSS colitis.

Representative YOD1 immunohistochemistry staining of colons from a control mouse and a DSS-treated mouse on day 8 of DSS treatment. Original magnification, ×40 and ×200. Scale bar = 100 μm.

Data information: Data are representative of three replicates.

Appendix Figure S2

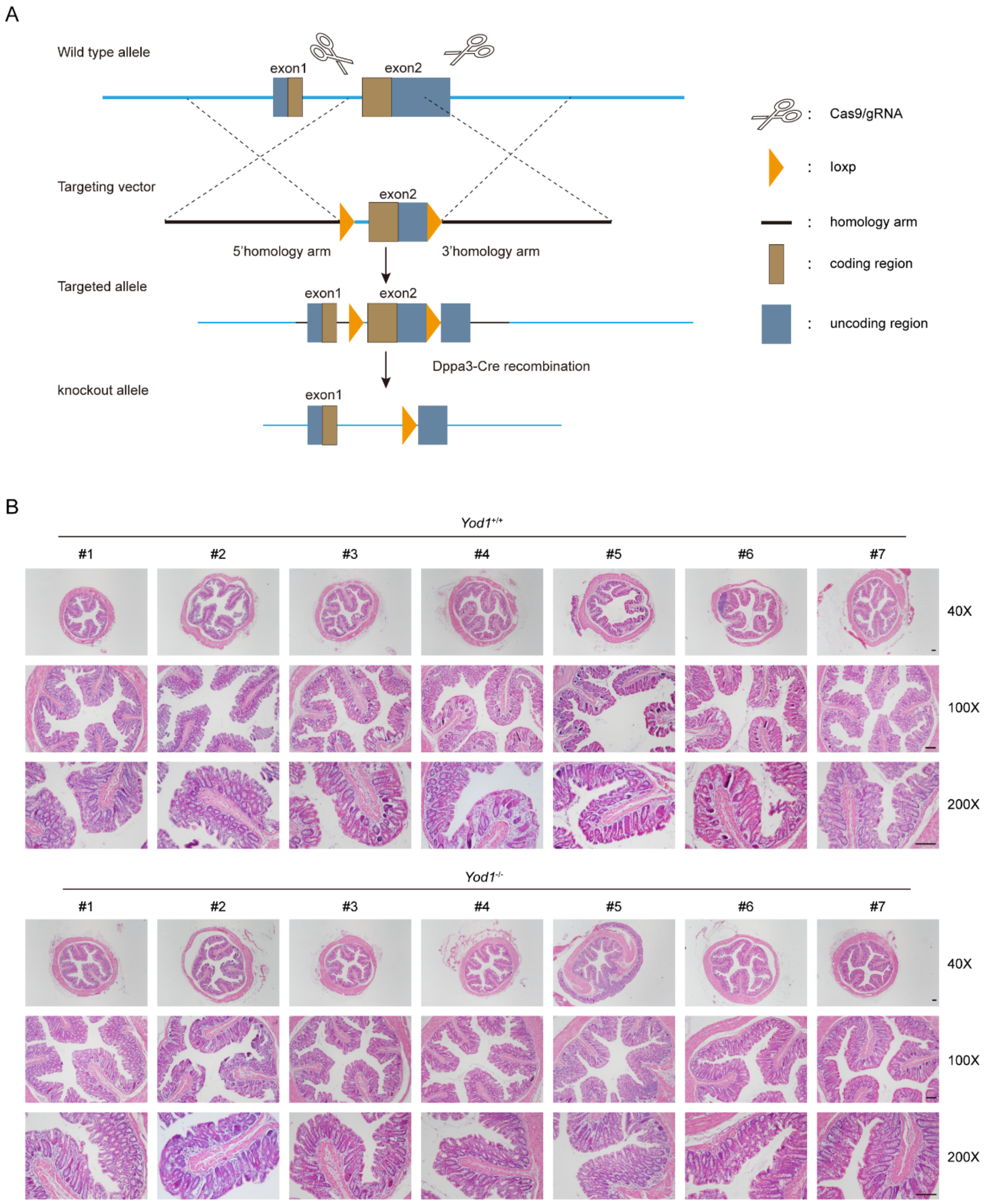

Appendix Figure S2. YOD1 ablation does not induce spontaneous colitis in mice.

(A) Schematic representation of the gene targeting strategy for the generation of *Yod1*$^{-/-}$ mice. (B) H&E staining of colons from one-year-old *Yod1*$^{+/+}$ and *Yod1*$^{-/-}$ mice. Original magnification, ×40, ×100, and ×200. Scale bar = 100 μm. The experiment in (B) was performed only once.

Appendix Figure S3

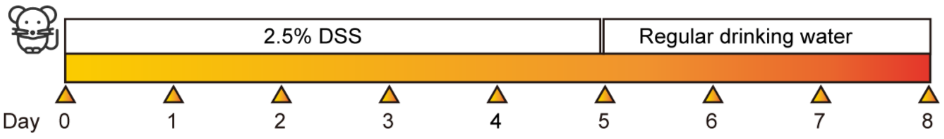

Appendix Figure S3. Experimental flowchart for the induction of DSS colitis. Mice were given drinking water containing 2.5% DSS for 5 consecutive days, followed by regular drinking water for 2-3 days. Mice were sacrificed on day 7 or 8.

Appendix Figure S4

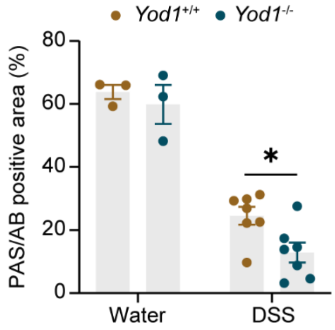

Appendix Figure S4. YOD1 ablation increased goblet cells loss during colitis. Quantification of colonic PAS/AB staining on day 8 after DSS treatment (n = 3-7, biological replicates).

Data information: Data are representative of three replicates. Data show the mean ± SEM. Statistical analyses were performed using two-way ANOVA followed by the Sidak post-test.

Appendix Figure S5

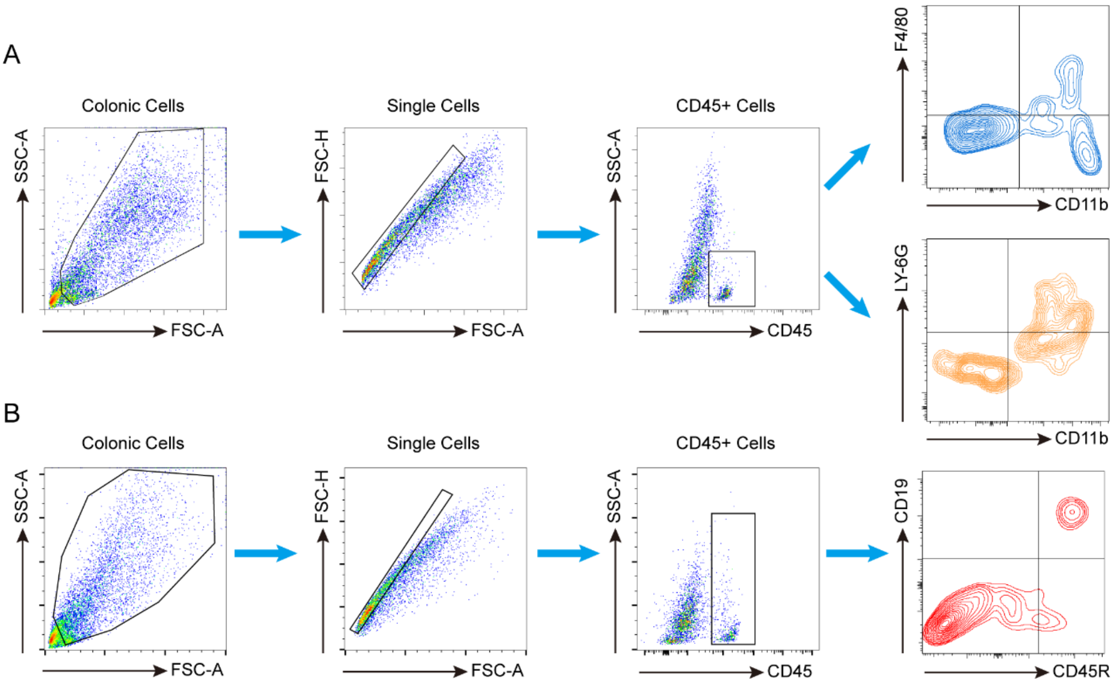

Appendix Figure S5. Gating strategy for mucosa-infiltrating leukocytes. (A) The Gating strategy for macrophages and neutrophils. (B). The Gating strategy for B cells.

Appendix Figure S6

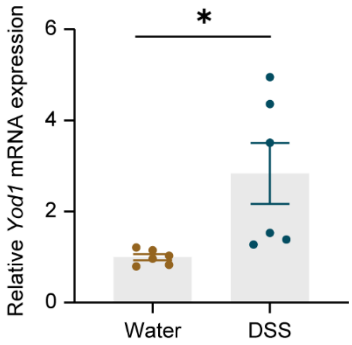

Appendix Figure S6. YOD1 expression is upregulated in colon-infiltrating macrophages during colitis. Colon-infiltrating macrophages were isolated from

control and DSS-treated mice on day 8. The mRNA levels of *Yod1* were determined by qRT-PCR (n = 6, biological replicates).

Data information: Data are representative of two replicates. Data show the mean ± SEM. Statistical analyses were performed using a two-tailed unpaired Student's *t*-test.

Appendix Figure S7

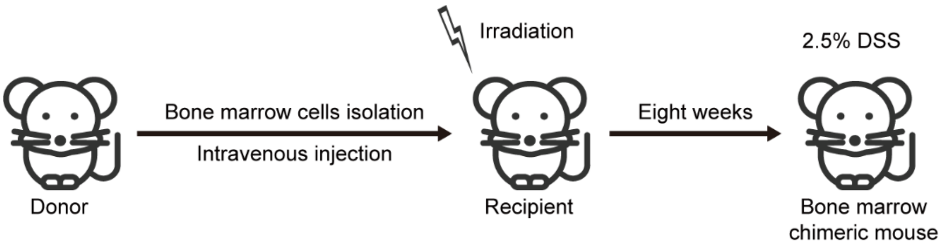

Appendix Figure S7. Experimental flowchart for the construction of bone marrow chimeric mice. The recipient mice were irradiated at a dose of 7.5 Gy. Within 6 h after irradiation, bone marrow cells from donor mice were intravenously injected into recipient mice. Eight weeks later, the recipient mice were subjected to DSS-induced colitis.

Appendix Figure S8

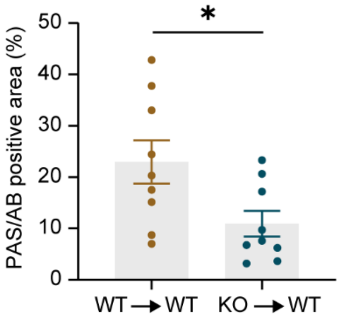

Appendix Figure S8. Pan-hematopoietic deletion of YOD1 increased goblet cells loss during colitis. Quantification of colonic PAS/AB staining on day 7 after DSS treatment (n = 9, biological replicates).

Data information: Data are representative of three replicates. Data show the mean ± SEM. Statistical analyses were performed using a two-tailed unpaired Student's *t*-test.

Appendix Figure S9

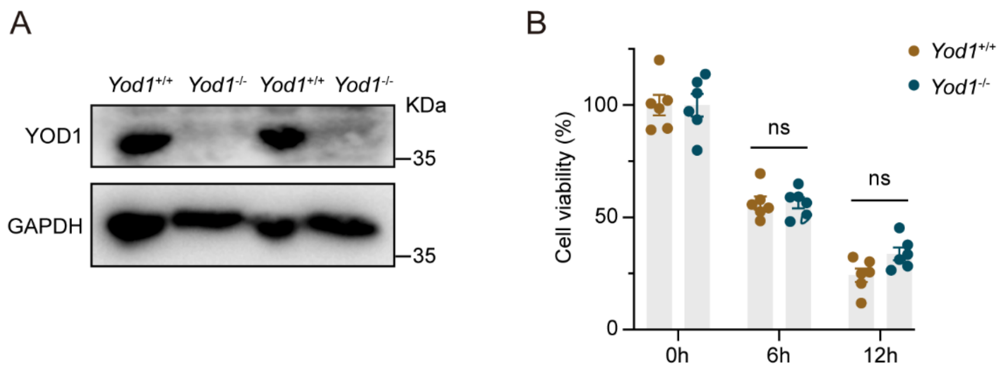

Appendix Figure S9. YOD1 deletion does not affect cell death of MODE-K cells. (A) The deletion of YOD1 in *Yod1*$^{-/-}$ MODE-K cells was confirmed by Western blot. (B) YOD1-sufficient and -deficient MODE-K cells were treated with TNF-α (40 ng/ml) and CHX (20 μg/ml) for the indicated time points. Thereafter, cell viability was determined with the CCK8 test (n = 6, technical replicates).

Data information: Data in (B) are representative of three replicates. The experiment in (A) was performed only once. Data in (B) show the mean ± SEM. Statistical analyses were performed using two-way ANOVA followed by the Sidak post-test.

Appendix Figure S10

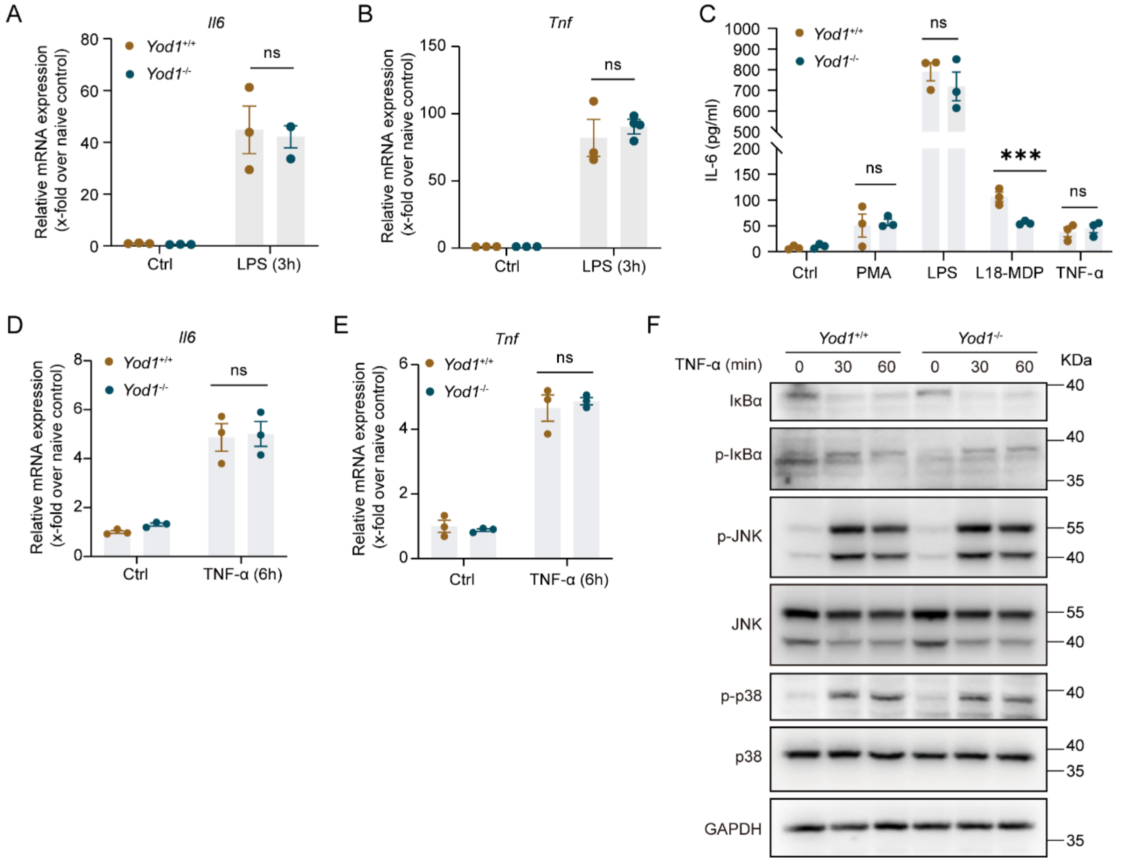

Appendix Figure S10. YOD1 deletion has no impact on cytokine production induced by LPS or TNF. (A-B) BMDMs isolated from *Yod1*$^{+/+}$ and *Yod1*$^{-/-}$ mice were stimulated with LPS (500 ng/ml) for 3 h or left untreated. The relative expression of *Il6* (A) and *Tnf* (B) mRNA was determined by qRT-PCR (n = 3, technical replicates). (C) BMDMs isolated from *Yod1*$^{+/+}$ and *Yod1*$^{-/-}$ mice were left untreated or stimulated with PMA (100 ng/ml), LPS (500 ng/ml), L18-MDP (200 ng/ml), or TNF-α (40 ng/ml) for 3 h. The concentration of IL-6 in the supernatant was determined by ELISA (n = 3, technical replicates). (D-E) BMDMs from *Yod1*$^{+/+}$ and *Yod1*$^{-/-}$ mice were stimulated with TNF-α (40 ng/ml) for 6 h or left untreated. The relative expression of *Il6* (D) and *Tnf* (E) mRNA was determined by qRT-PCR (n = 3, technical replicates). (F) BMDMs

from *Yod1*$^{+/+}$ and *Yod1*$^{-/-}$ mice were treated with TNF-α (40 ng/ml) for indicated time. Cell lysates were analyzed by Western blot with indicated antibodies.

Data information: Data are representative of three replicates. Data in (A-E) show the mean ± SEM. Statistical analyses were performed using two-way ANOVA followed by the Sidak post-test.

Appendix Figure S11

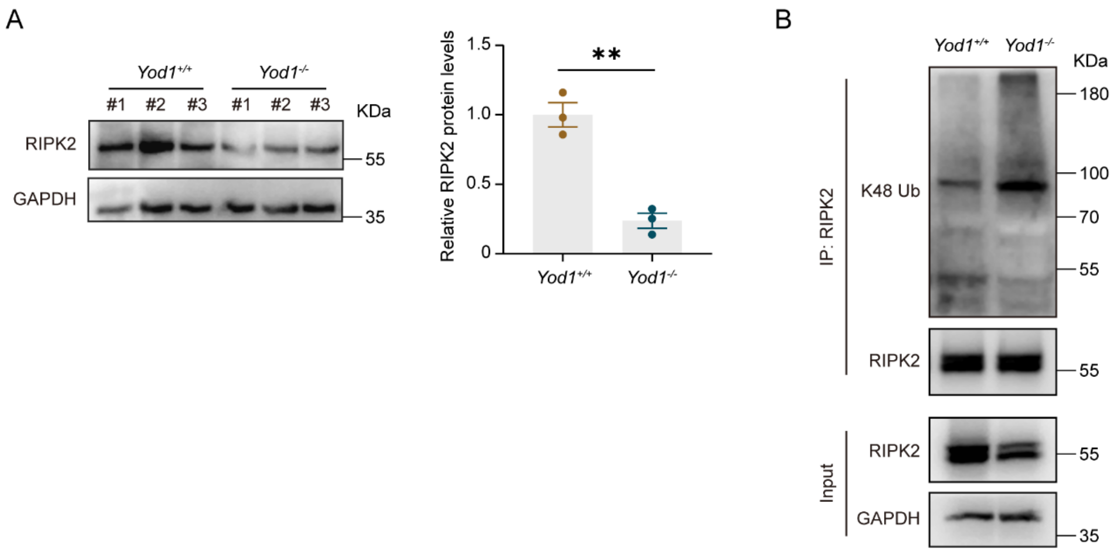

Appendix Figure S11 RIPK2 protein levels are reduced in the colon of *Yod1*$^{-/-}$ mice.

(A) RIPK2 protein abundance in the colon of *Yod1*$^{+/+}$ and *Yod1*$^{-/-}$ mice was determined by Western blot (left). The right panel shows the relative quantification (n = 3, biological replicates). (B) Proteins immunoprecipitated with anti-RIPK2 antibody from colon lysates of *Yod1*$^{+/+}$ and *Yod1*$^{-/-}$ mice were analyzed by Western blot with indicated antibodies.

Data information: Data are representative of three replicates. Data (B) show the mean ± SEM. Statistical analyses were performed using a two-tailed unpaired Student's *t*-test.

Appendix Figure S12

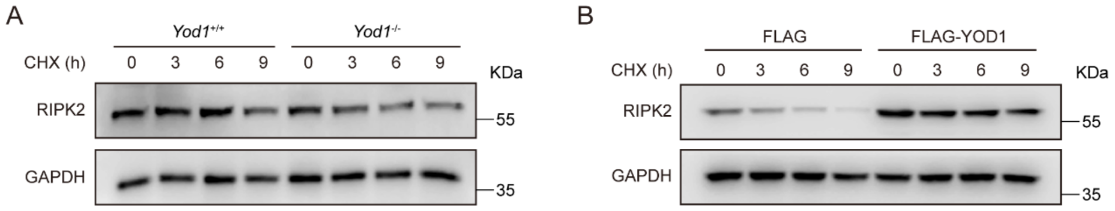

Appendix Figure S12. YOD1 inhibits the degradation of RIPK2. (A) BMDMs from *Yod1*$^{+/+}$ and *Yod1*$^{-/-}$ mice were treated with CHX (20 ng/ml) for the indicated time points. Whole-cell lysates were analyzed by Western blot with antibodies against RIPK2 and GAPDH. (B) RAW264.7 cells were transfected with FLAG or FLAG-YOD1 plasmids for 24 h, followed by treatment with CHX (20 ng/ml) for the indicated time points. Whole-cell lysates were analyzed by Western blot with antibodies against RIPK2 and GAPDH.

Data information: Data are representative of three replicates.

Appendix Figure S13

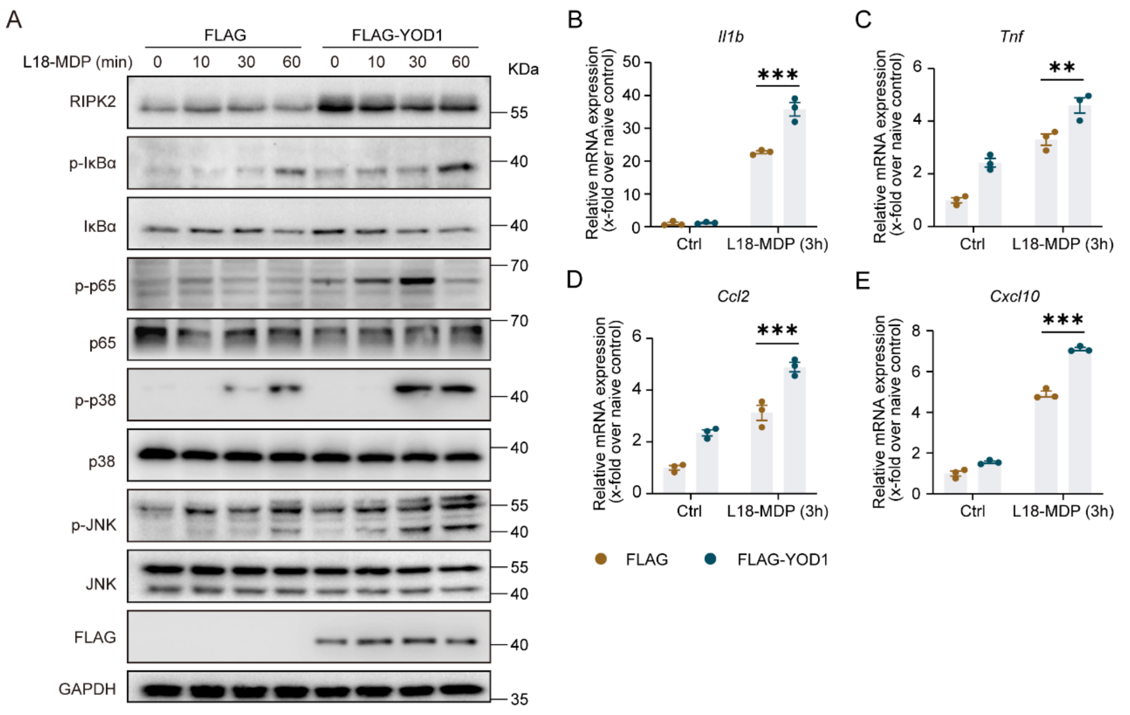

Appendix Figure S13. YOD1 overexpression enhances L18-MDP-induced signaling and gene transcription. RAW264.7 cells were transfected with FLAG or FLAG-YOD1 plasmids for 24 h. Then, cells were stimulated with L18-MDP (200 ng/ml) for the indicated time points. (A) Whole-cell lysates were analyzed by Western blot with indicated antibodies. (B-E) The relative expression of *Ilb* (B), *Tnf* (C), *Ccl2* (D), and *Cxcl10* (E) mRNA was detected by qRT-PCR (n = 3, technical replicates).

Data information: Data are representative of three replicates. Data in (B-E) show the mean ± SEM. Statistical analyses were performed using two-way ANOVA followed by the Sidak post-test.

Appendix Figure S14

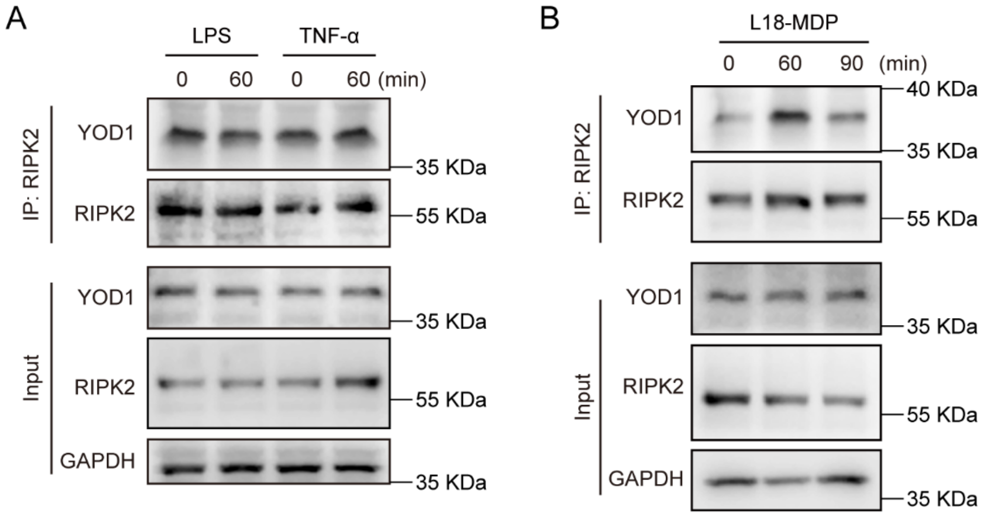

Appendix Figure S14. L18-MDP induces the interaction between YOD1 and RIPK2. BMDMs were treated with LPS, TNF-α (A), or L18-MDP (B) for indicated time before lysis. Proteins immunoprecipitated with anti-RIPK2 antibody were analyzed by Western blot with indicated antibodies. Data are representative of two replicates.

Appendix Figure S15

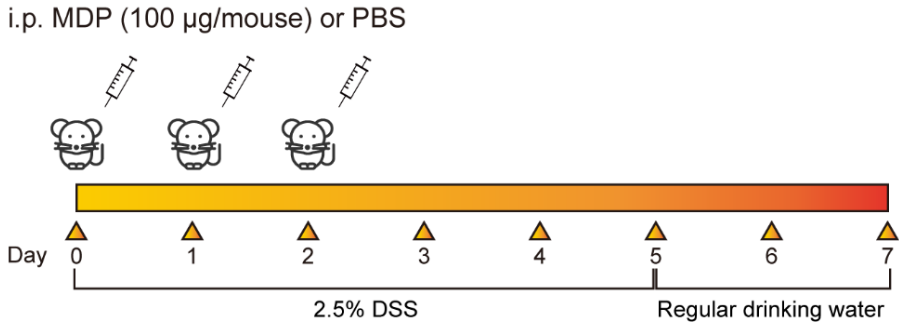

Appendix Figure S15. Experimental flowchart for the MDP treatment. Mice were intraperitoneally administered with MDP (100 μg/mouse) or volume-matched PBS for 3 days starting on day 0.

Appendix Figure S16

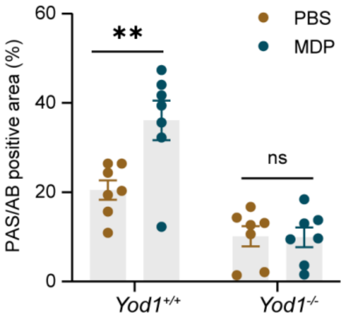

Appendix Figure S16. MDP treatment reduced goblet cell loss in *Yod1*$^{+/+}$ mice during colitis, but not in *Yod1*$^{-/-}$ mice. MDP treatment increased goblet cells loss during colitis. Quantification of colonic PAS/AB staining on day 7 after DSS treatment (n = 7, biological replicates).

Data information: Data are representative of three replicates. Data show the mean ± SEM. Statistical analyses were performed using two-way ANOVA followed by the Sidak post-test.

Appendix Table S1. Primers for qRT-PCR.

**Gene**	**Species**	**Forward primer**	**Reverse primer**
Yod1	Mouse	ATGTAAACCGCTTCACCCTGAGATG	ATGGTCCCTTGCTTCTGCTTGTC
Il1b	Mouse	TCGCAGCAGCACATCAACAAGAG	AGGTCCACGGGAAAGACACAGG
Il6	Mouse	ACAACCACGGCCTTCCCTAC	TCCACGATTTCCCAGAGAACA
Il12	Mouse	TGGTTTGCCATCGTTTTGC	GGGAGTCCAGTCCACCTCTACA
Tnf	Mouse	ATGTCTCAGCCTCTTCTCATTC	GCTTGTCACTCGAATTTTGAGA
Ccl2	Mouse	GAGCAGTGTGGAGTTCGAGG	CCGGATCTAGGCAGGTTTGA
Cxcl10	Mouse	CTCATCCTGCTGGGTCTGAG	CCTATGGCCCTCATTCTCAC
Ripk2	Mouse	TCGTGTGGATCCTCTCTGCTCT	TTCCAGGACAGTGGTGTGCCTT
Actin	Mouse	CTACCTCATGAAGATCCTGACC	CACAGCTTCTCTTTGATGTCAC

Appendix Table S2. Patient information.

Nr.	Sex	Age	Pathological diagnosis	Group
E22-05372	Male	46	ulcerative colitis	UC
E22-06707	Female	30	ulcerative colitis	UC
E22-08720	Female	44	ulcerative colitis	UC
E22-10158	Male	19	ulcerative colitis	UC
E22-13123	Male	58	ulcerative colitis	UC
22-79744	Male	29	ulcerative colitis	UC
22-85879	Male	69	ulcerative colitis	UC
22-86624	Male	36	ulcerative colitis	UC
23-05188	Male	28	ulcerative colitis	UC
D23-03119	Male	69	Sigmoid colon cancer	Control
D23-03120	Male	85	Rectal cancer	Control
D23-03237	Male	75	Rectal cancer	Control
D23-05105	Male	74	Rectal cancer	Control
D23-05106	Female	50	Sigmoid colon cancer	Control
D23-05107	Male	70	Rectal cancer	Control
D23-05234	Male	56	Ascending colon cancer	Control
