# Supplementary figures and images for: YOD1 sustains NOD2-mediated protective signaling in colitis by stabilizing RIPK2 (part 1 of 3)
Source: EMBO Rep. 2024 Sep 27;25(11):4827–45. doi: 10.1038/s44319-024-00276-6 (PMC11549337; doi:10.1038/s44319-024-00276-6)

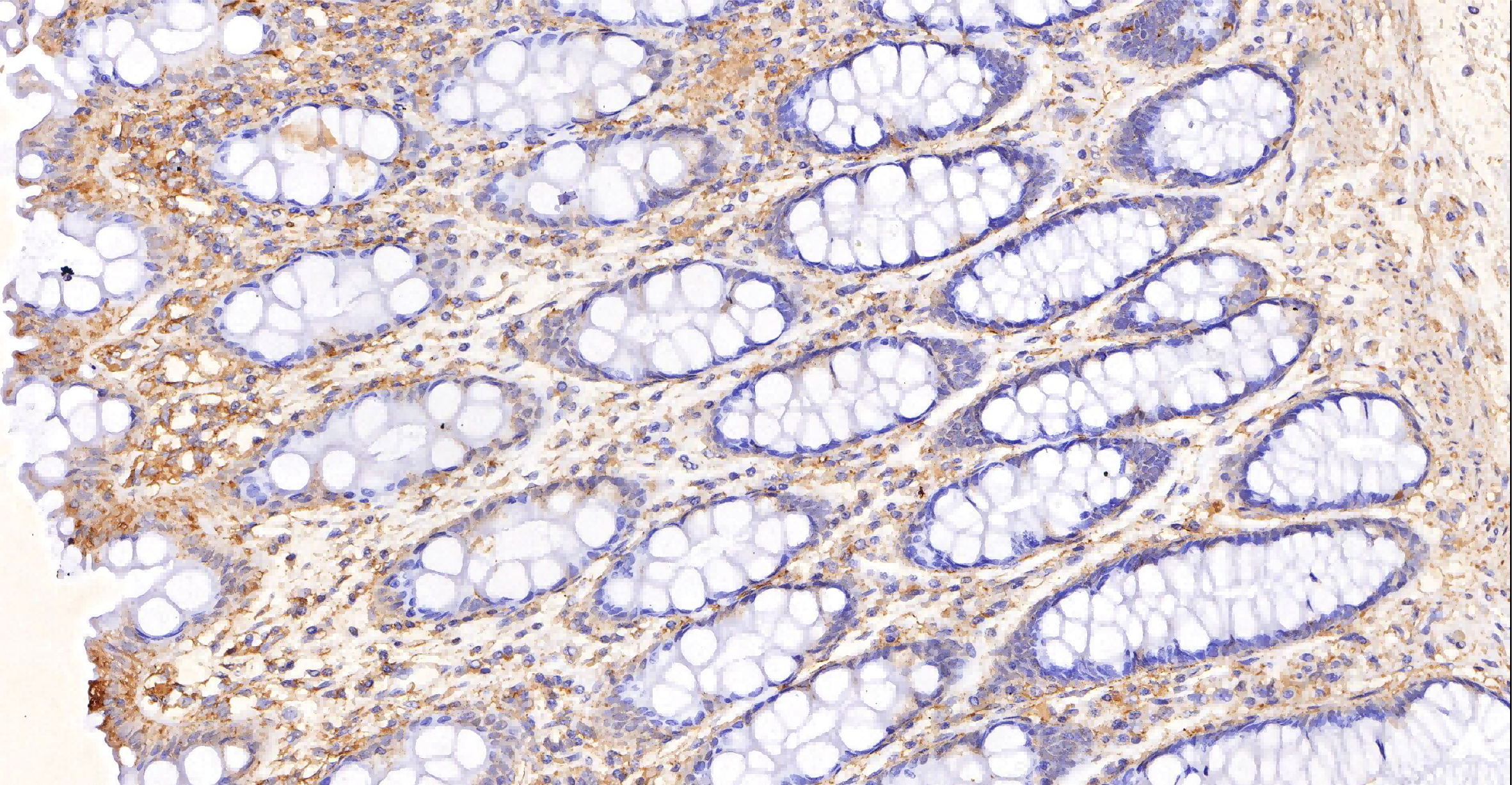

Supplement: Supplementary file 6 — Source data Fig. 1 [file 44319_2024_276_MOESM6_ESM.zip › Fig 1/1B/Control.png]

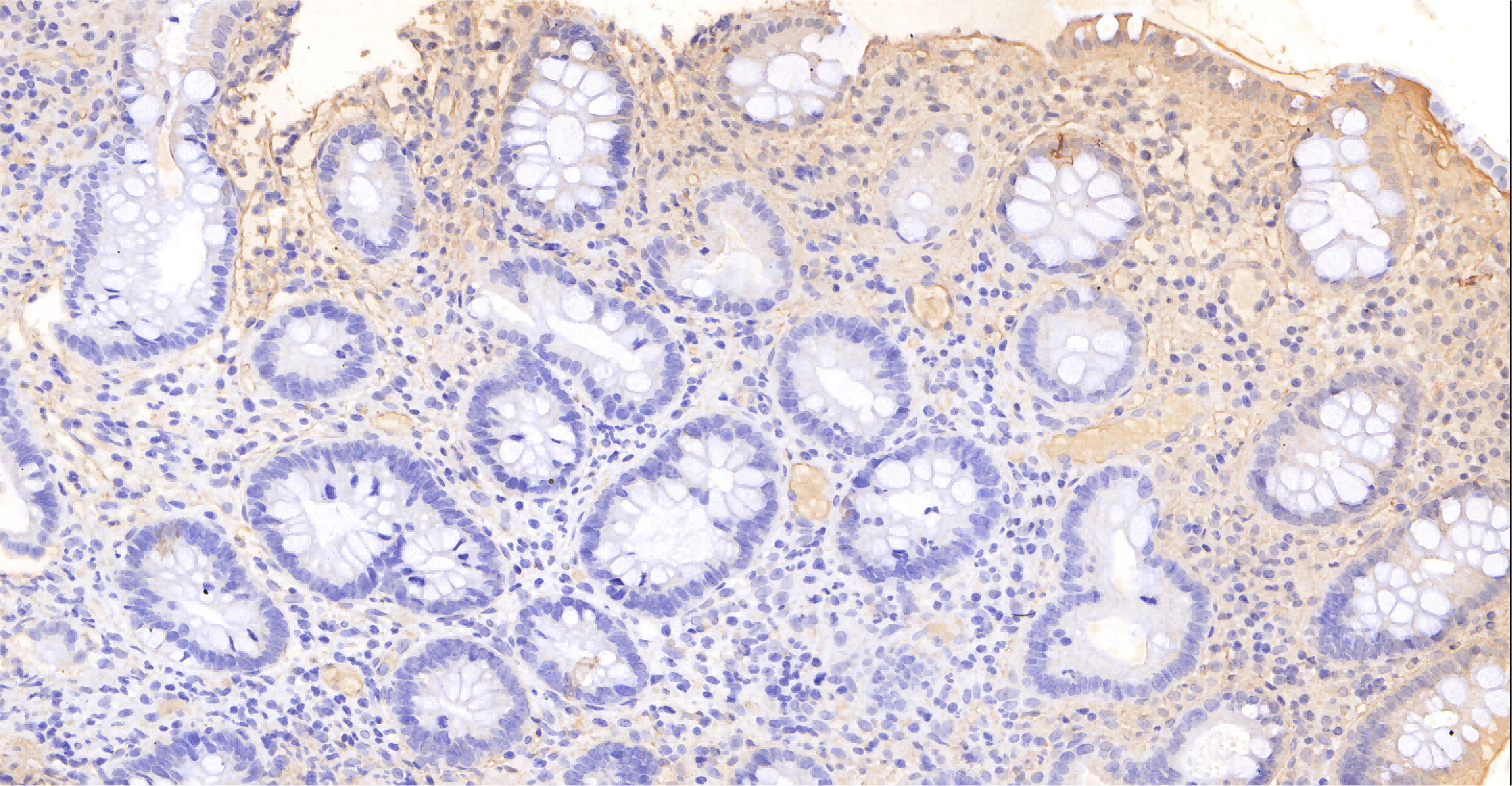

Supplement: Supplementary file 6 — Source data Fig. 1 [file 44319_2024_276_MOESM6_ESM.zip › Fig 1/1B/UC.png]

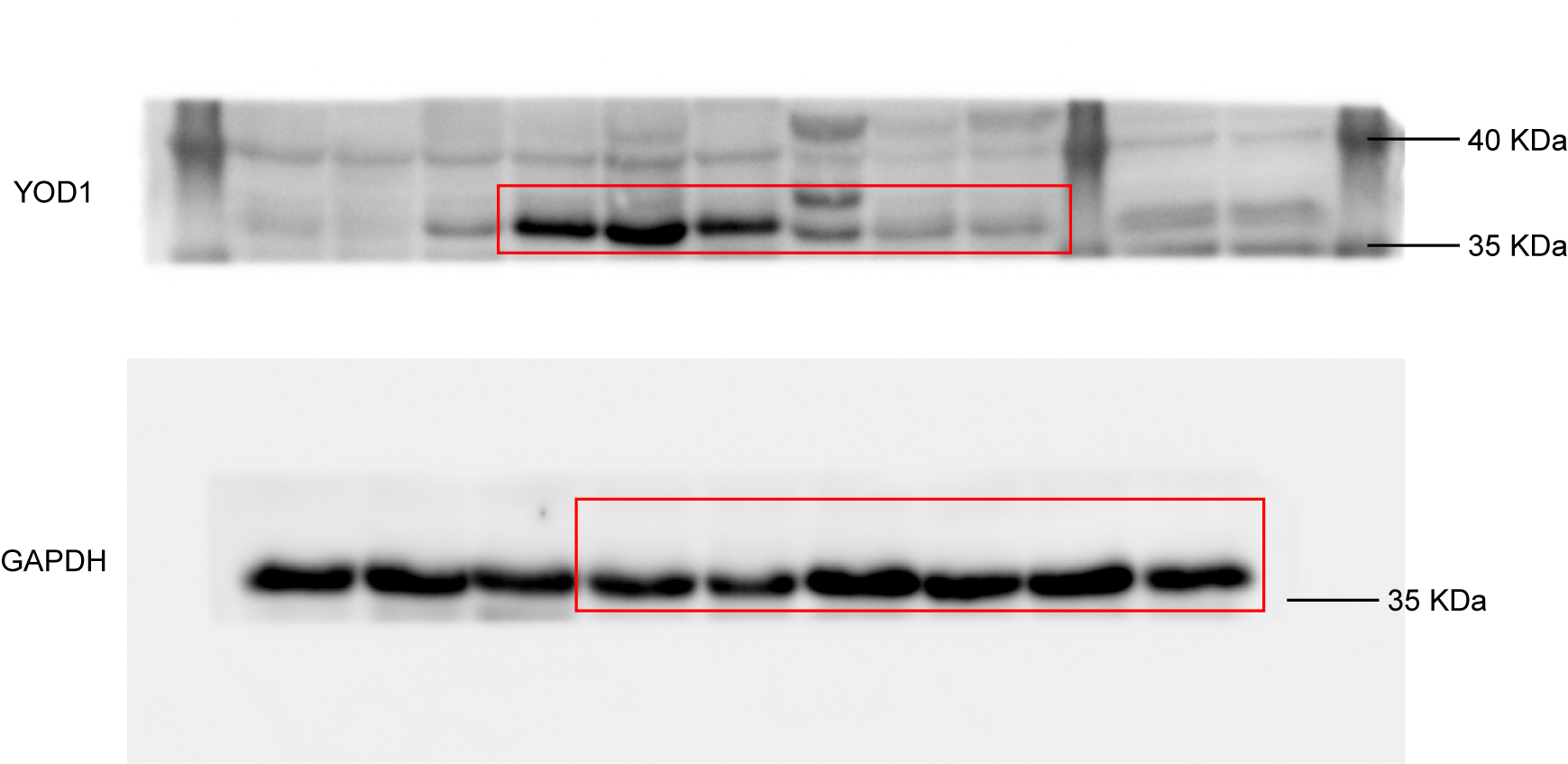

Supplement: Supplementary file 6 — Source data Fig. 1 [file 44319_2024_276_MOESM6_ESM.zip › Fig 1/1D/1D.tif]

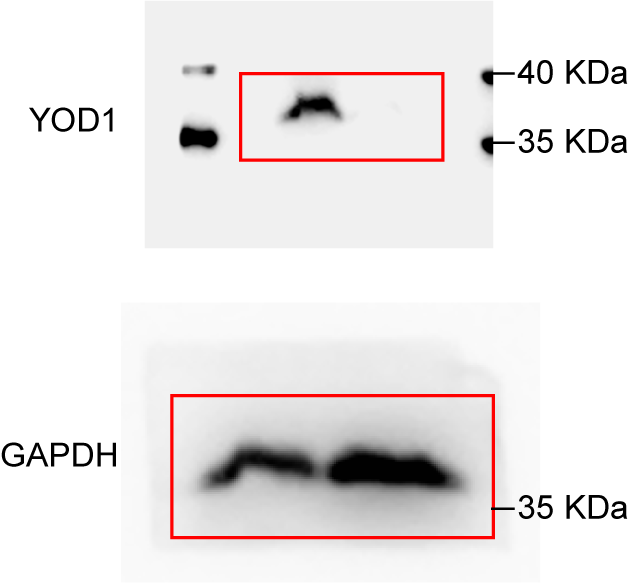

Supplement: Supplementary file 6 — Source data Fig. 1 [file 44319_2024_276_MOESM6_ESM.zip › Fig 1/1E/1E.tif]

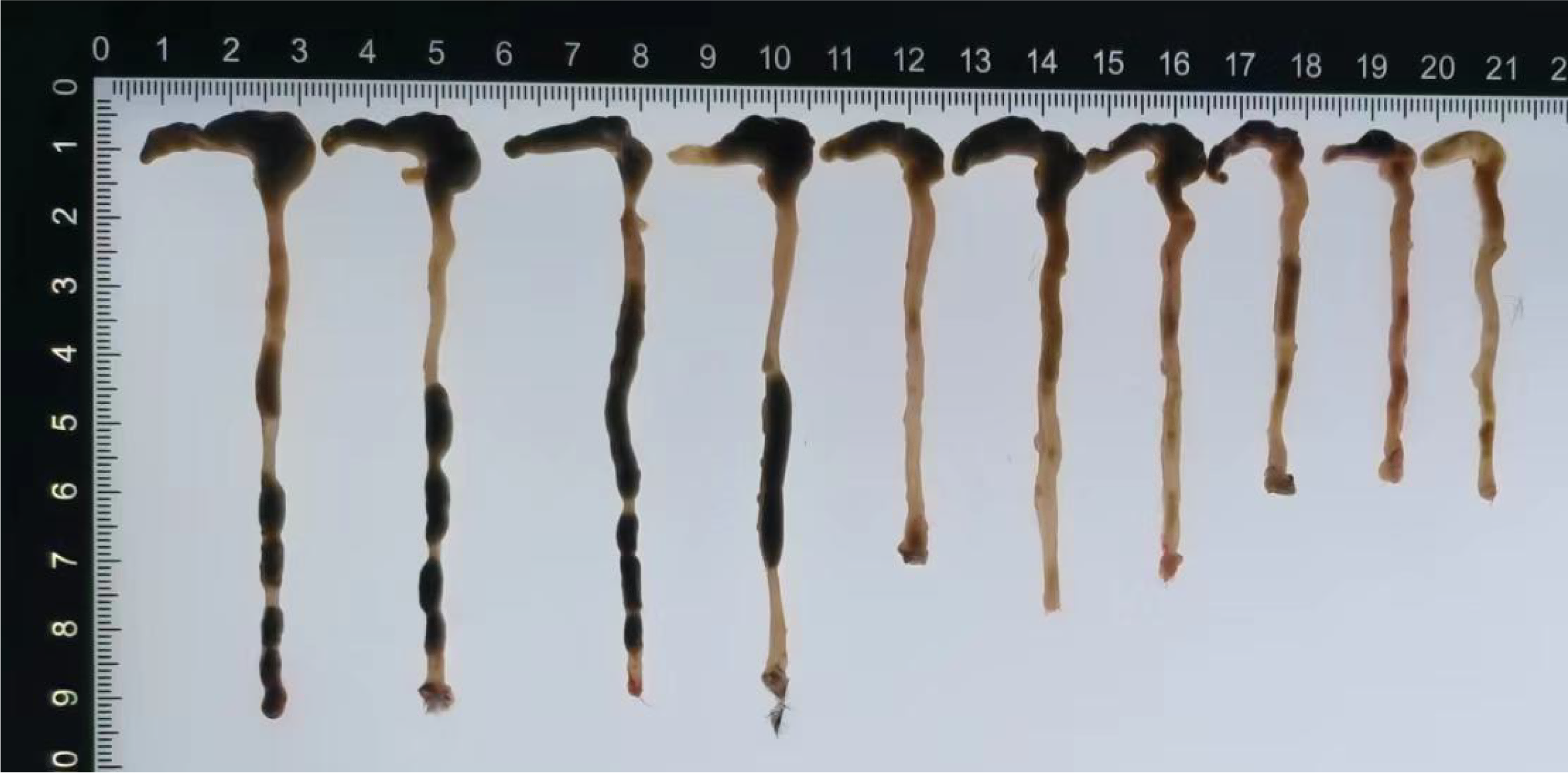

Supplement: Supplementary file 6 — Source data Fig. 1 [file 44319_2024_276_MOESM6_ESM.zip › Fig 1/1G/1G.png]

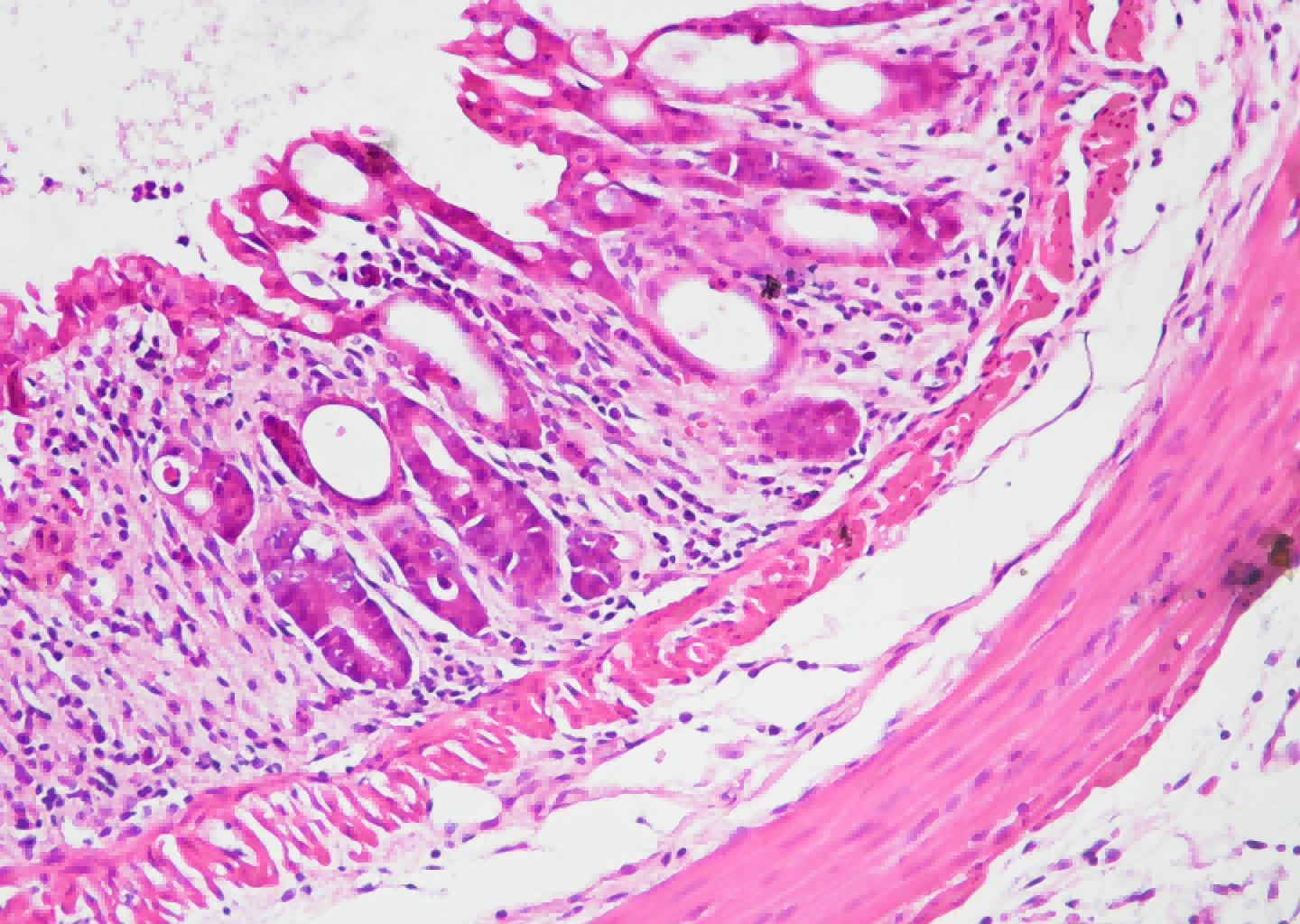

Supplement: Supplementary file 6 — Source data Fig. 1 [file 44319_2024_276_MOESM6_ESM.zip › Fig 1/1M/HE staining/Yod1++_DSS_200×.png]

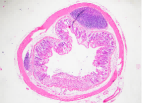

Supplement: Supplementary file 6 — Source data Fig. 1 [file 44319_2024_276_MOESM6_ESM.zip › Fig 1/1M/HE staining/Yod1++_DSS_40×.png]

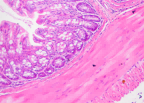

Supplement: Supplementary file 6 — Source data Fig. 1 [file 44319_2024_276_MOESM6_ESM.zip › Fig 1/1M/HE staining/Yod1++_water_200×.png]

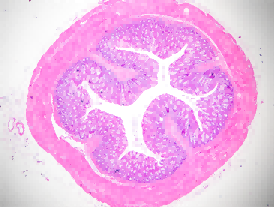

Supplement: Supplementary file 6 — Source data Fig. 1 [file 44319_2024_276_MOESM6_ESM.zip › Fig 1/1M/HE staining/Yod1++_water_40×.png]

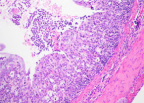

Supplement: Supplementary file 6 — Source data Fig. 1 [file 44319_2024_276_MOESM6_ESM.zip › Fig 1/1M/HE staining/Yod1--_DSS_200×.png]

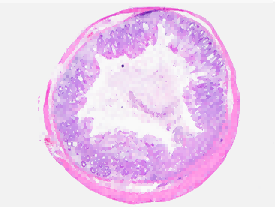

Supplement: Supplementary file 6 — Source data Fig. 1 [file 44319_2024_276_MOESM6_ESM.zip › Fig 1/1M/HE staining/Yod1--_DSS_40×.png]

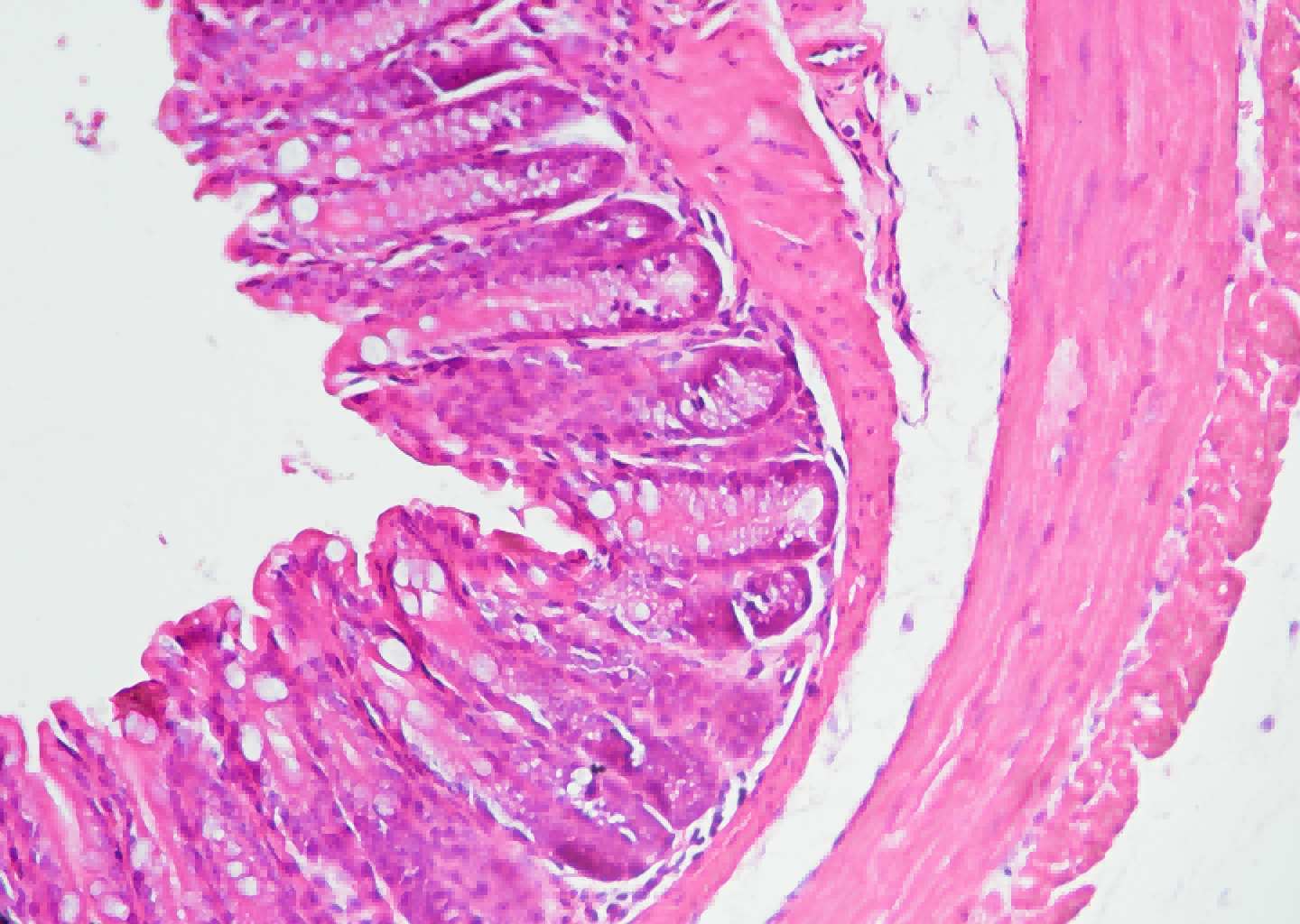

Supplement: Supplementary file 6 — Source data Fig. 1 [file 44319_2024_276_MOESM6_ESM.zip › Fig 1/1M/HE staining/Yod1--_water_200×.png]

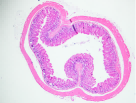

Supplement: Supplementary file 6 — Source data Fig. 1 [file 44319_2024_276_MOESM6_ESM.zip › Fig 1/1M/HE staining/Yod1--_water_40×.png]

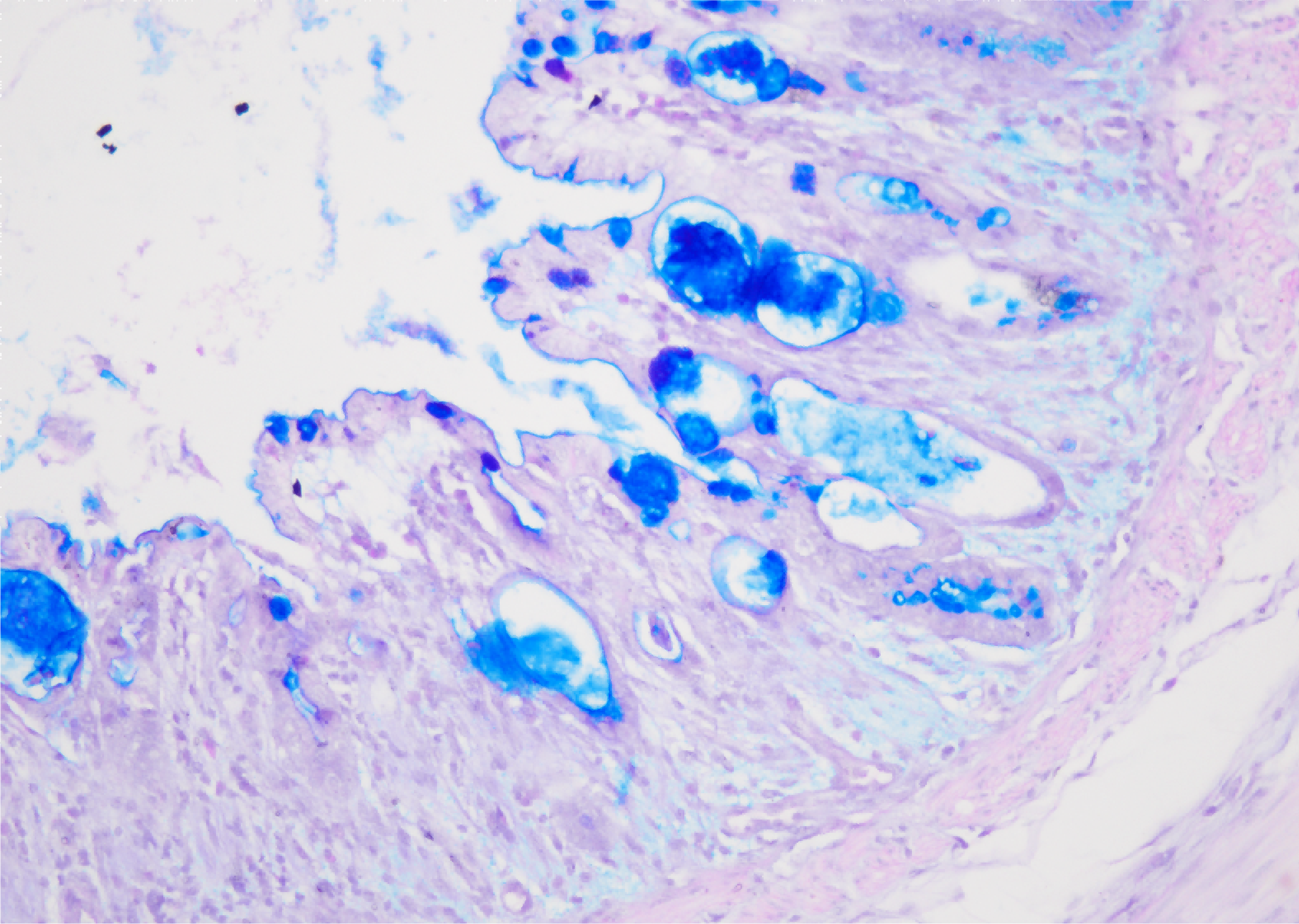

Supplement: Supplementary file 6 — Source data Fig. 1 [file 44319_2024_276_MOESM6_ESM.zip › Fig 1/1M/PAS_AB staining/Yod1++_DSS-200×.png]

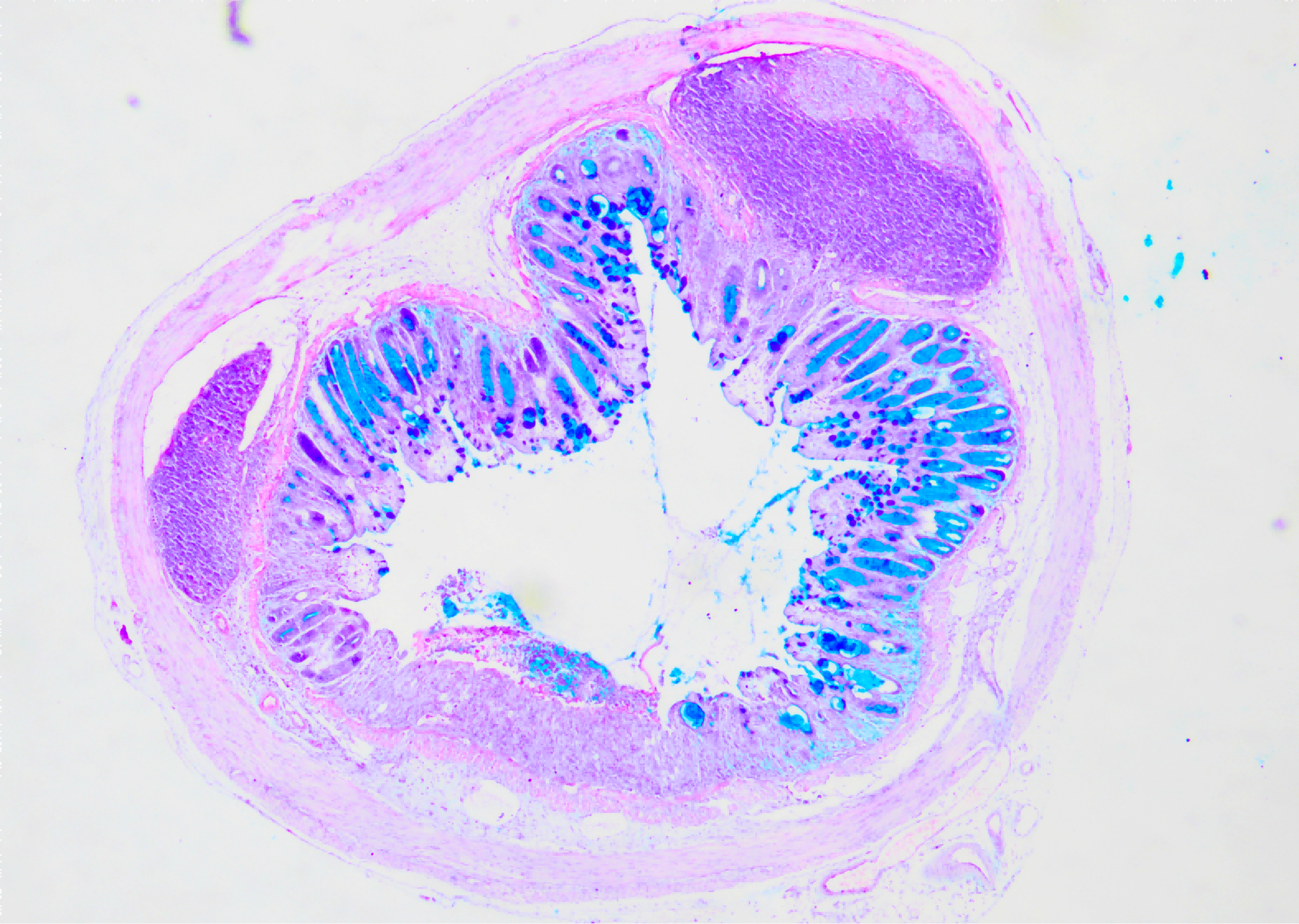

Supplement: Supplementary file 6 — Source data Fig. 1 [file 44319_2024_276_MOESM6_ESM.zip › Fig 1/1M/PAS_AB staining/Yod1++_DSS-40×.png]

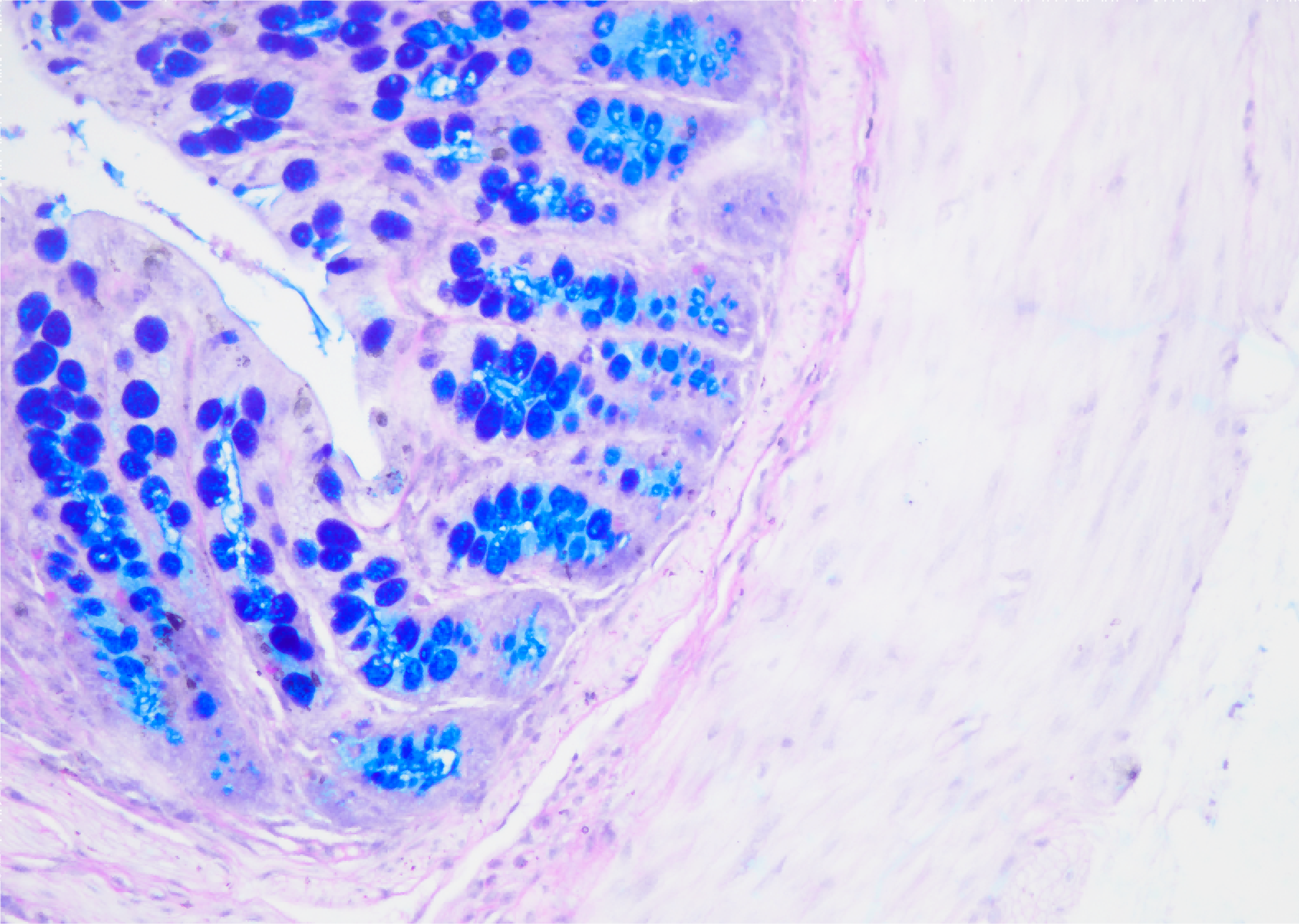

Supplement: Supplementary file 6 — Source data Fig. 1 [file 44319_2024_276_MOESM6_ESM.zip › Fig 1/1M/PAS_AB staining/Yod1++_water-200×.png]

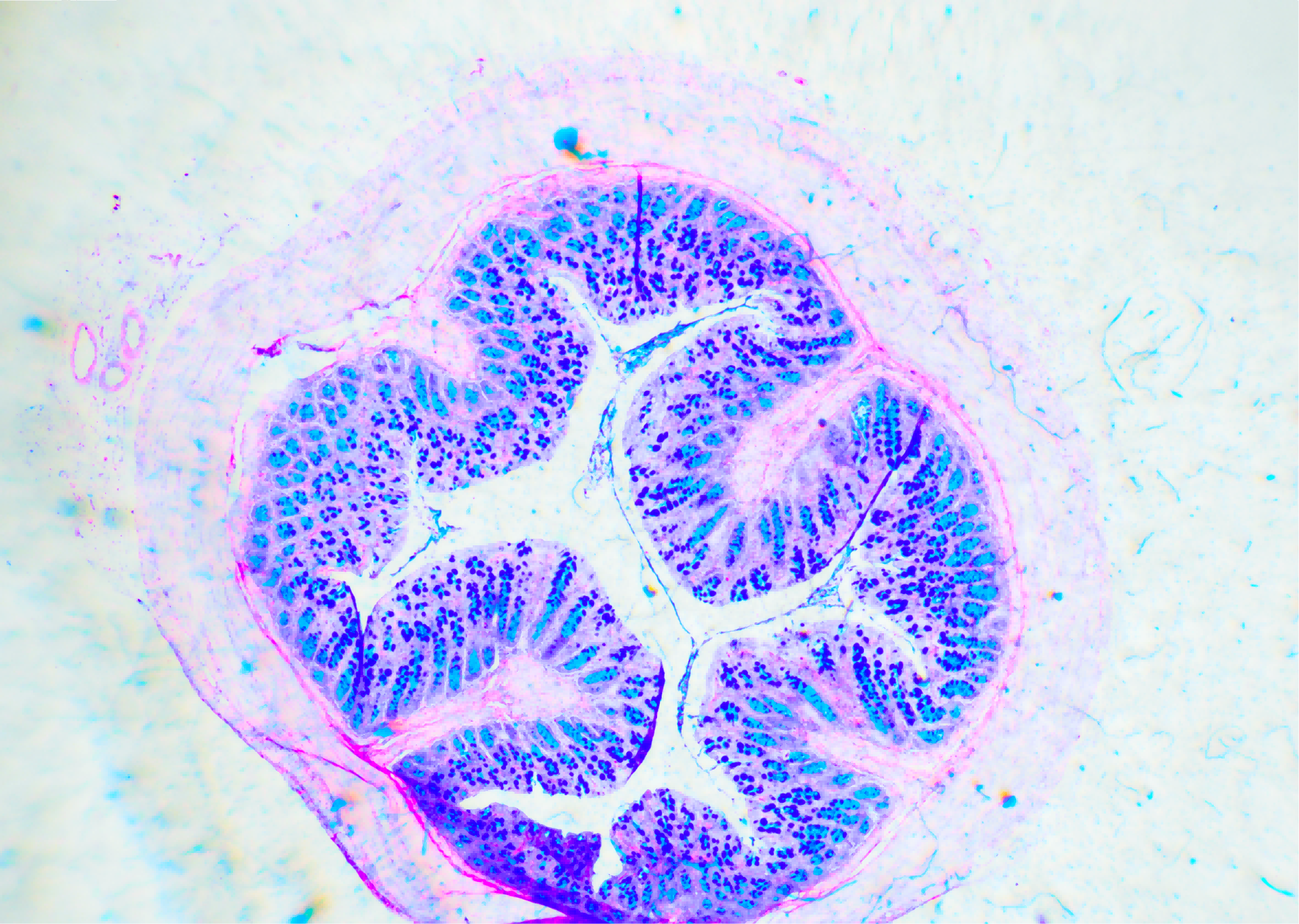

Supplement: Supplementary file 6 — Source data Fig. 1 [file 44319_2024_276_MOESM6_ESM.zip › Fig 1/1M/PAS_AB staining/Yod1++_water-40×.png]

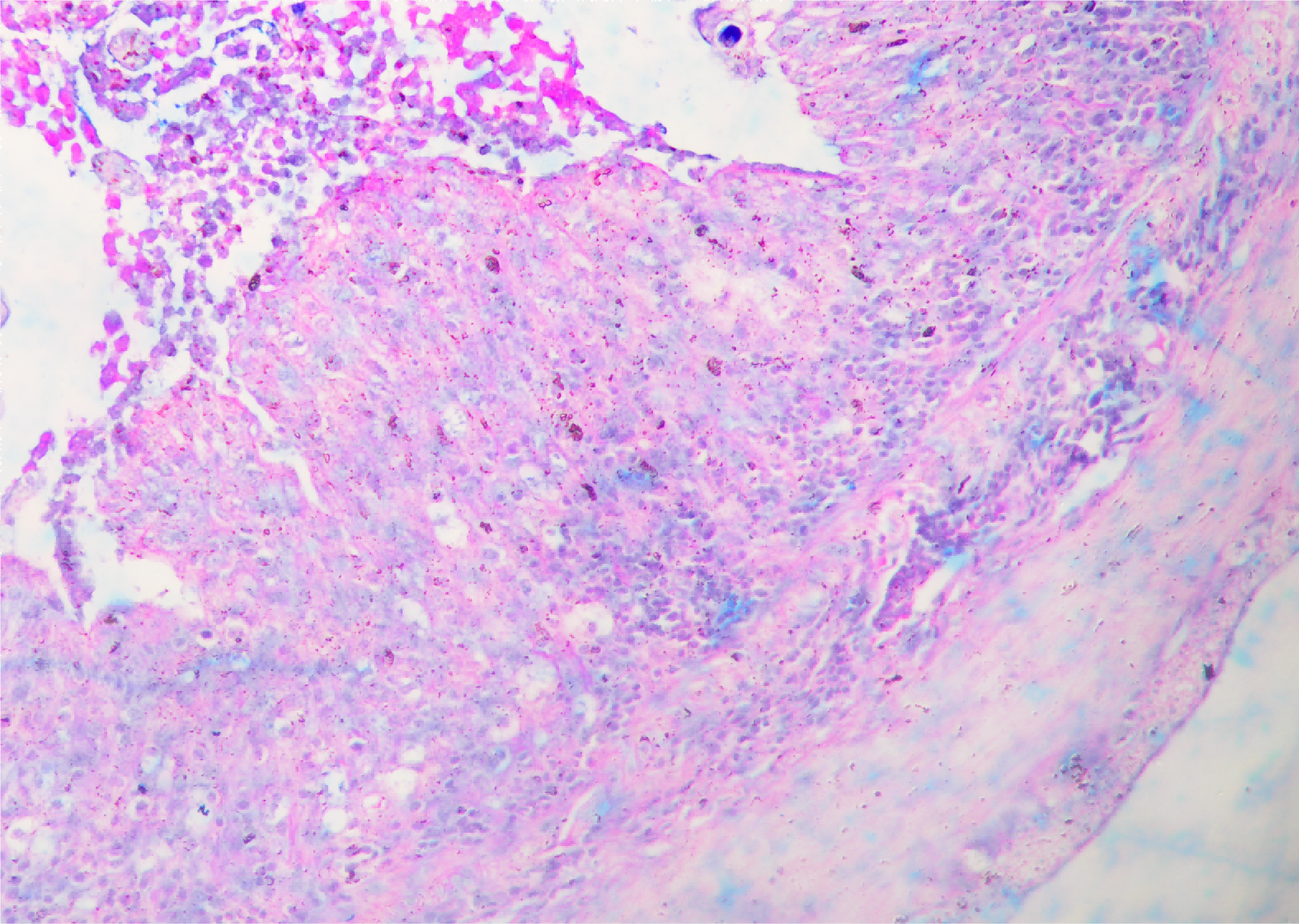

Supplement: Supplementary file 6 — Source data Fig. 1 [file 44319_2024_276_MOESM6_ESM.zip › Fig 1/1M/PAS_AB staining/Yod1--_DSS-200×.png]

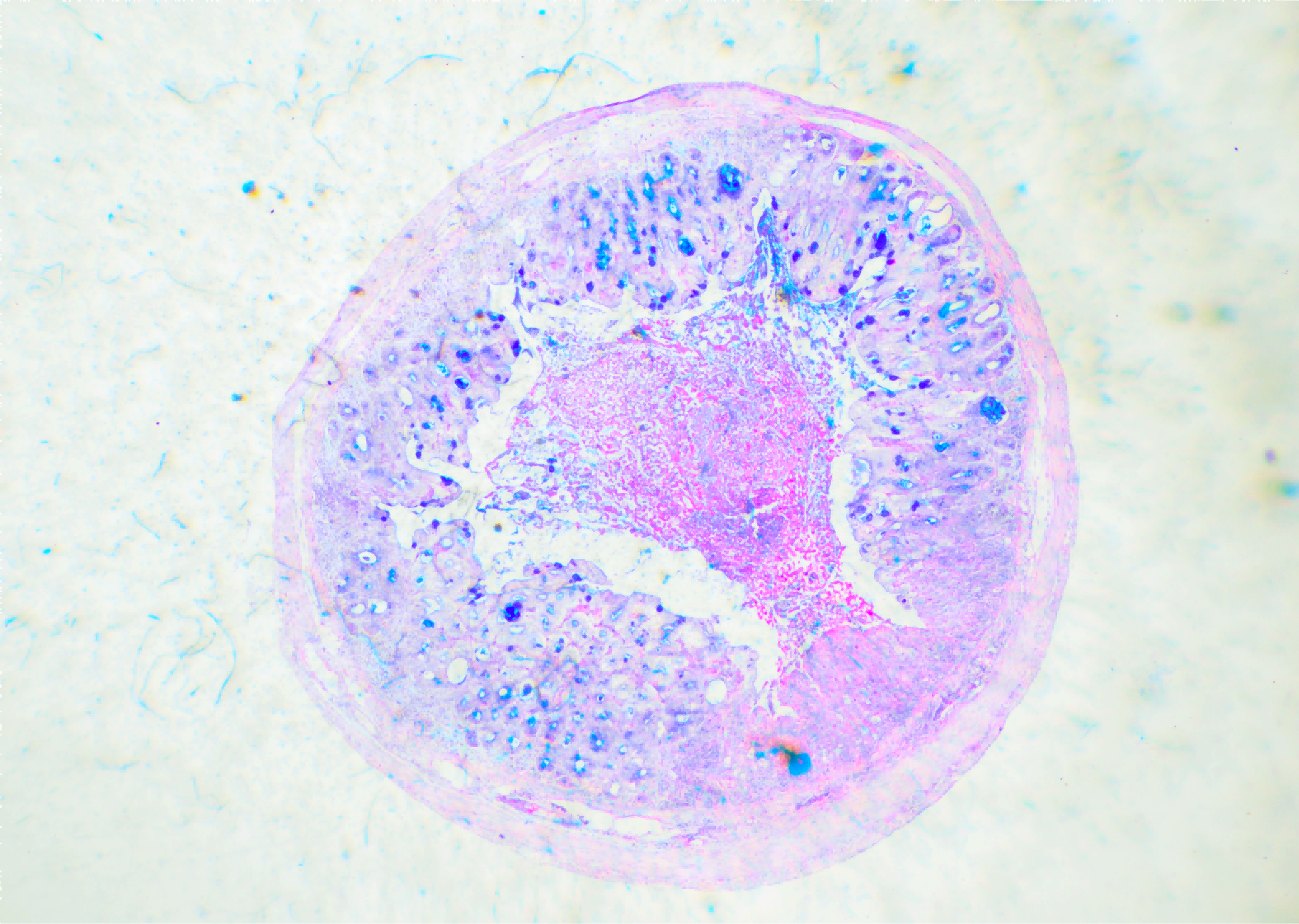

Supplement: Supplementary file 6 — Source data Fig. 1 [file 44319_2024_276_MOESM6_ESM.zip › Fig 1/1M/PAS_AB staining/Yod1--_DSS-40×.png]

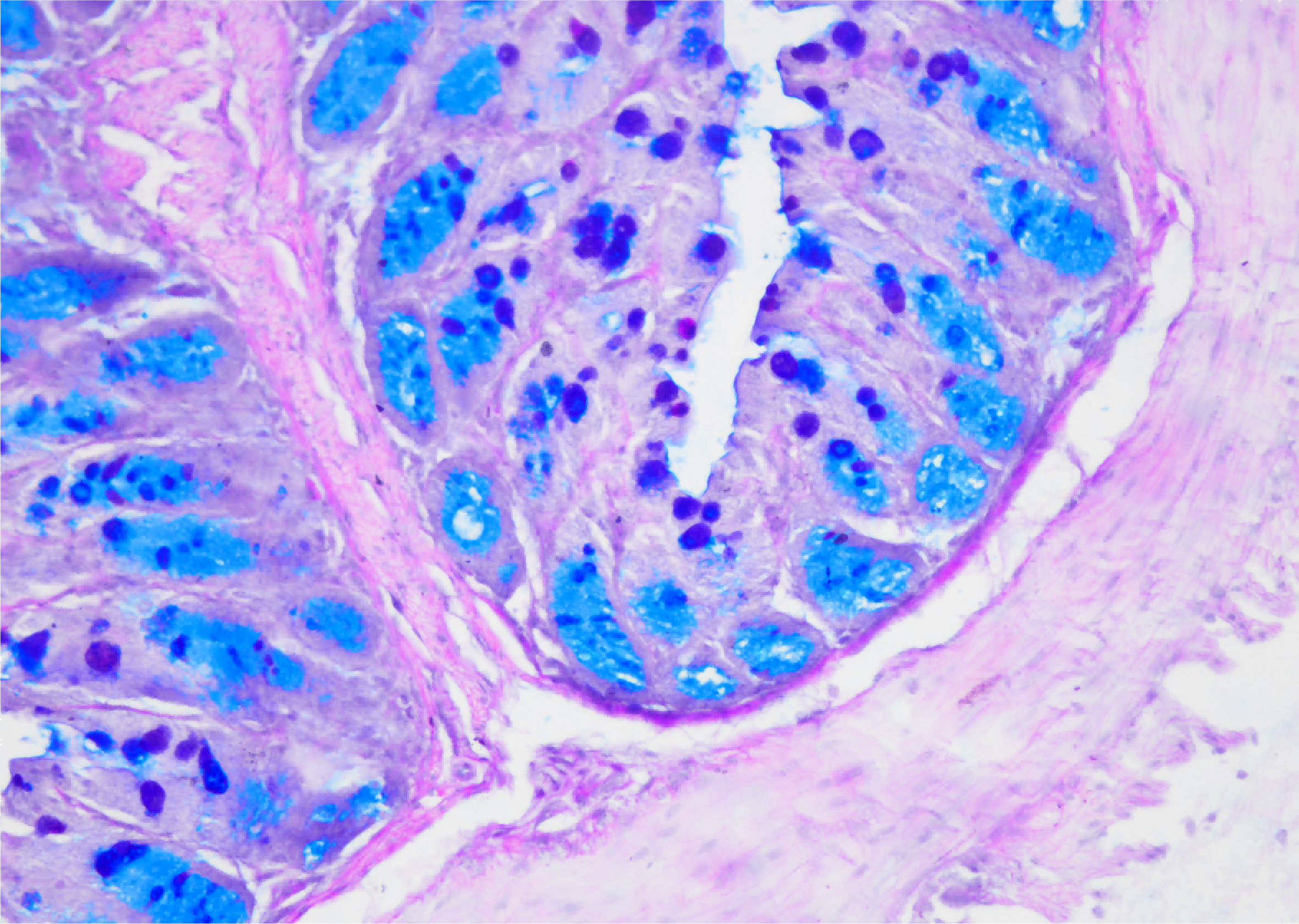

Supplement: Supplementary file 6 — Source data Fig. 1 [file 44319_2024_276_MOESM6_ESM.zip › Fig 1/1M/PAS_AB staining/Yod1--_water-200×.png]

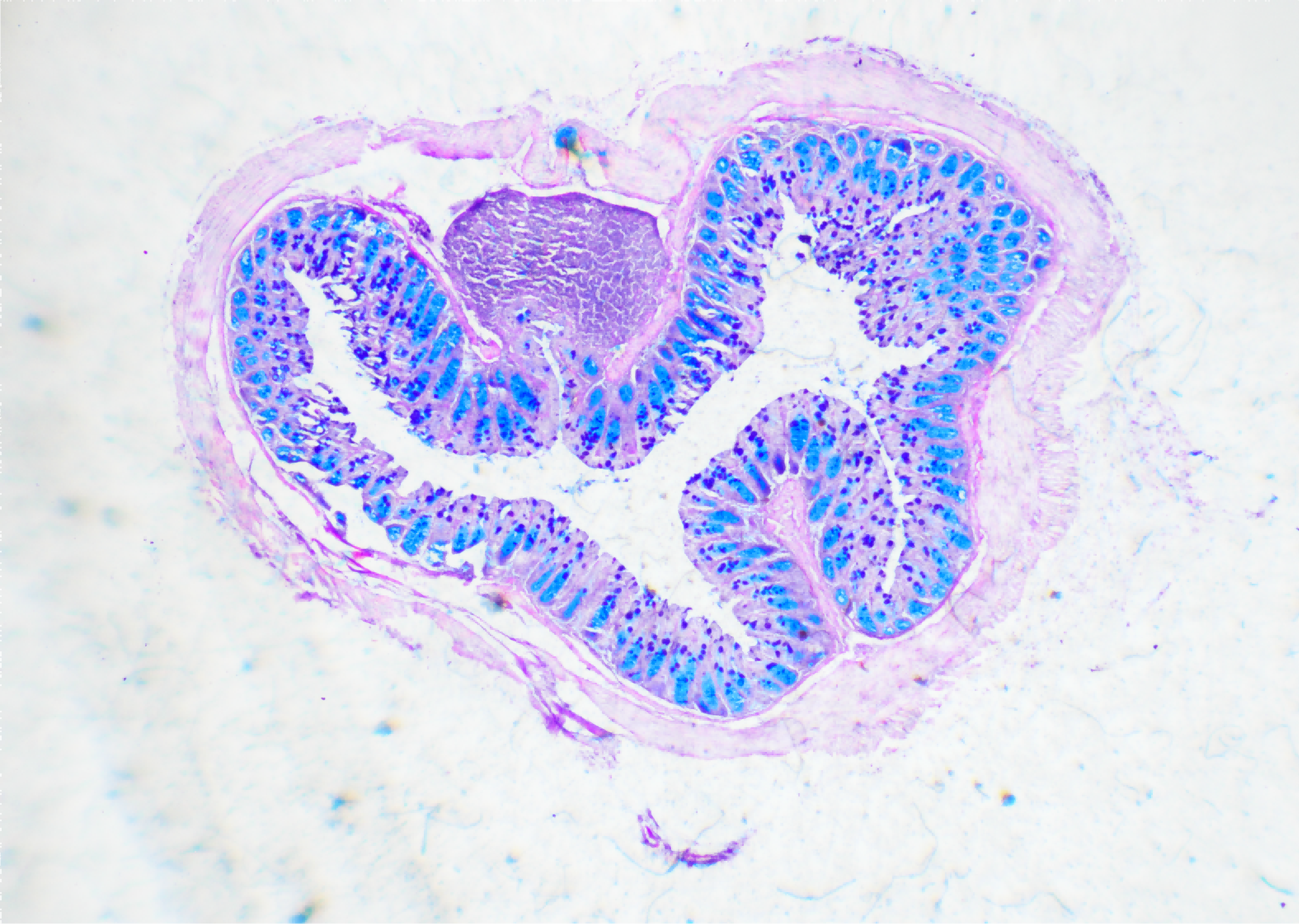

Supplement: Supplementary file 6 — Source data Fig. 1 [file 44319_2024_276_MOESM6_ESM.zip › Fig 1/1M/PAS_AB staining/Yod1--_water-40×.png]

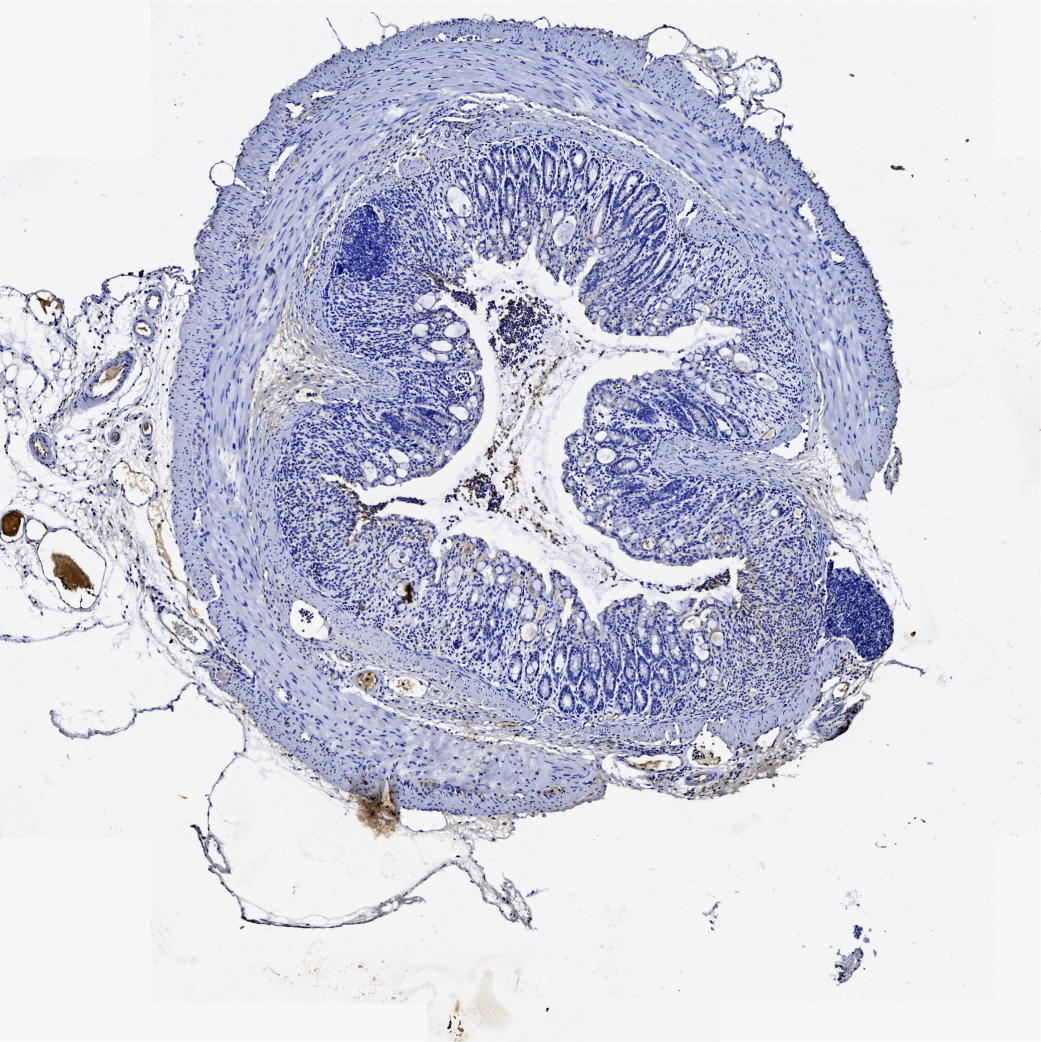

Supplement: Supplementary file 7 — Source data Fig. 2 [file 44319_2024_276_MOESM7_ESM.zip › Fig 2/2G/Yod1++_DSS_overall view.png]

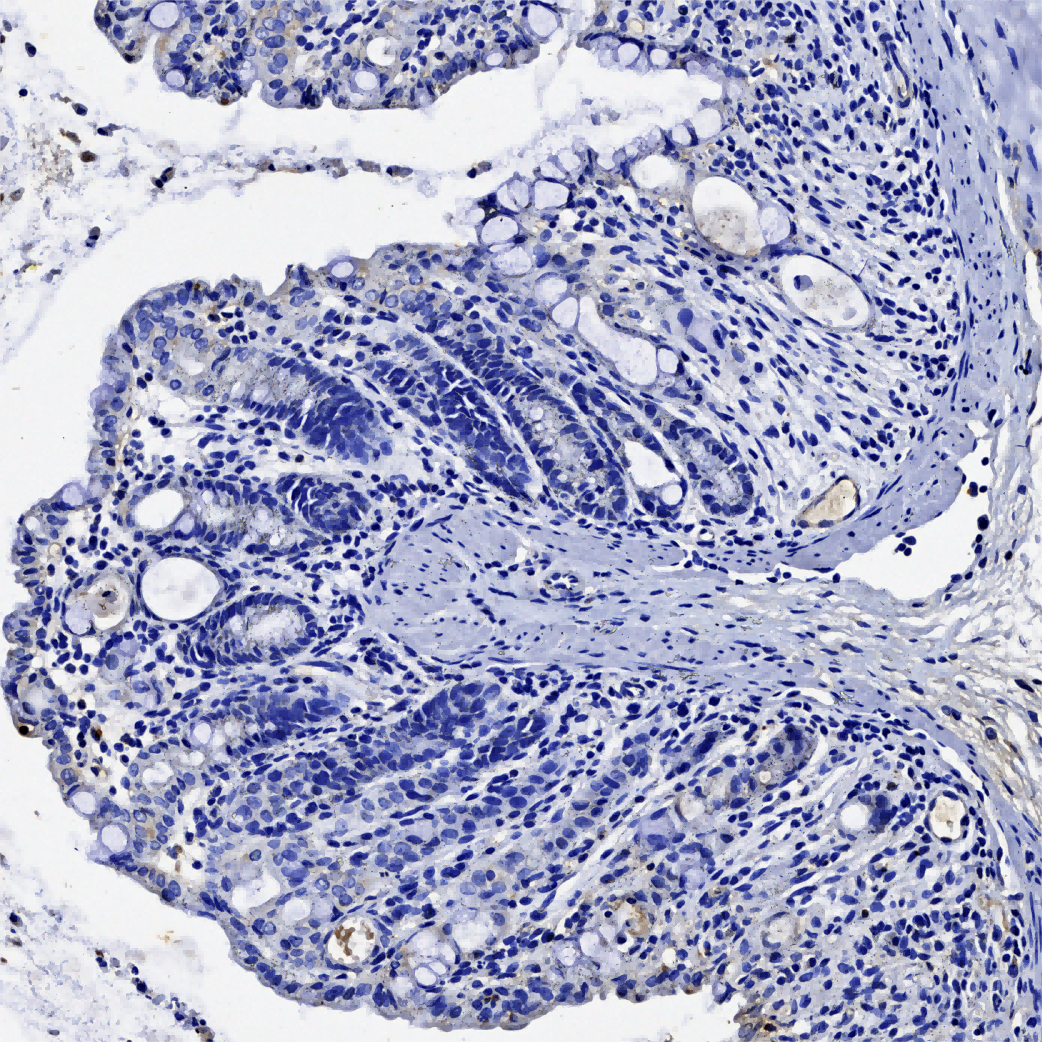

Supplement: Supplementary file 7 — Source data Fig. 2 [file 44319_2024_276_MOESM7_ESM.zip › Fig 2/2G/Yod1++_DSS_partial view.png]

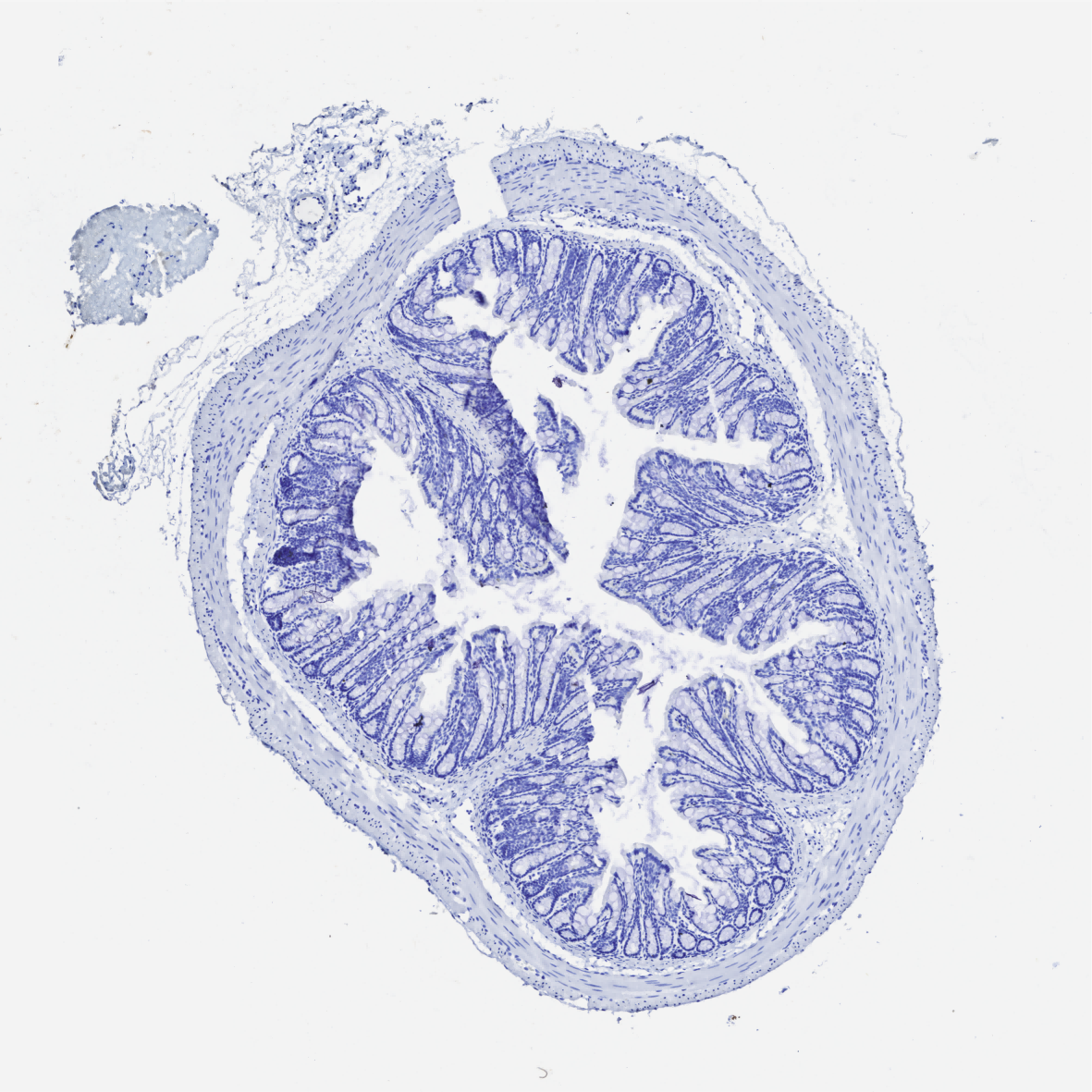

Supplement: Supplementary file 7 — Source data Fig. 2 [file 44319_2024_276_MOESM7_ESM.zip › Fig 2/2G/Yod1++_water_overall view.png]

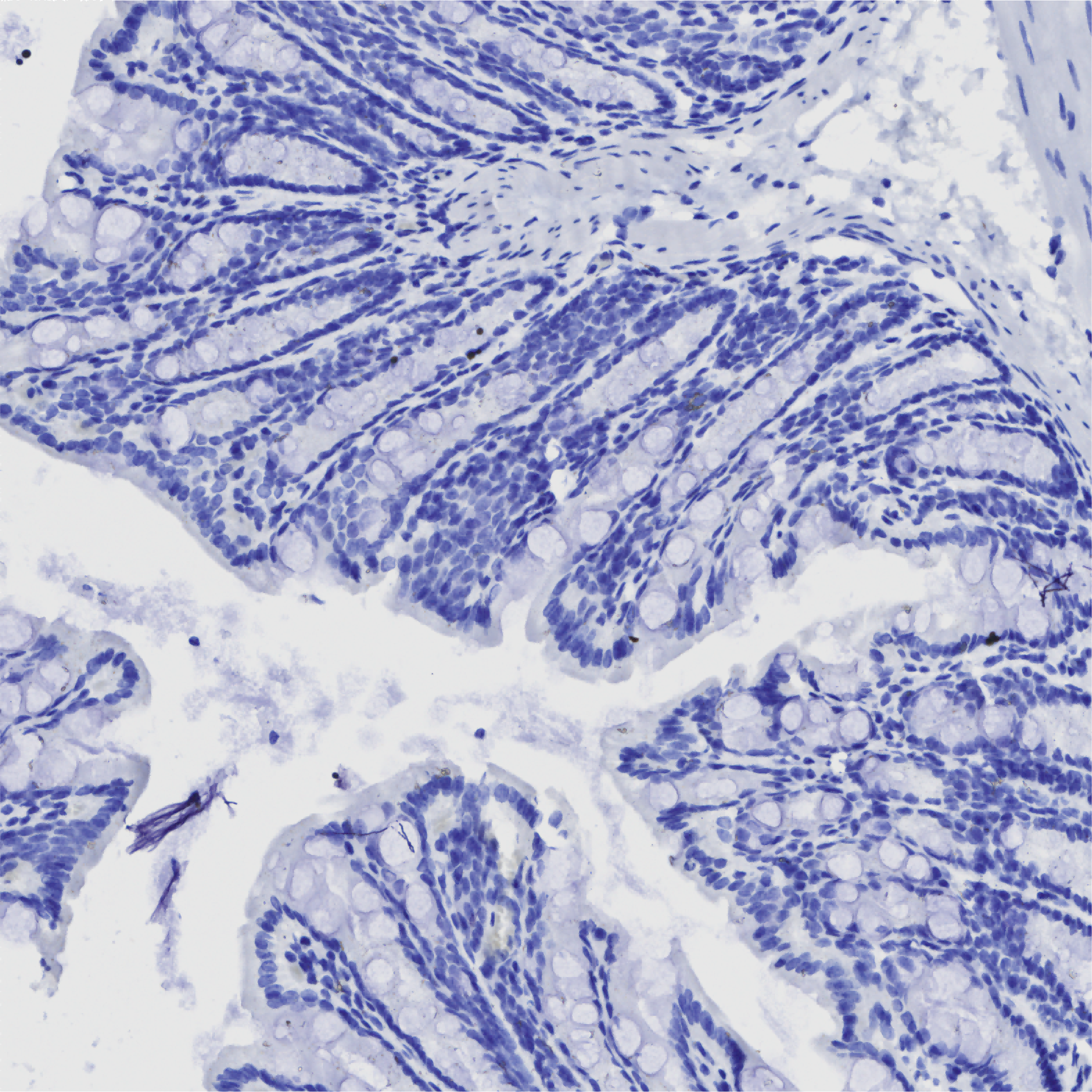

Supplement: Supplementary file 7 — Source data Fig. 2 [file 44319_2024_276_MOESM7_ESM.zip › Fig 2/2G/Yod1++_water_partial view.png]

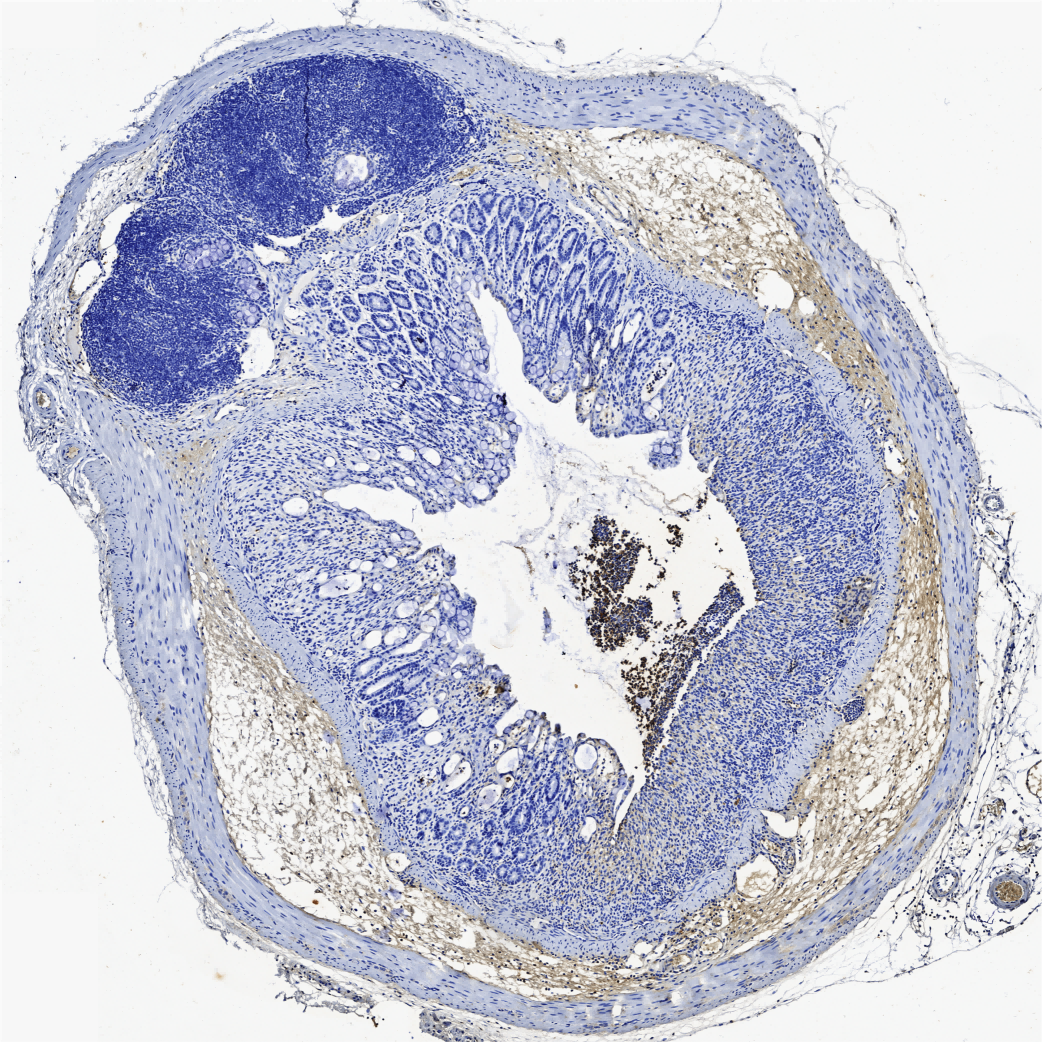

Supplement: Supplementary file 7 — Source data Fig. 2 [file 44319_2024_276_MOESM7_ESM.zip › Fig 2/2G/Yod1--_DSS_overall view.png]

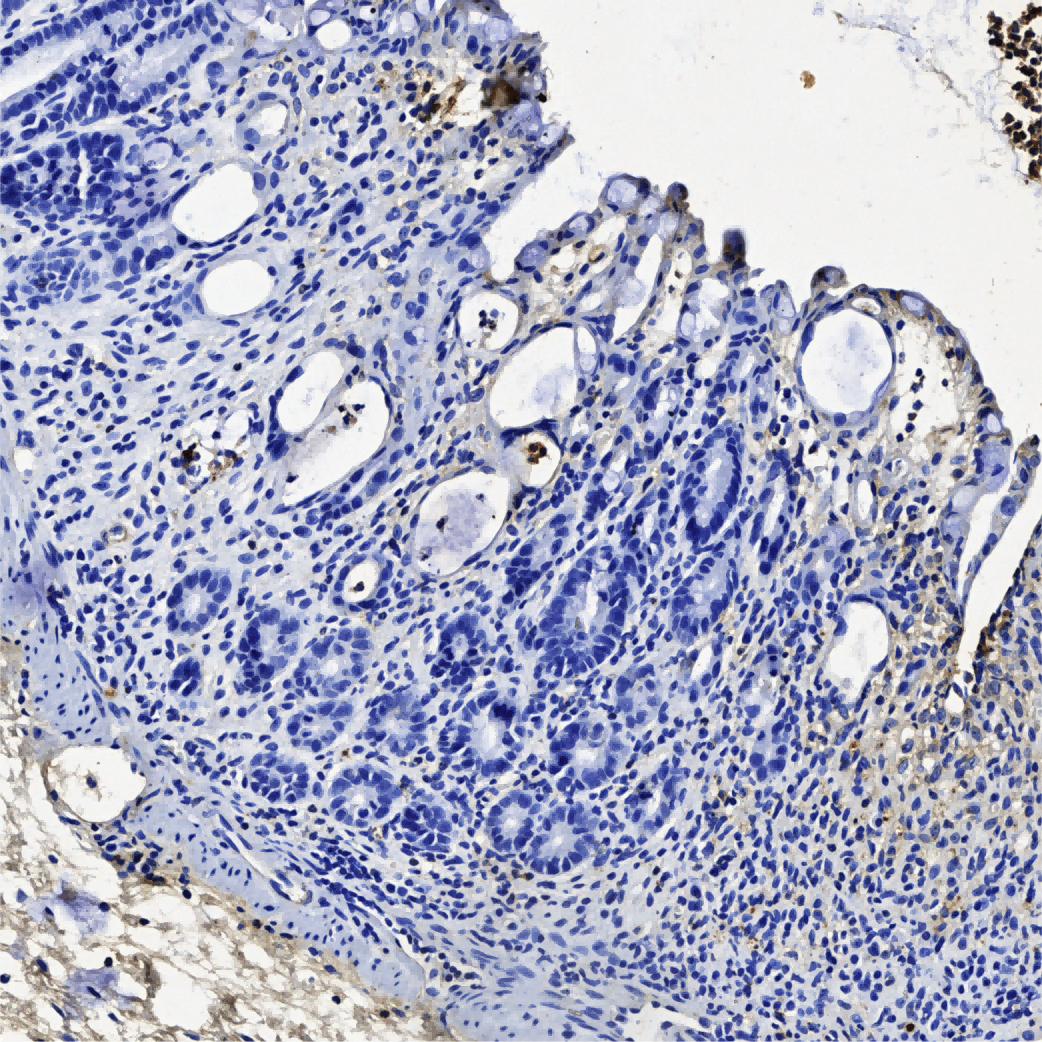

Supplement: Supplementary file 7 — Source data Fig. 2 [file 44319_2024_276_MOESM7_ESM.zip › Fig 2/2G/Yod1--_DSS_partial view.png]

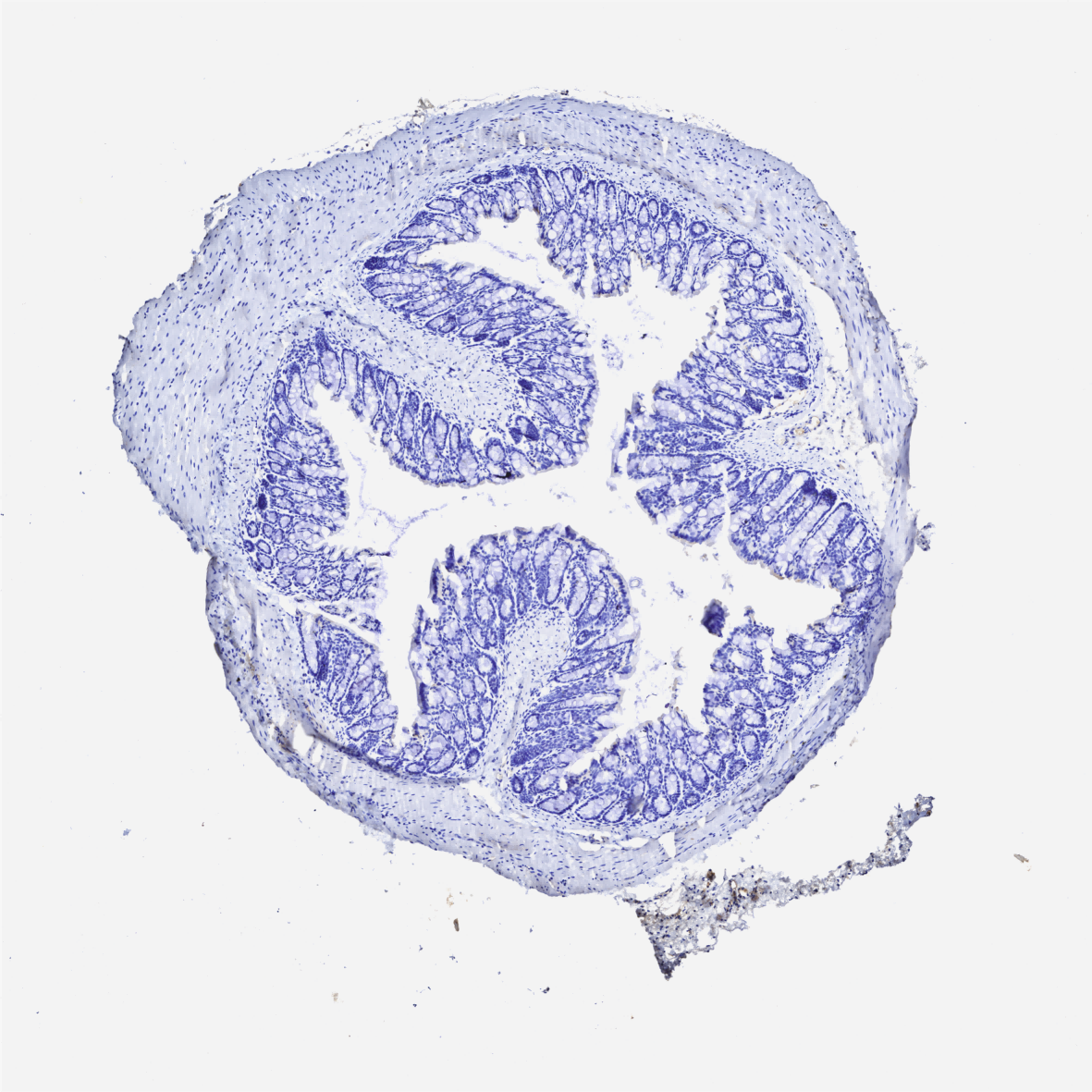

Supplement: Supplementary file 7 — Source data Fig. 2 [file 44319_2024_276_MOESM7_ESM.zip › Fig 2/2G/Yod1--_water_overall view.png]

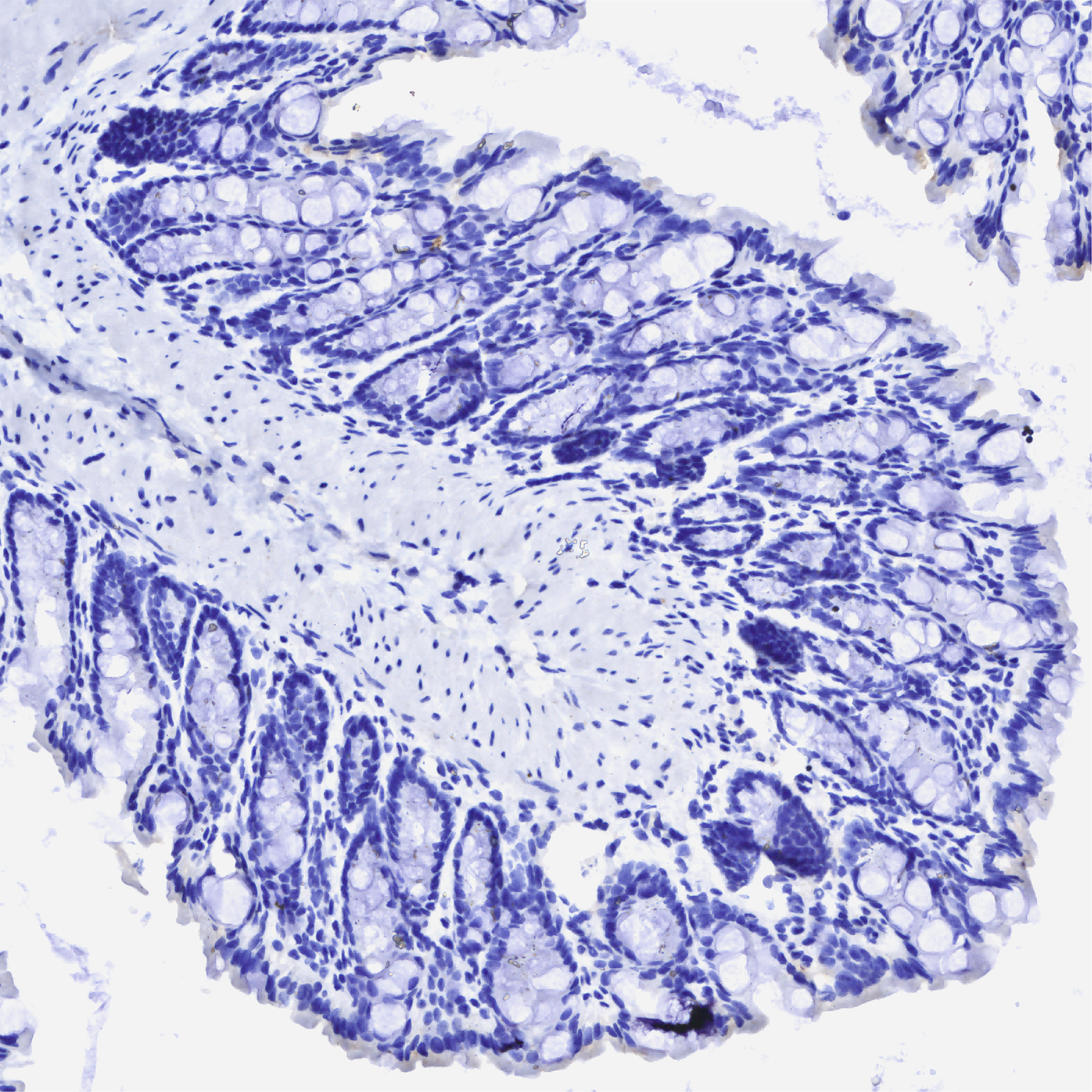

Supplement: Supplementary file 7 — Source data Fig. 2 [file 44319_2024_276_MOESM7_ESM.zip › Fig 2/2G/Yod1--_water_partial view.png]

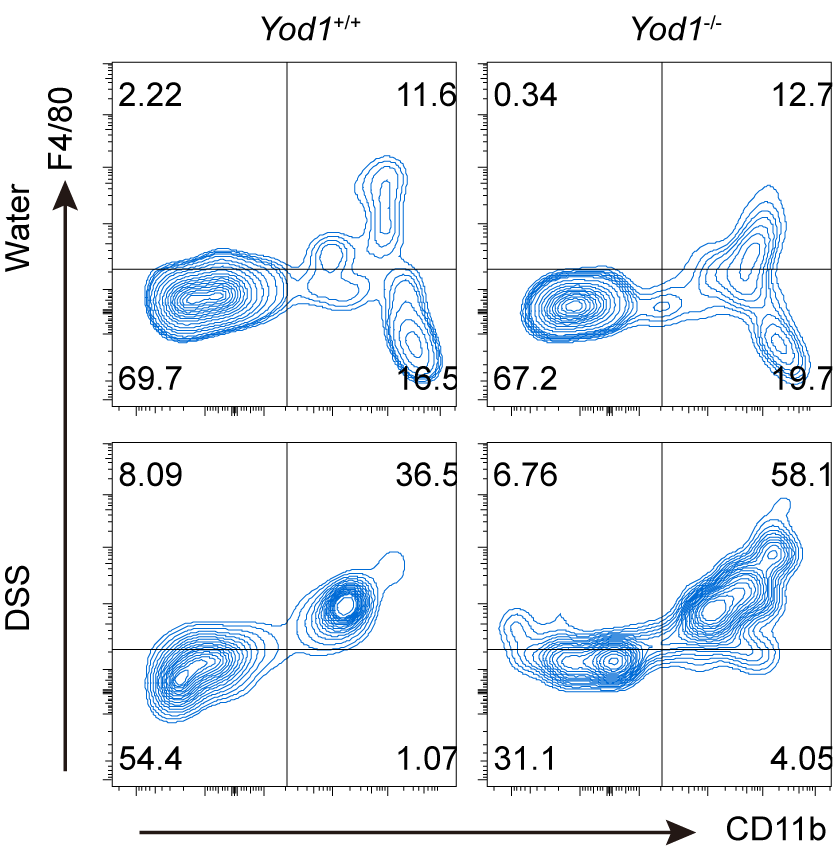

Supplement: Supplementary file 7 — Source data Fig. 2 [file 44319_2024_276_MOESM7_ESM.zip › Fig 2/2H/2H.tif]

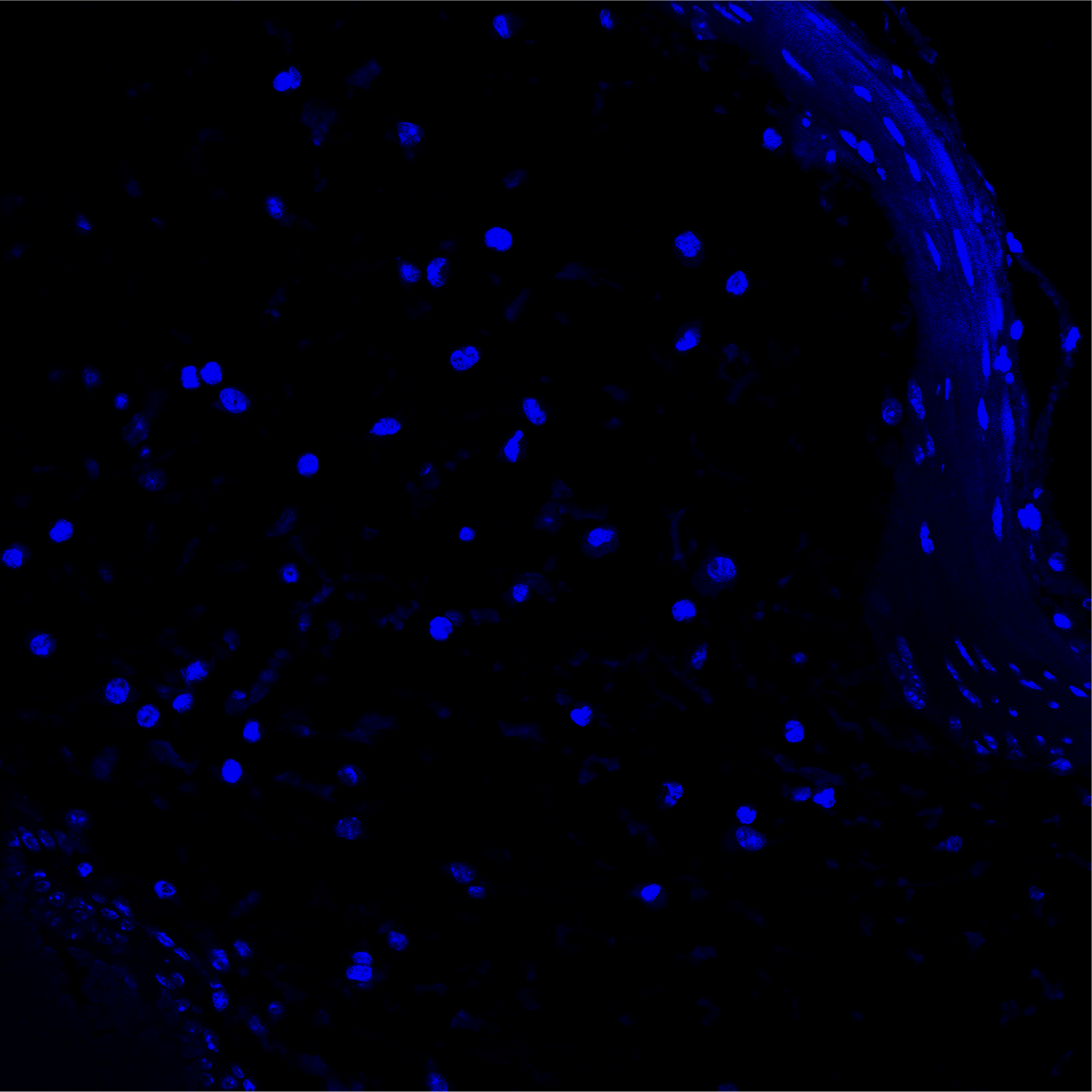

Supplement: Supplementary file 8 — Source data Fig. 3 [file 44319_2024_276_MOESM8_ESM.zip › Fig 3/3A/DSS_DAPI.png]

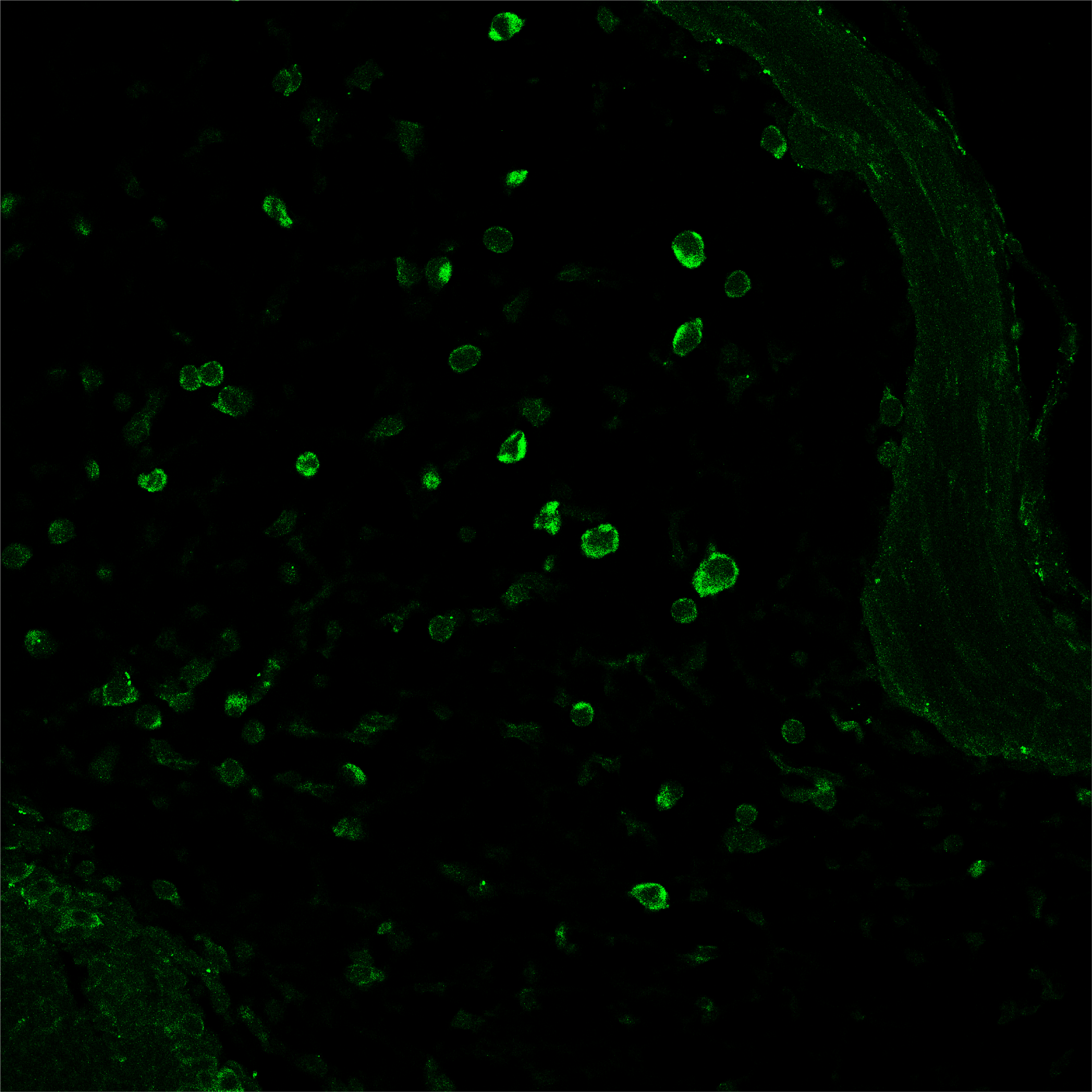

Supplement: Supplementary file 8 — Source data Fig. 3 [file 44319_2024_276_MOESM8_ESM.zip › Fig 3/3A/DSS_F4_80.png]

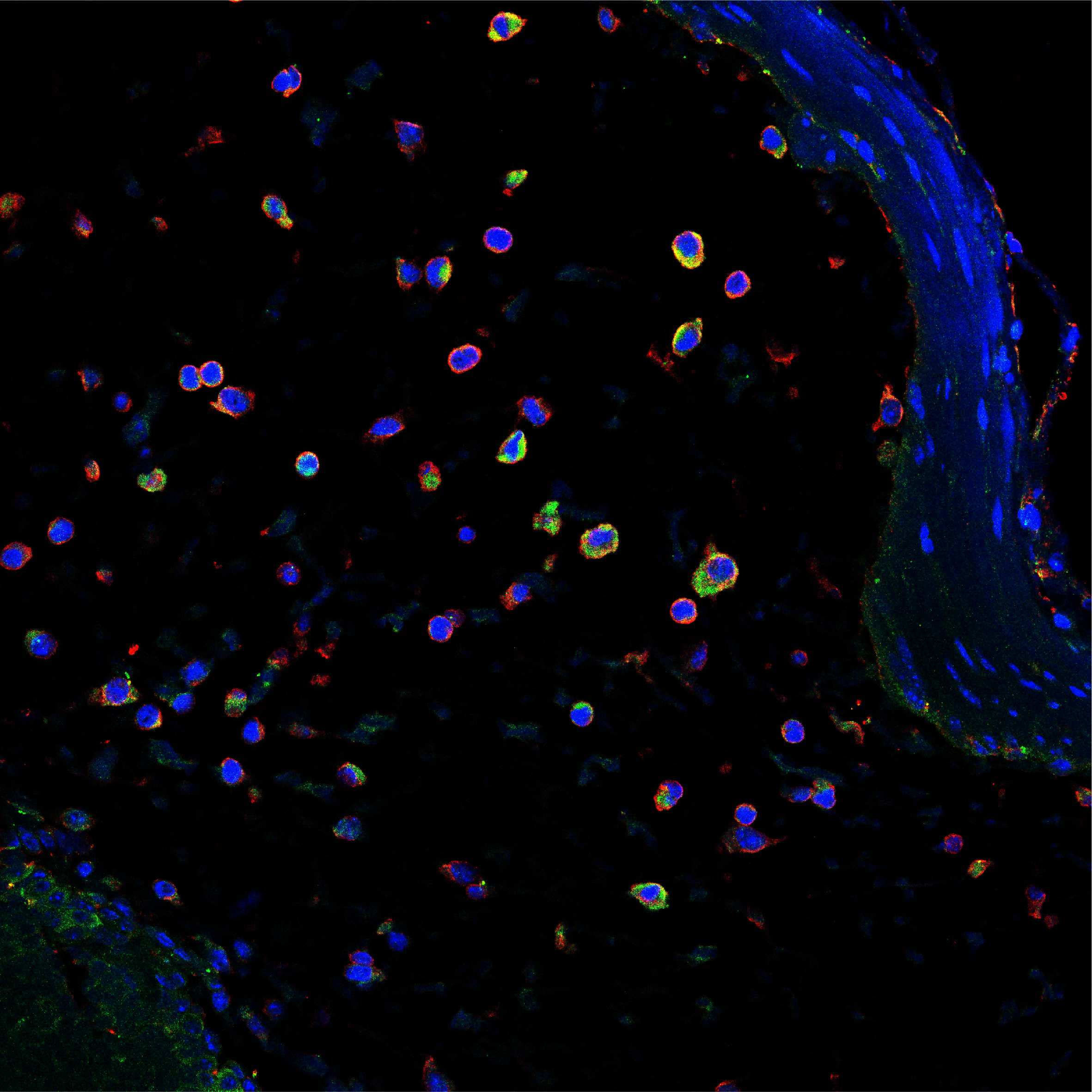

Supplement: Supplementary file 8 — Source data Fig. 3 [file 44319_2024_276_MOESM8_ESM.zip › Fig 3/3A/DSS_Merge.png]

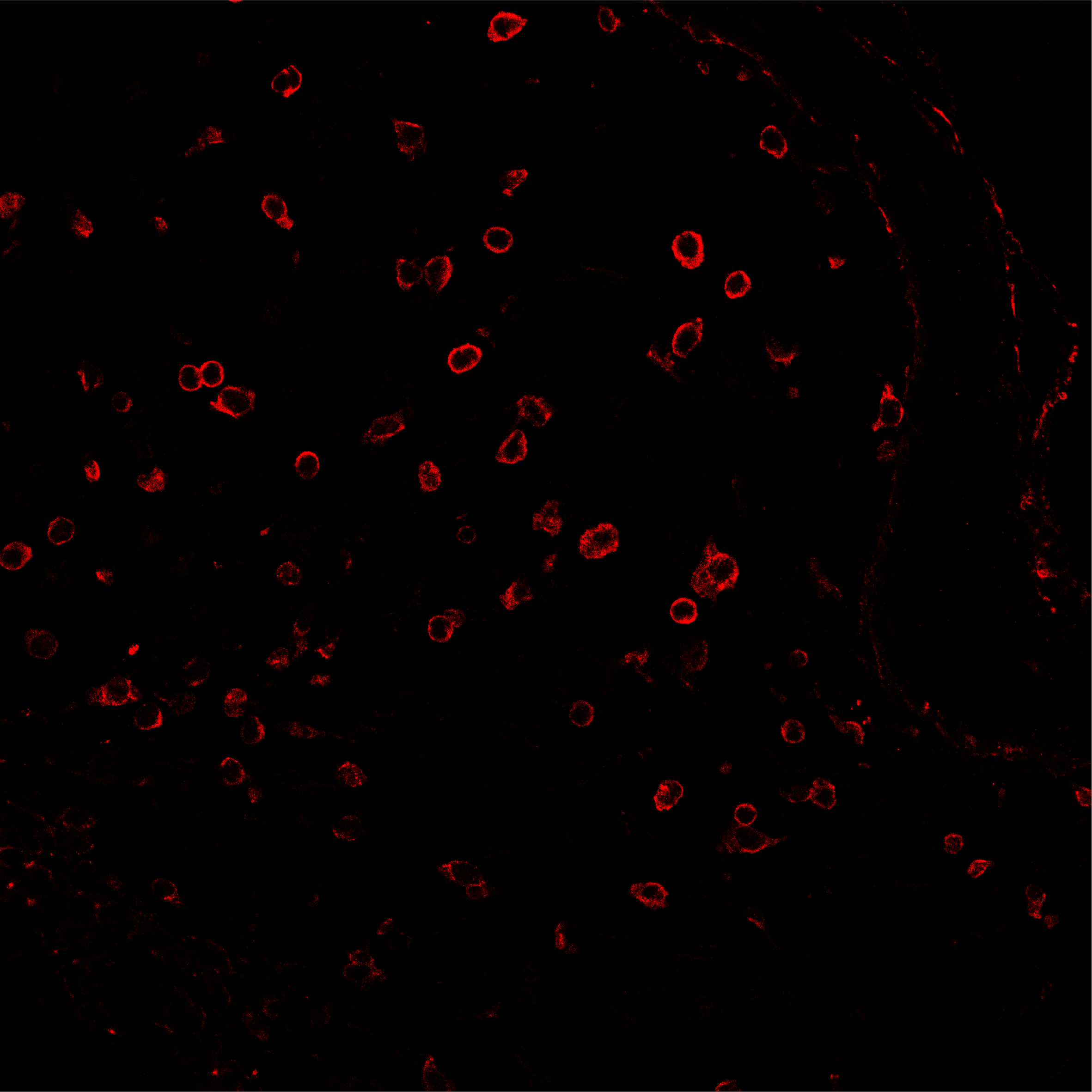

Supplement: Supplementary file 8 — Source data Fig. 3 [file 44319_2024_276_MOESM8_ESM.zip › Fig 3/3A/DSS_YOD1.png]

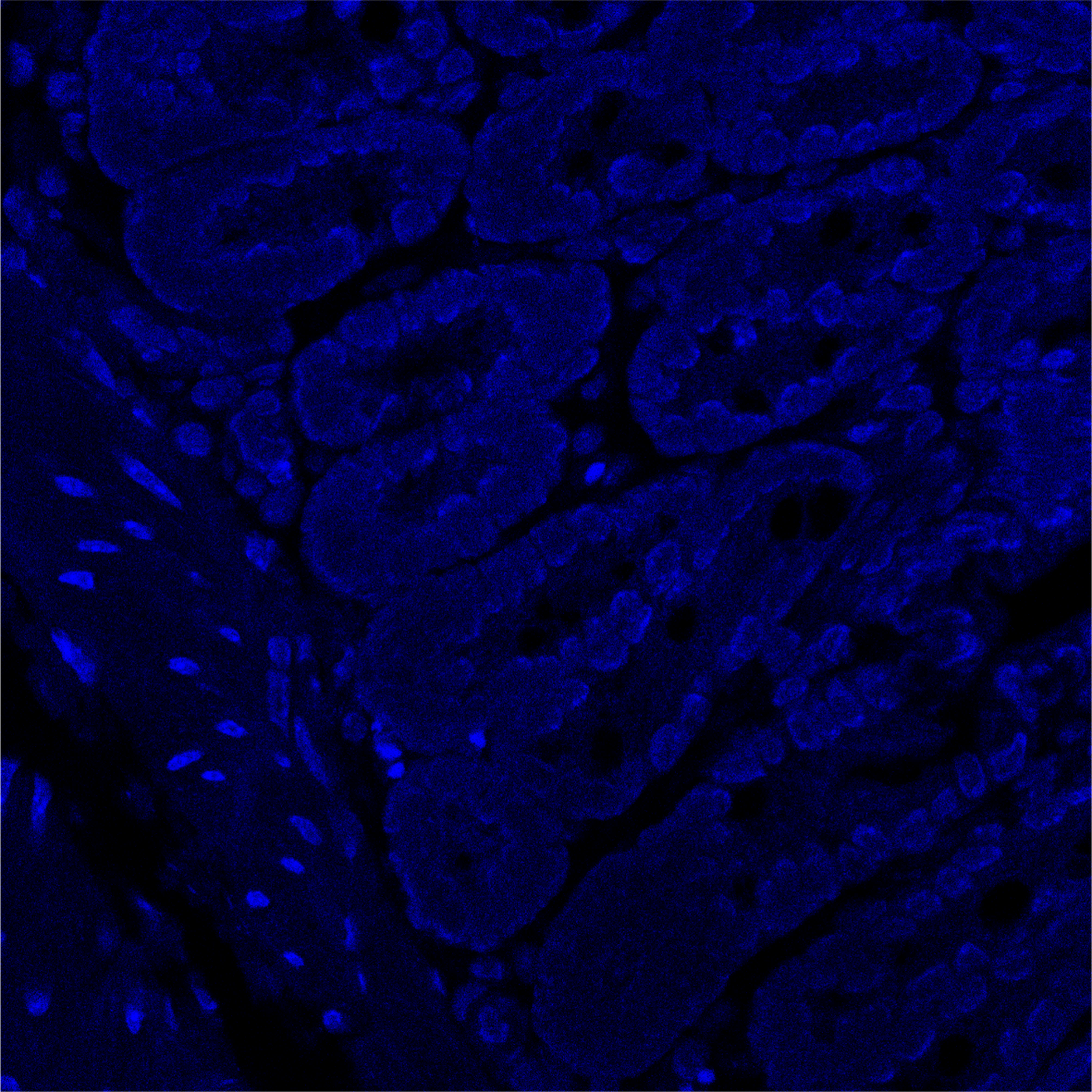

Supplement: Supplementary file 8 — Source data Fig. 3 [file 44319_2024_276_MOESM8_ESM.zip › Fig 3/3A/Water_DAPI.png]

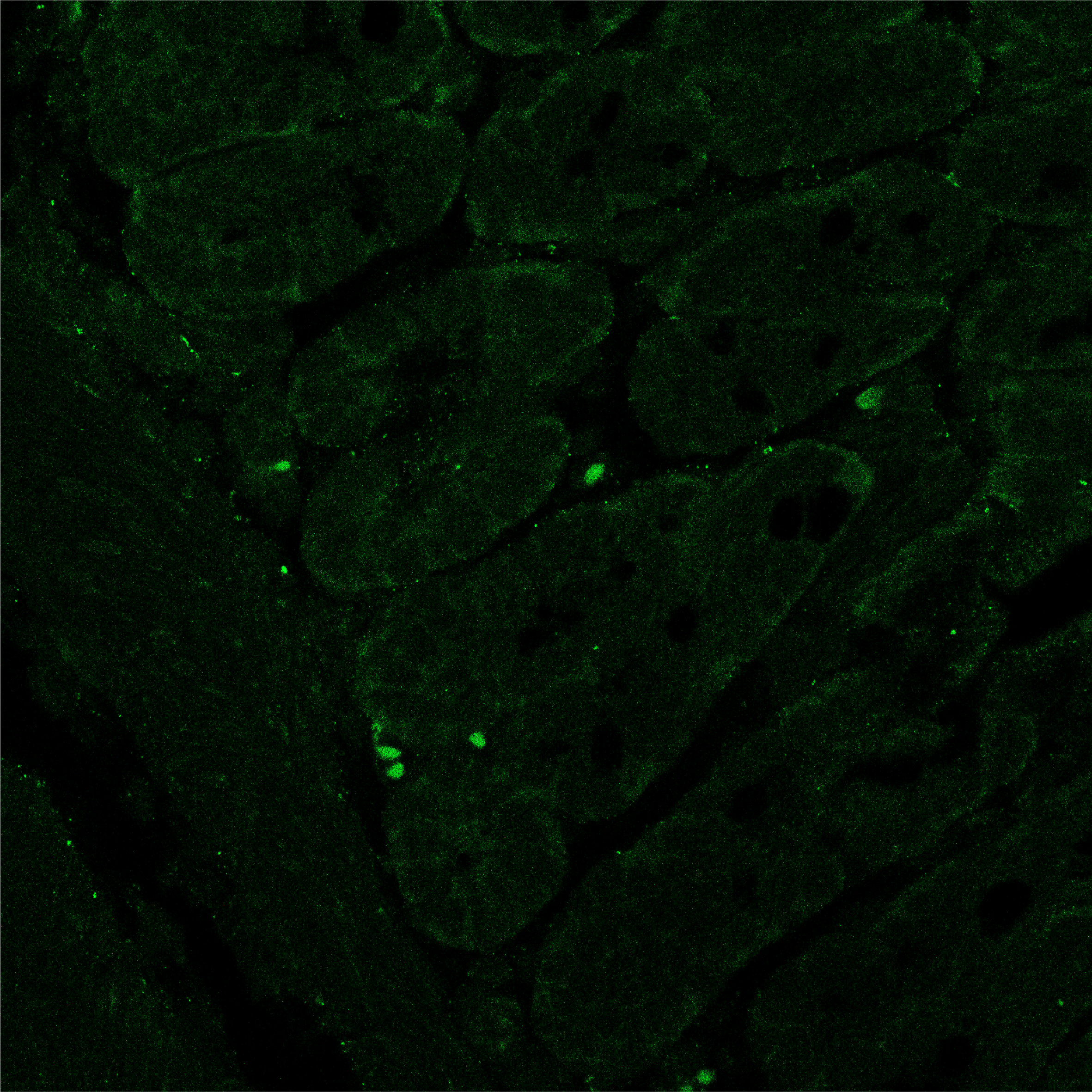

Supplement: Supplementary file 8 — Source data Fig. 3 [file 44319_2024_276_MOESM8_ESM.zip › Fig 3/3A/Water_F4_80.png]

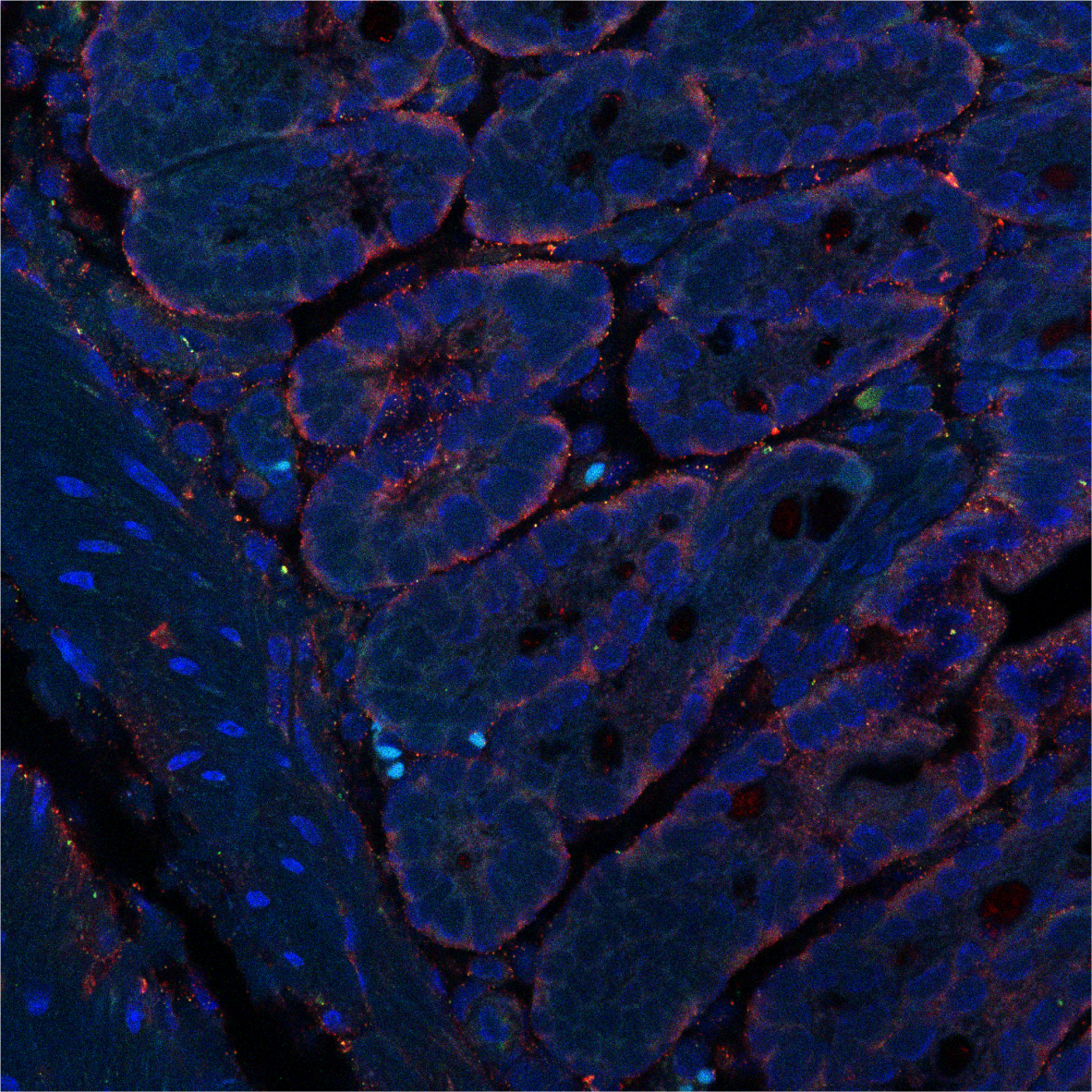

Supplement: Supplementary file 8 — Source data Fig. 3 [file 44319_2024_276_MOESM8_ESM.zip › Fig 3/3A/Water_Merge.png]

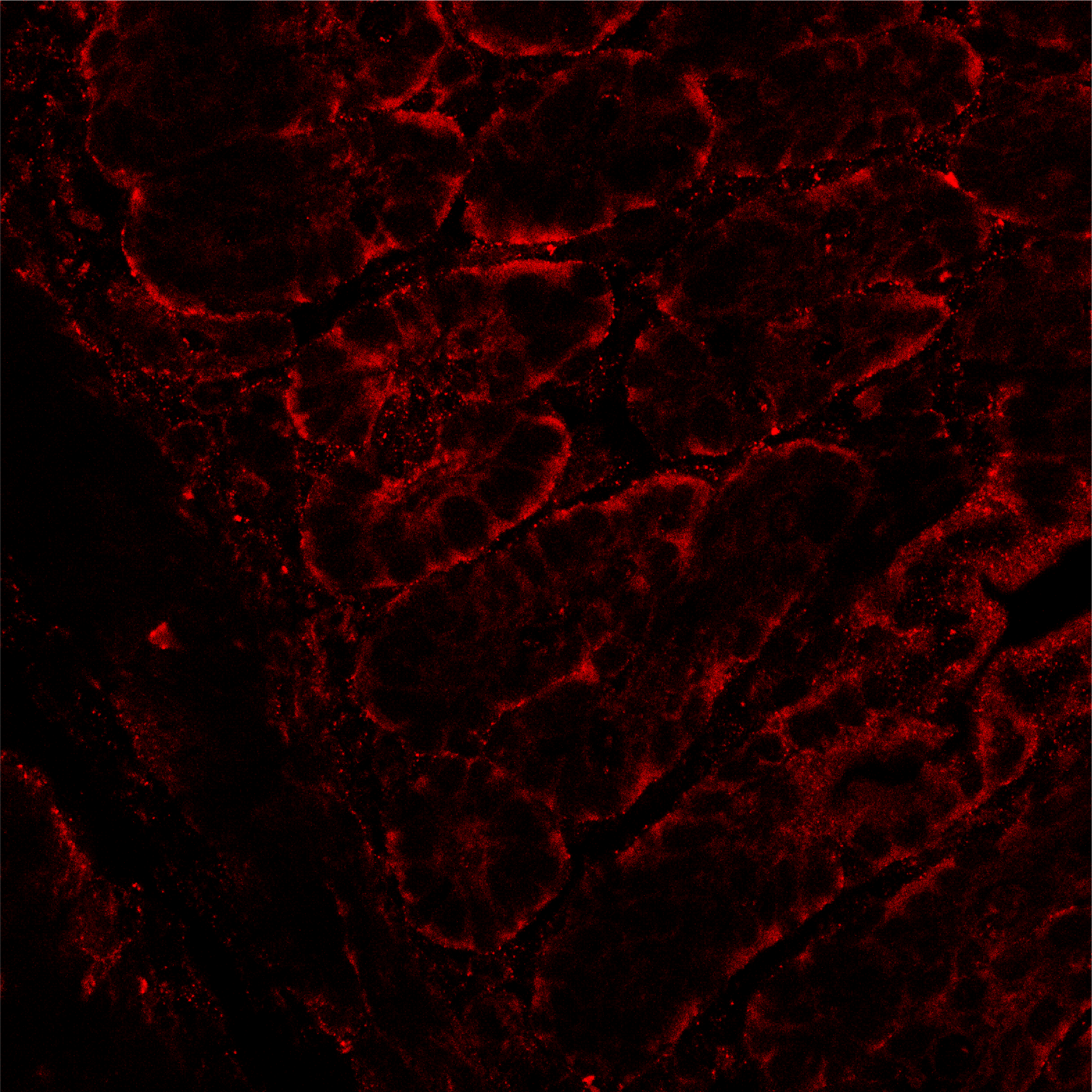

Supplement: Supplementary file 8 — Source data Fig. 3 [file 44319_2024_276_MOESM8_ESM.zip › Fig 3/3A/Water_YOD1.png]

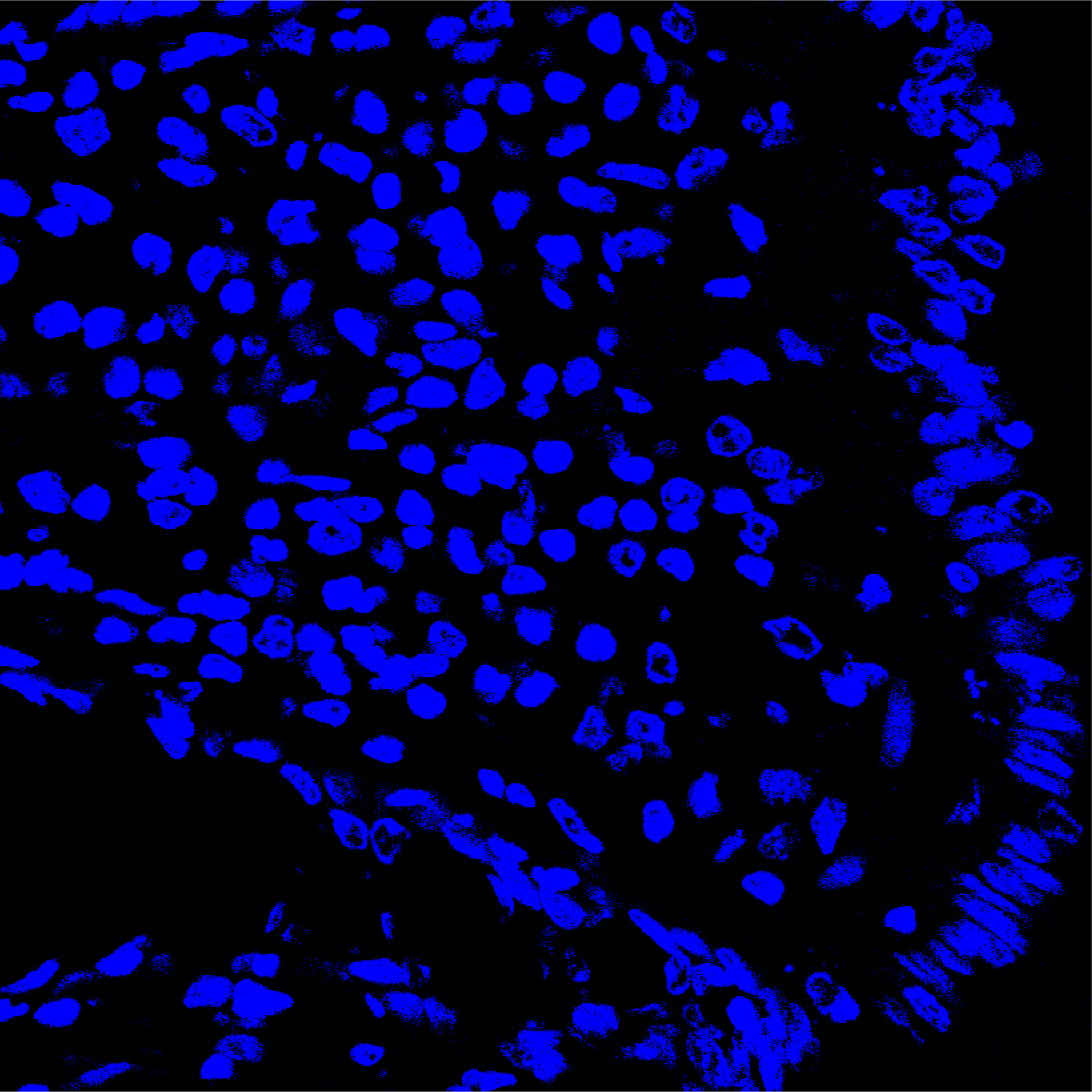

Supplement: Supplementary file 8 — Source data Fig. 3 [file 44319_2024_276_MOESM8_ESM.zip › Fig 3/3B/Control_DAPI.tif]

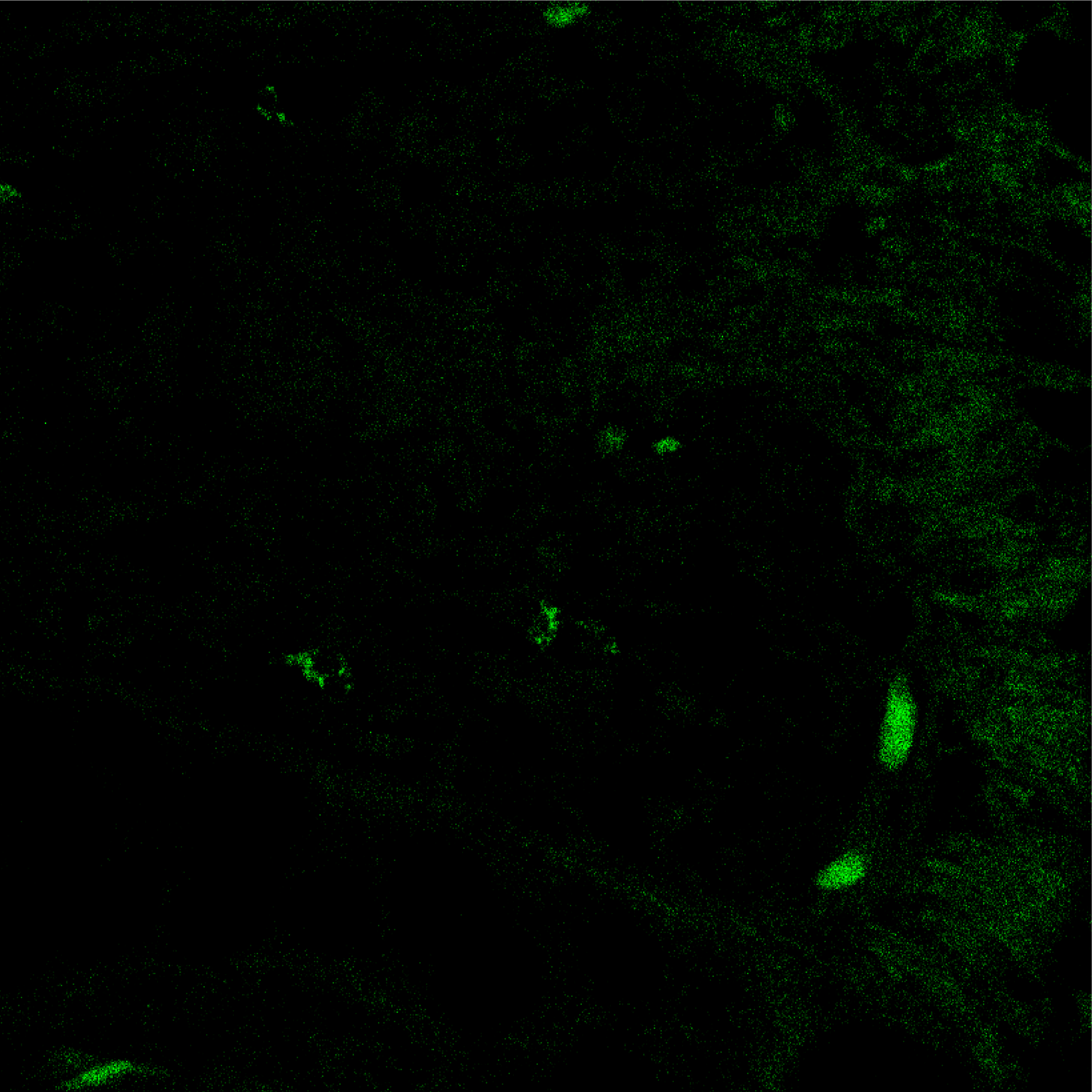

Supplement: Supplementary file 8 — Source data Fig. 3 [file 44319_2024_276_MOESM8_ESM.zip › Fig 3/3B/Control_F4_80.png]

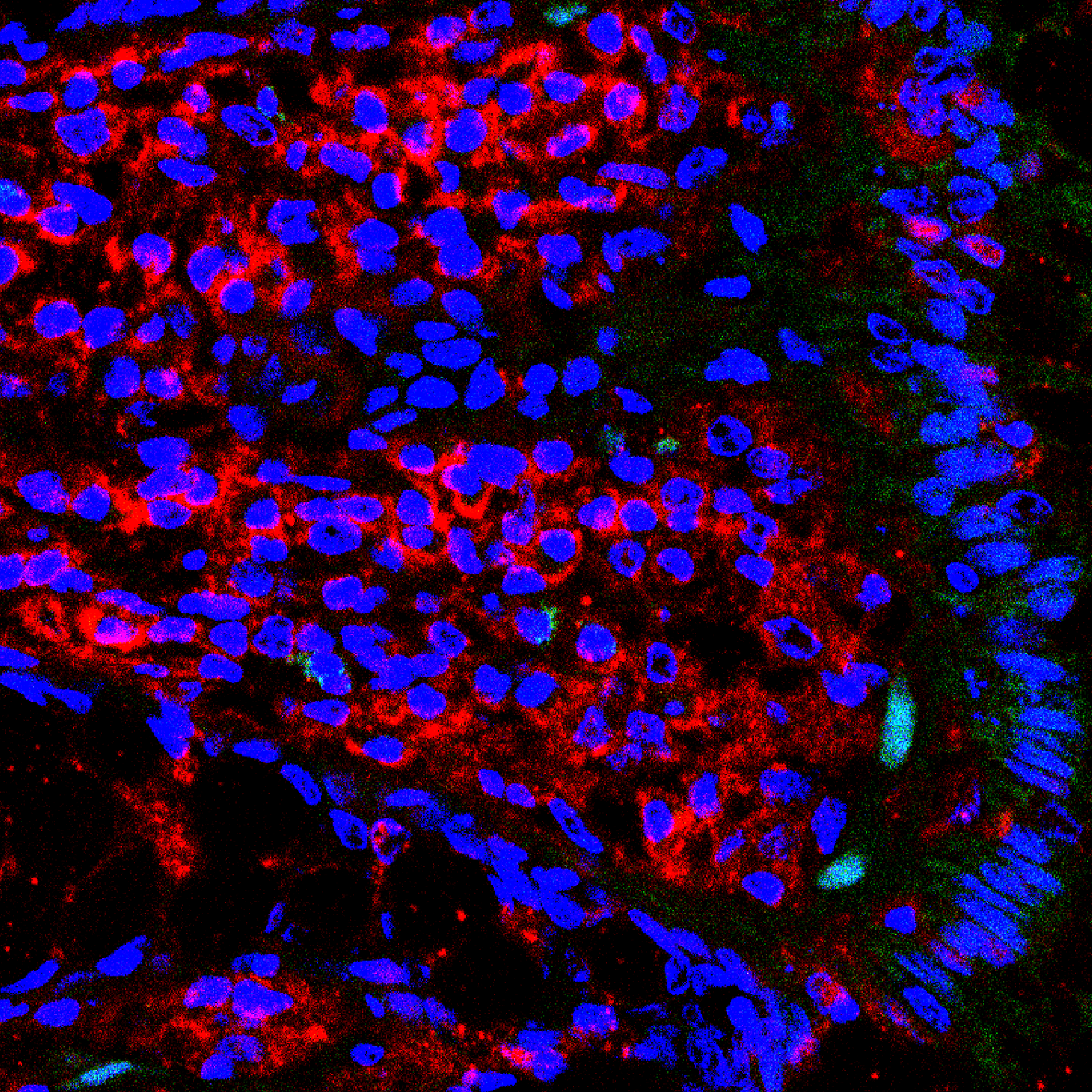

Supplement: Supplementary file 8 — Source data Fig. 3 [file 44319_2024_276_MOESM8_ESM.zip › Fig 3/3B/Control_Merge.png]

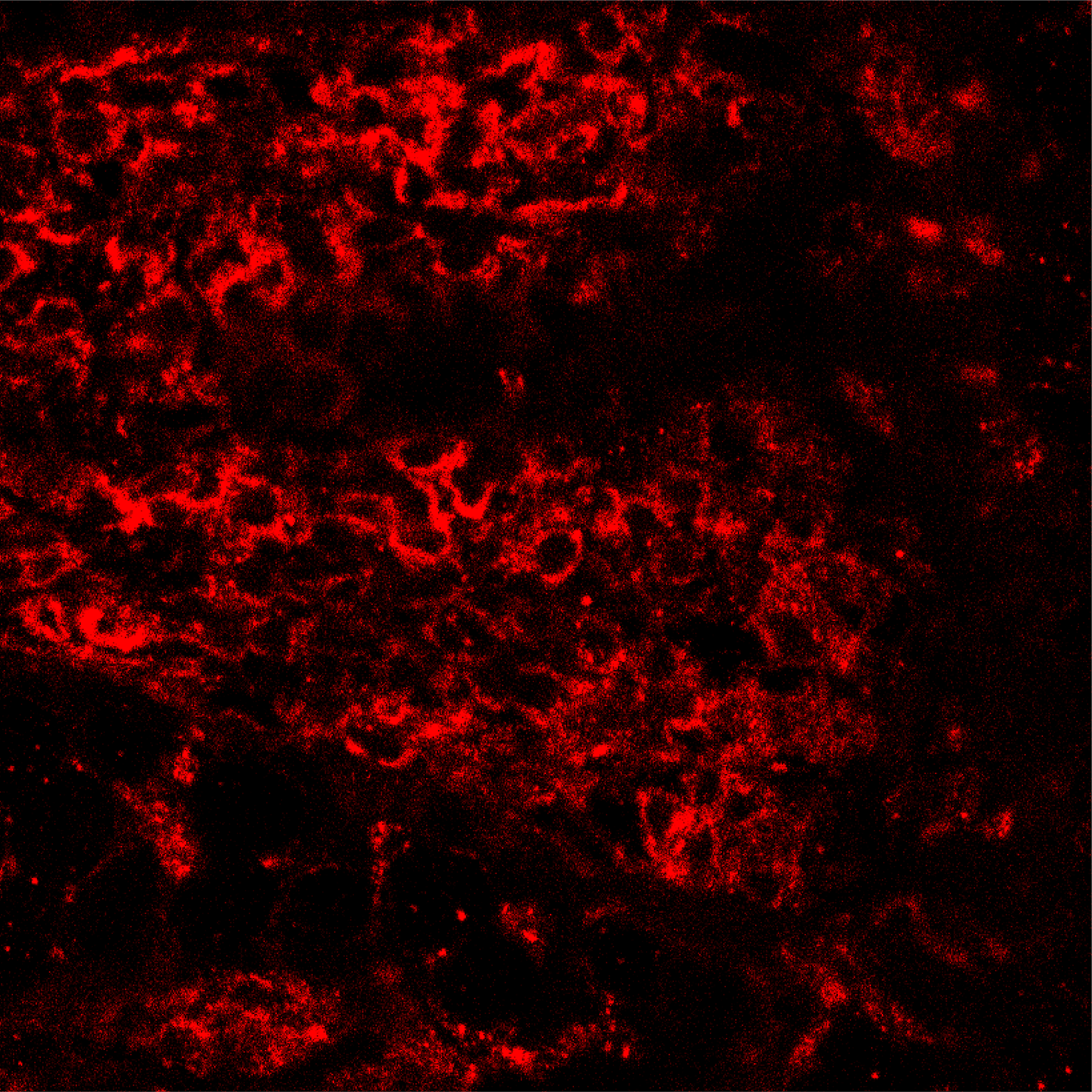

Supplement: Supplementary file 8 — Source data Fig. 3 [file 44319_2024_276_MOESM8_ESM.zip › Fig 3/3B/Control_YOD1.png]

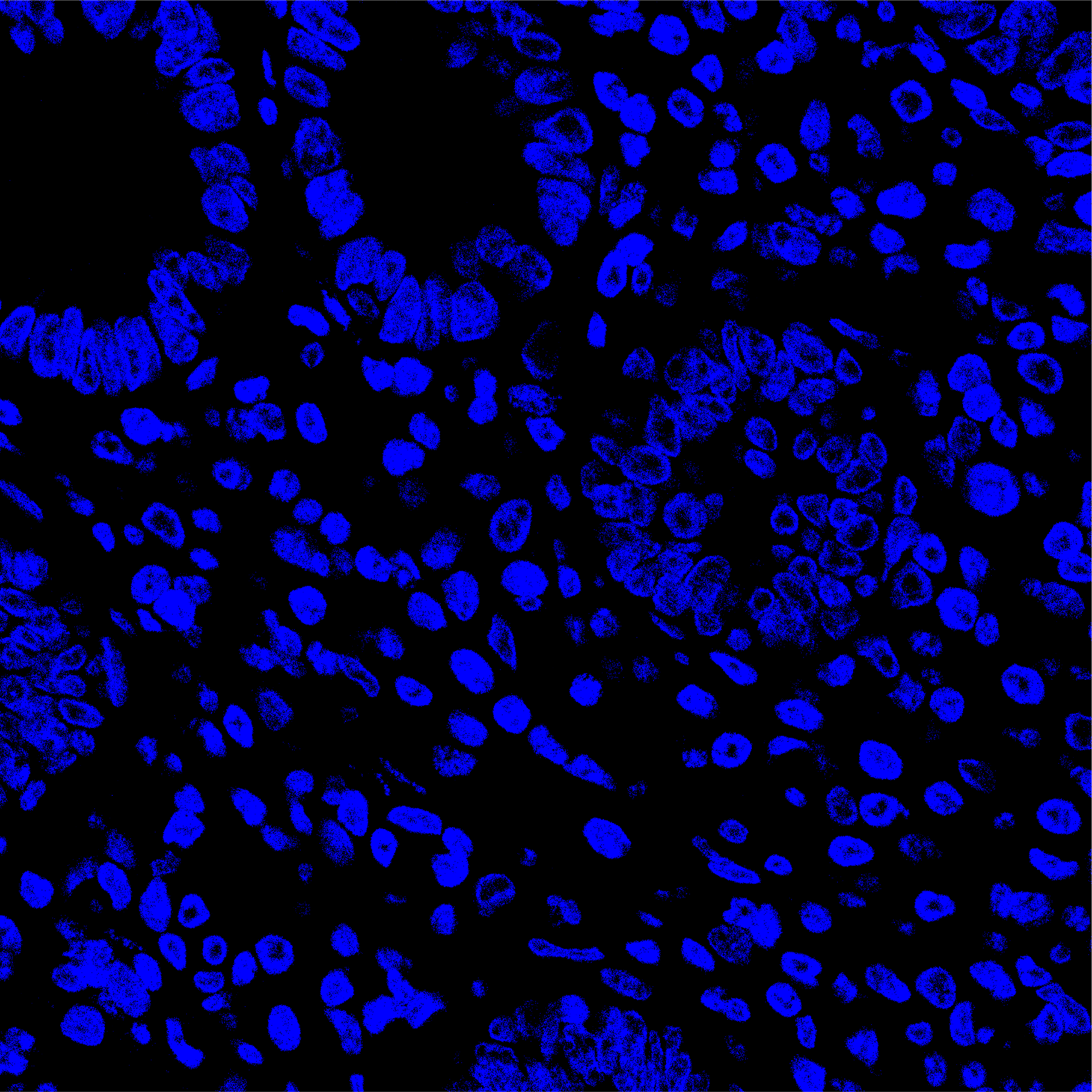

Supplement: Supplementary file 8 — Source data Fig. 3 [file 44319_2024_276_MOESM8_ESM.zip › Fig 3/3B/UC_DAPI.png]

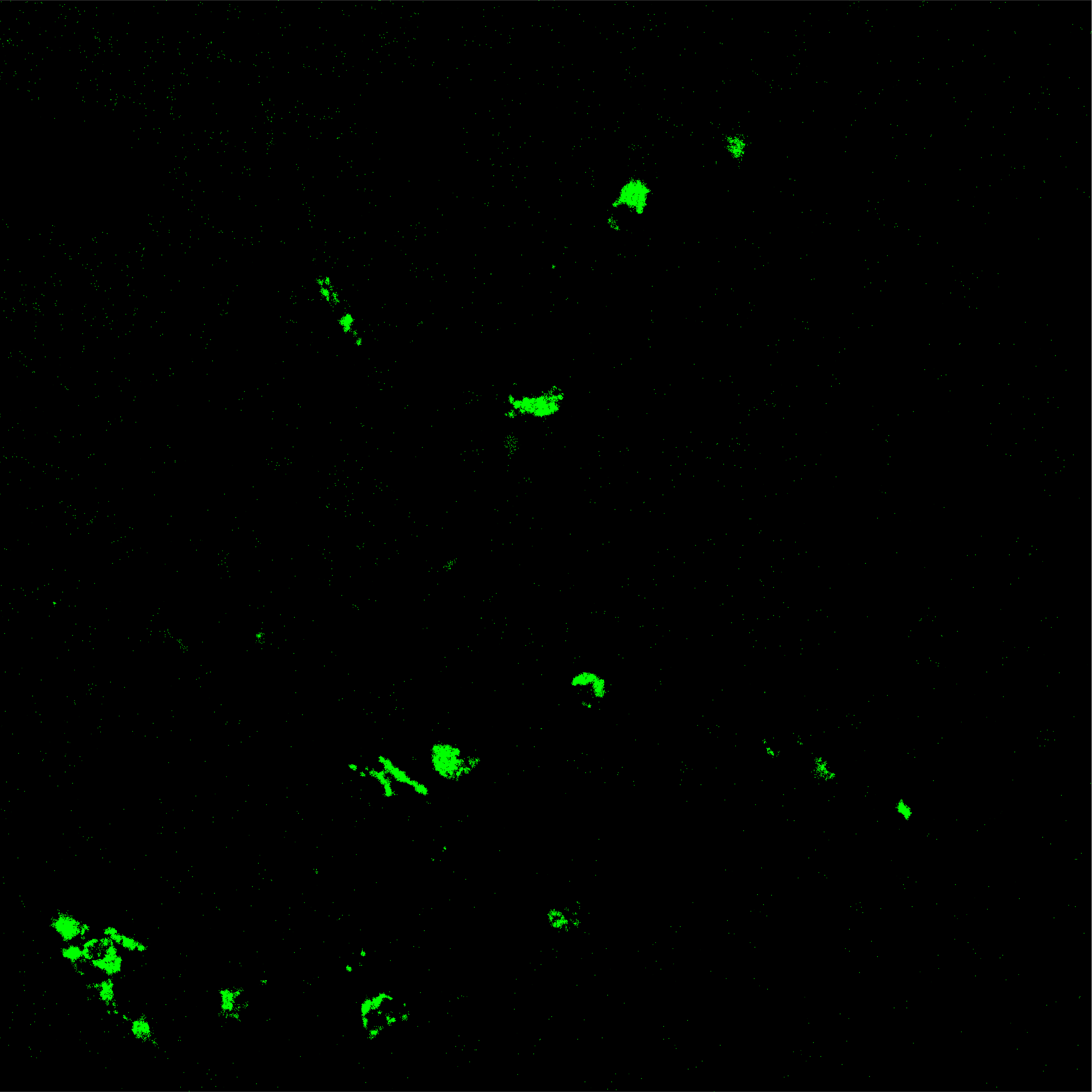

Supplement: Supplementary file 8 — Source data Fig. 3 [file 44319_2024_276_MOESM8_ESM.zip › Fig 3/3B/UC_F4_80.tif]

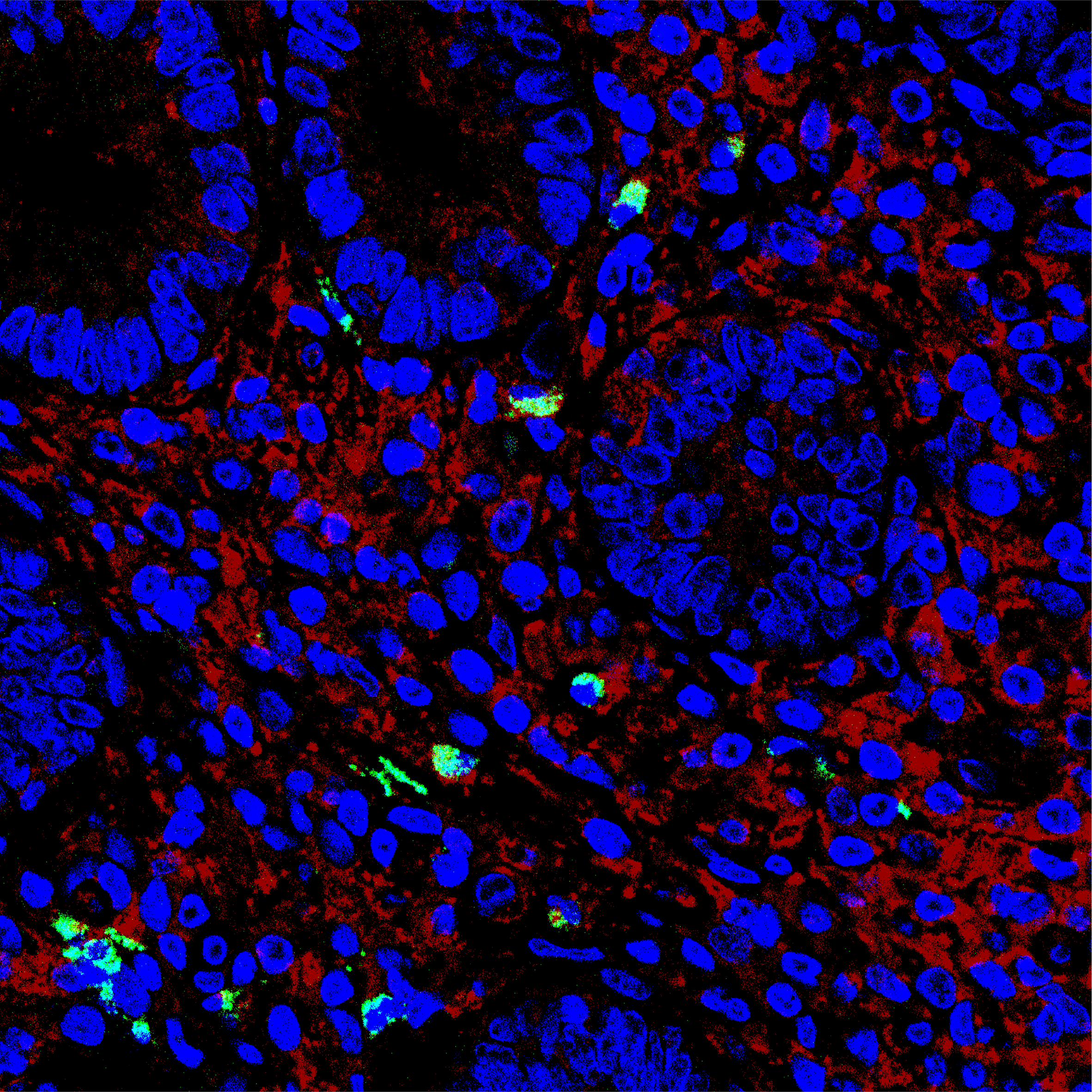

Supplement: Supplementary file 8 — Source data Fig. 3 [file 44319_2024_276_MOESM8_ESM.zip › Fig 3/3B/UC_Merge.png]

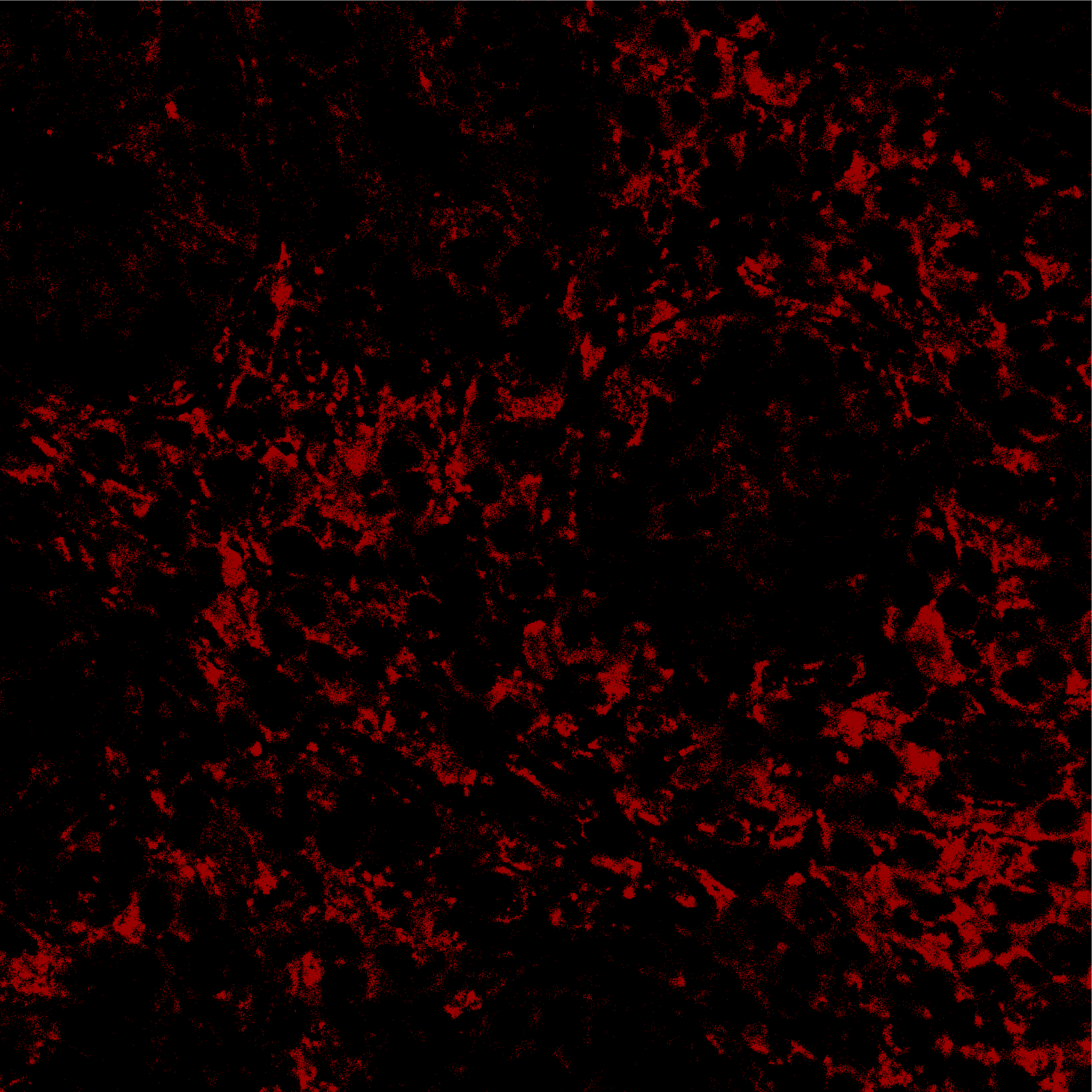

Supplement: Supplementary file 8 — Source data Fig. 3 [file 44319_2024_276_MOESM8_ESM.zip › Fig 3/3B/UC_YOD1.png]

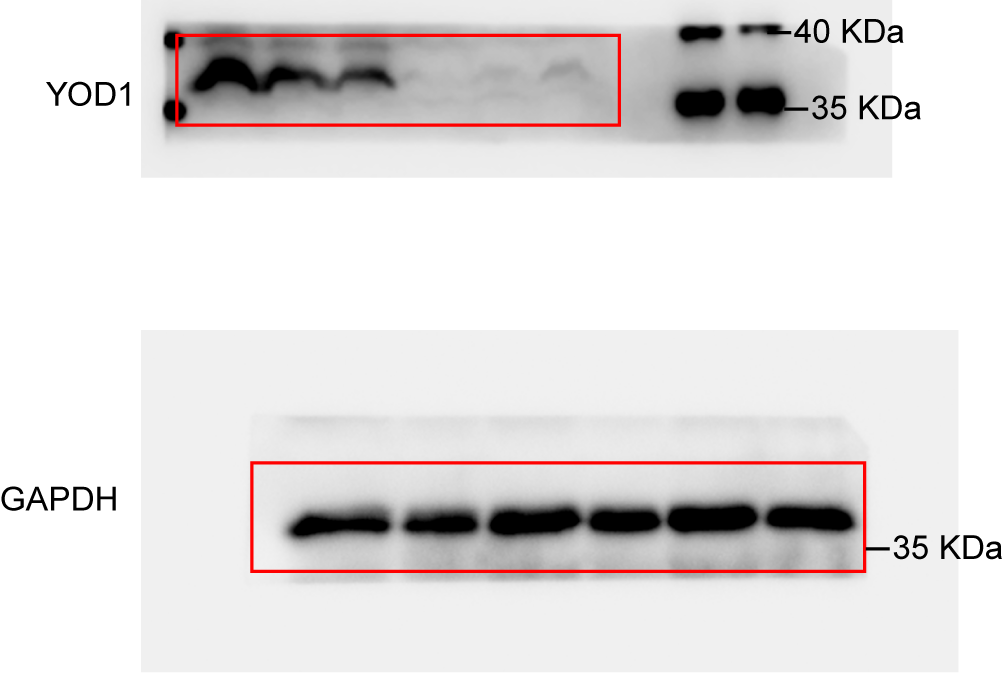

Supplement: Supplementary file 8 — Source data Fig. 3 [file 44319_2024_276_MOESM8_ESM.zip › Fig 3/3C/3C.tif]

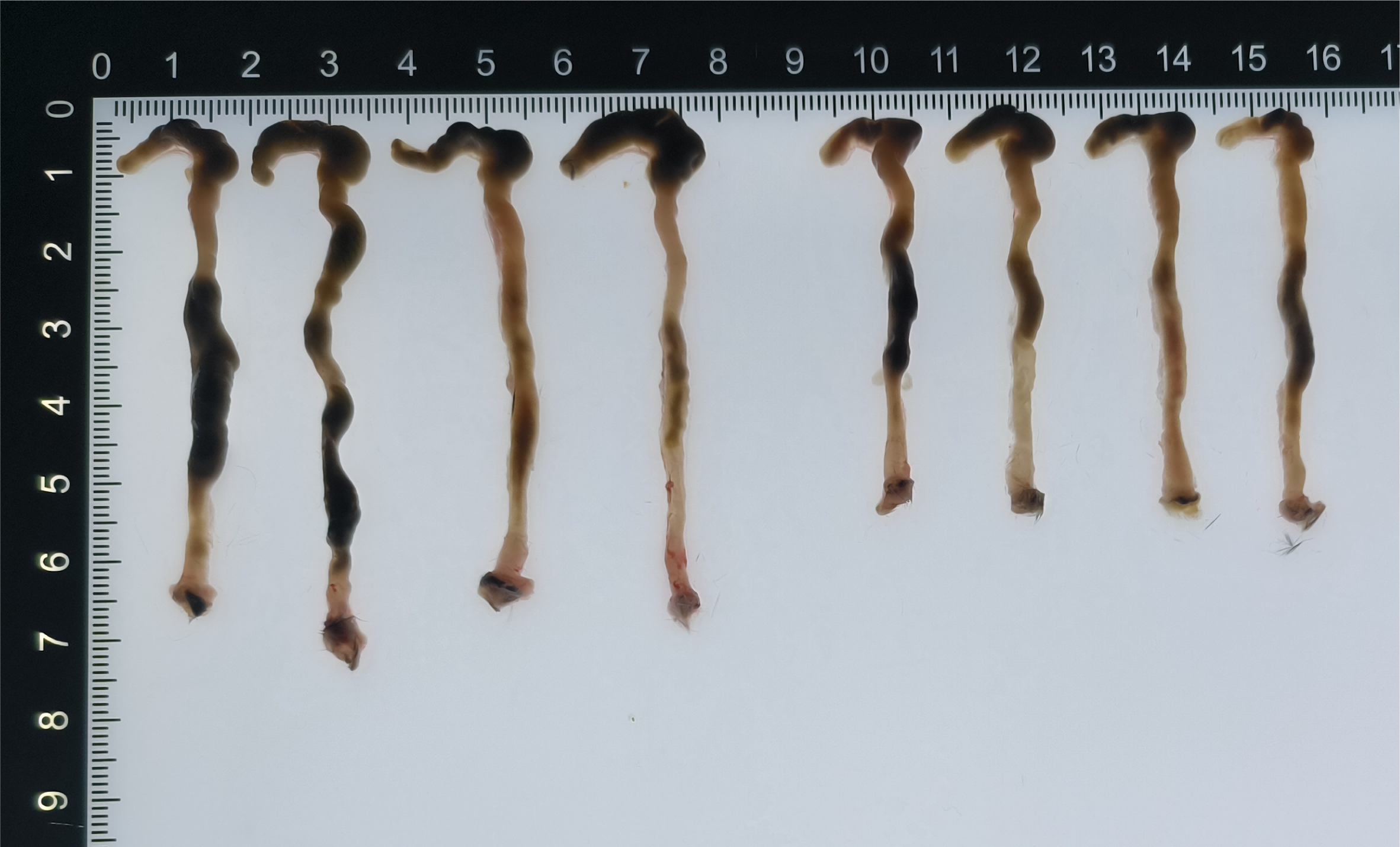

Supplement: Supplementary file 8 — Source data Fig. 3 [file 44319_2024_276_MOESM8_ESM.zip › Fig 3/3G/3G.png]

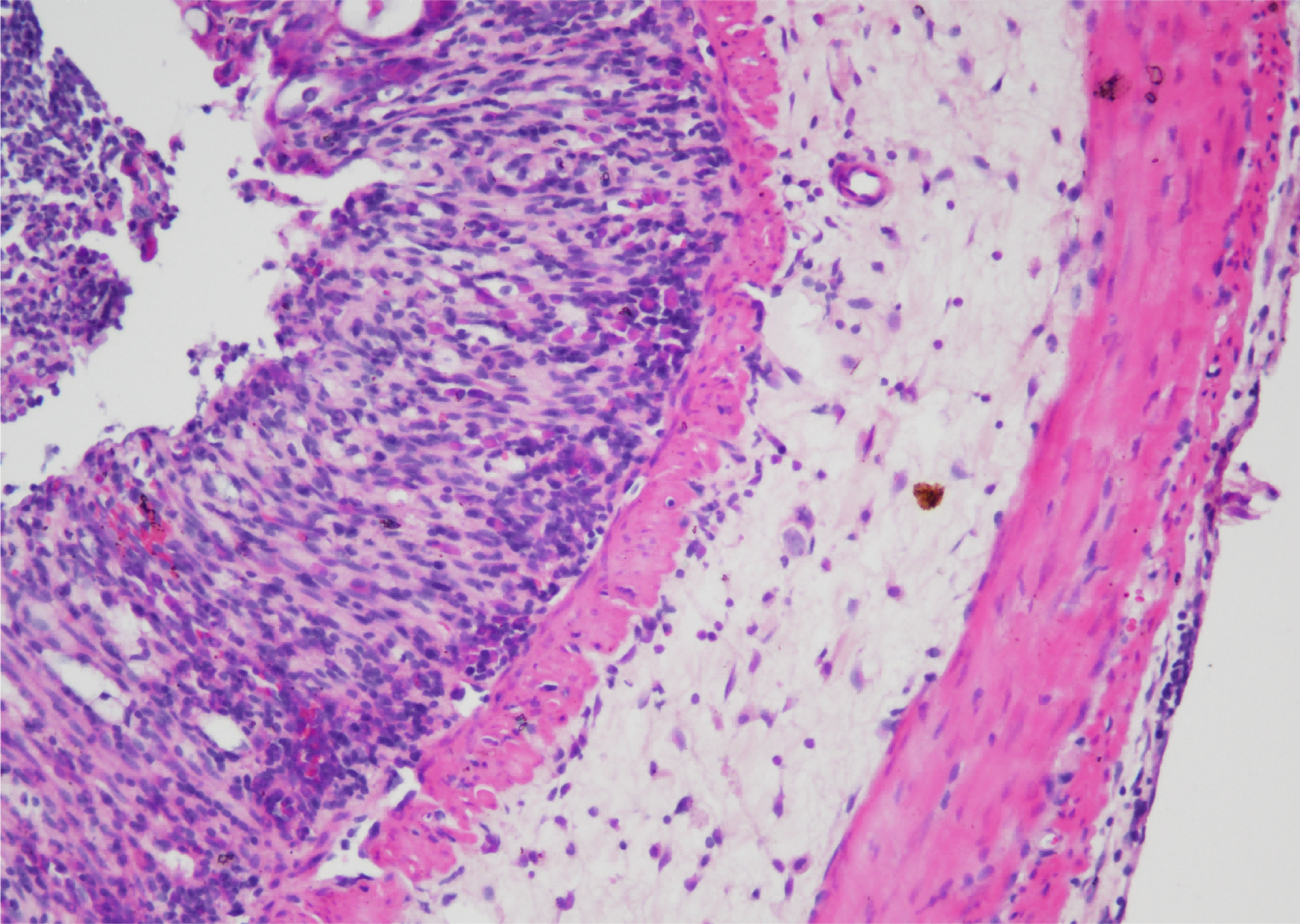

Supplement: Supplementary file 8 — Source data Fig. 3 [file 44319_2024_276_MOESM8_ESM.zip › Fig 3/3J/HE staining/KO to WT_200x.png]

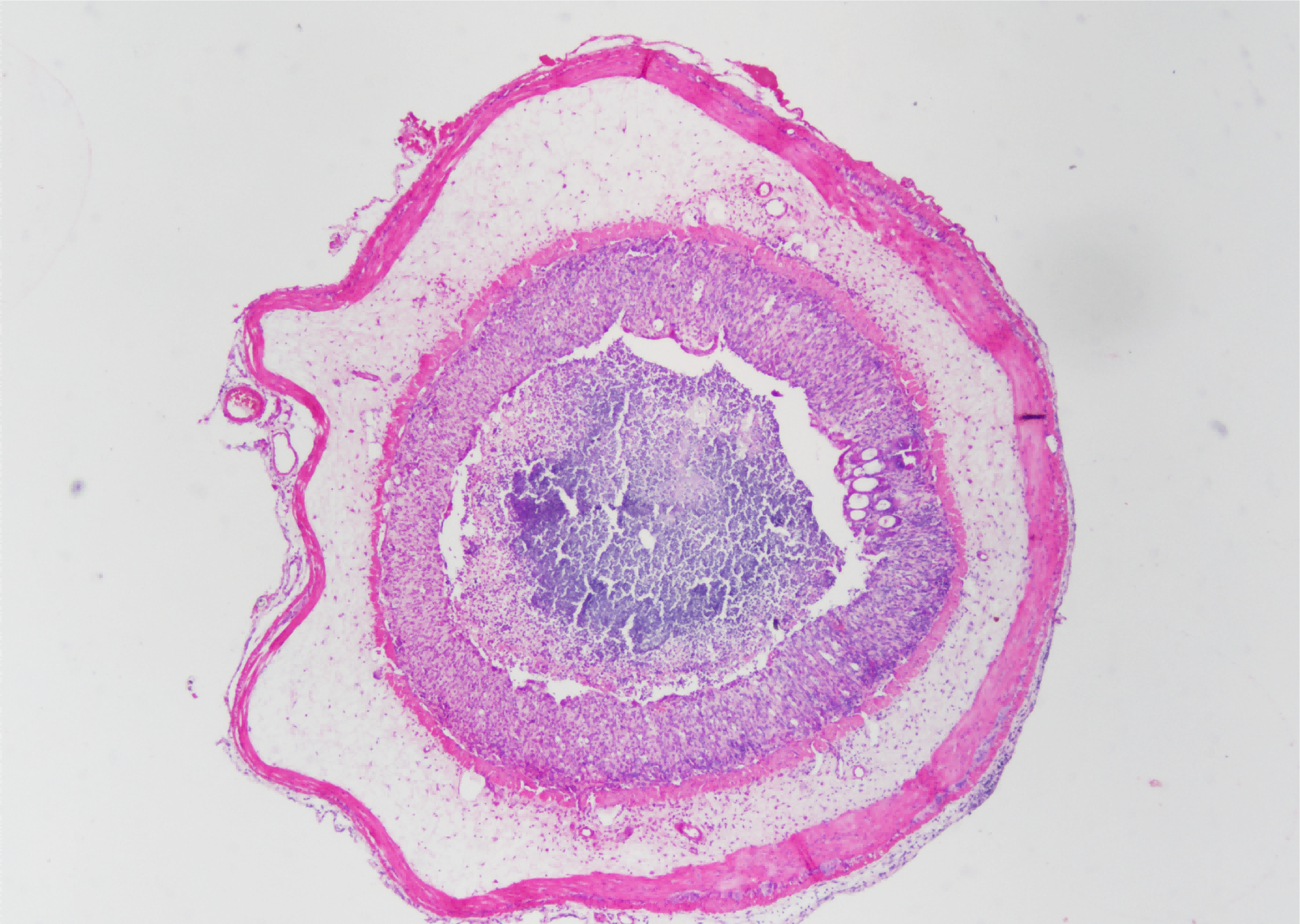

Supplement: Supplementary file 8 — Source data Fig. 3 [file 44319_2024_276_MOESM8_ESM.zip › Fig 3/3J/HE staining/KO to WT_40x.png]

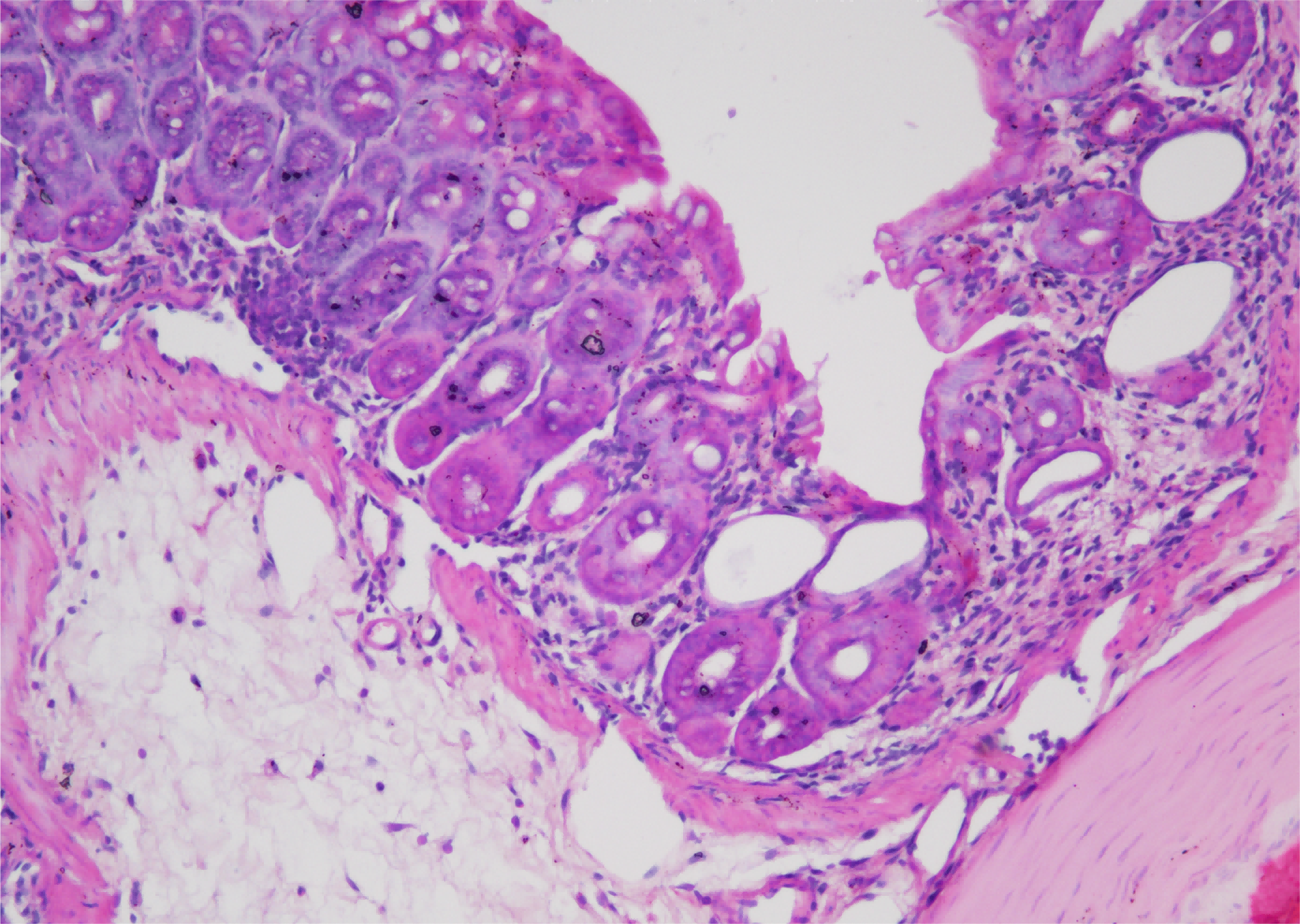

Supplement: Supplementary file 8 — Source data Fig. 3 [file 44319_2024_276_MOESM8_ESM.zip › Fig 3/3J/HE staining/WT to WT_200x.png]

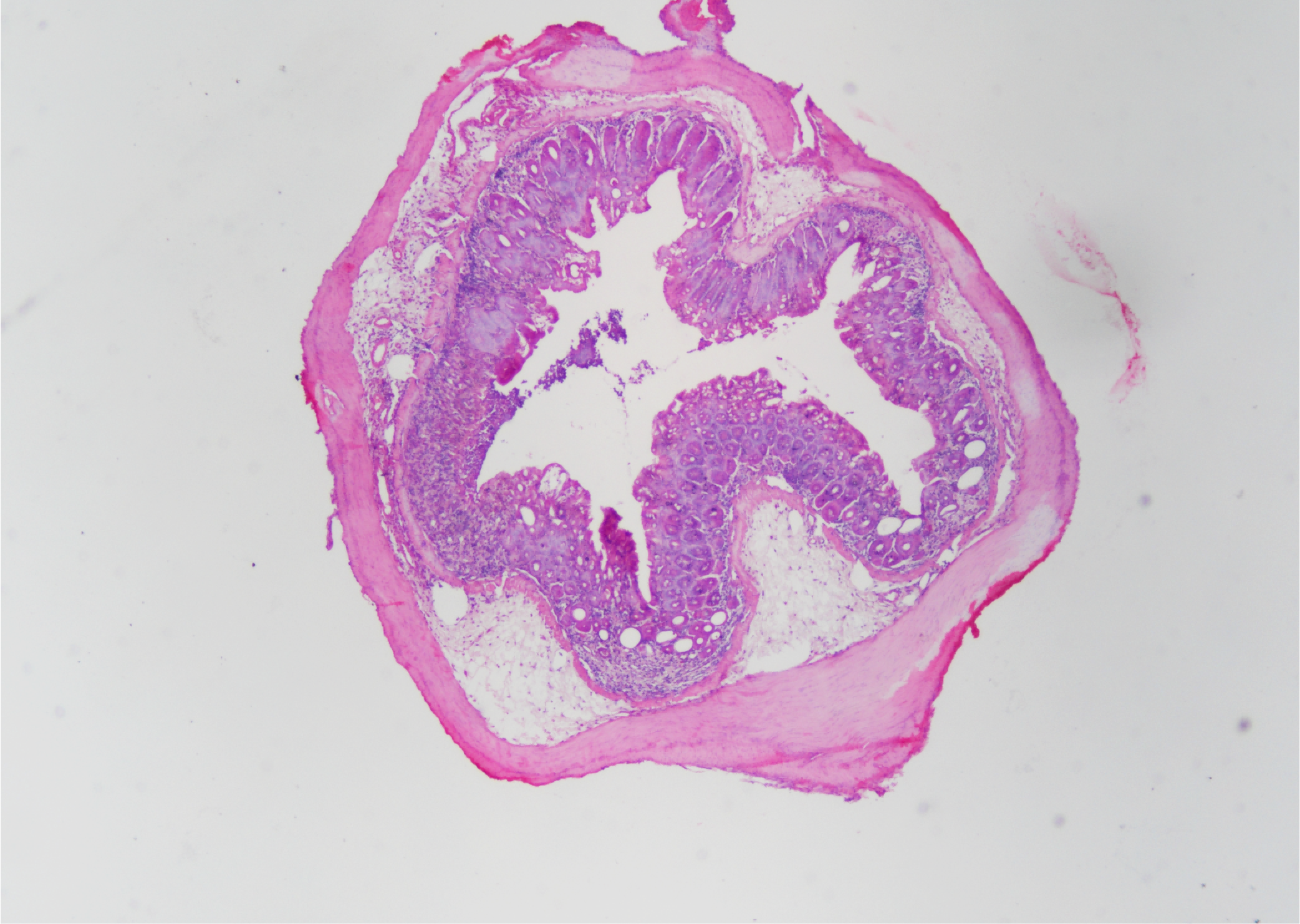

Supplement: Supplementary file 8 — Source data Fig. 3 [file 44319_2024_276_MOESM8_ESM.zip › Fig 3/3J/HE staining/WT to WT_40x.png]

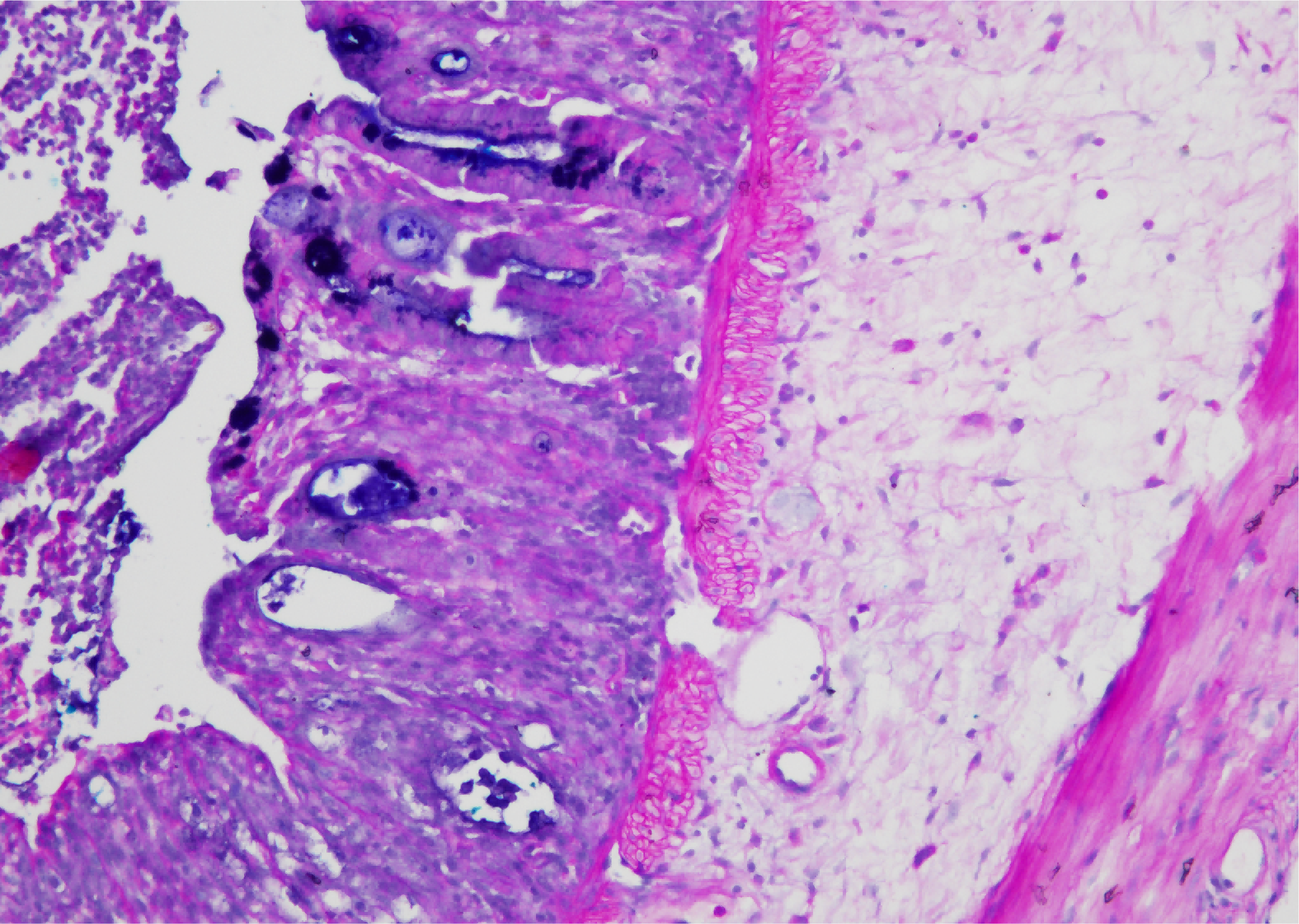

Supplement: Supplementary file 8 — Source data Fig. 3 [file 44319_2024_276_MOESM8_ESM.zip › Fig 3/3J/PAS_AB staining/KO to WT_200×.png]

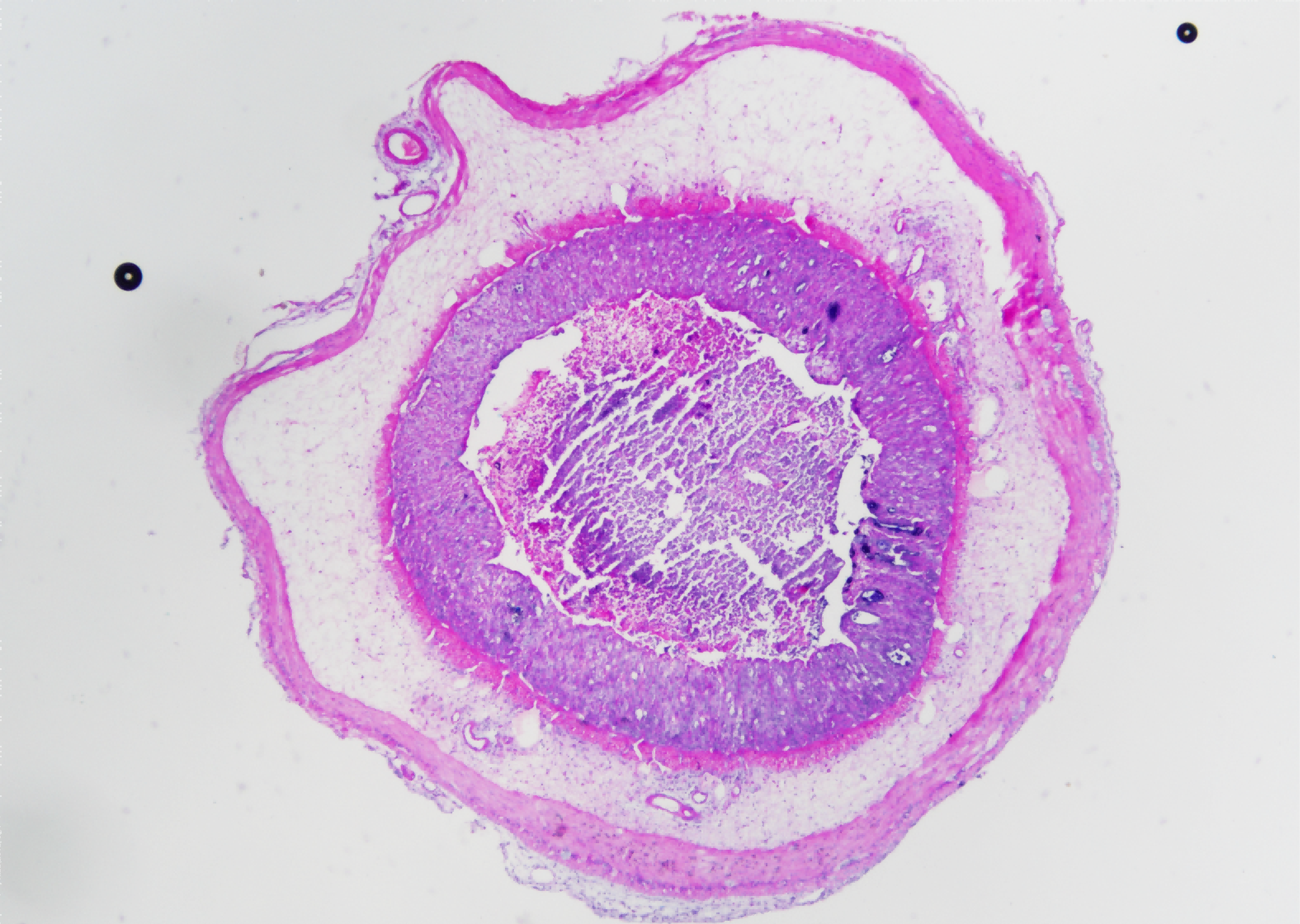

Supplement: Supplementary file 8 — Source data Fig. 3 [file 44319_2024_276_MOESM8_ESM.zip › Fig 3/3J/PAS_AB staining/KO to WT_40×.png]

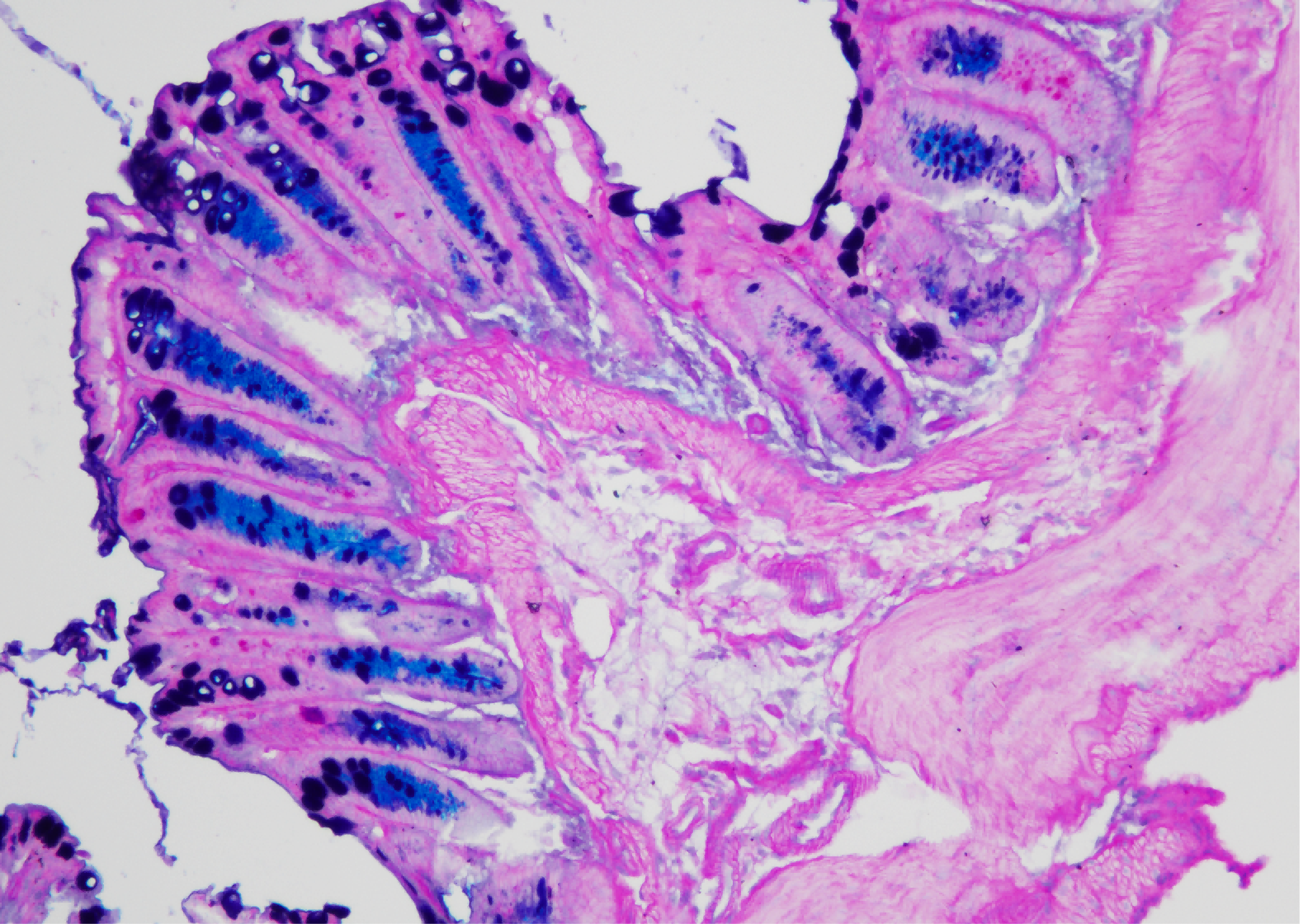

Supplement: Supplementary file 8 — Source data Fig. 3 [file 44319_2024_276_MOESM8_ESM.zip › Fig 3/3J/PAS_AB staining/WT to WT_200×.png]

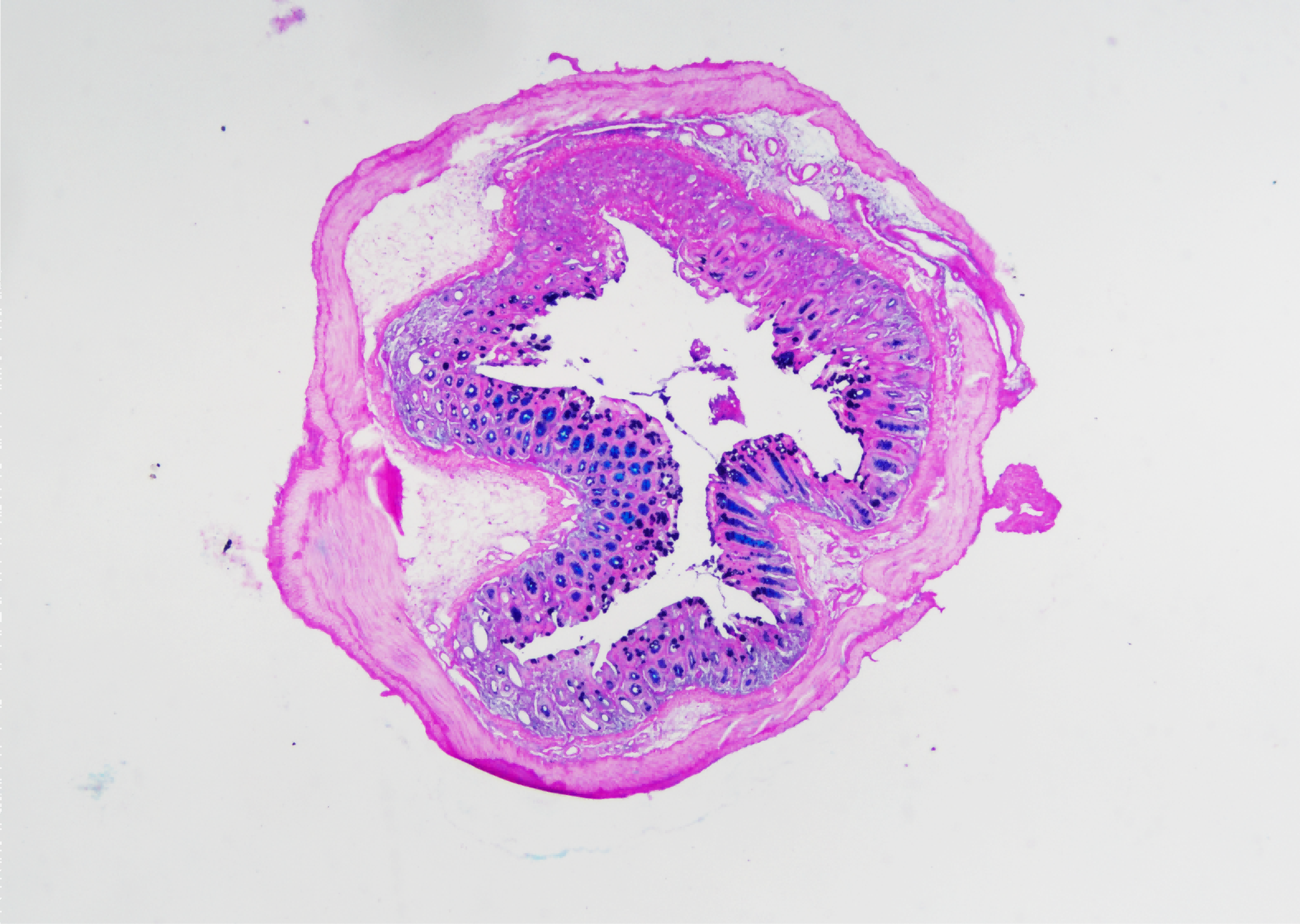

Supplement: Supplementary file 8 — Source data Fig. 3 [file 44319_2024_276_MOESM8_ESM.zip › Fig 3/3J/PAS_AB staining/WT to WT_40×.png]

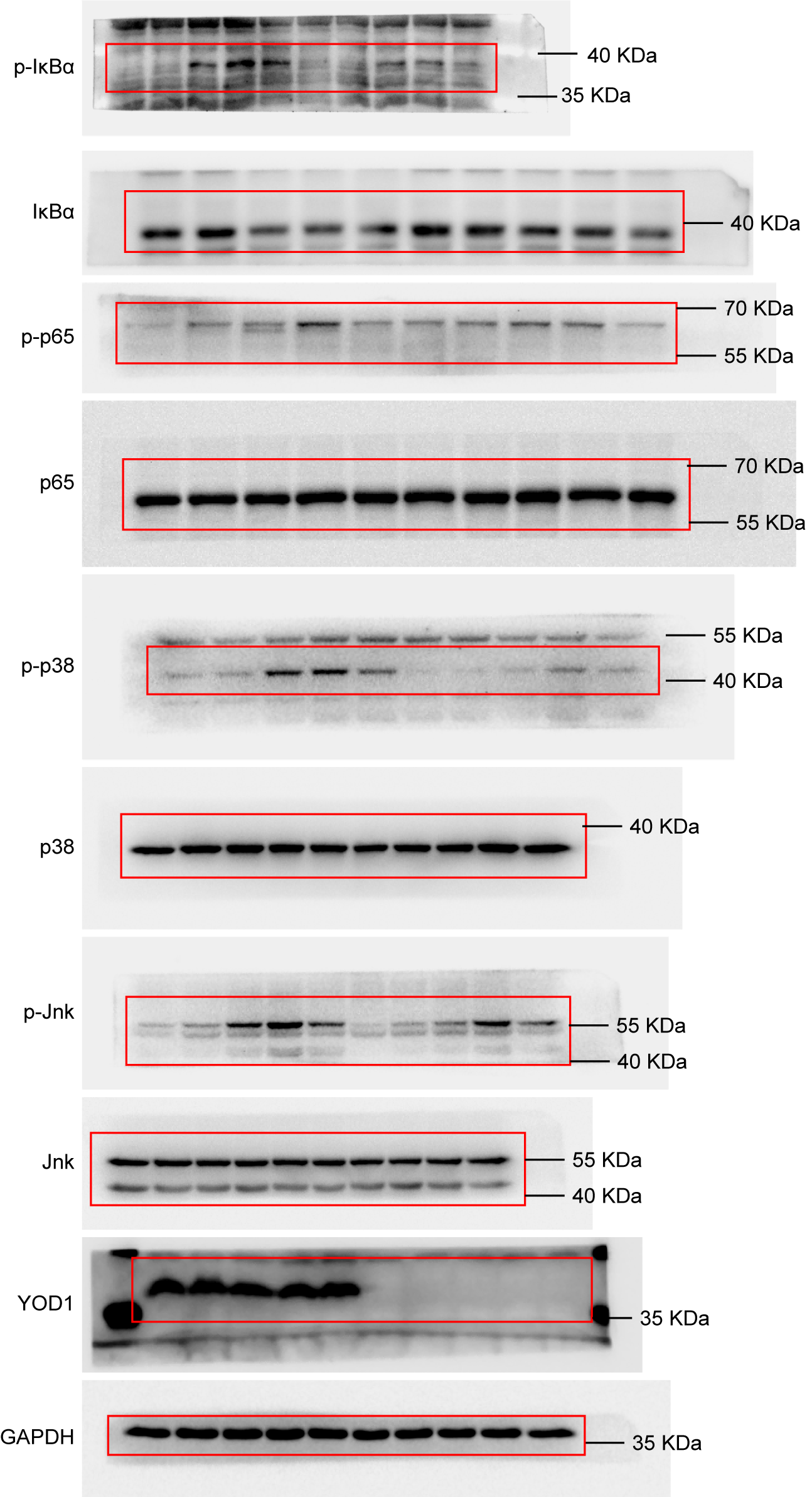

Supplement: Supplementary file 10 — Source data Fig. 5 [file 44319_2024_276_MOESM10_ESM.zip › Fig 5/5A/5A.png]

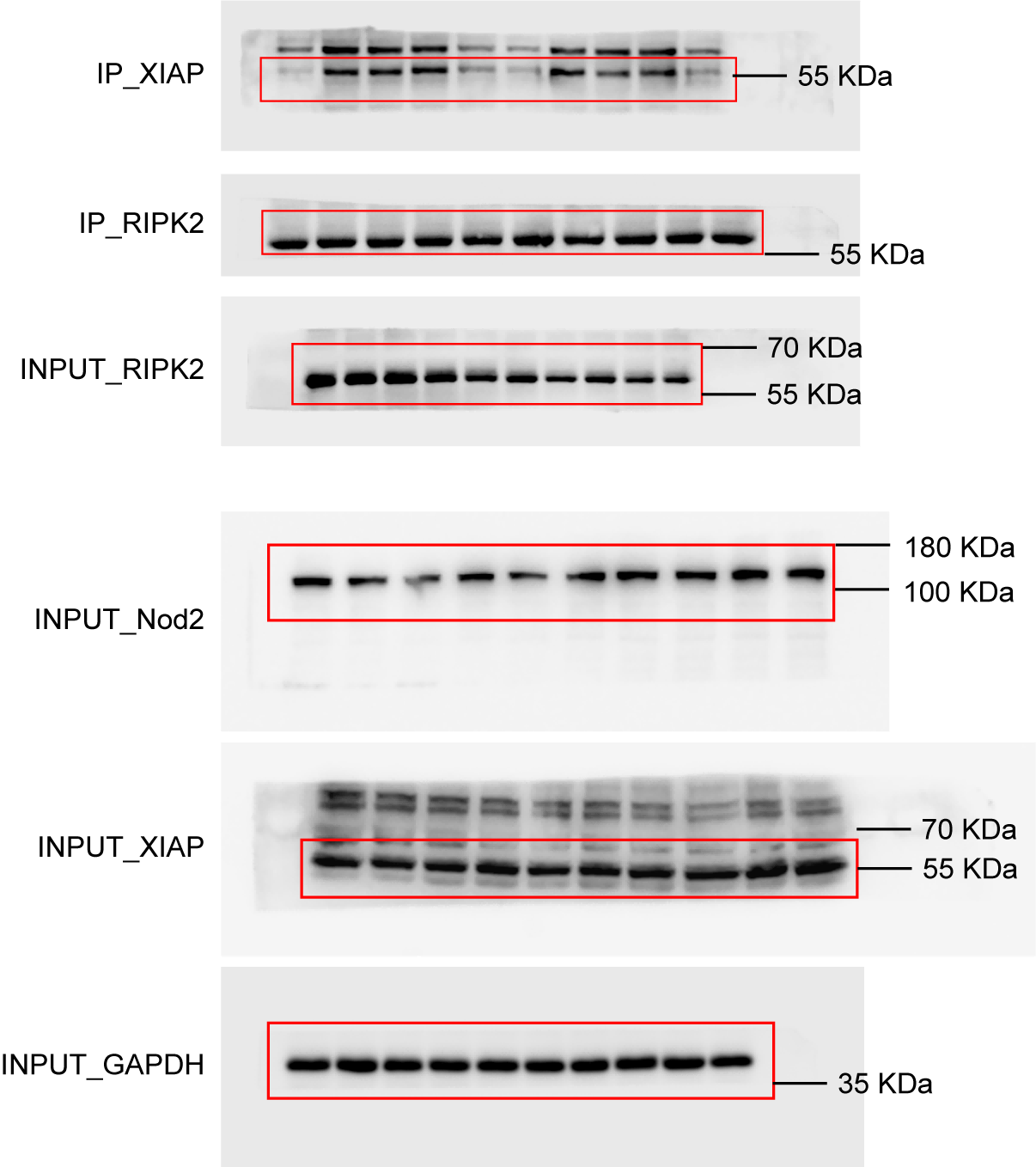

Supplement: Supplementary file 10 — Source data Fig. 5 [file 44319_2024_276_MOESM10_ESM.zip › Fig 5/5C/5C.png]

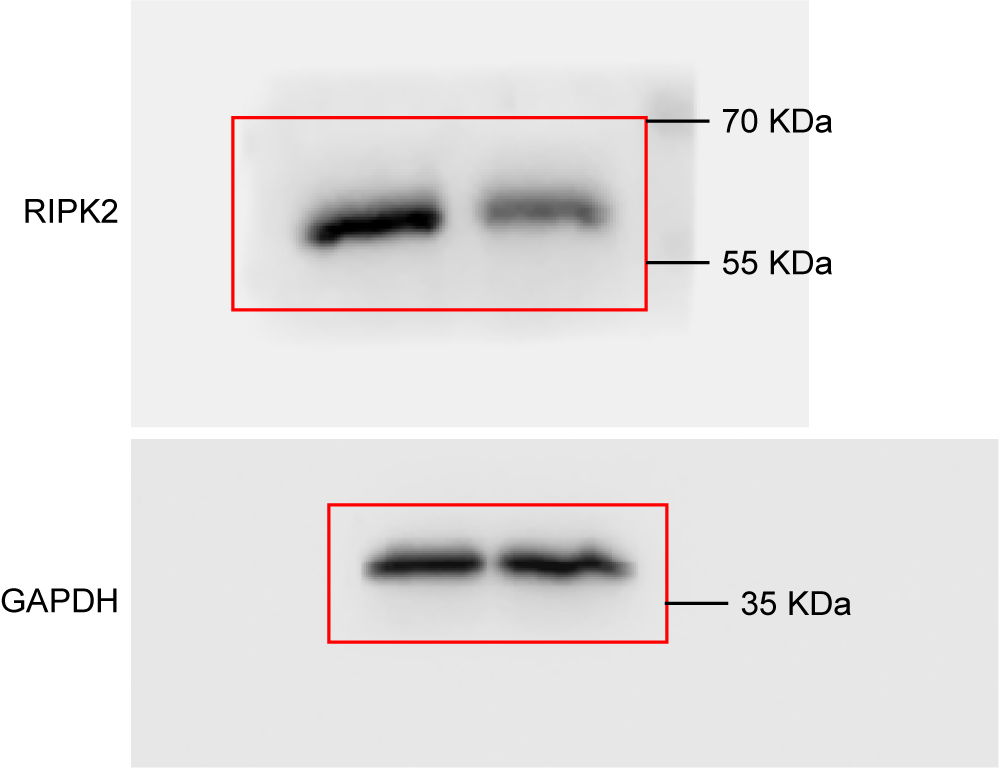

Supplement: Supplementary file 10 — Source data Fig. 5 [file 44319_2024_276_MOESM10_ESM.zip › Fig 5/5D/5D.tif]

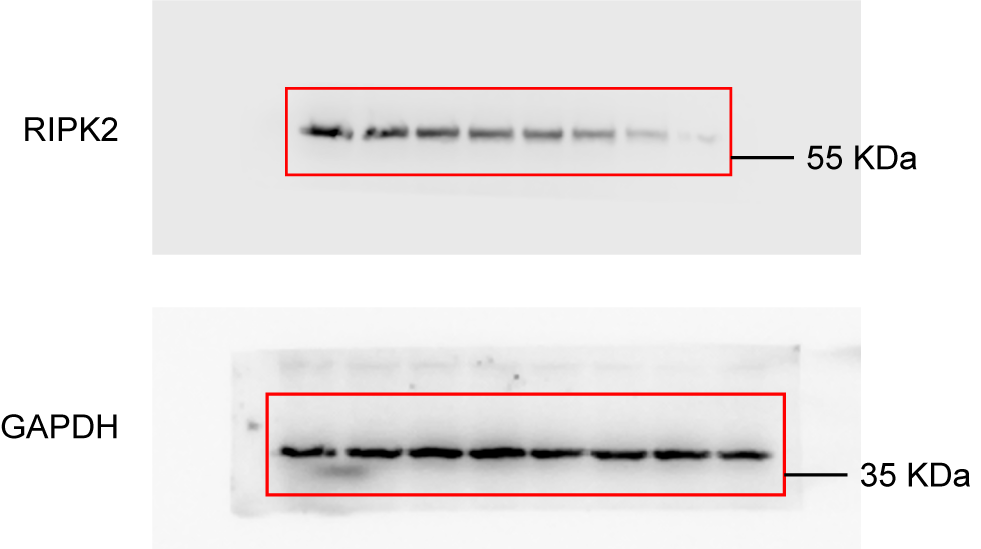

Supplement: Supplementary file 10 — Source data Fig. 5 [file 44319_2024_276_MOESM10_ESM.zip › Fig 5/5G/5G.tif]

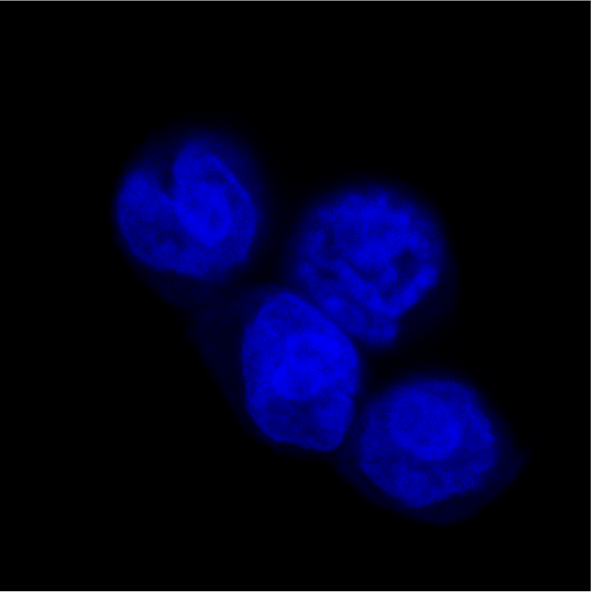

Supplement: Supplementary file 10 — Source data Fig. 5 [file 44319_2024_276_MOESM10_ESM.zip › Fig 5/5H/DAPI_FLAG-YOD1_0h.tif]

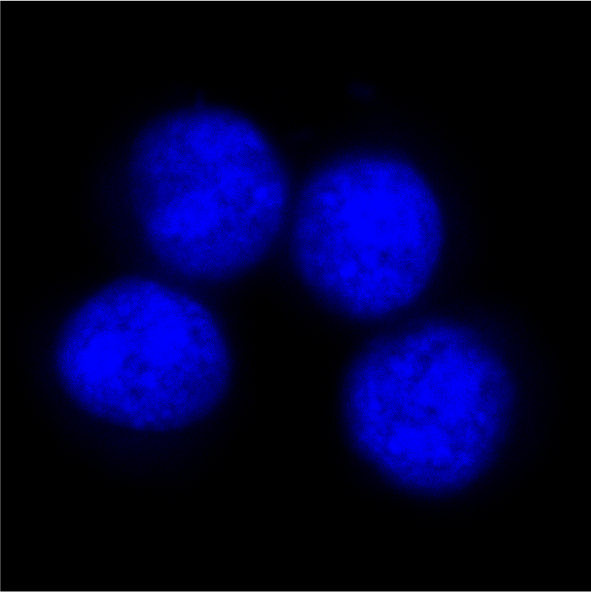

Supplement: Supplementary file 10 — Source data Fig. 5 [file 44319_2024_276_MOESM10_ESM.zip › Fig 5/5H/DAPI_FLAG-YOD1_3h.tif]

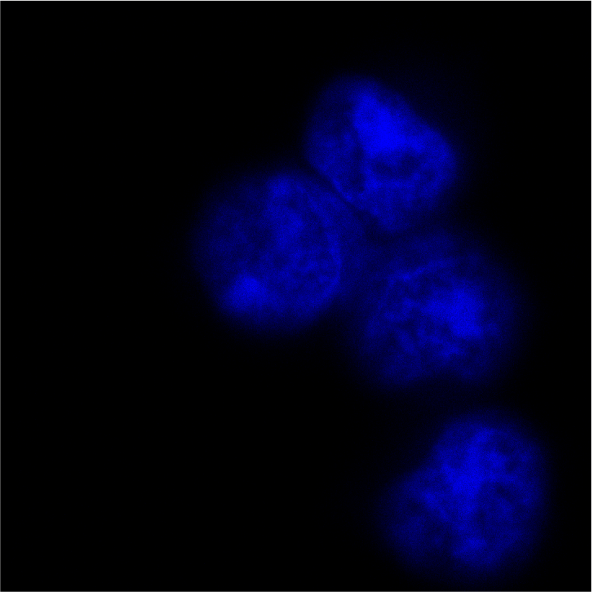

Supplement: Supplementary file 10 — Source data Fig. 5 [file 44319_2024_276_MOESM10_ESM.zip › Fig 5/5H/DAPI_FLAG-YOD1_6h.tif]

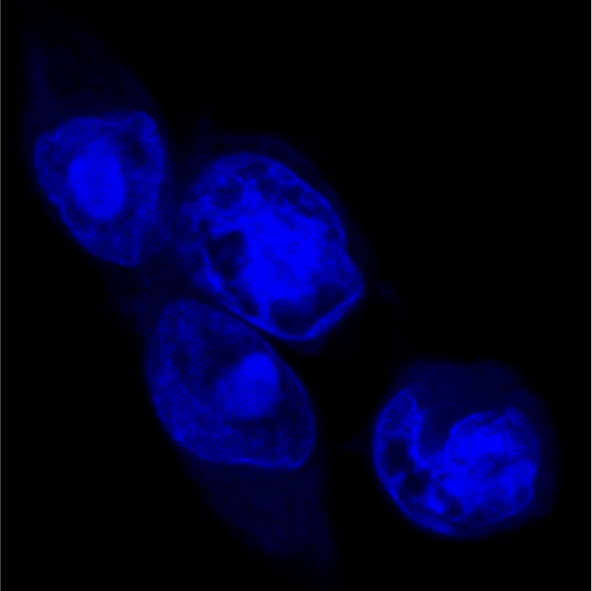

Supplement: Supplementary file 10 — Source data Fig. 5 [file 44319_2024_276_MOESM10_ESM.zip › Fig 5/5H/DAPI_FLAG-YOD1_9h.tif]

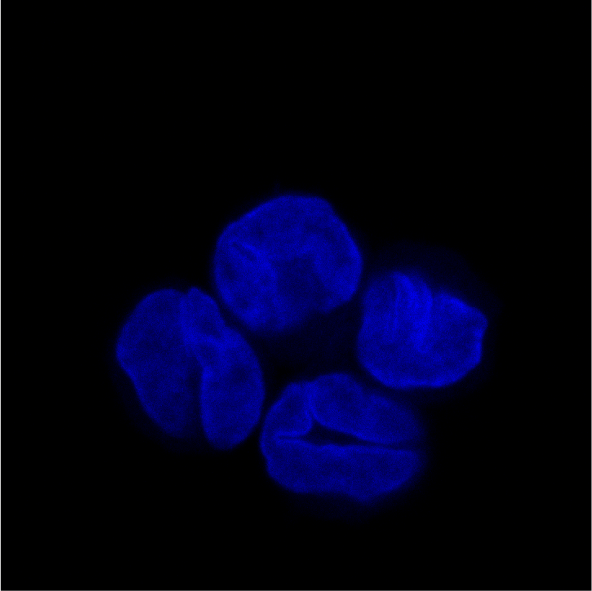

Supplement: Supplementary file 10 — Source data Fig. 5 [file 44319_2024_276_MOESM10_ESM.zip › Fig 5/5H/DAPI_FLAG_0h.tif]

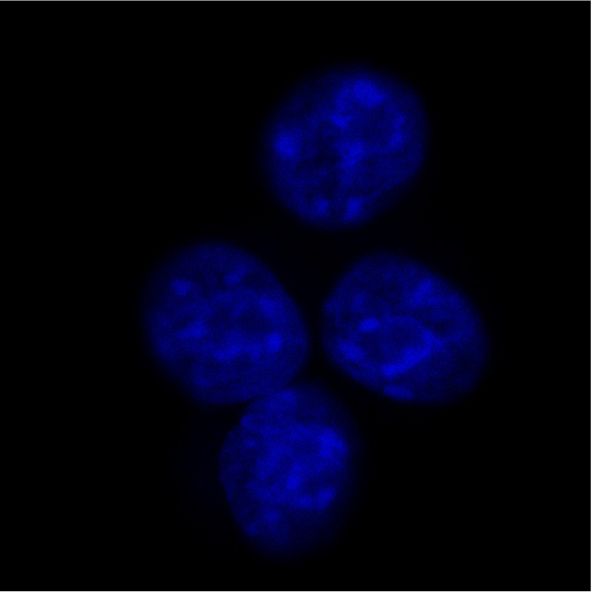

Supplement: Supplementary file 10 — Source data Fig. 5 [file 44319_2024_276_MOESM10_ESM.zip › Fig 5/5H/DAPI_FLAG_3h.tif]

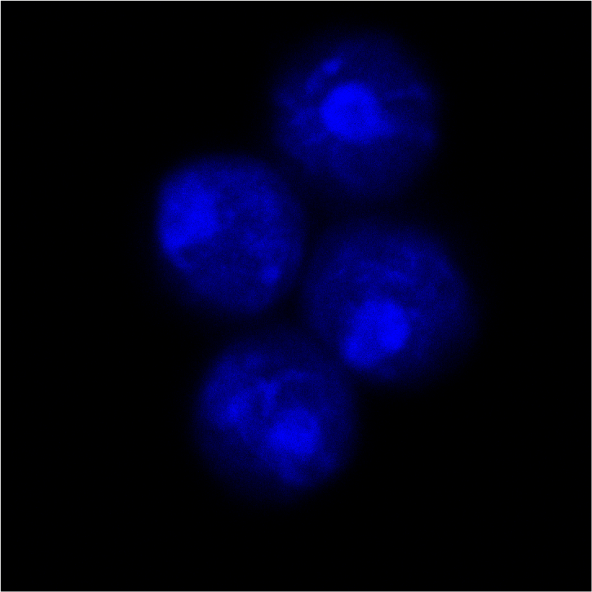

Supplement: Supplementary file 10 — Source data Fig. 5 [file 44319_2024_276_MOESM10_ESM.zip › Fig 5/5H/DAPI_FLAG_6h.tif]

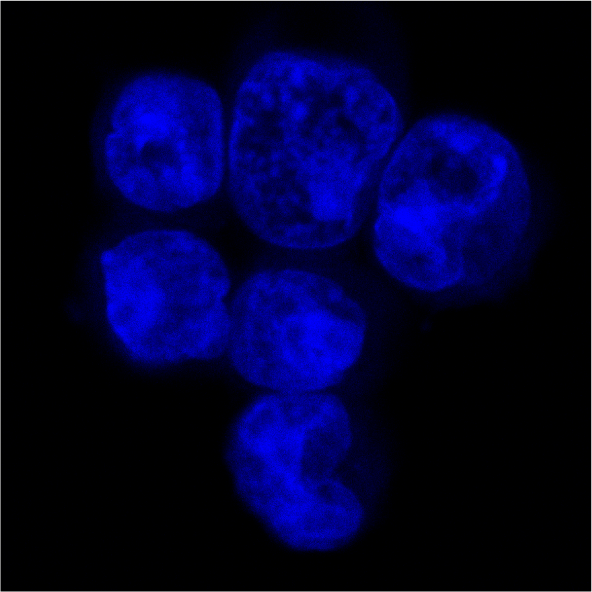

Supplement: Supplementary file 10 — Source data Fig. 5 [file 44319_2024_276_MOESM10_ESM.zip › Fig 5/5H/DAPI_FLAG_9h.tif]

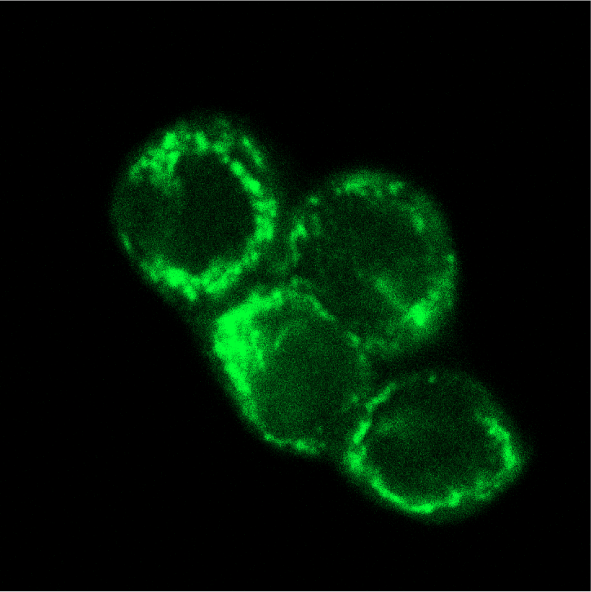

Supplement: Supplementary file 10 — Source data Fig. 5 [file 44319_2024_276_MOESM10_ESM.zip › Fig 5/5H/RIPK2_FLAG-YOD1_0h.tif]

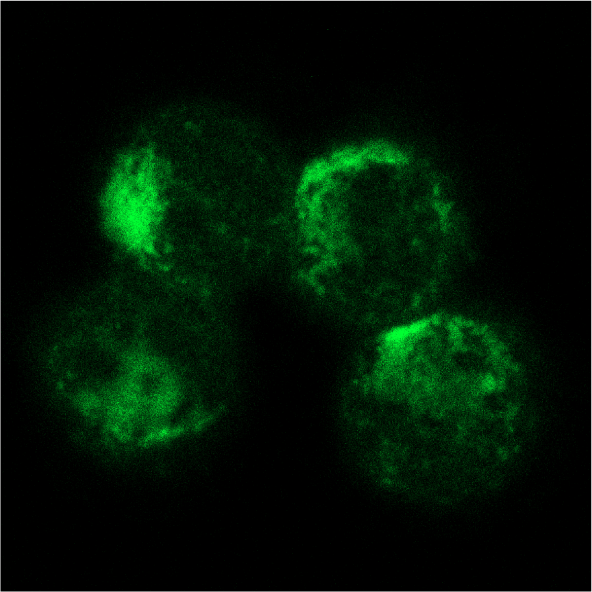

Supplement: Supplementary file 10 — Source data Fig. 5 [file 44319_2024_276_MOESM10_ESM.zip › Fig 5/5H/RIPK2_FLAG-YOD1_3h.tif]

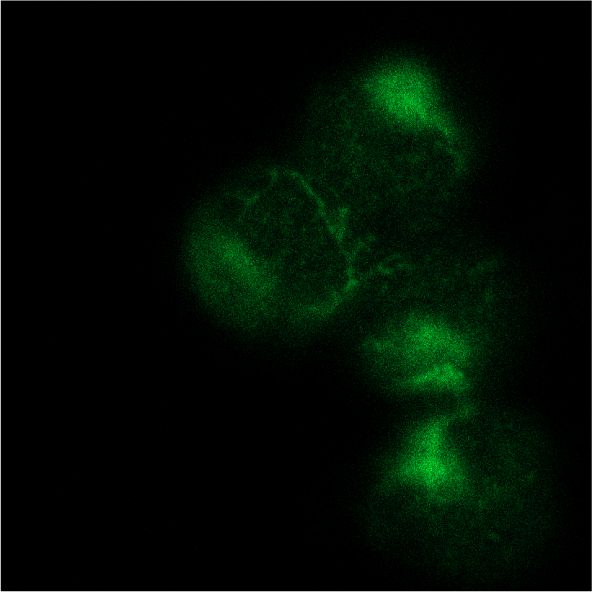

Supplement: Supplementary file 10 — Source data Fig. 5 [file 44319_2024_276_MOESM10_ESM.zip › Fig 5/5H/RIPK2_FLAG-YOD1_6h.tif]

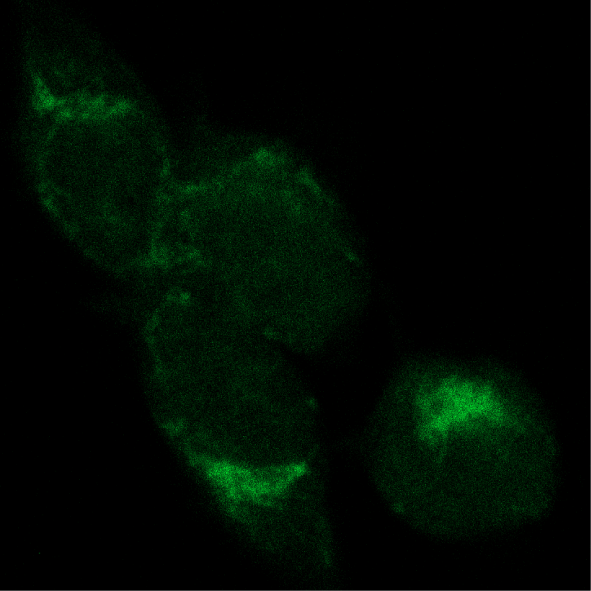

Supplement: Supplementary file 10 — Source data Fig. 5 [file 44319_2024_276_MOESM10_ESM.zip › Fig 5/5H/RIPK2_FLAG-YOD1_9h.tif]

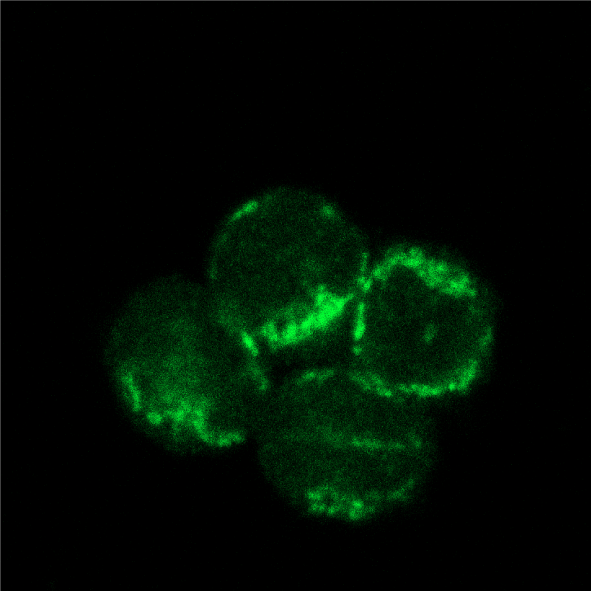

Supplement: Supplementary file 10 — Source data Fig. 5 [file 44319_2024_276_MOESM10_ESM.zip › Fig 5/5H/RIPK2_FLAG_0h.tif]

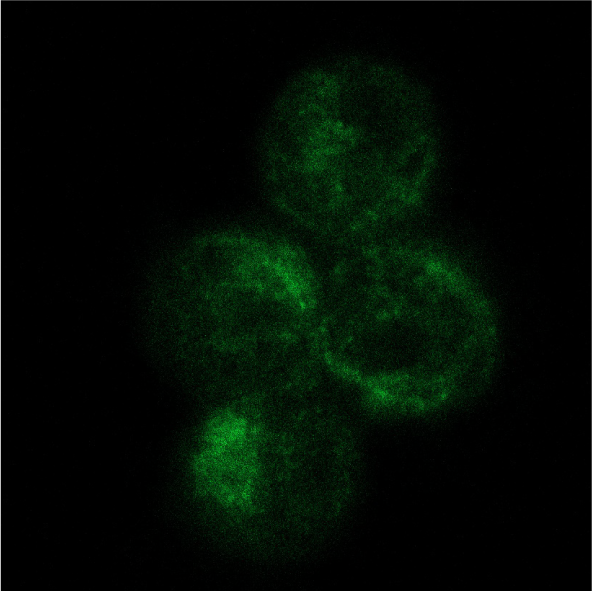

Supplement: Supplementary file 10 — Source data Fig. 5 [file 44319_2024_276_MOESM10_ESM.zip › Fig 5/5H/RIPK2_FLAG_3h.tif]

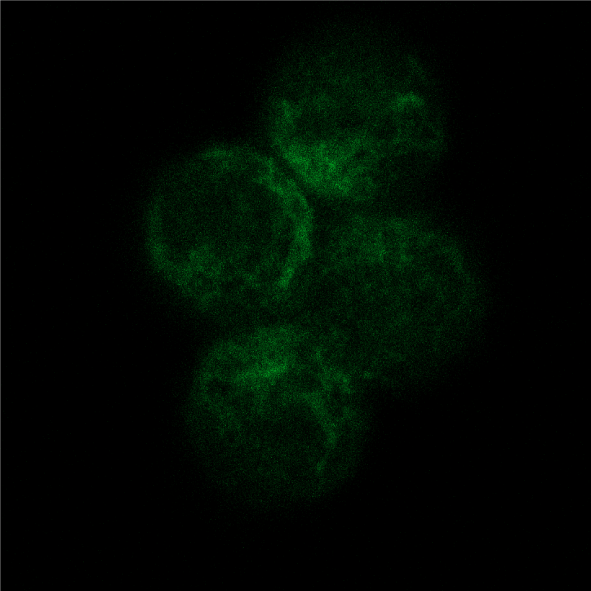

Supplement: Supplementary file 10 — Source data Fig. 5 [file 44319_2024_276_MOESM10_ESM.zip › Fig 5/5H/RIPK2_FLAG_6h.tif]

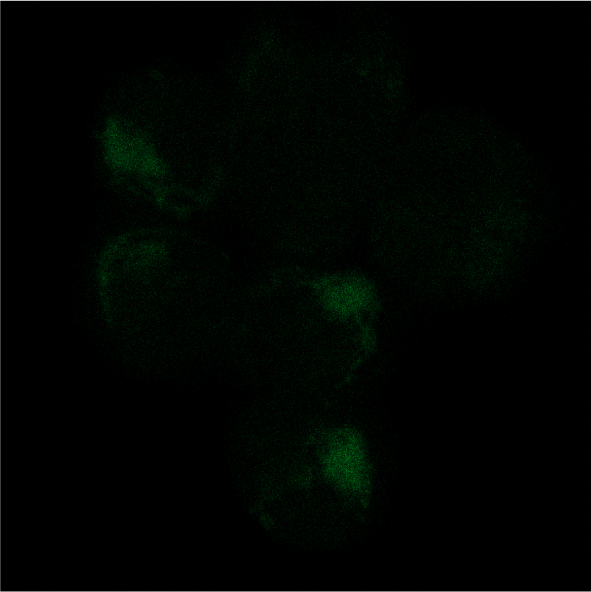

Supplement: Supplementary file 10 — Source data Fig. 5 [file 44319_2024_276_MOESM10_ESM.zip › Fig 5/5H/RIPK2_FLAG_9h.tif]

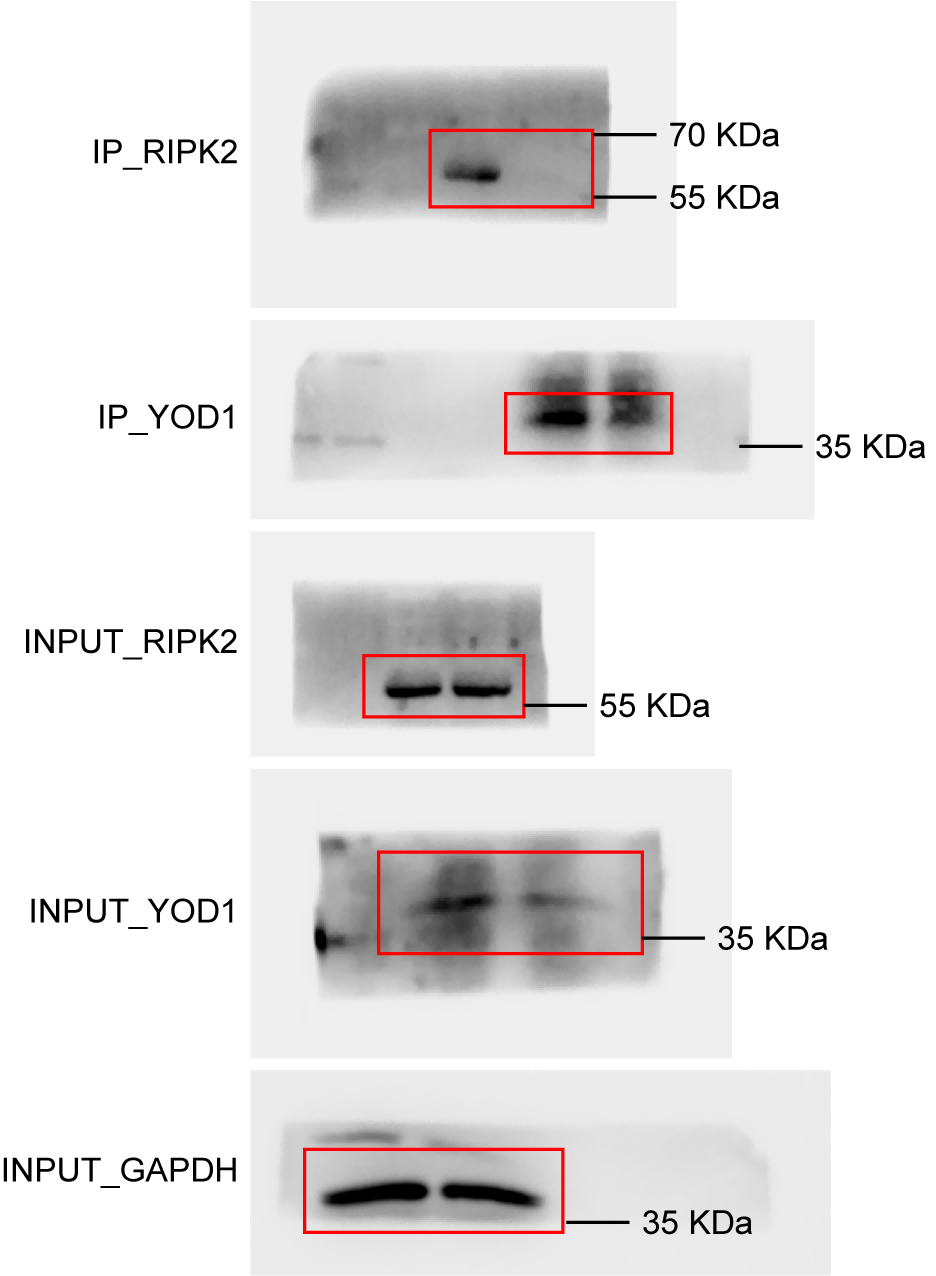

Supplement: Supplementary file 11 — Source data Fig. 6 [file 44319_2024_276_MOESM11_ESM.zip › Fig 6/6A/6A.png]

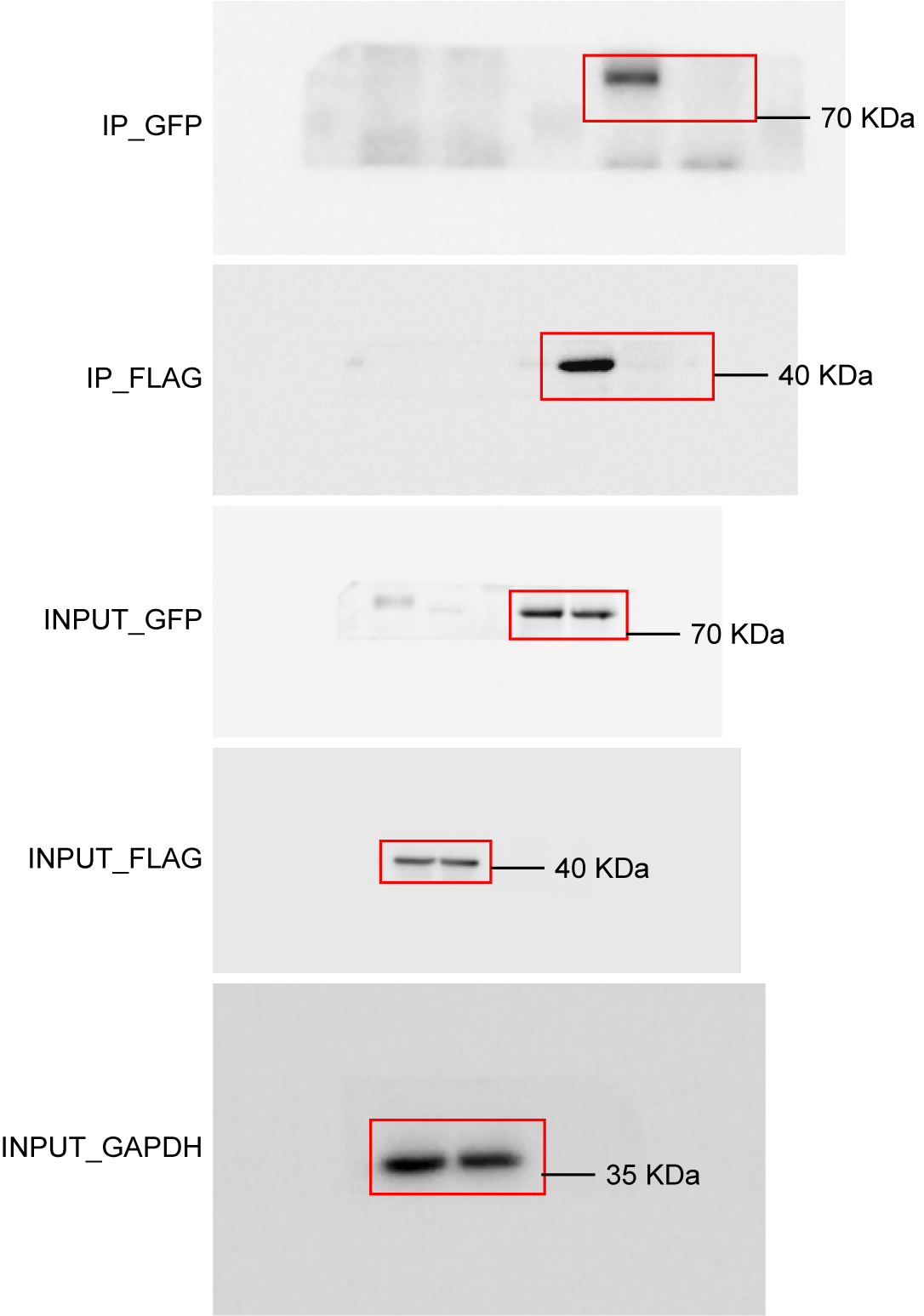

Supplement: Supplementary file 11 — Source data Fig. 6 [file 44319_2024_276_MOESM11_ESM.zip › Fig 6/6B/6B.png]

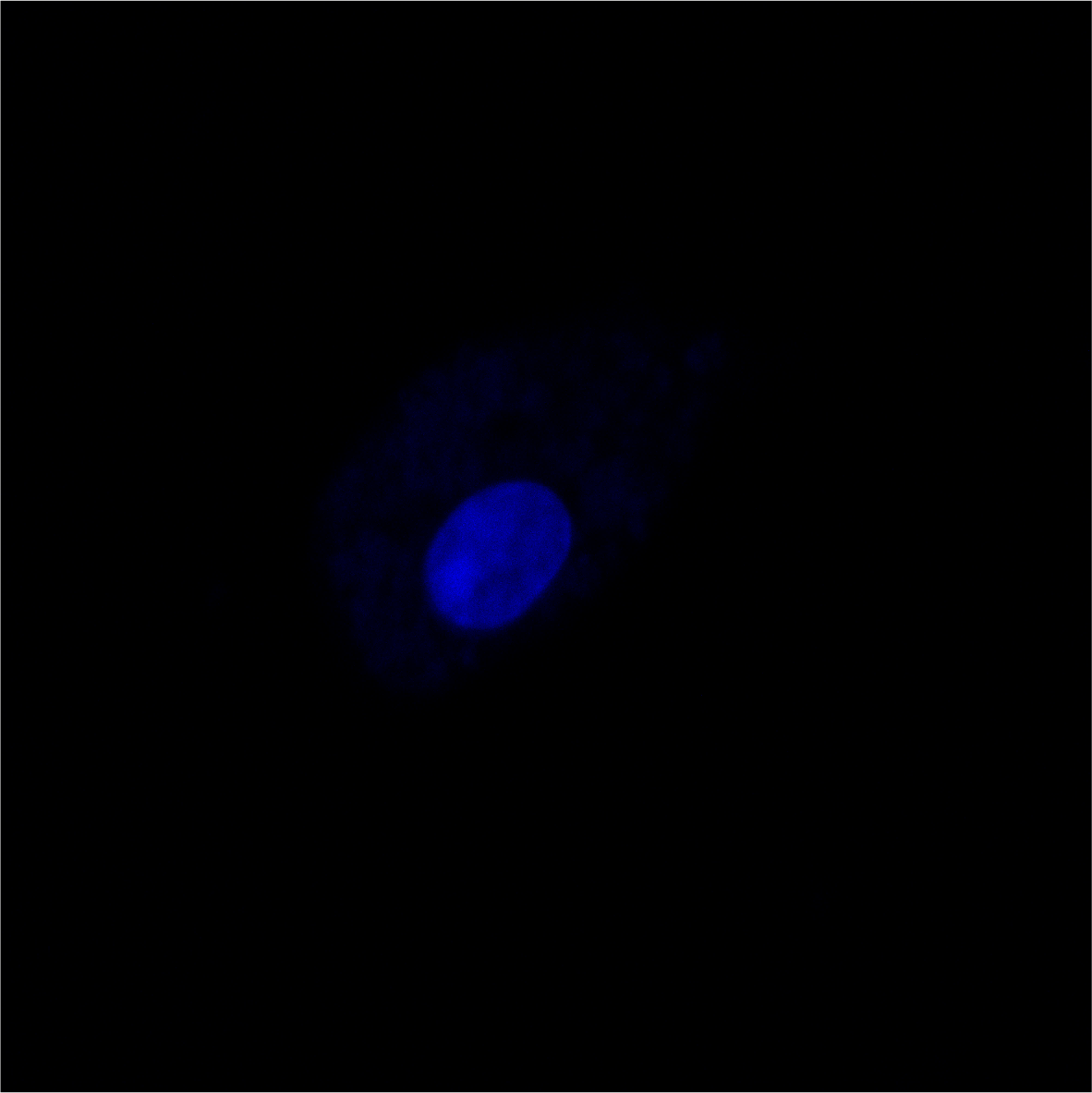

Supplement: Supplementary file 11 — Source data Fig. 6 [file 44319_2024_276_MOESM11_ESM.zip › Fig 6/6C/DAPI.png]

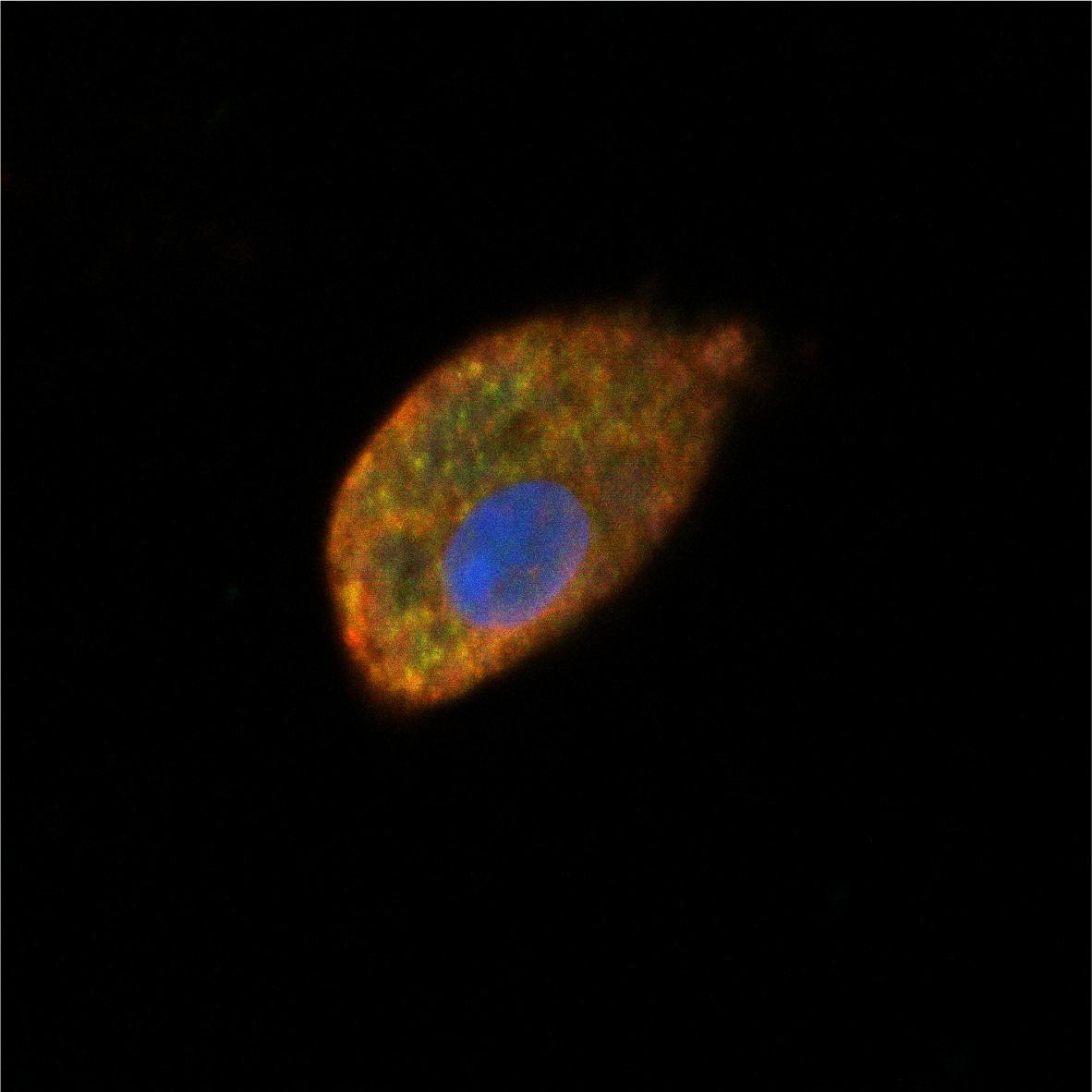

Supplement: Supplementary file 11 — Source data Fig. 6 [file 44319_2024_276_MOESM11_ESM.zip › Fig 6/6C/Merge.png]

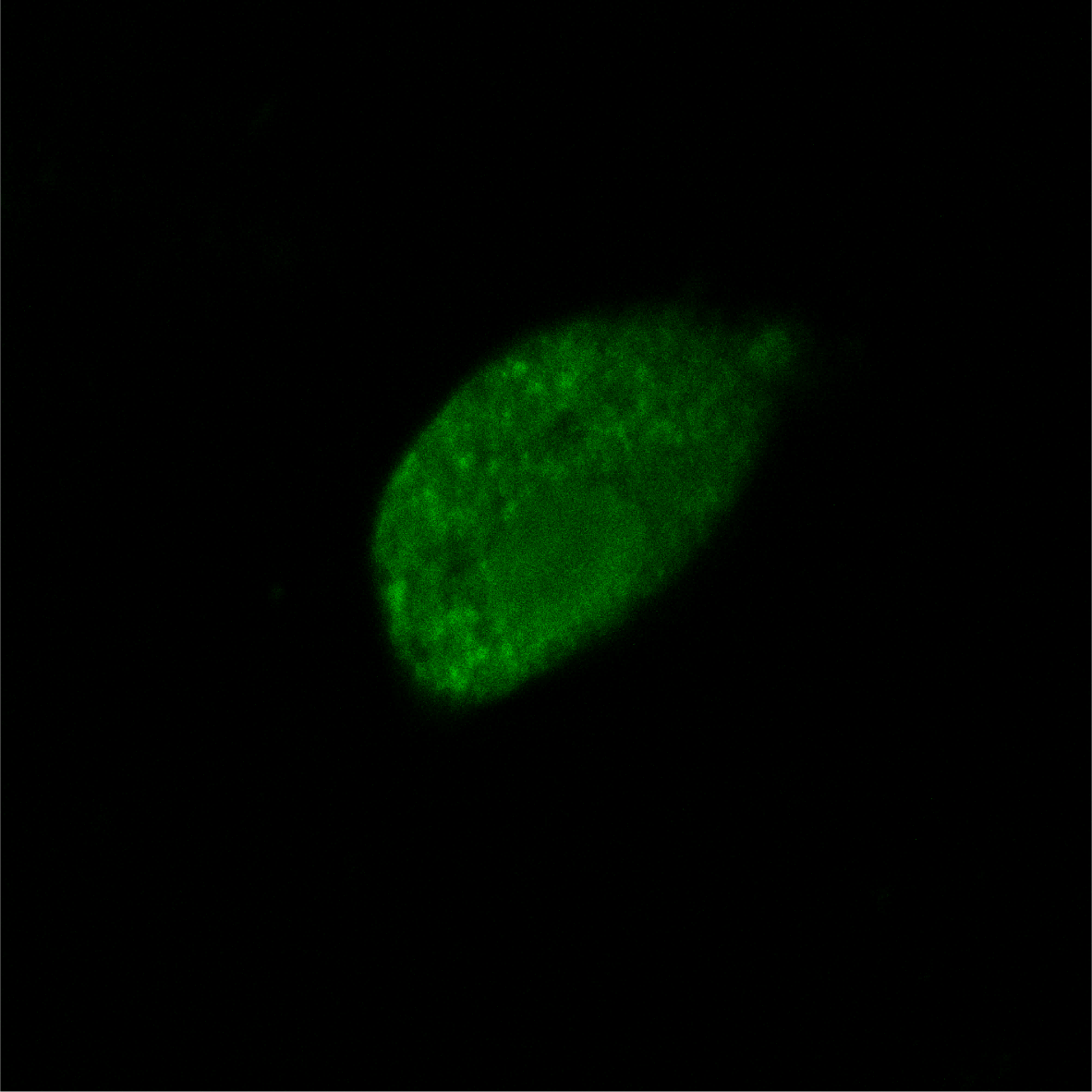

Supplement: Supplementary file 11 — Source data Fig. 6 [file 44319_2024_276_MOESM11_ESM.zip › Fig 6/6C/RIPK2.png]

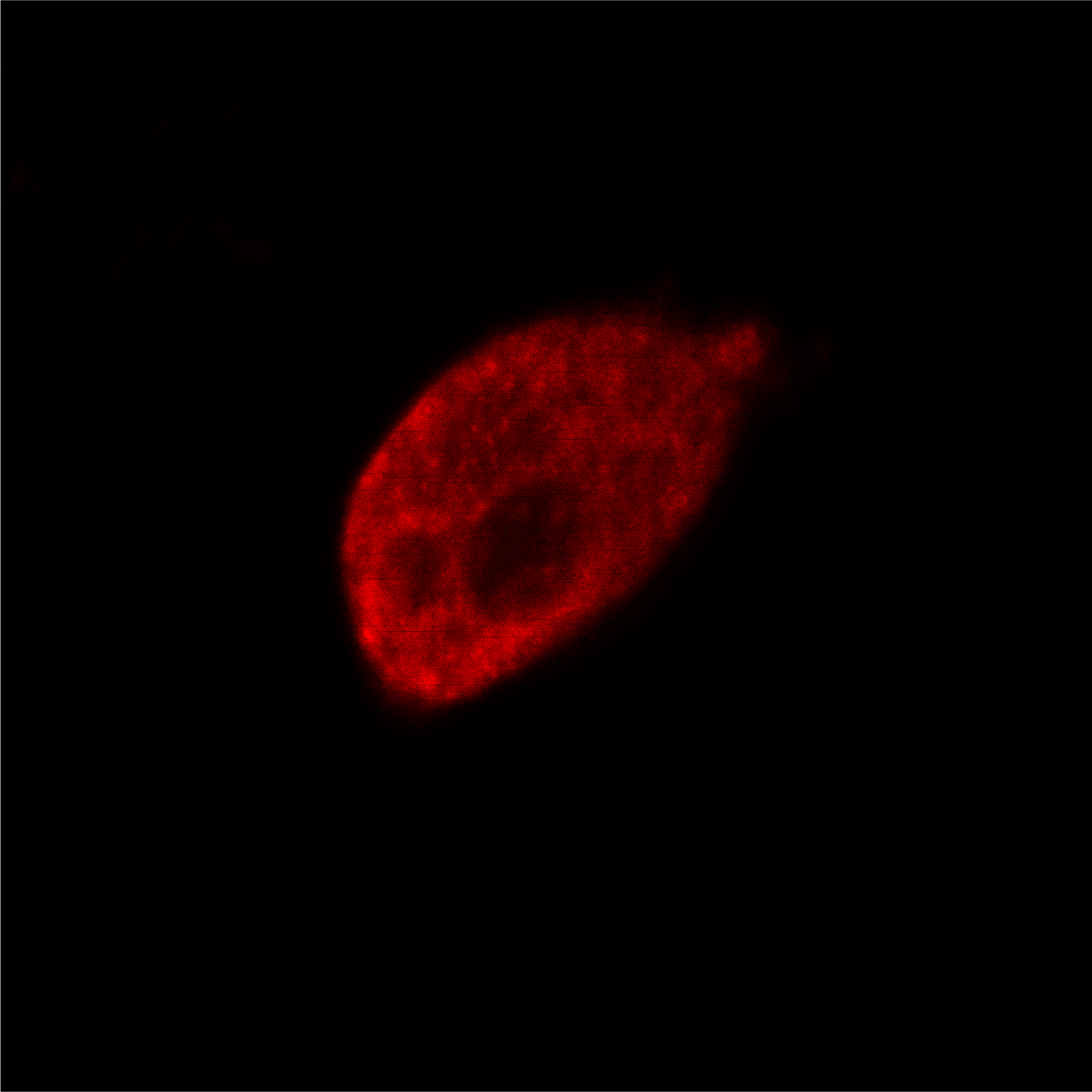

Supplement: Supplementary file 11 — Source data Fig. 6 [file 44319_2024_276_MOESM11_ESM.zip › Fig 6/6C/YOD1.png]

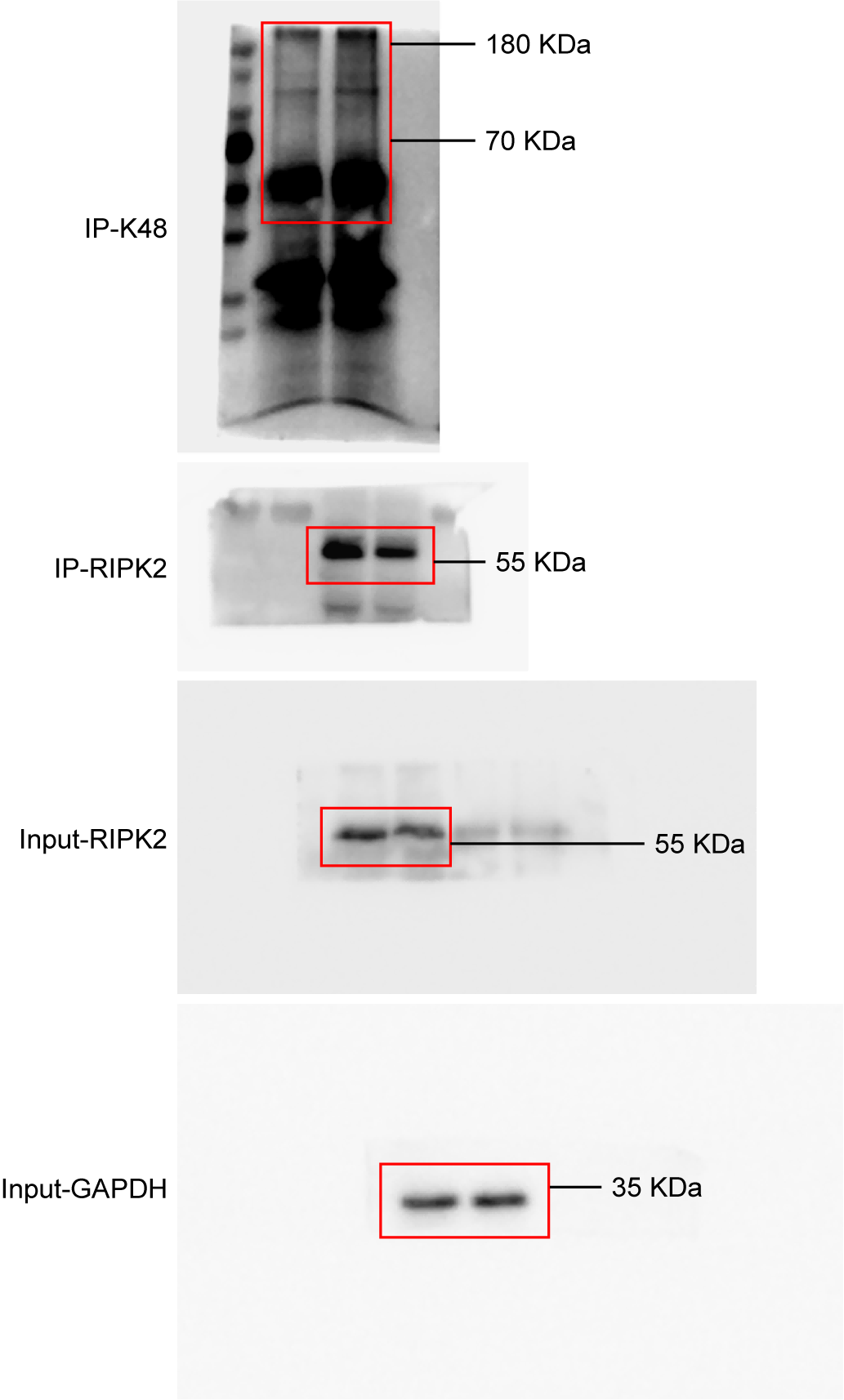

Supplement: Supplementary file 11 — Source data Fig. 6 [file 44319_2024_276_MOESM11_ESM.zip › Fig 6/6D/6D.png]

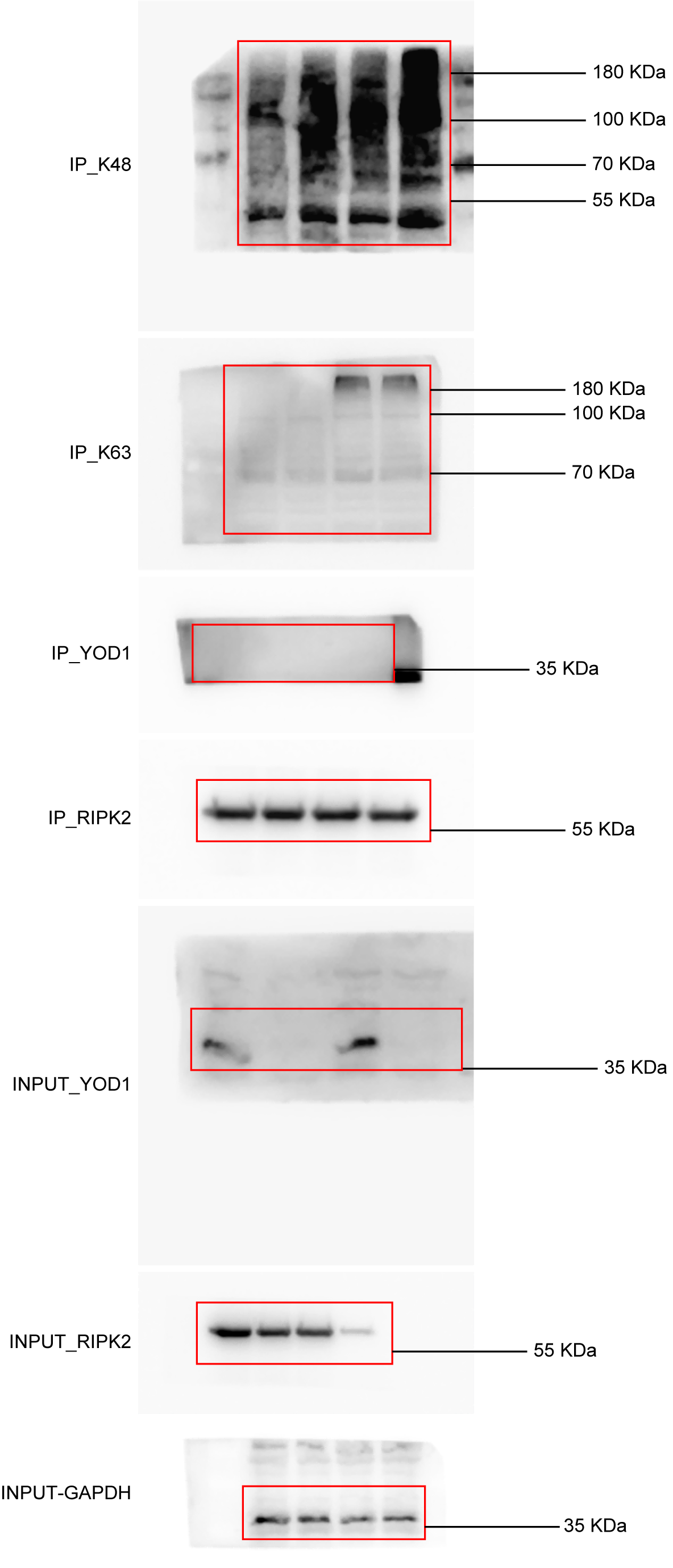

Supplement: Supplementary file 11 — Source data Fig. 6 [file 44319_2024_276_MOESM11_ESM.zip › Fig 6/6E/6E.png]

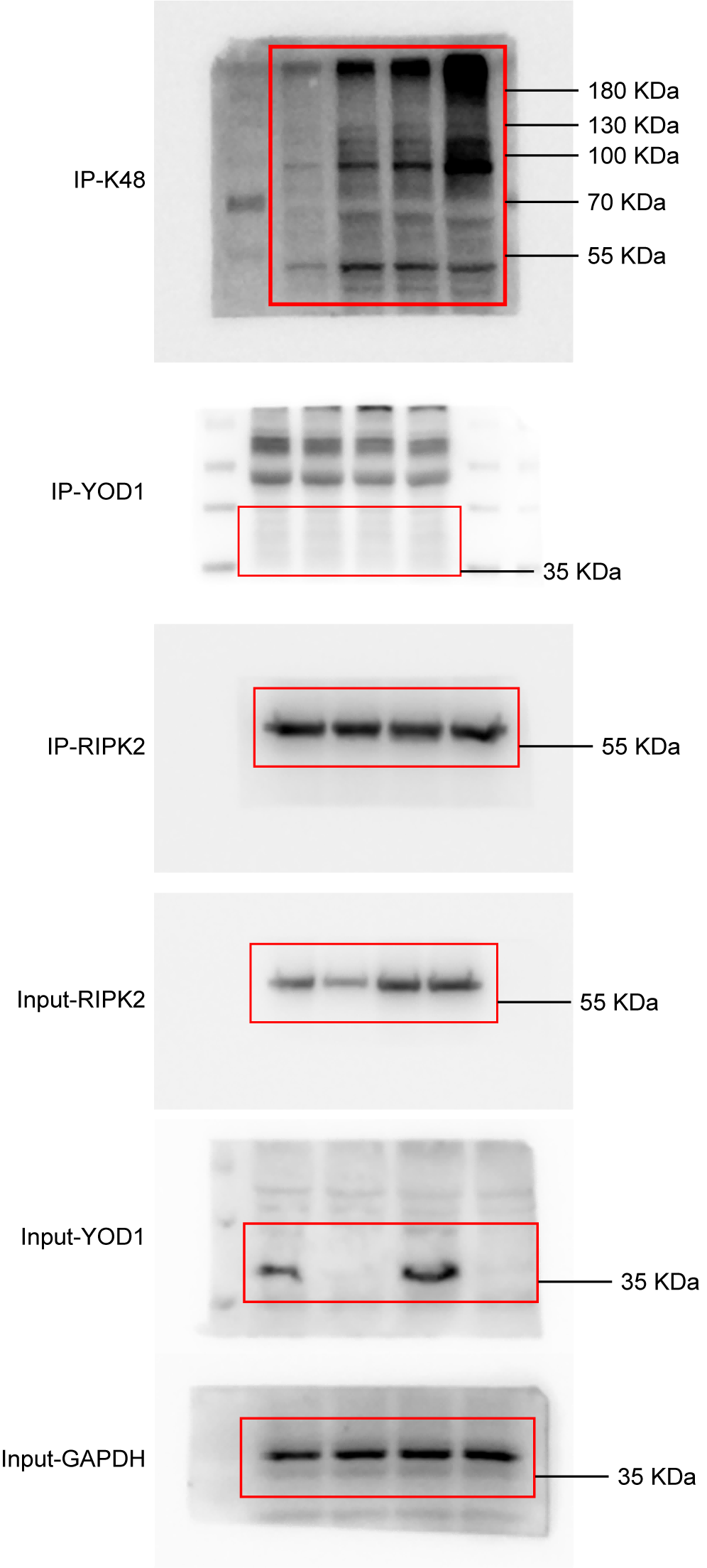

Supplement: Supplementary file 11 — Source data Fig. 6 [file 44319_2024_276_MOESM11_ESM.zip › Fig 6/6F/6F.png]

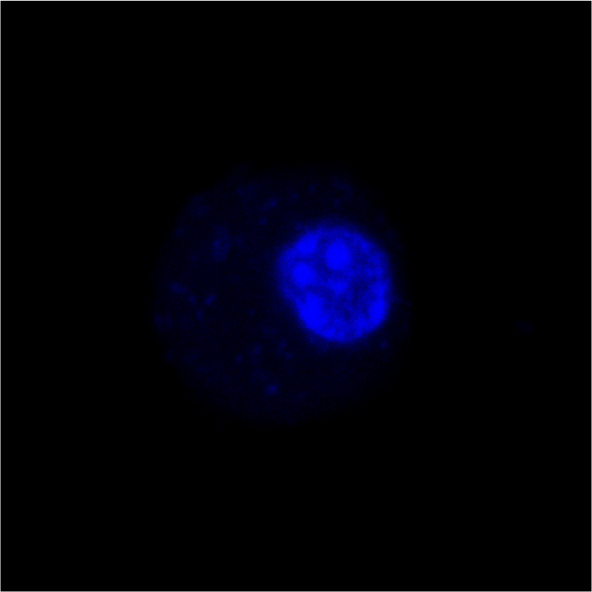

Supplement: Supplementary file 11 — Source data Fig. 6 [file 44319_2024_276_MOESM11_ESM.zip › Fig 6/6G/Yod1++_DAPI.png]

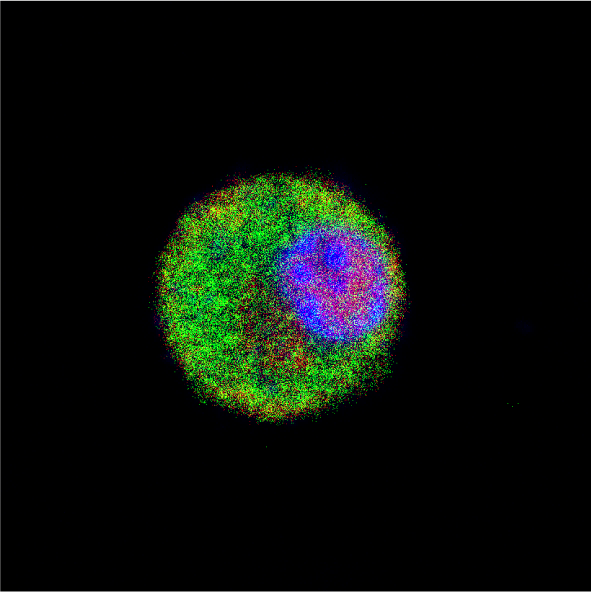

Supplement: Supplementary file 11 — Source data Fig. 6 [file 44319_2024_276_MOESM11_ESM.zip › Fig 6/6G/Yod1++_Merge.png]

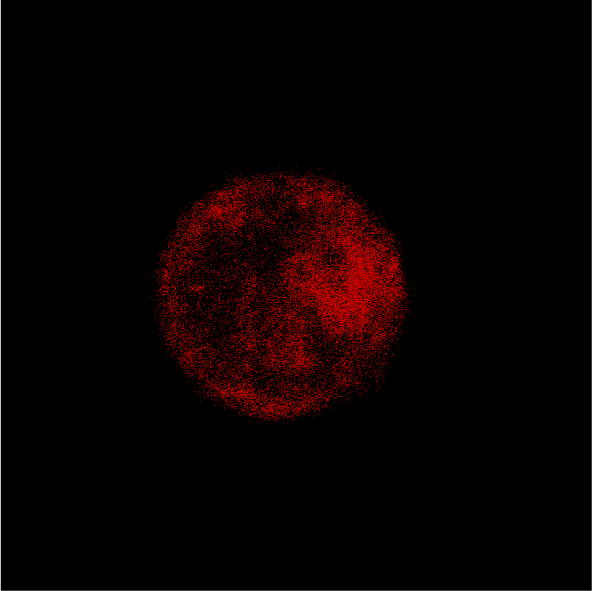

Supplement: Supplementary file 11 — Source data Fig. 6 [file 44319_2024_276_MOESM11_ESM.zip › Fig 6/6G/Yod1++_PSMD7.png]

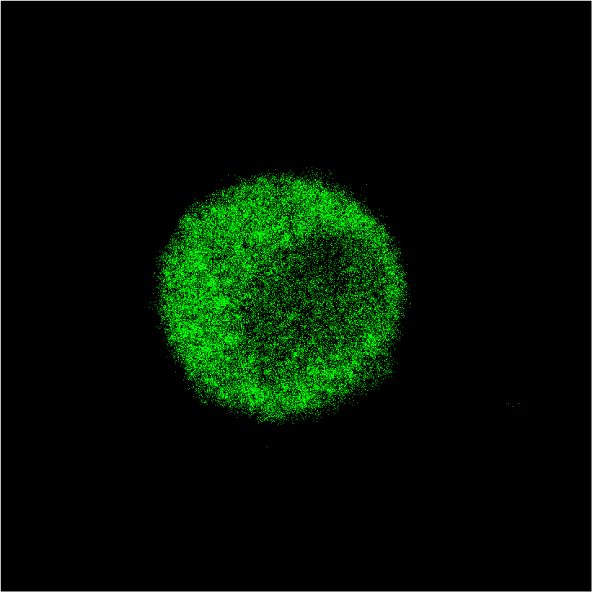

Supplement: Supplementary file 11 — Source data Fig. 6 [file 44319_2024_276_MOESM11_ESM.zip › Fig 6/6G/Yod1++_RIPK2.png]

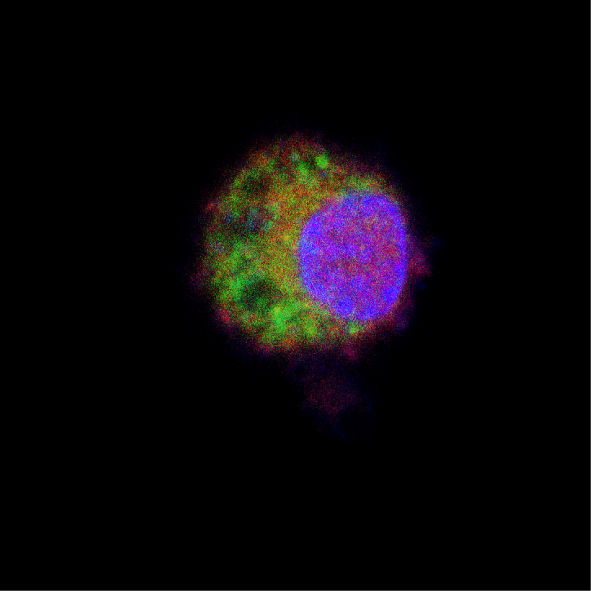

Supplement: Supplementary file 11 — Source data Fig. 6 [file 44319_2024_276_MOESM11_ESM.zip › Fig 6/6G/Yod1--_Merge.png]

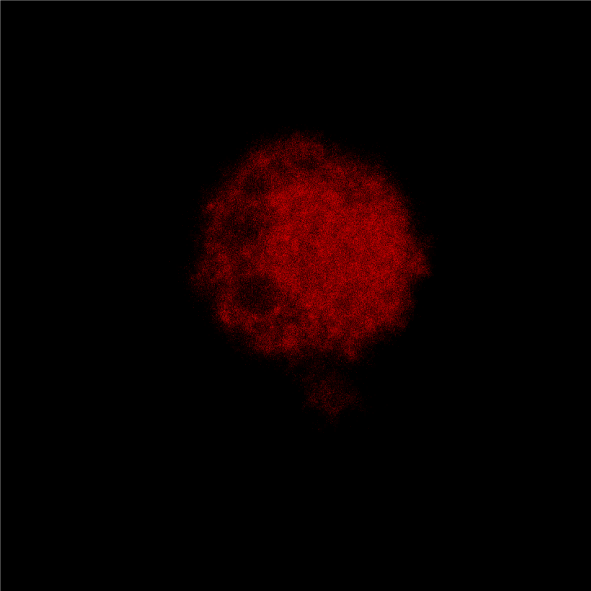

Supplement: Supplementary file 11 — Source data Fig. 6 [file 44319_2024_276_MOESM11_ESM.zip › Fig 6/6G/Yod1--_PSMD7.png]

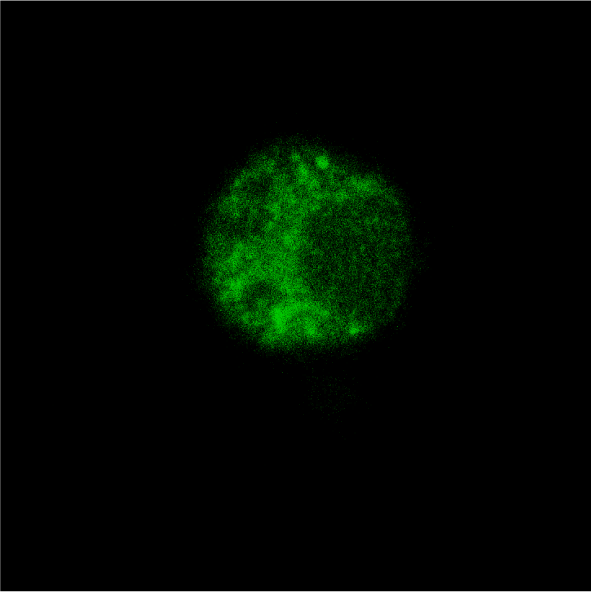

Supplement: Supplementary file 11 — Source data Fig. 6 [file 44319_2024_276_MOESM11_ESM.zip › Fig 6/6G/Yod1--_RIPK2.png]

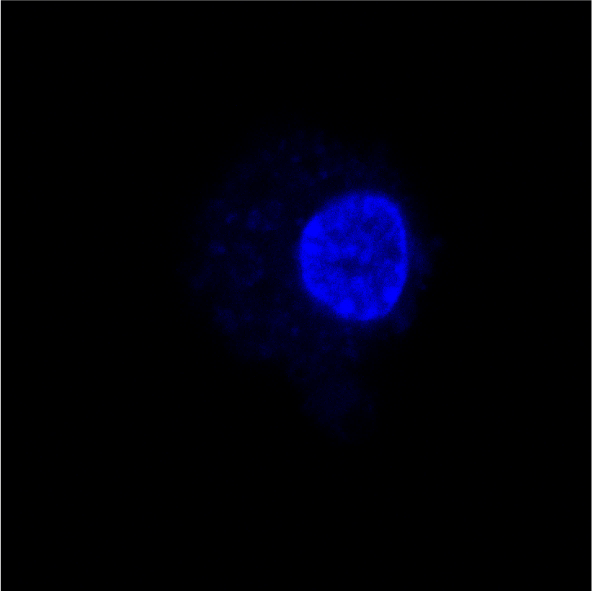

Supplement: Supplementary file 11 — Source data Fig. 6 [file 44319_2024_276_MOESM11_ESM.zip › Fig 6/6G/Yod1--DAPI.png]

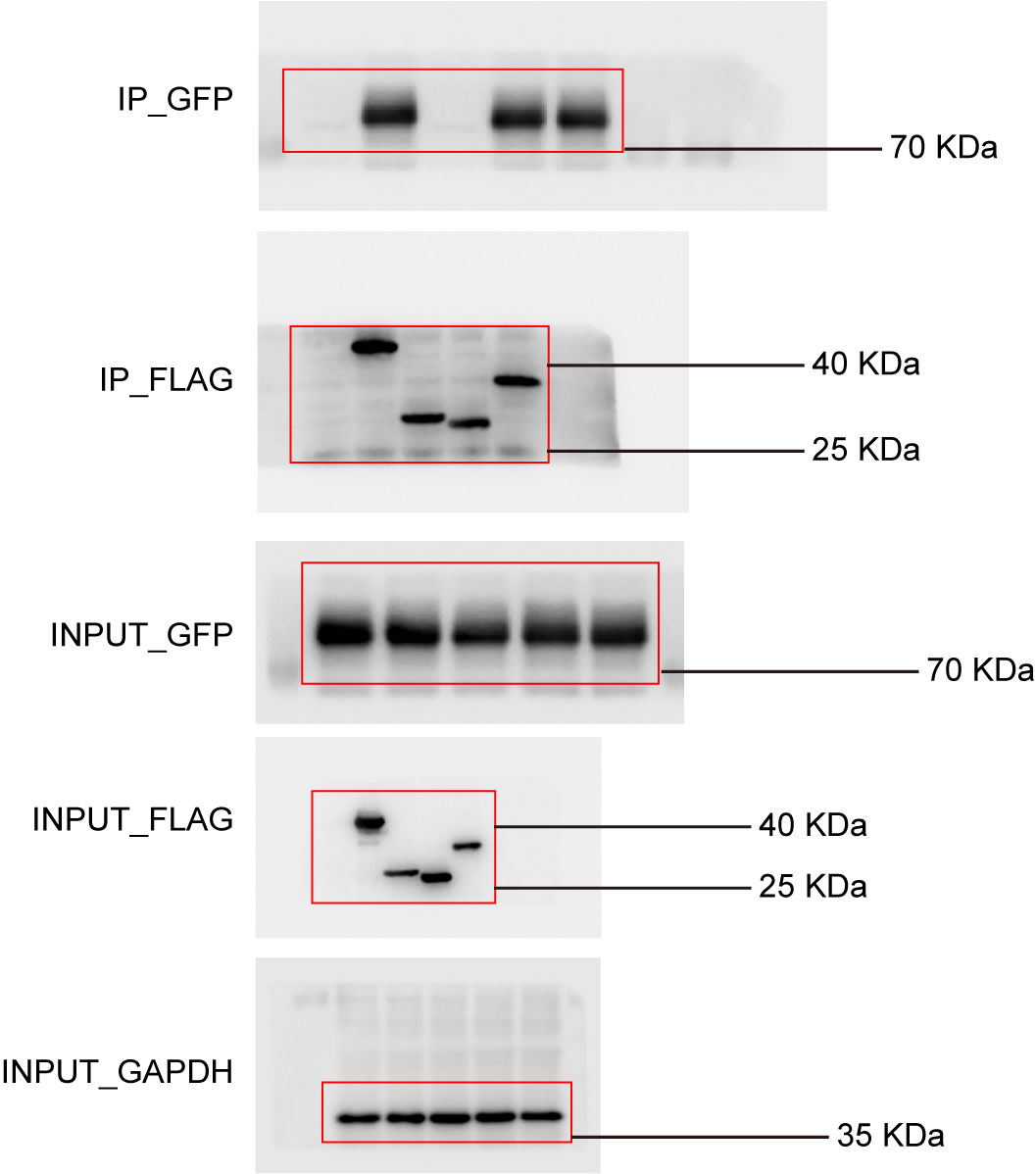

Supplement: Supplementary file 11 — Source data Fig. 6 [file 44319_2024_276_MOESM11_ESM.zip › Fig 6/6I/6I.png]

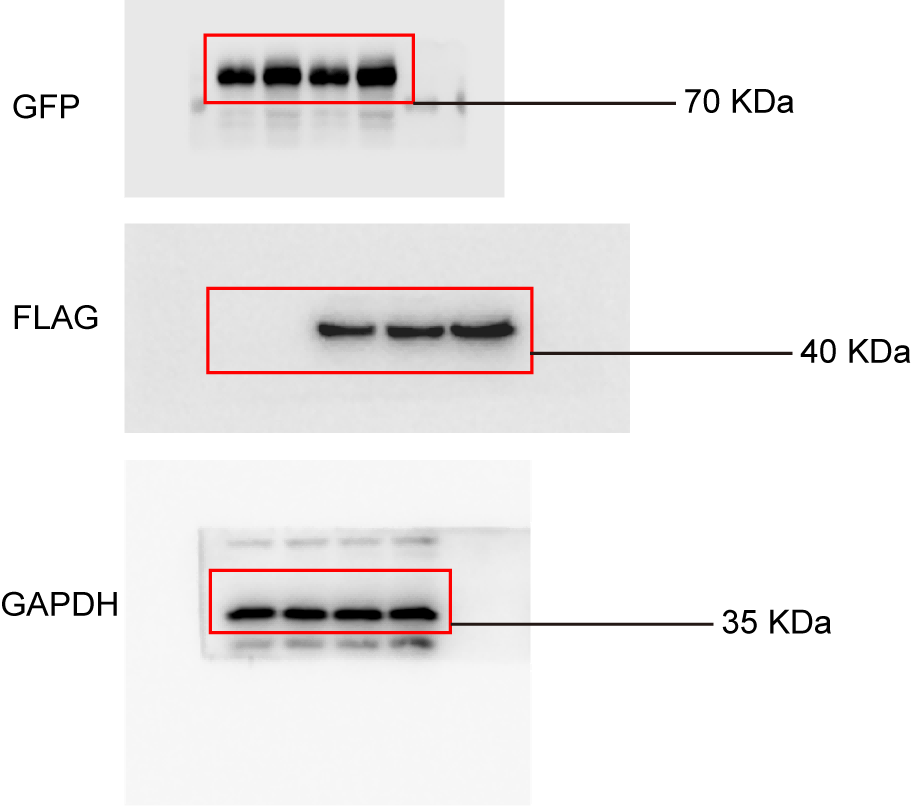

Supplement: Supplementary file 11 — Source data Fig. 6 [file 44319_2024_276_MOESM11_ESM.zip › Fig 6/6K/6K.png]

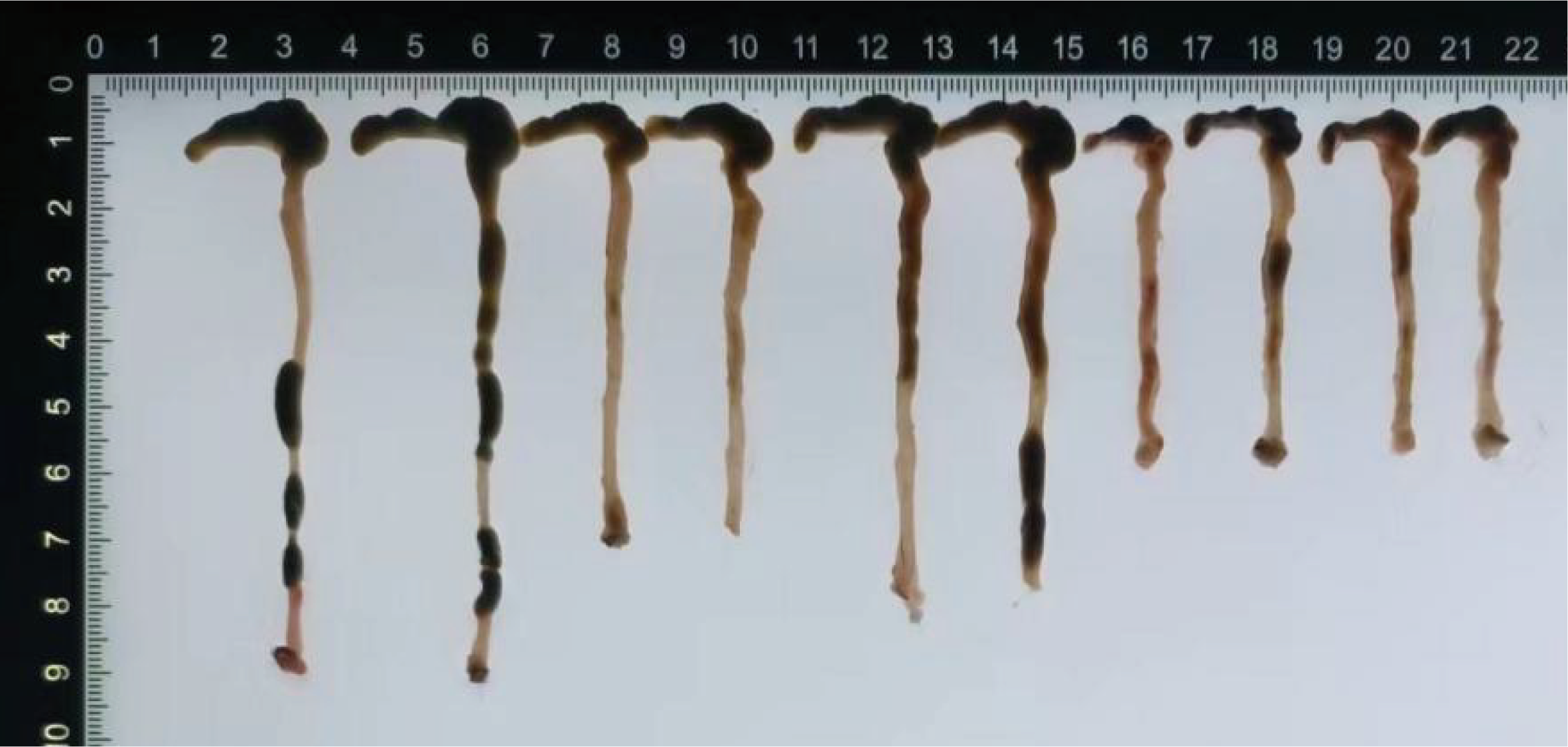

Supplement: Supplementary file 12 — Source data Fig. 7 [file 44319_2024_276_MOESM12_ESM.zip › Fig 7/7D/7D.tif]

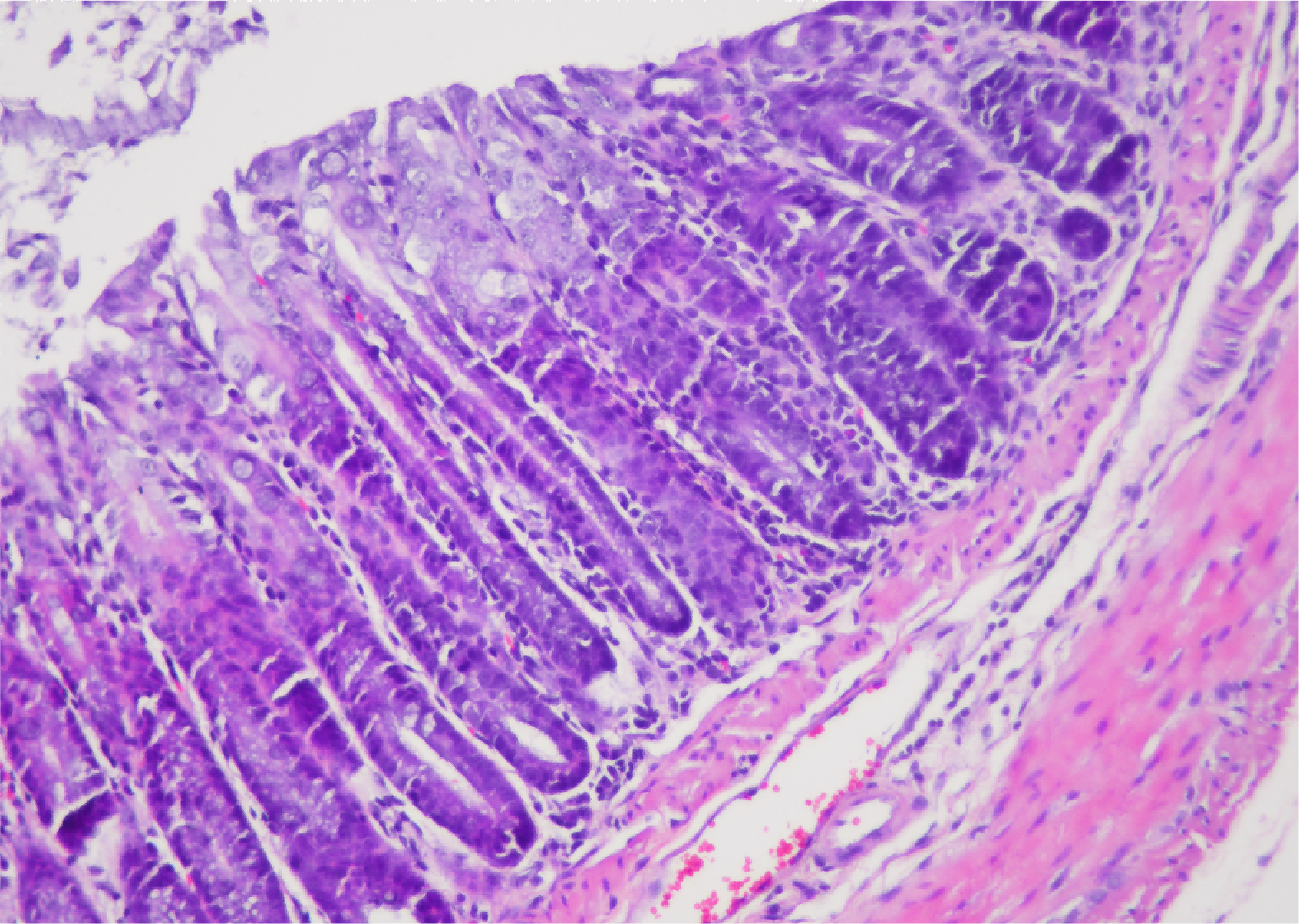

Supplement: Supplementary file 12 — Source data Fig. 7 [file 44319_2024_276_MOESM12_ESM.zip › Fig 7/7G/Yod1++_MDP_200×.png]

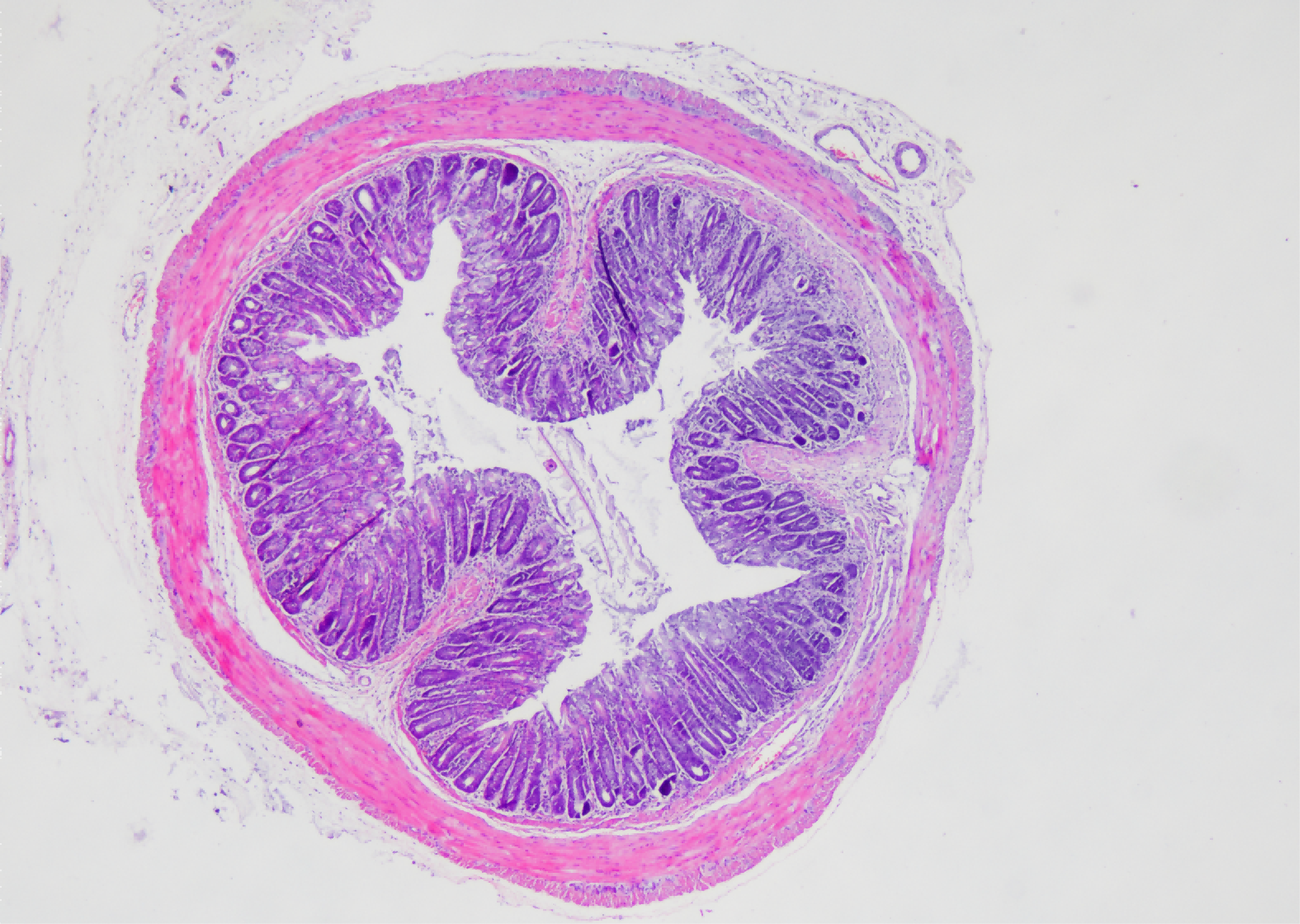

Supplement: Supplementary file 12 — Source data Fig. 7 [file 44319_2024_276_MOESM12_ESM.zip › Fig 7/7G/Yod1++_MDP_40×.png]

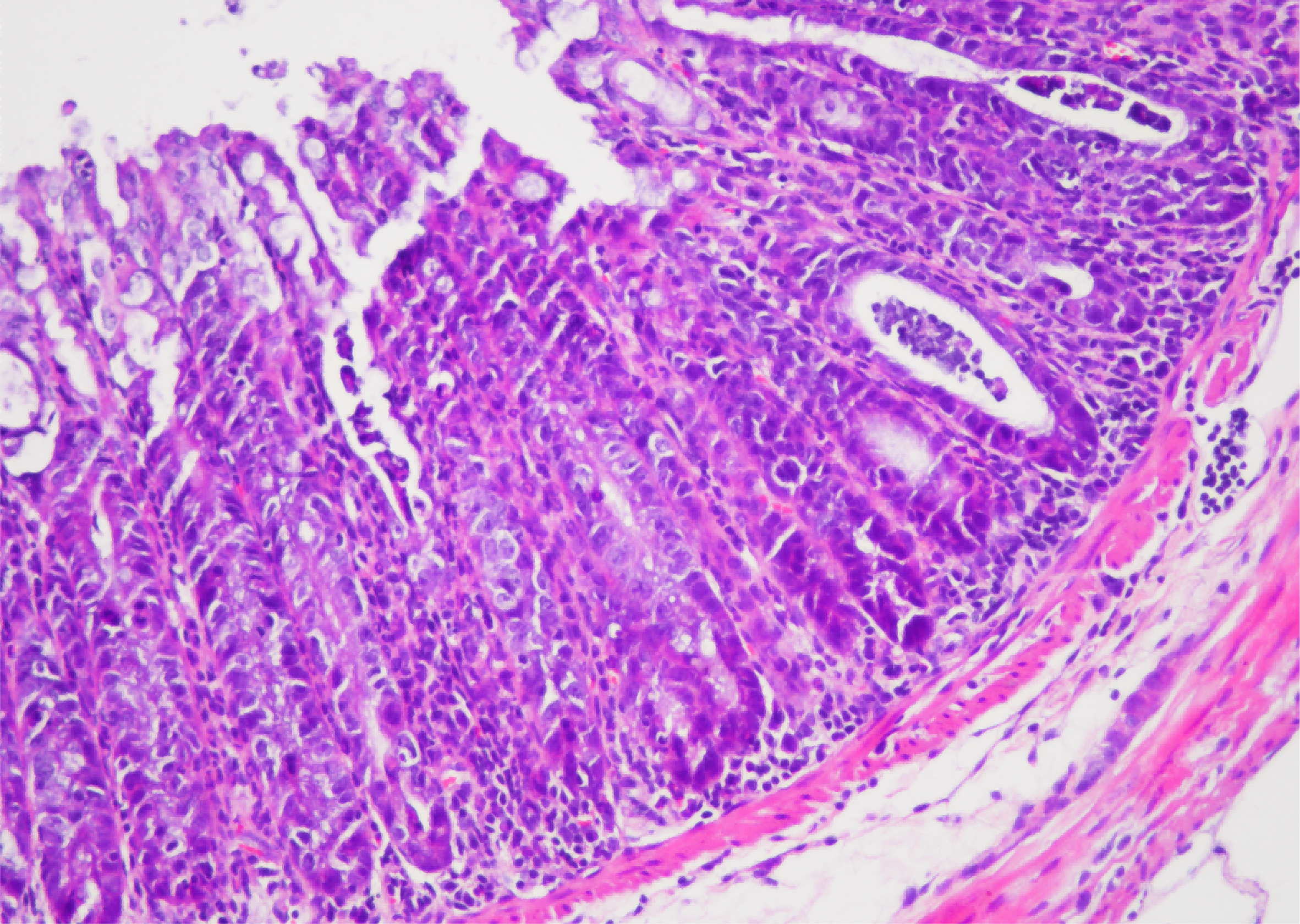

Supplement: Supplementary file 12 — Source data Fig. 7 [file 44319_2024_276_MOESM12_ESM.zip › Fig 7/7G/Yod1++_PBS_200×.png]

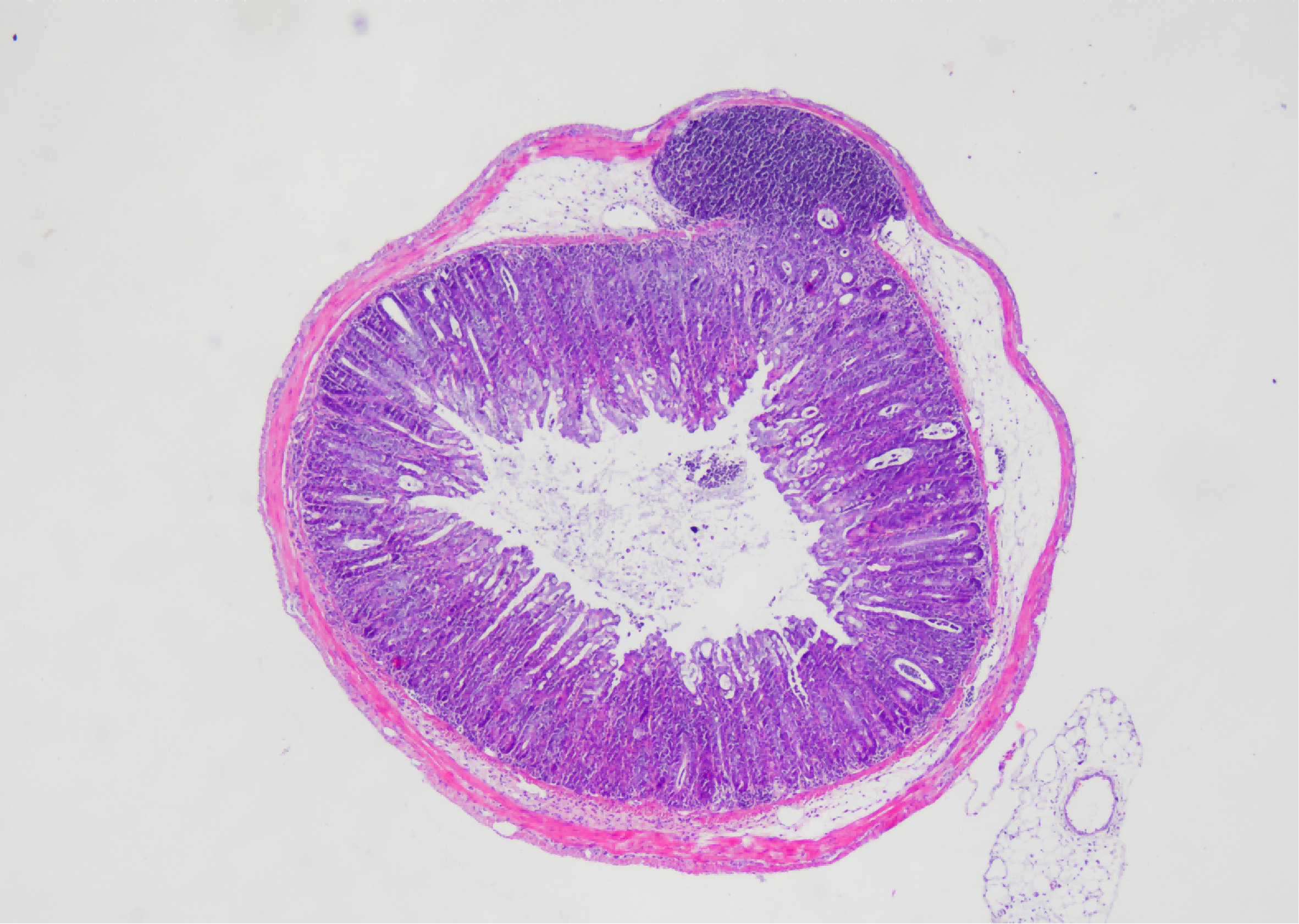

Supplement: Supplementary file 12 — Source data Fig. 7 [file 44319_2024_276_MOESM12_ESM.zip › Fig 7/7G/Yod1++_PBS_40×.png]
