# Supplementary figures and images for: YOD1 sustains NOD2-mediated protective signaling in colitis by stabilizing RIPK2 (part 2 of 3)
Source: EMBO Rep. 2024 Sep 27;25(11):4827–45. doi: 10.1038/s44319-024-00276-6 (PMC11549337; doi:10.1038/s44319-024-00276-6)

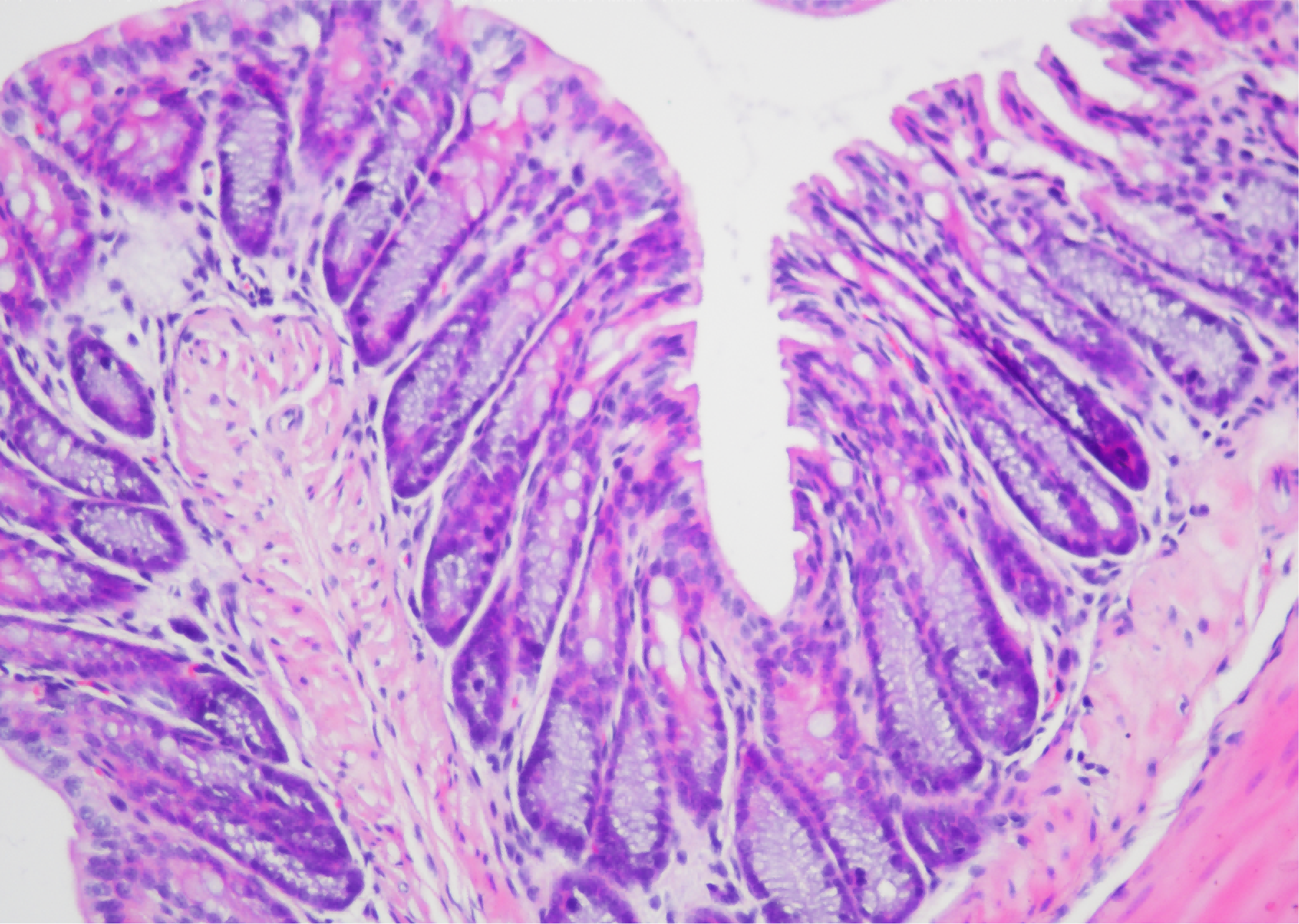

Supplement: Supplementary file 12 — Source data Fig. 7 [file 44319_2024_276_MOESM12_ESM.zip › Fig 7/7G/Yod1++_Water_200×.png]

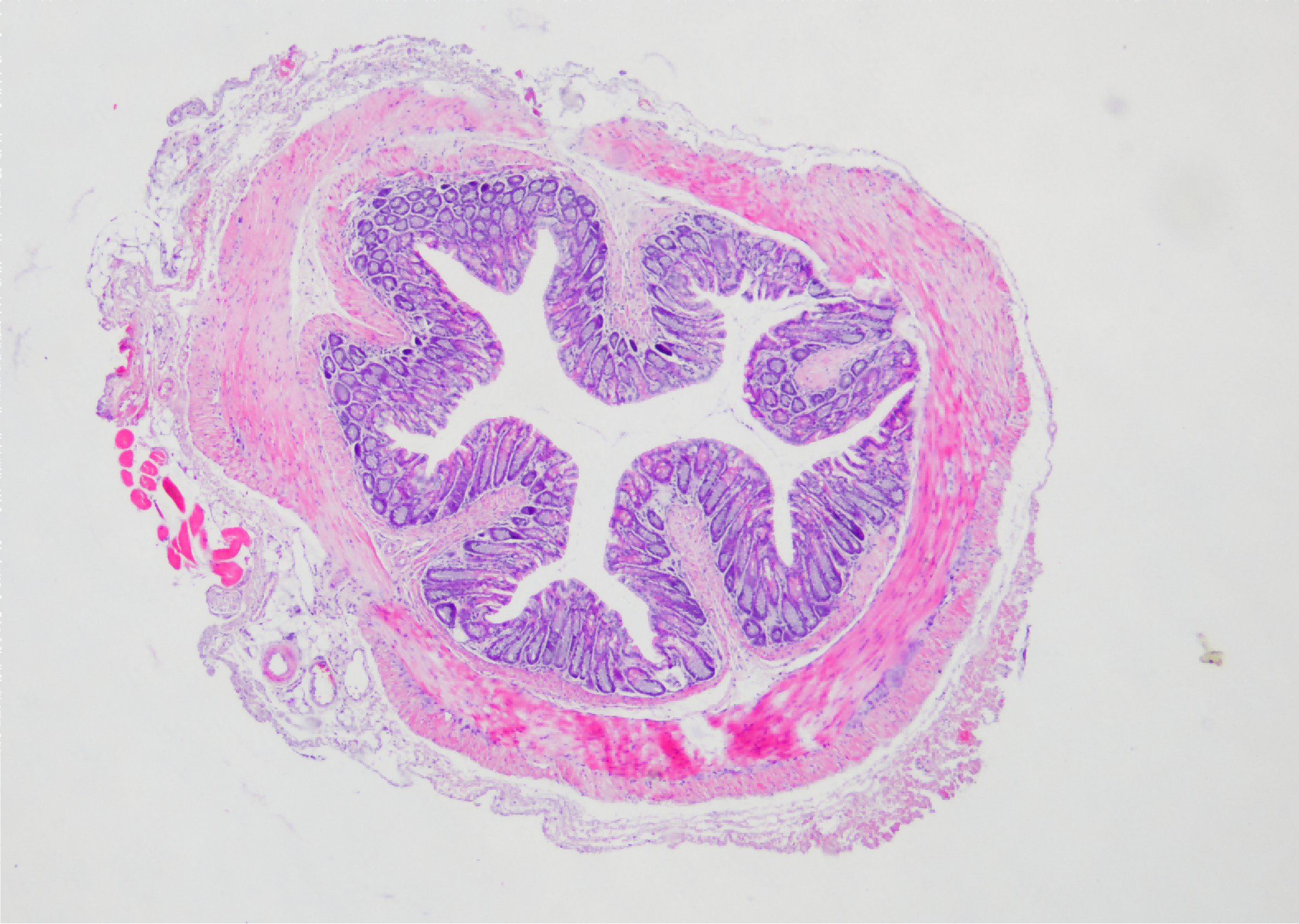

Supplement: Supplementary file 12 — Source data Fig. 7 [file 44319_2024_276_MOESM12_ESM.zip › Fig 7/7G/Yod1++_Water_40×.png]

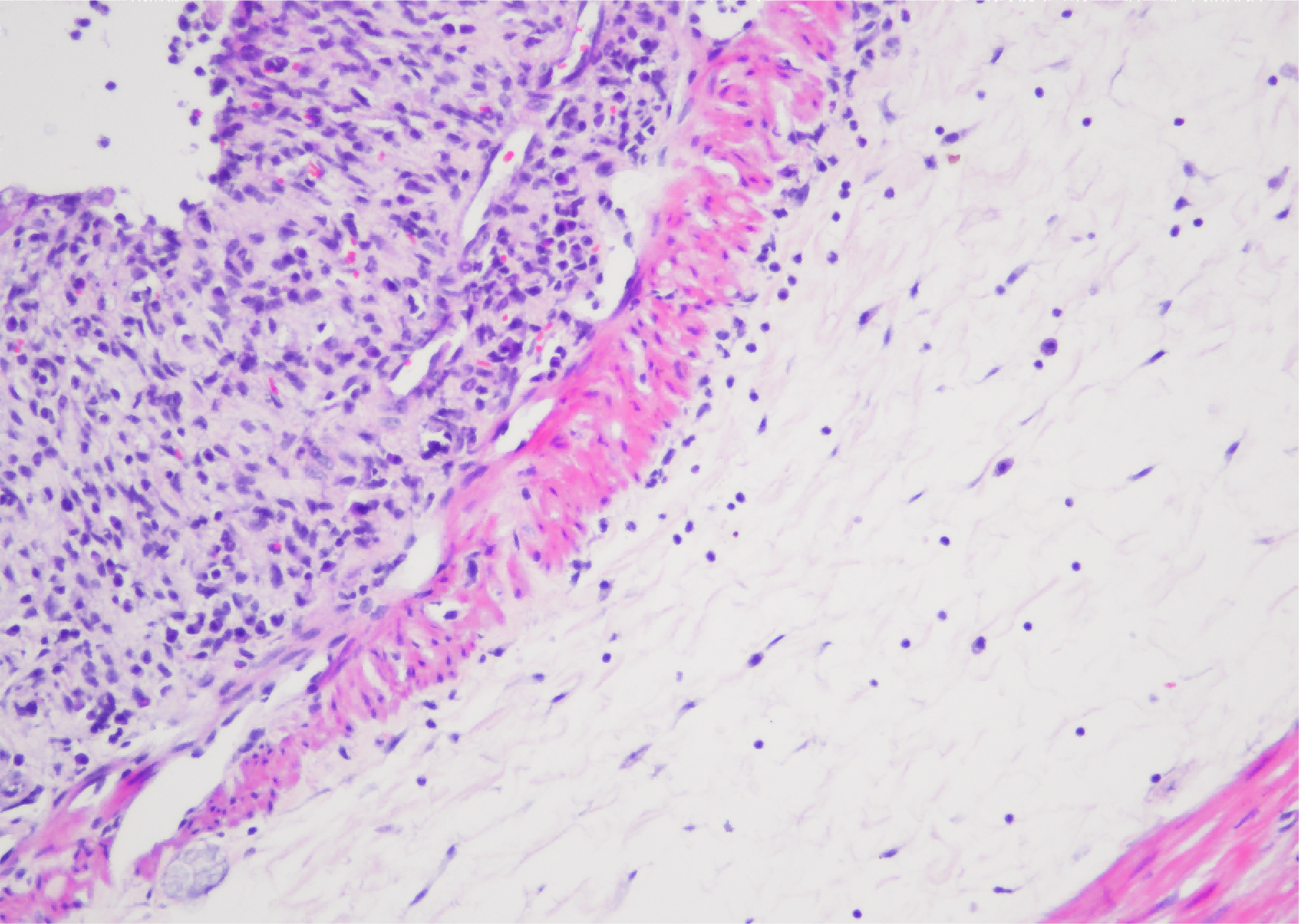

Supplement: Supplementary file 12 — Source data Fig. 7 [file 44319_2024_276_MOESM12_ESM.zip › Fig 7/7G/Yod1--_MDP_200×.png]

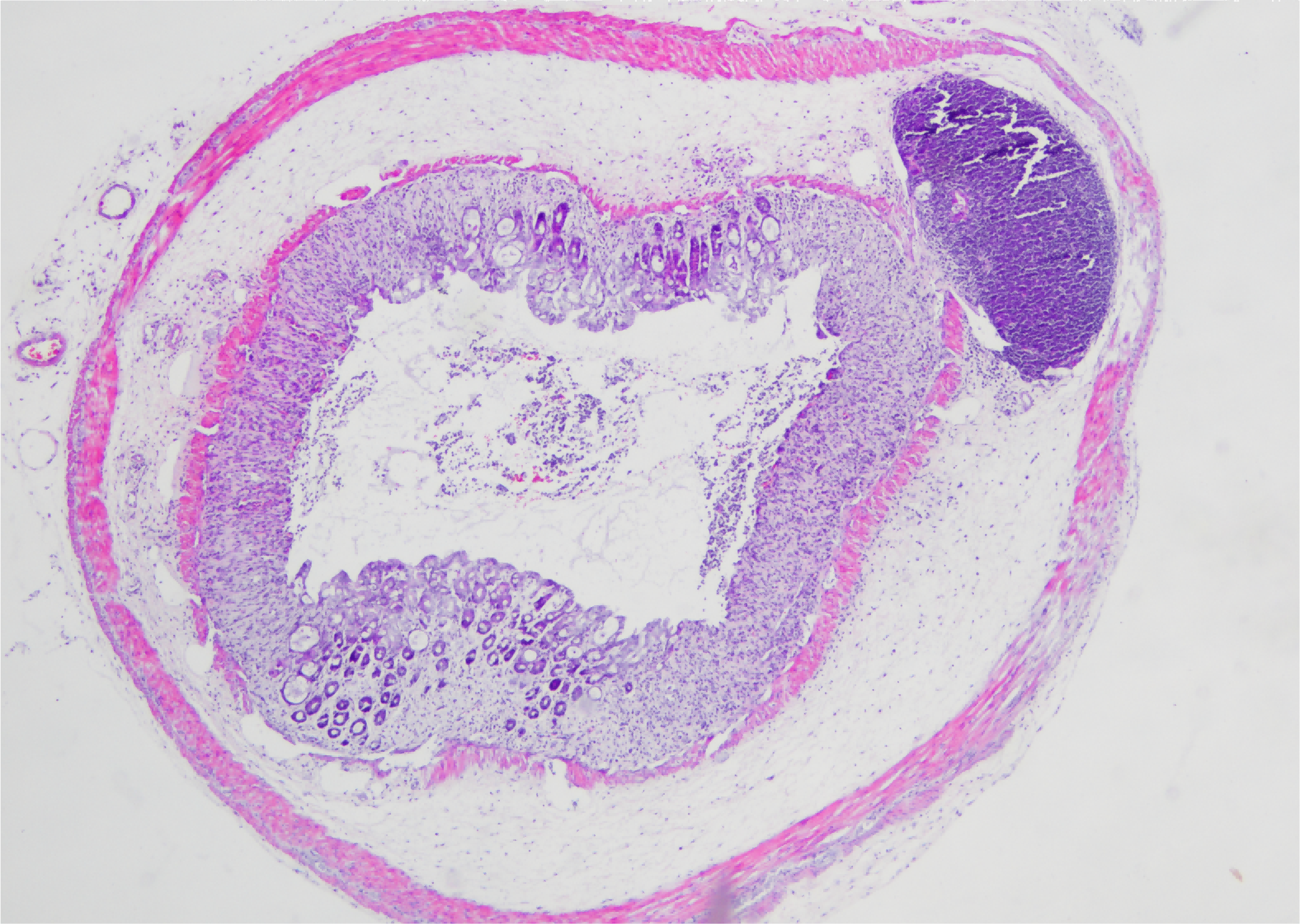

Supplement: Supplementary file 12 — Source data Fig. 7 [file 44319_2024_276_MOESM12_ESM.zip › Fig 7/7G/Yod1--_MDP_40×.png]

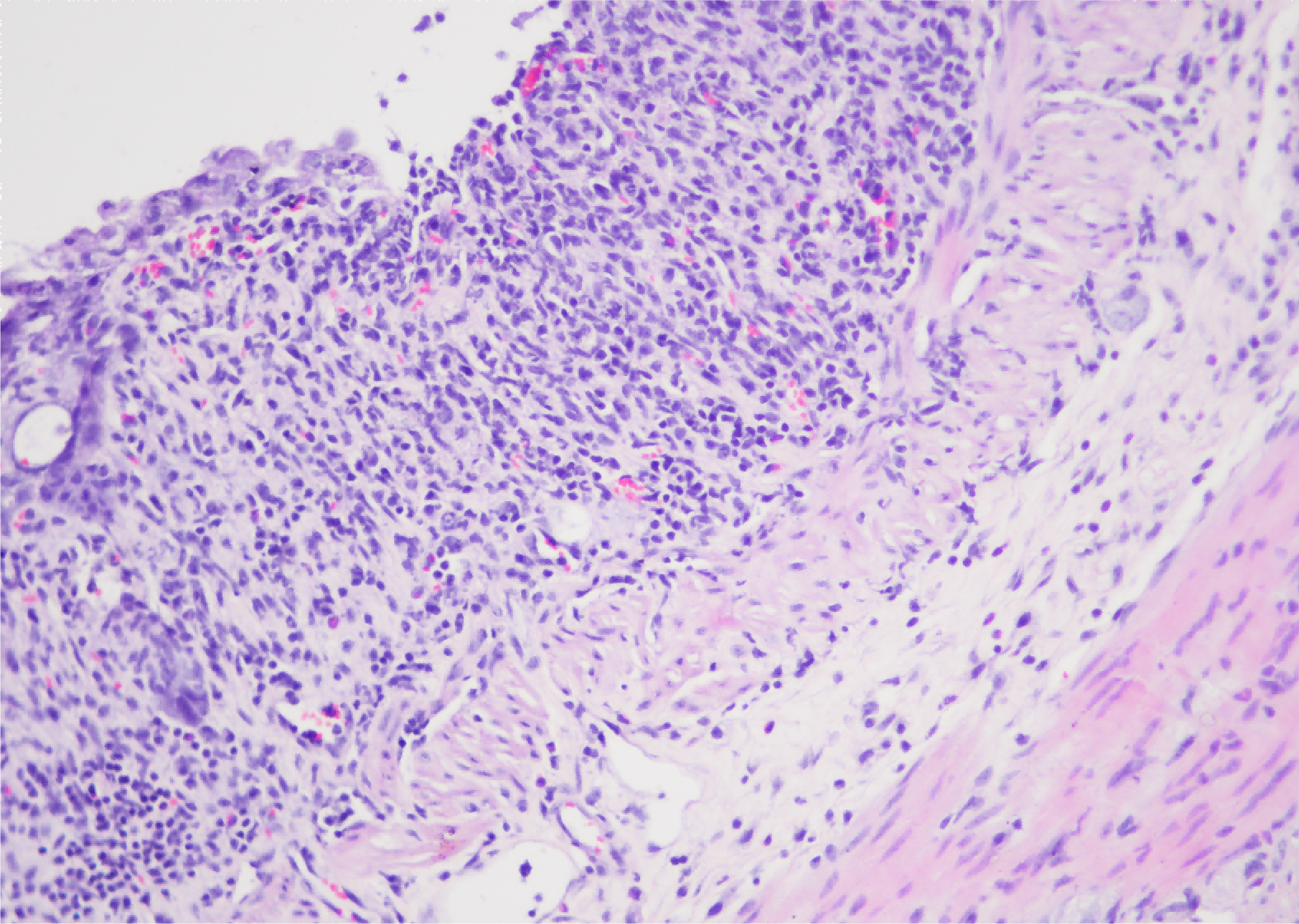

Supplement: Supplementary file 12 — Source data Fig. 7 [file 44319_2024_276_MOESM12_ESM.zip › Fig 7/7G/Yod1--_PBS_200×.png]

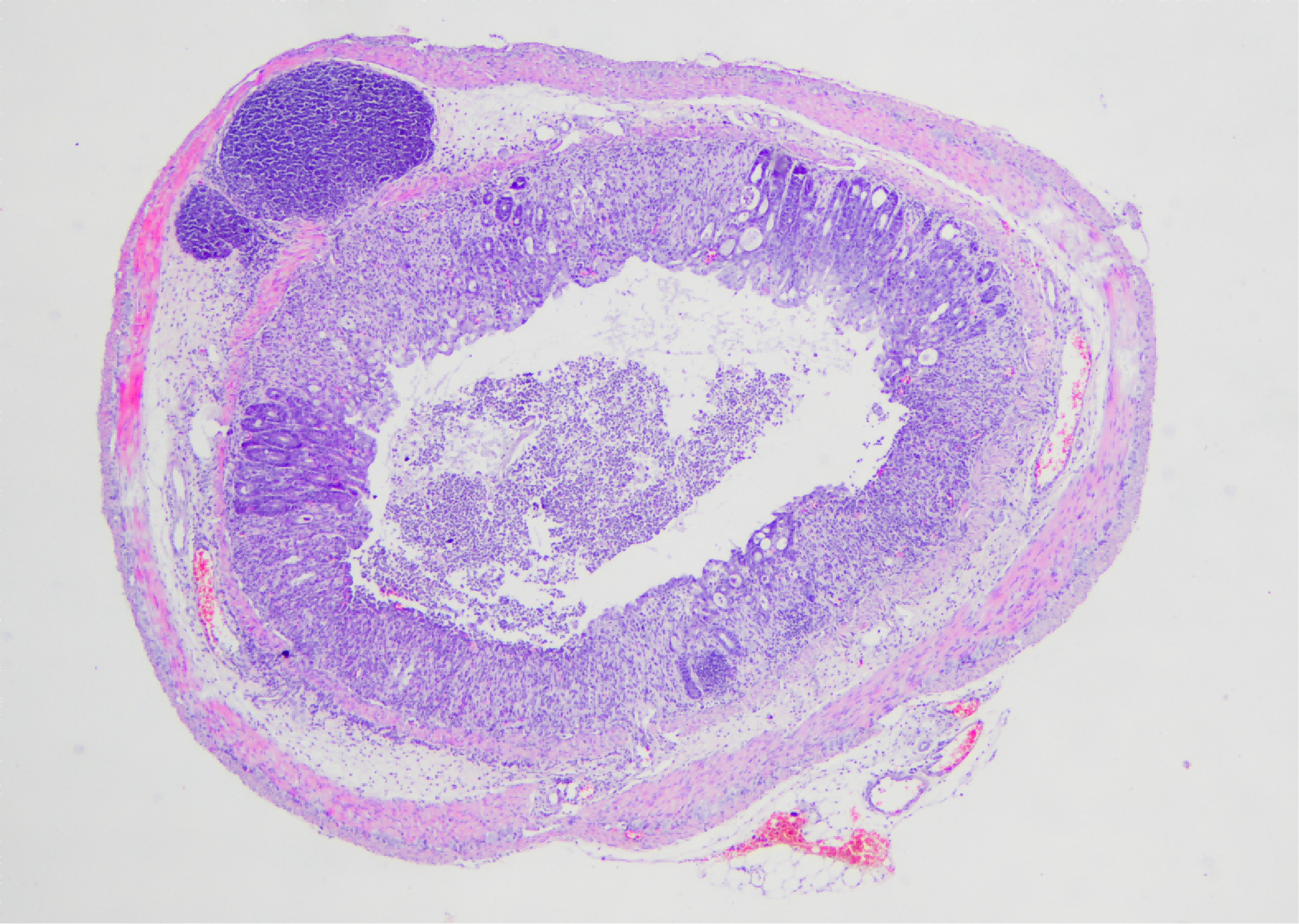

Supplement: Supplementary file 12 — Source data Fig. 7 [file 44319_2024_276_MOESM12_ESM.zip › Fig 7/7G/Yod1--_PBS_40×.png]

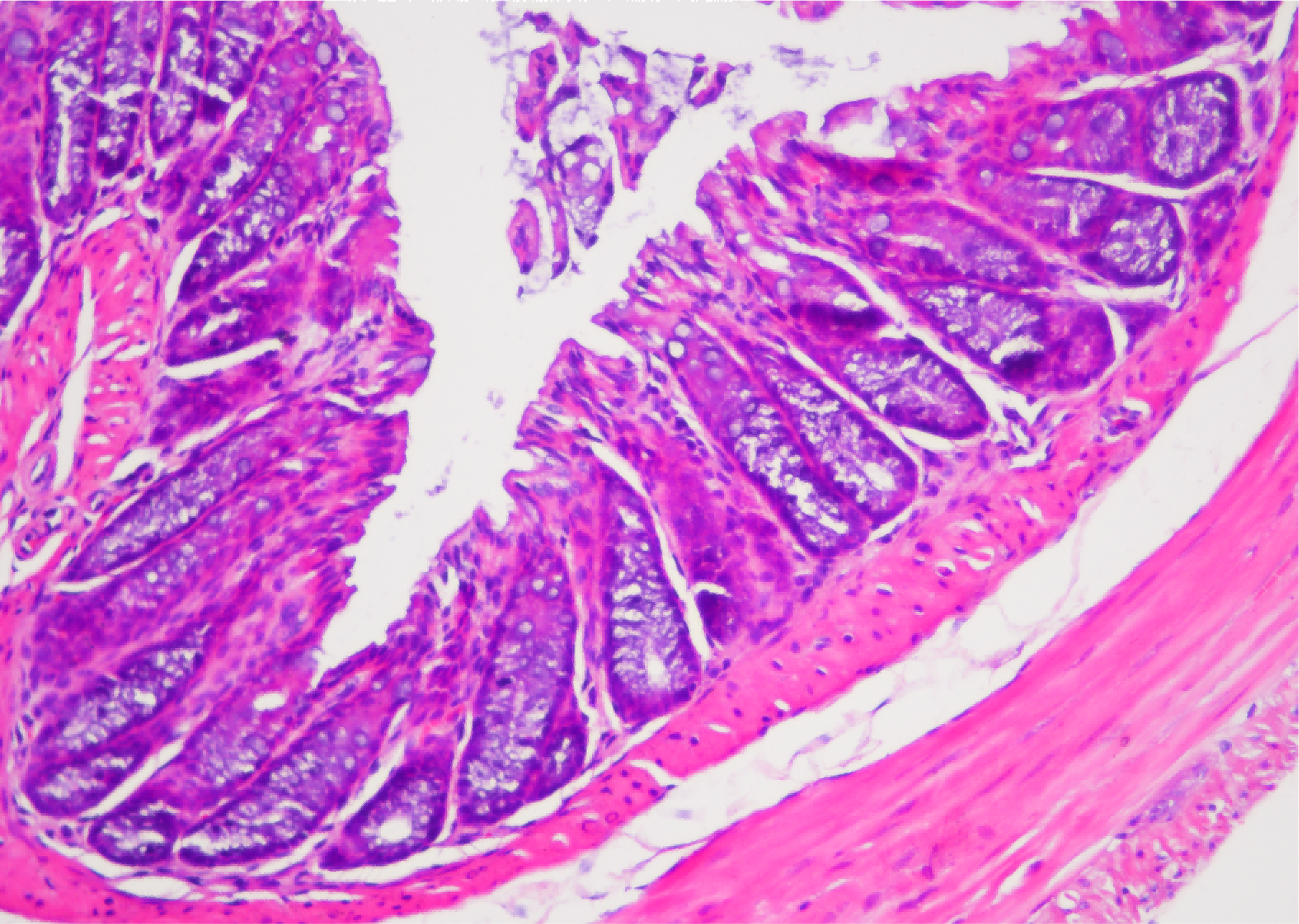

Supplement: Supplementary file 12 — Source data Fig. 7 [file 44319_2024_276_MOESM12_ESM.zip › Fig 7/7G/Yod1--_Water_200×.png]

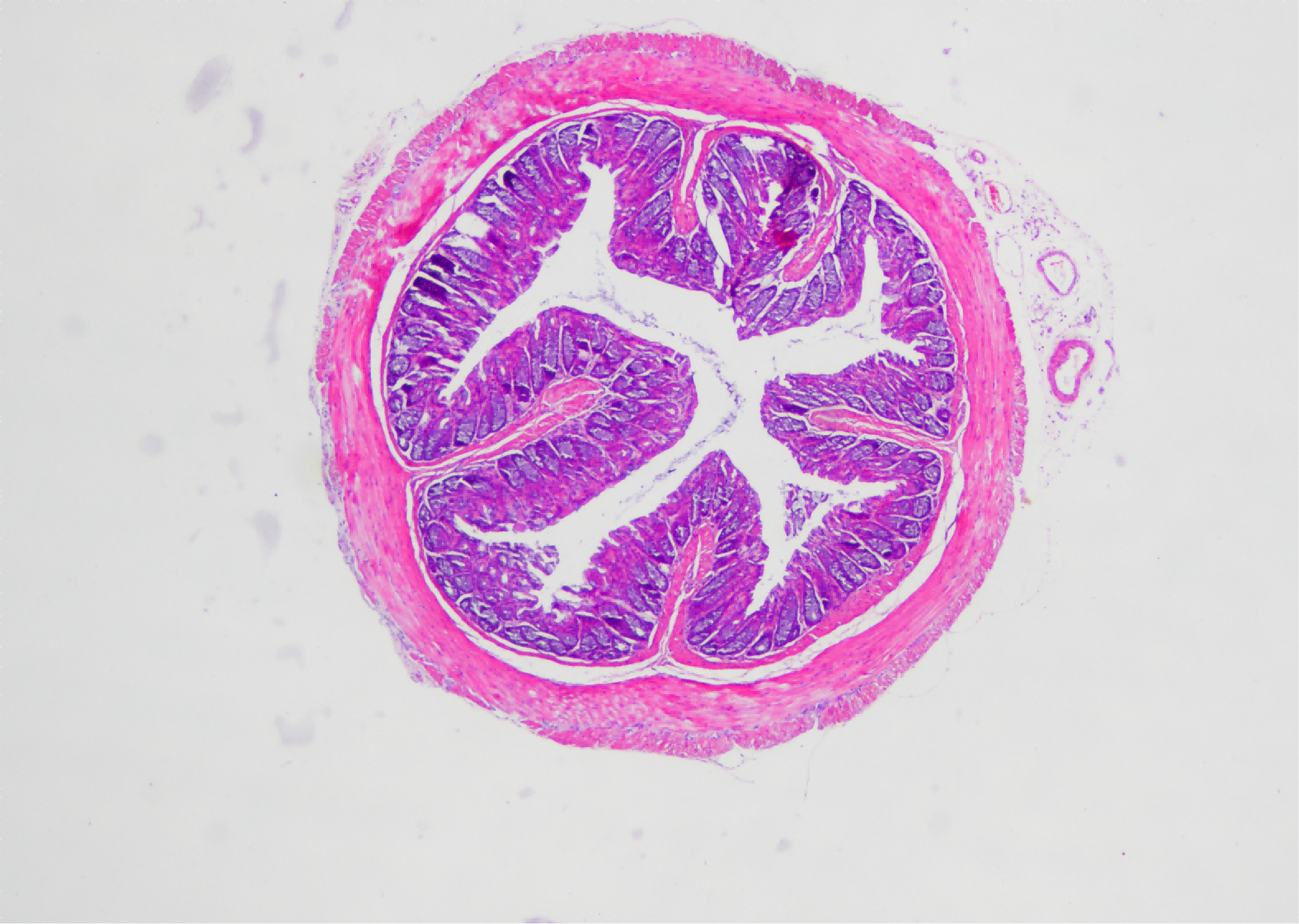

Supplement: Supplementary file 12 — Source data Fig. 7 [file 44319_2024_276_MOESM12_ESM.zip › Fig 7/7G/Yod1--_Water_40×.png]

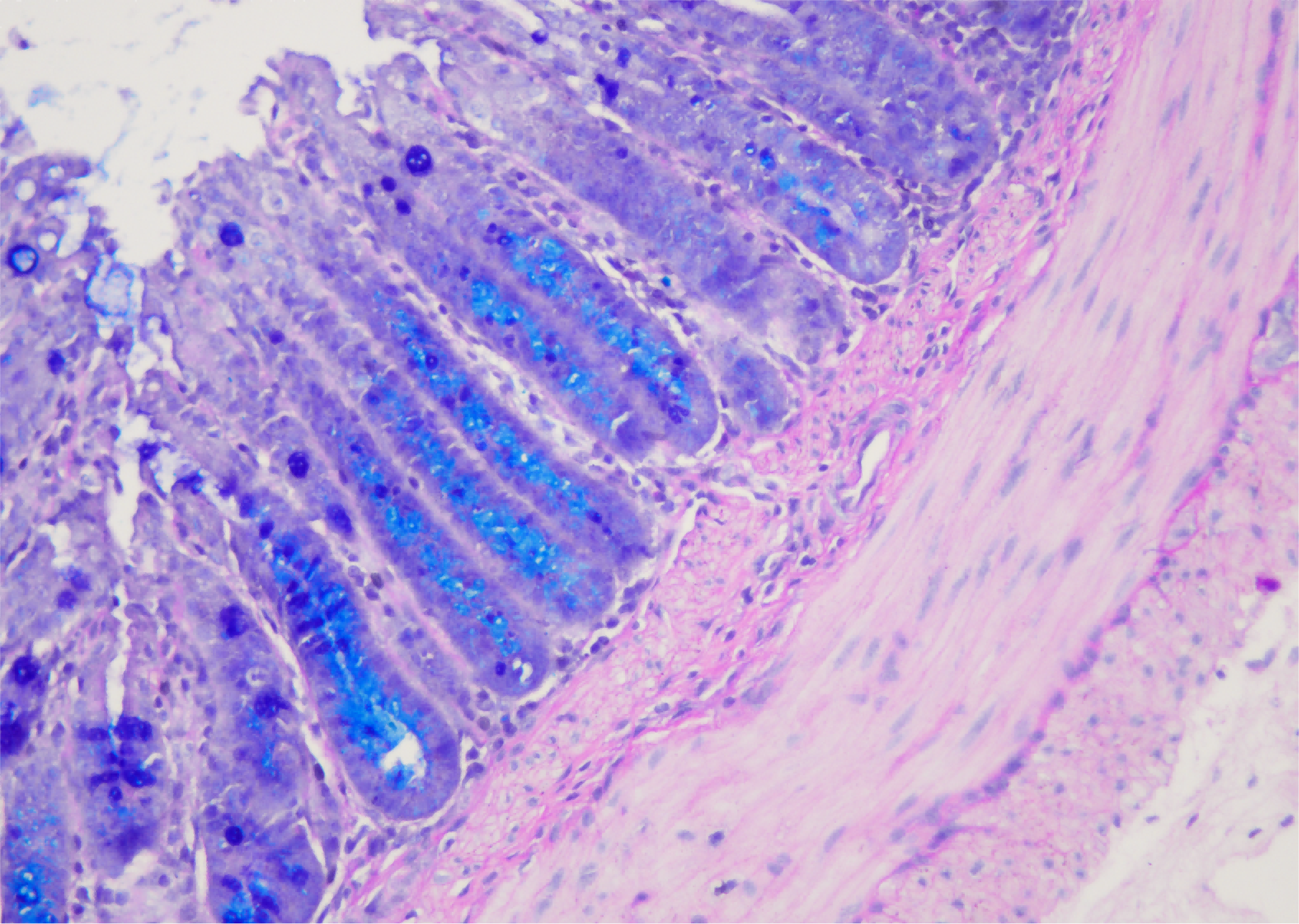

Supplement: Supplementary file 12 — Source data Fig. 7 [file 44319_2024_276_MOESM12_ESM.zip › Fig 7/7H/Yod1++_DSS_MDP_200×.png]

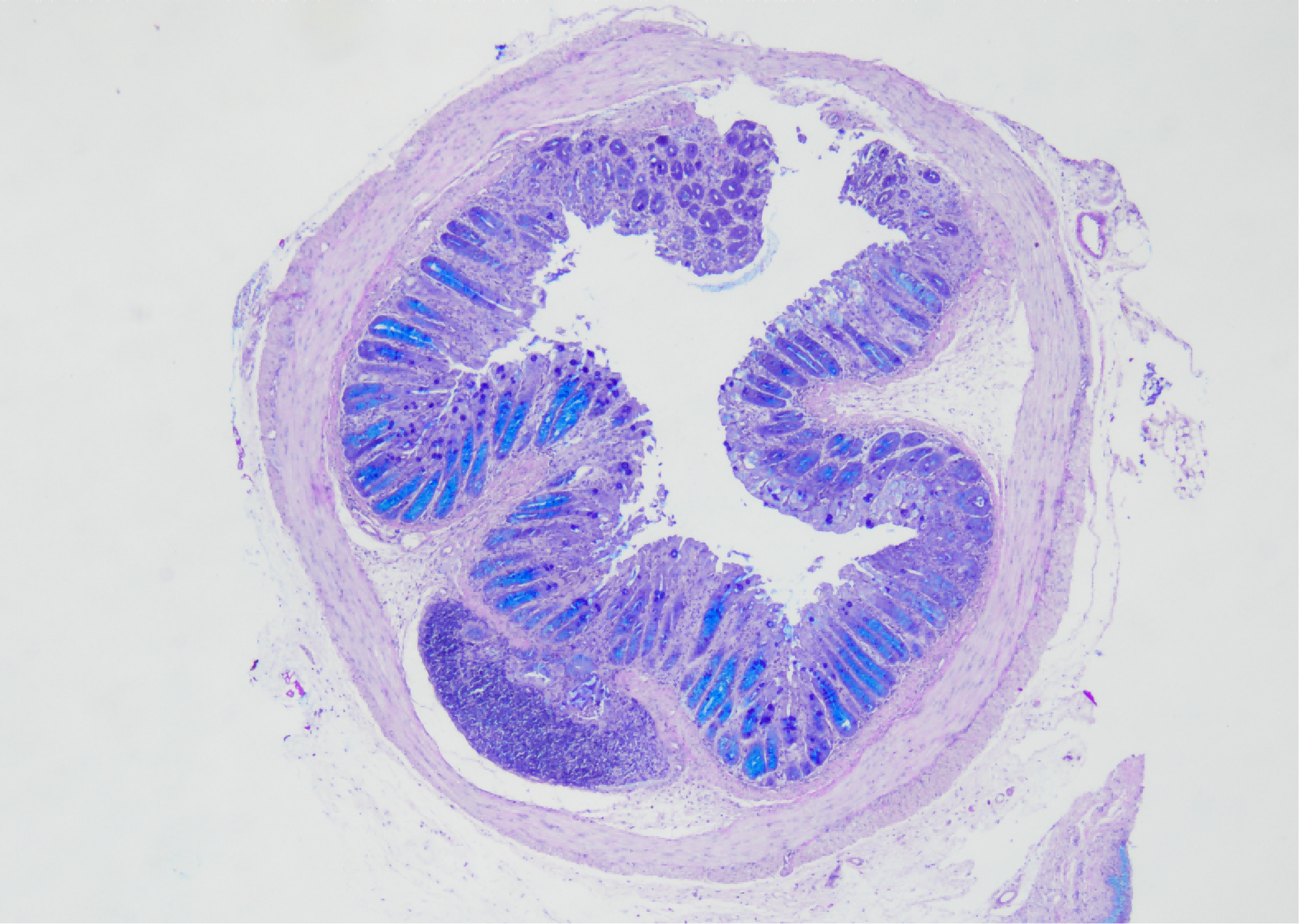

Supplement: Supplementary file 12 — Source data Fig. 7 [file 44319_2024_276_MOESM12_ESM.zip › Fig 7/7H/Yod1++_DSS_MDP_40×.png]

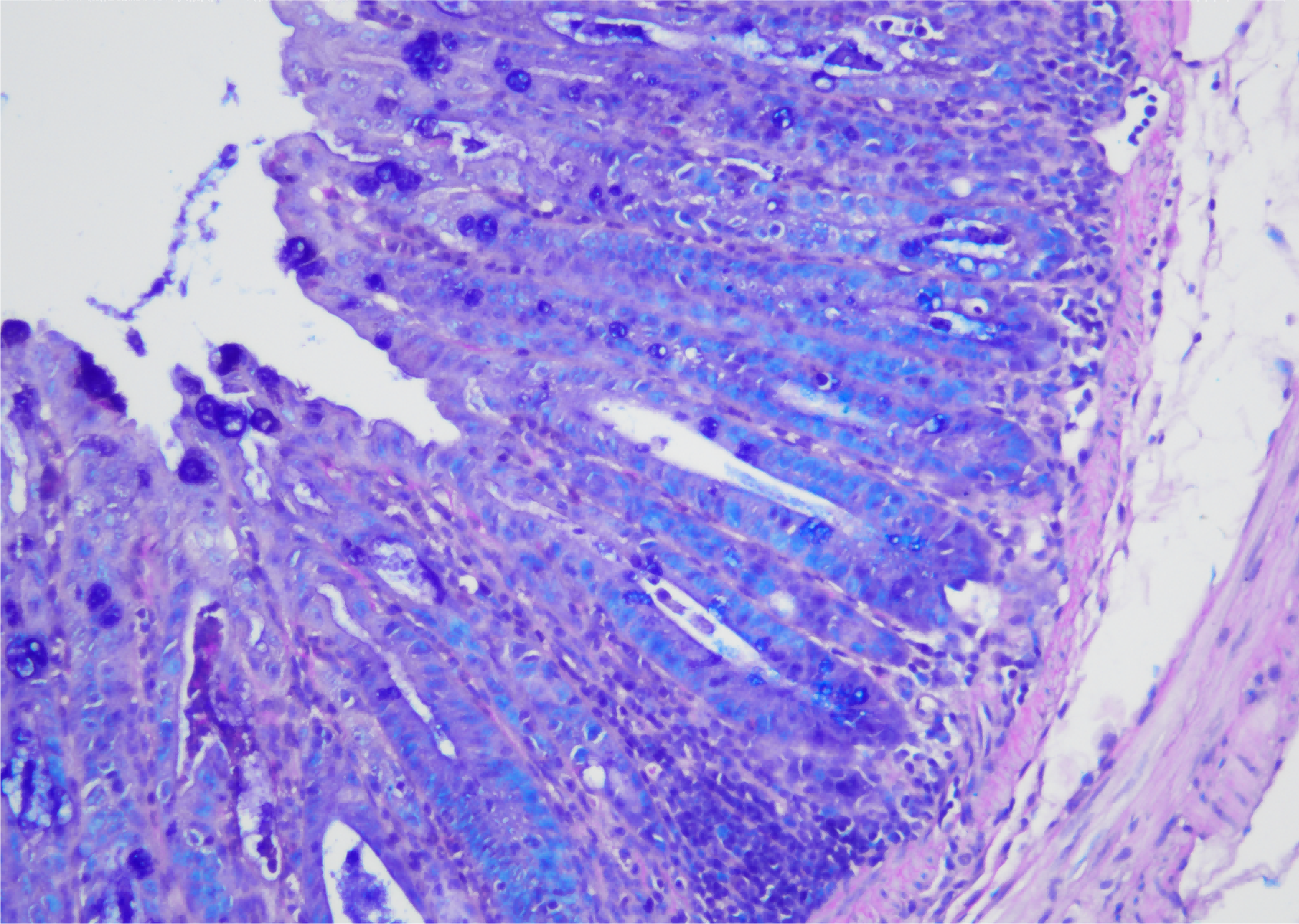

Supplement: Supplementary file 12 — Source data Fig. 7 [file 44319_2024_276_MOESM12_ESM.zip › Fig 7/7H/Yod1++_DSS_PBS_200×.png]

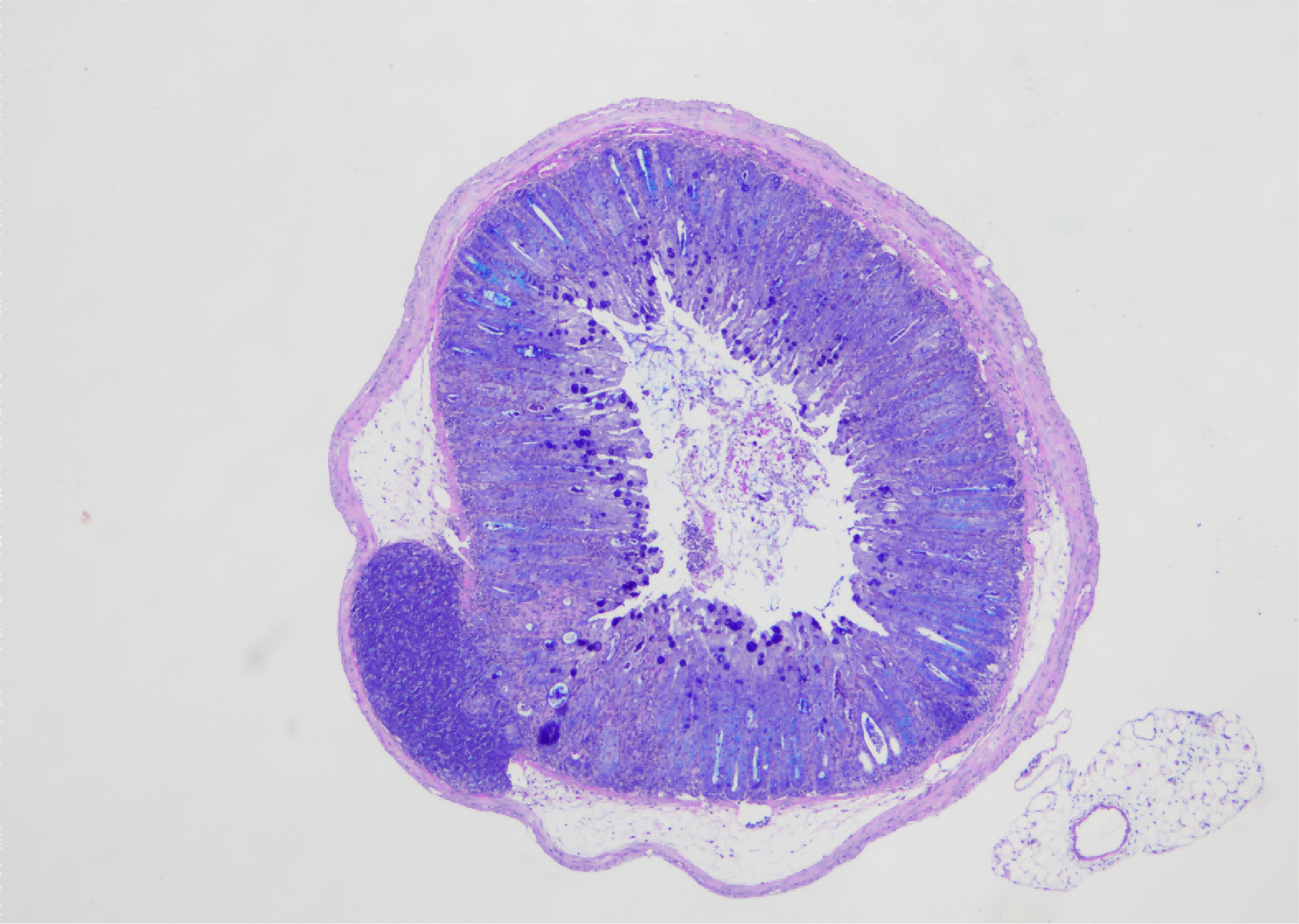

Supplement: Supplementary file 12 — Source data Fig. 7 [file 44319_2024_276_MOESM12_ESM.zip › Fig 7/7H/Yod1++_DSS_PBS_40×.png]

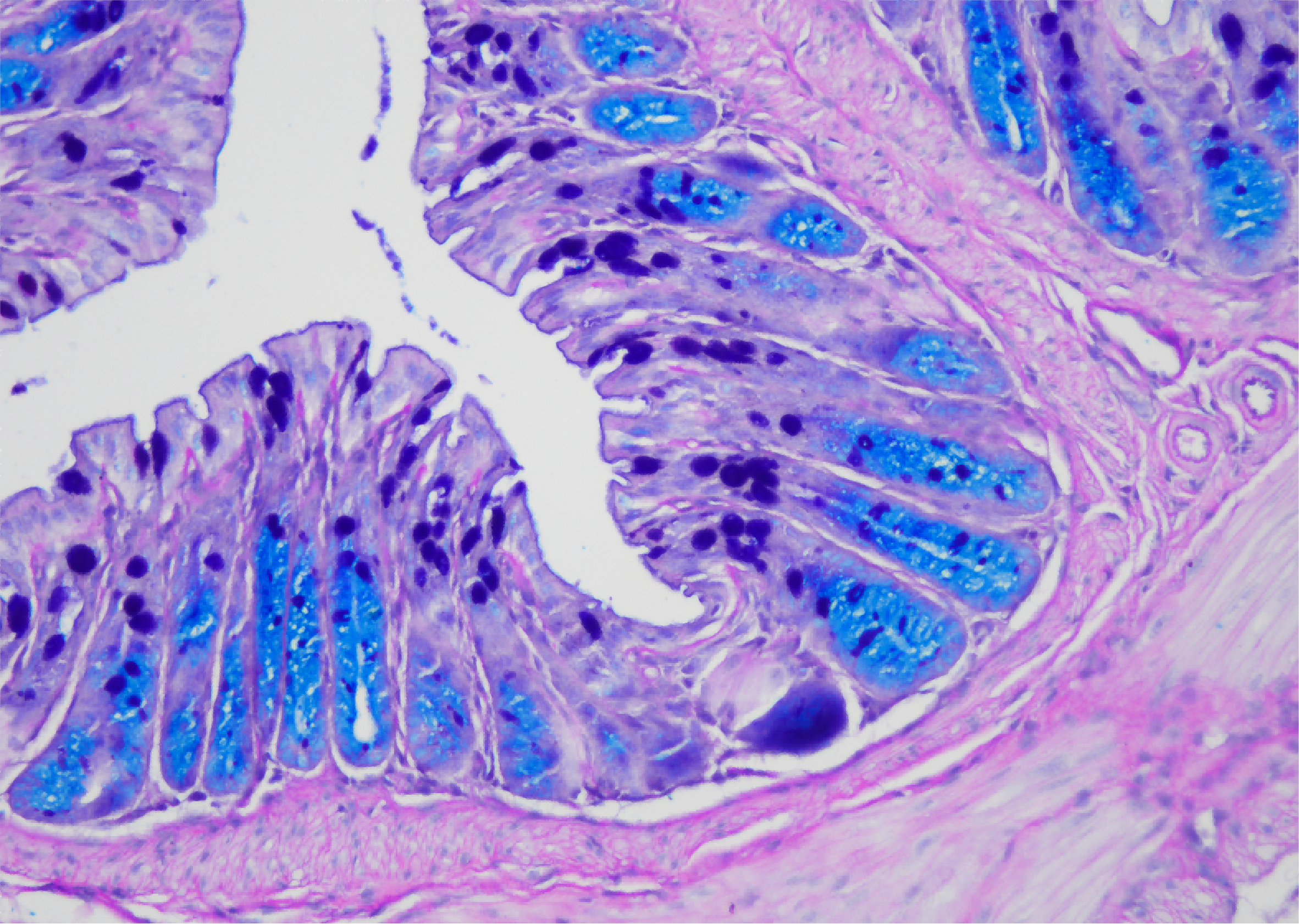

Supplement: Supplementary file 12 — Source data Fig. 7 [file 44319_2024_276_MOESM12_ESM.zip › Fig 7/7H/Yod1++_Water_200×(1).png]

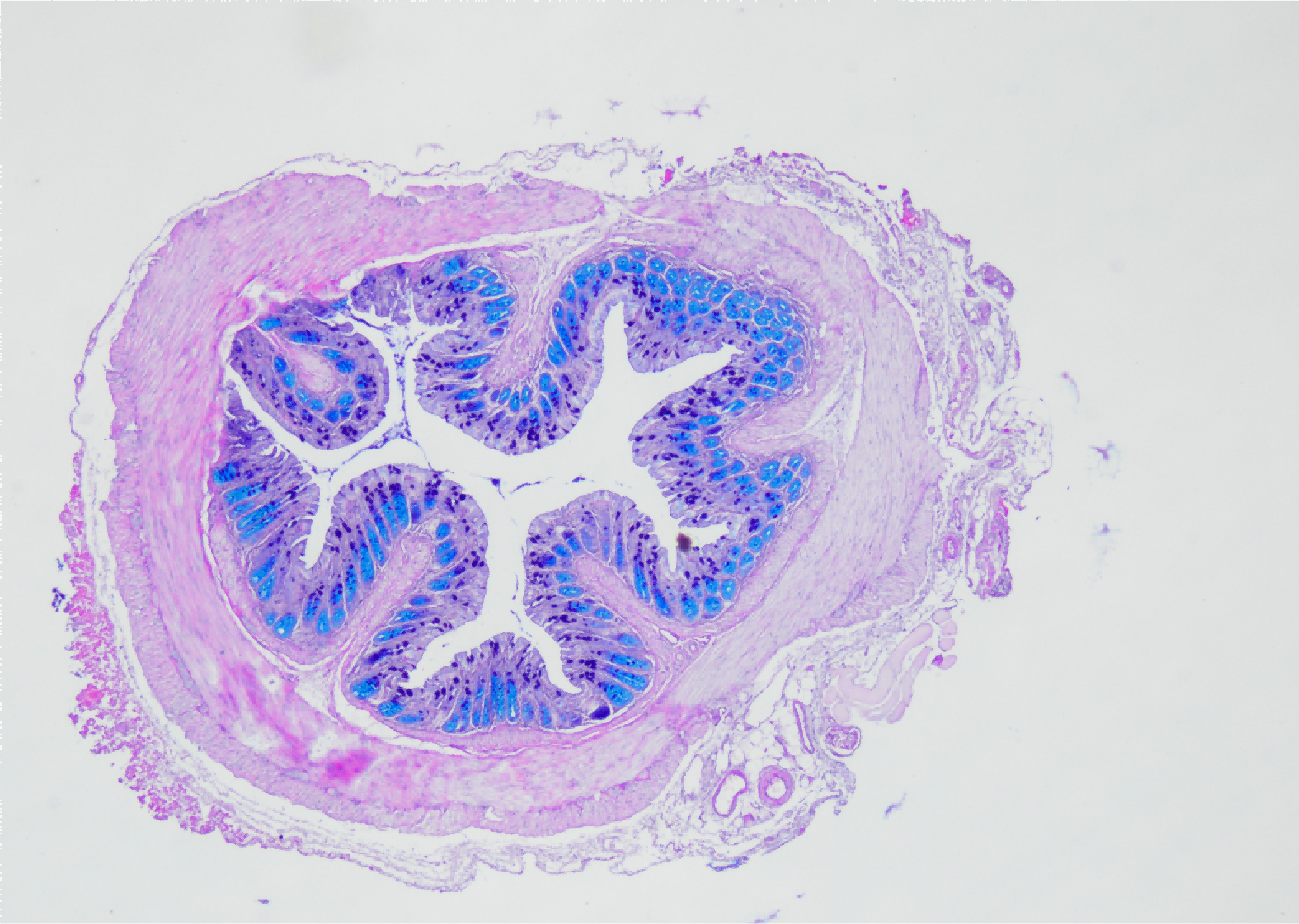

Supplement: Supplementary file 12 — Source data Fig. 7 [file 44319_2024_276_MOESM12_ESM.zip › Fig 7/7H/Yod1++_Water_40×(1).png]

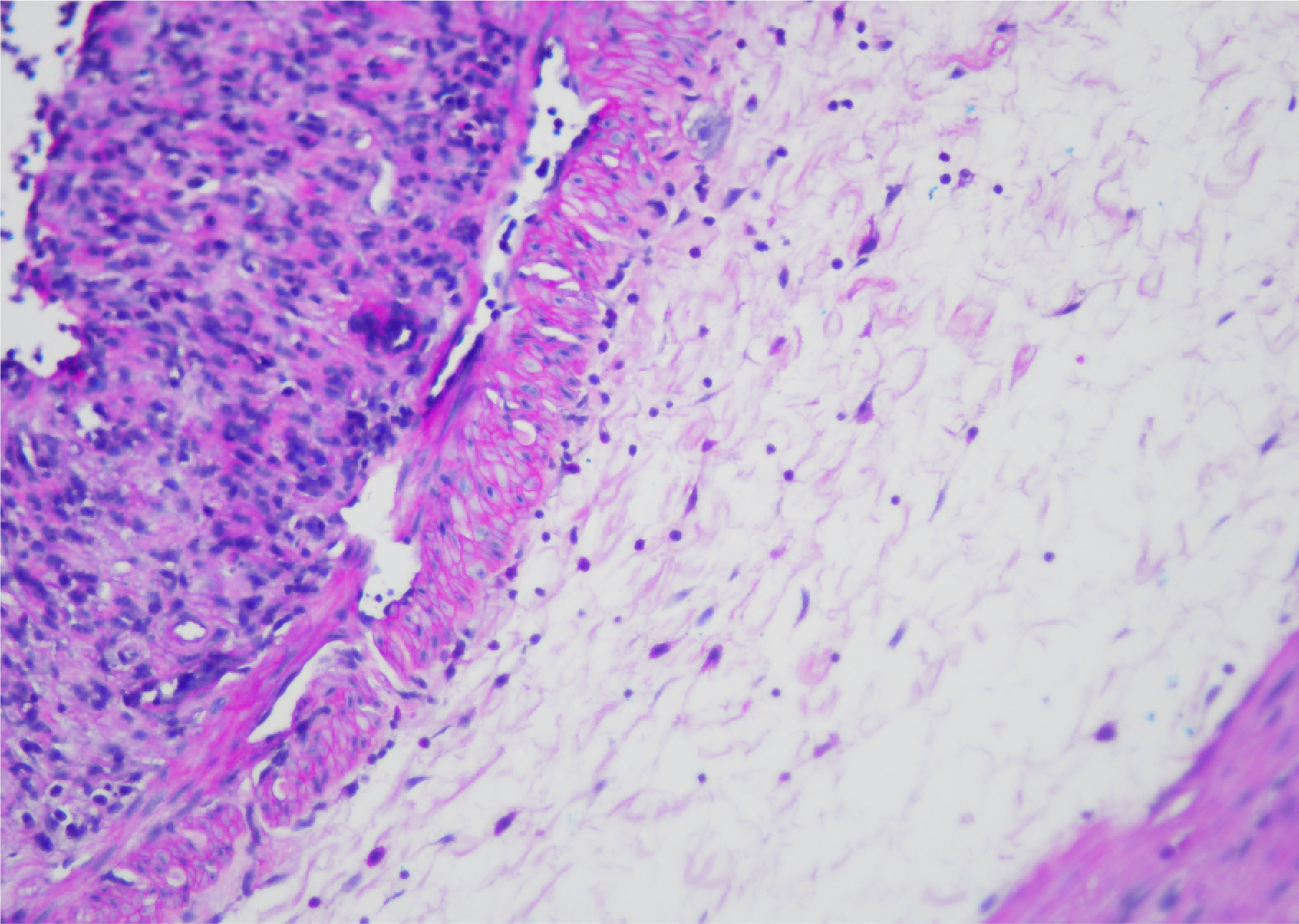

Supplement: Supplementary file 12 — Source data Fig. 7 [file 44319_2024_276_MOESM12_ESM.zip › Fig 7/7H/Yod1--_DSS_MDP_200×.png]

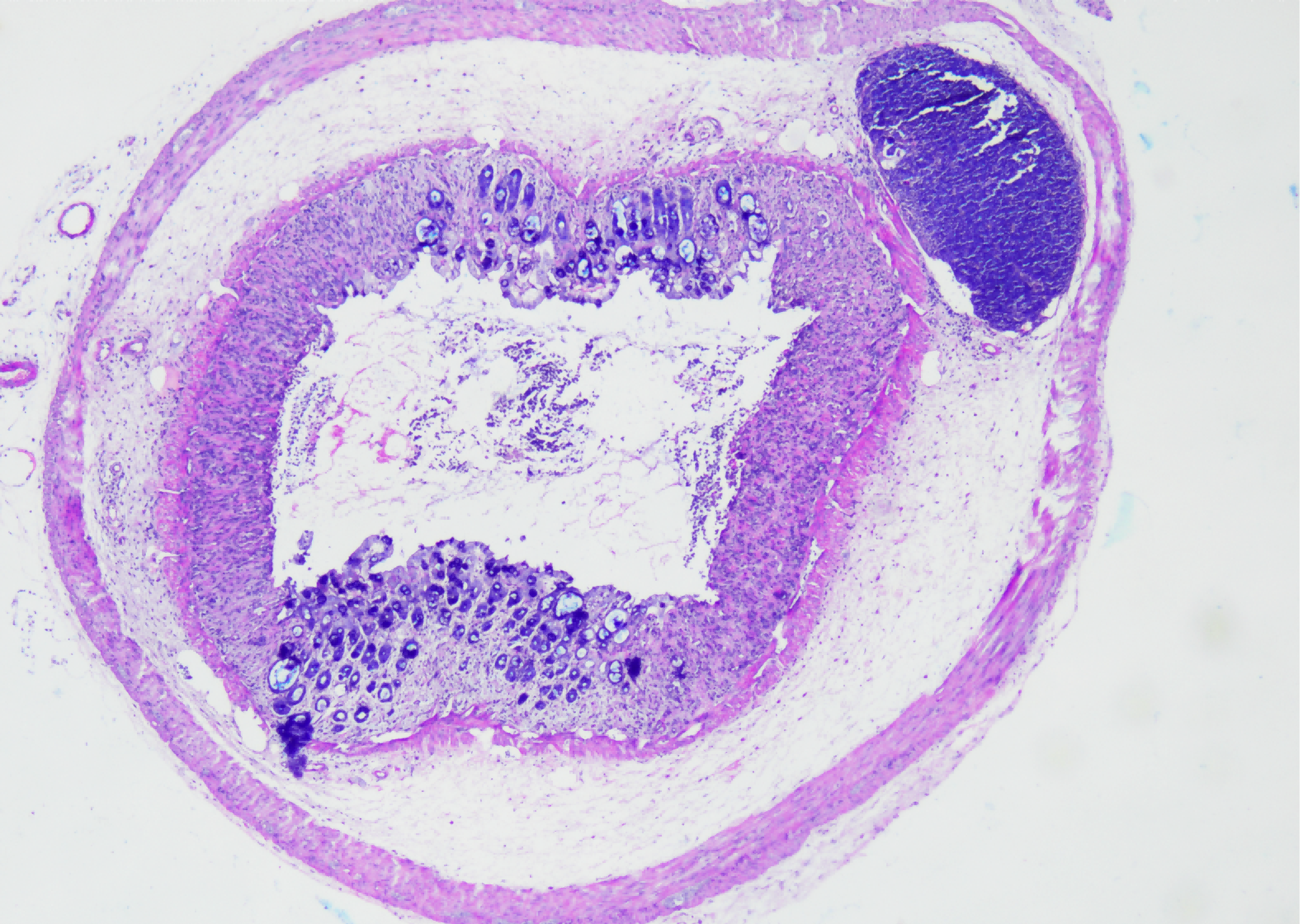

Supplement: Supplementary file 12 — Source data Fig. 7 [file 44319_2024_276_MOESM12_ESM.zip › Fig 7/7H/Yod1--_DSS_MDP_40×.png]

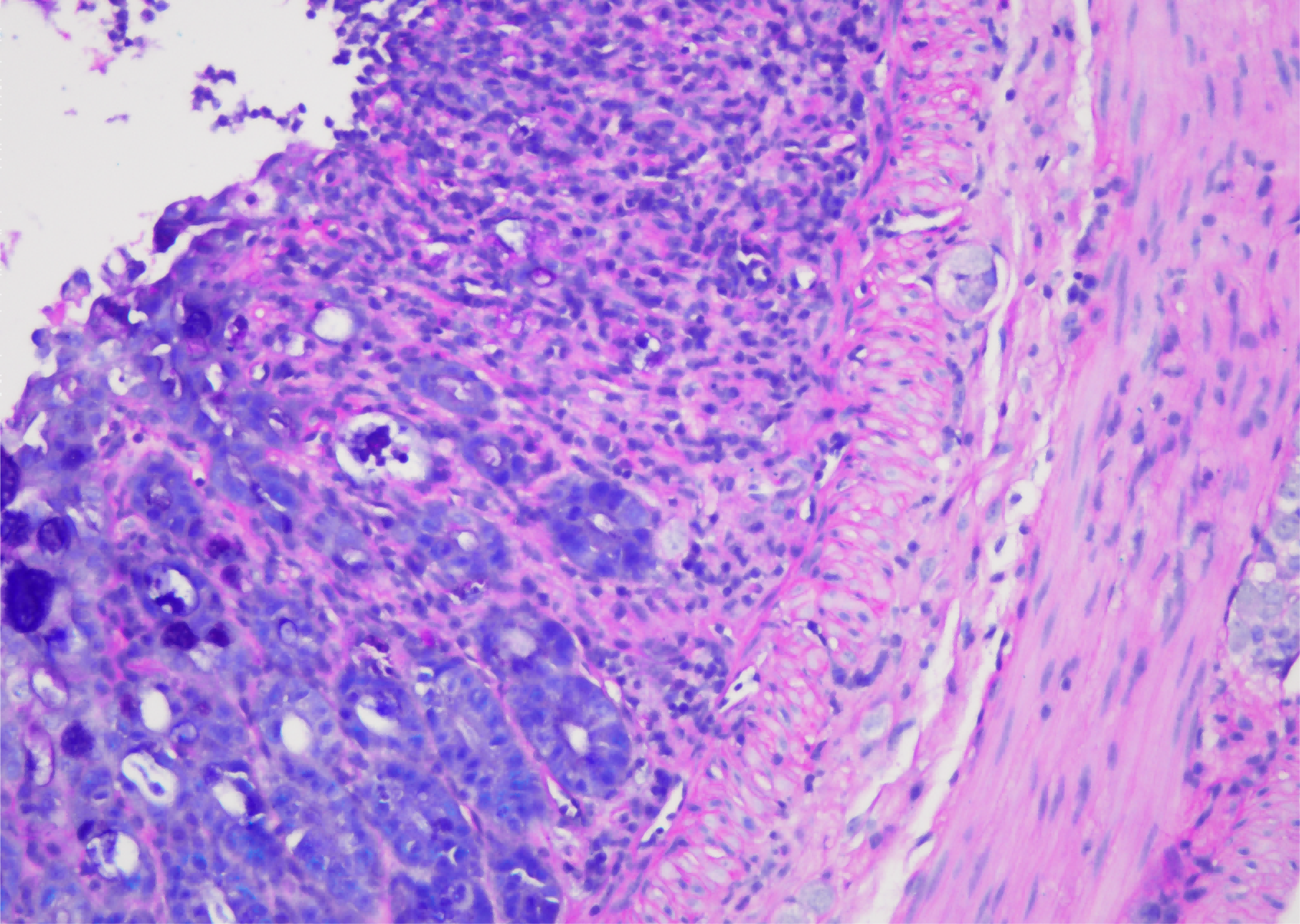

Supplement: Supplementary file 12 — Source data Fig. 7 [file 44319_2024_276_MOESM12_ESM.zip › Fig 7/7H/Yod1--_DSS_PBS_200×.png]

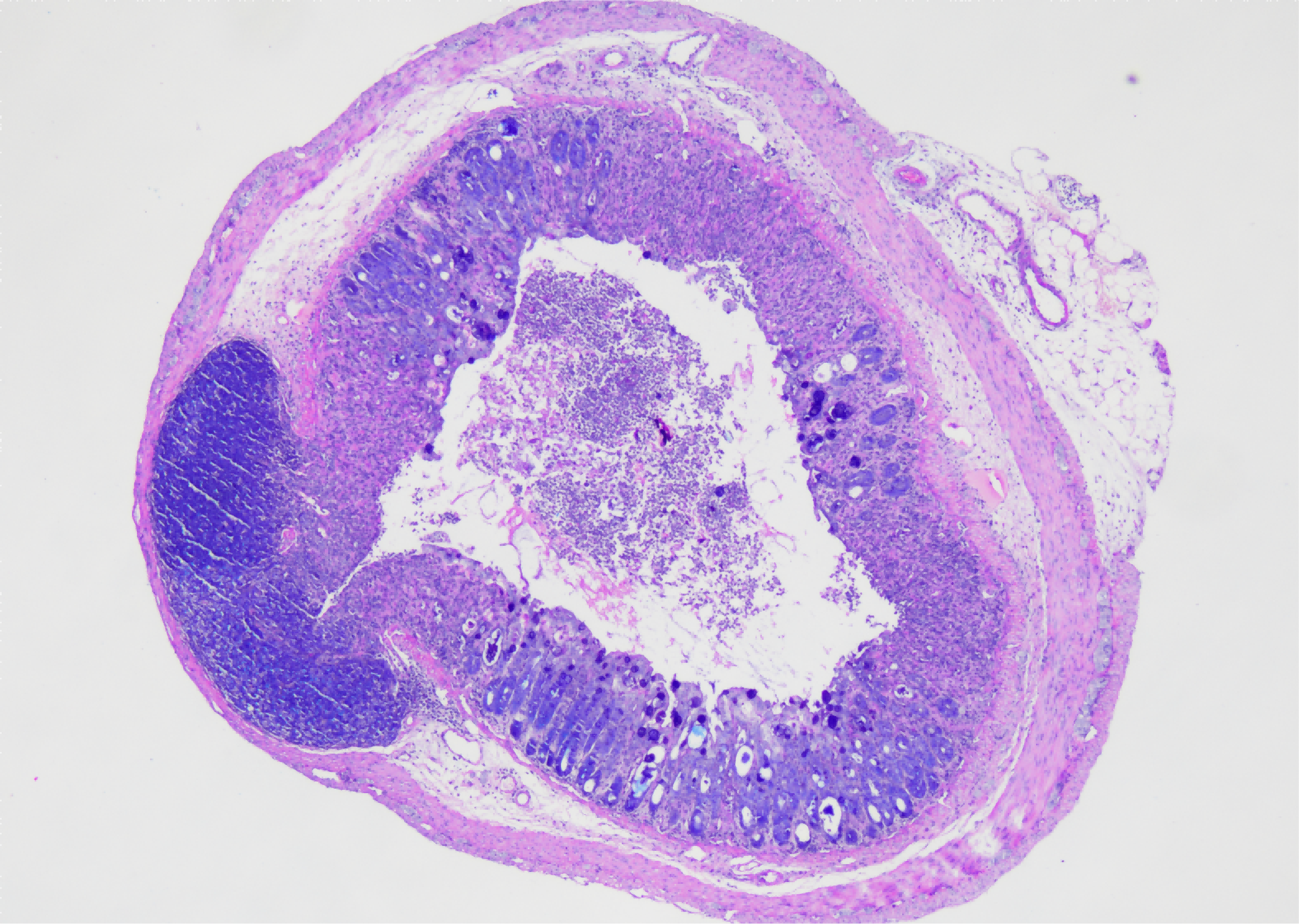

Supplement: Supplementary file 12 — Source data Fig. 7 [file 44319_2024_276_MOESM12_ESM.zip › Fig 7/7H/Yod1--_DSS_PBS_40×.png]

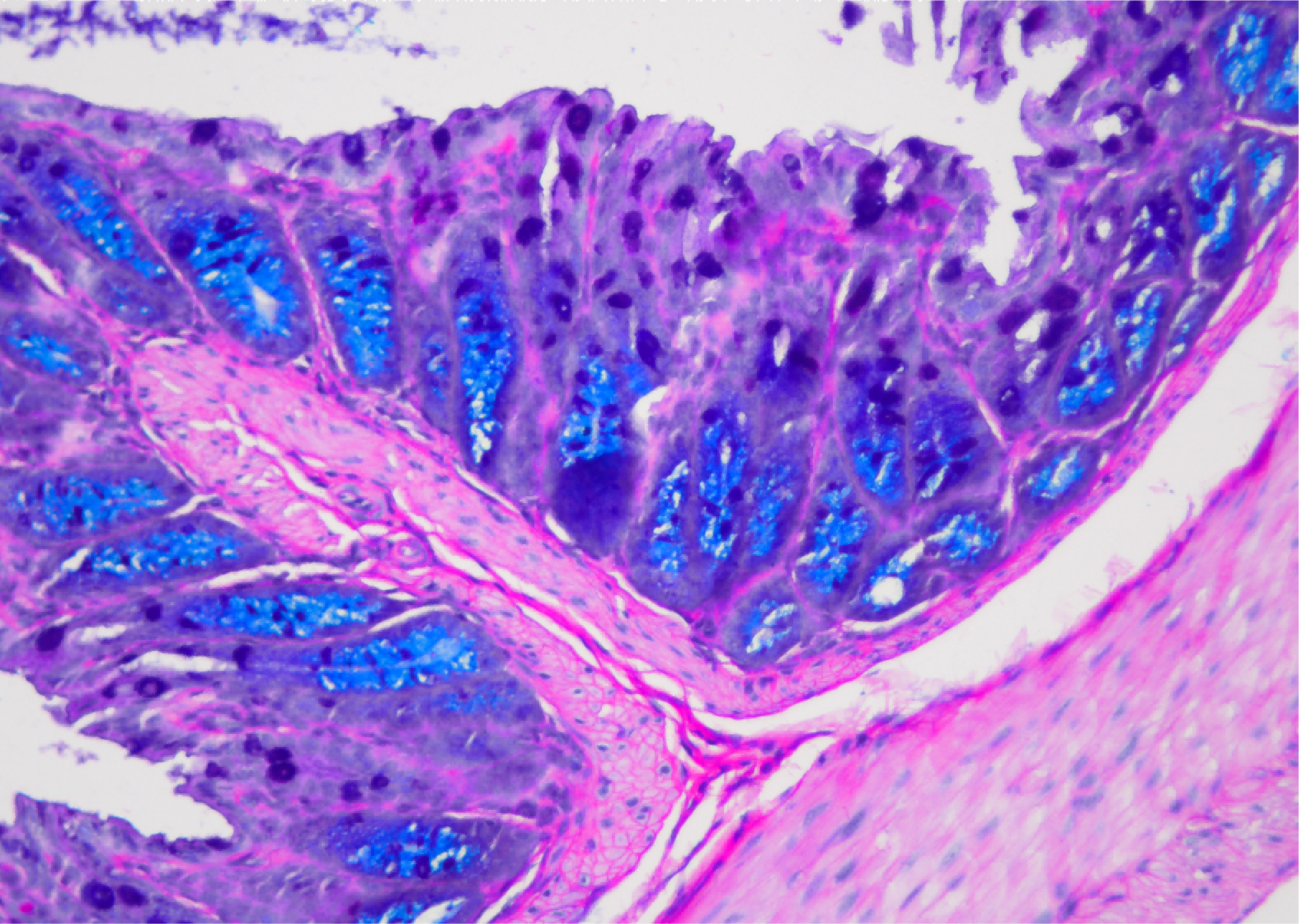

Supplement: Supplementary file 12 — Source data Fig. 7 [file 44319_2024_276_MOESM12_ESM.zip › Fig 7/7H/Yod1--_Water_200×(1).png]

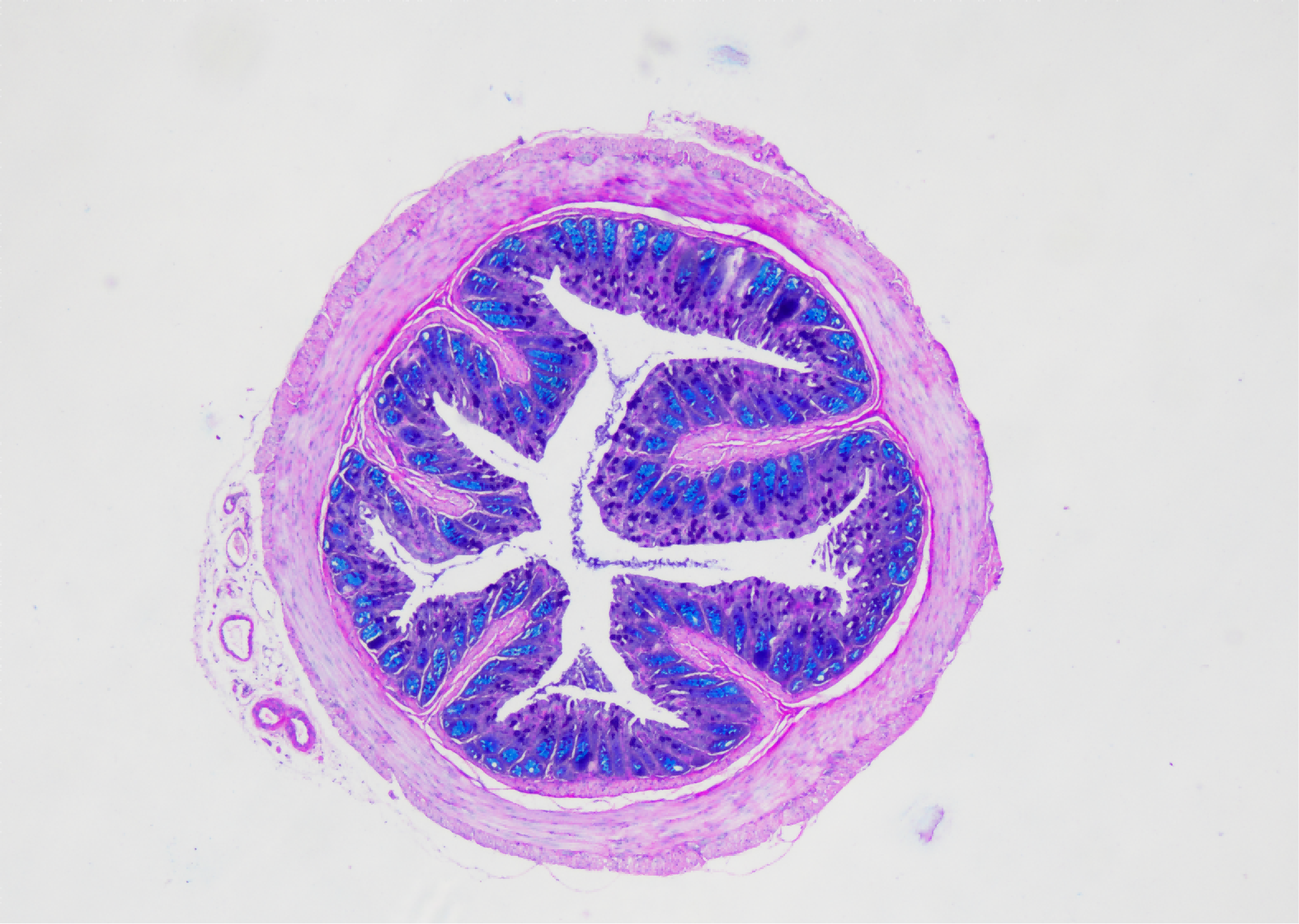

Supplement: Supplementary file 12 — Source data Fig. 7 [file 44319_2024_276_MOESM12_ESM.zip › Fig 7/7H/Yod1--_Water_40×(1).png]

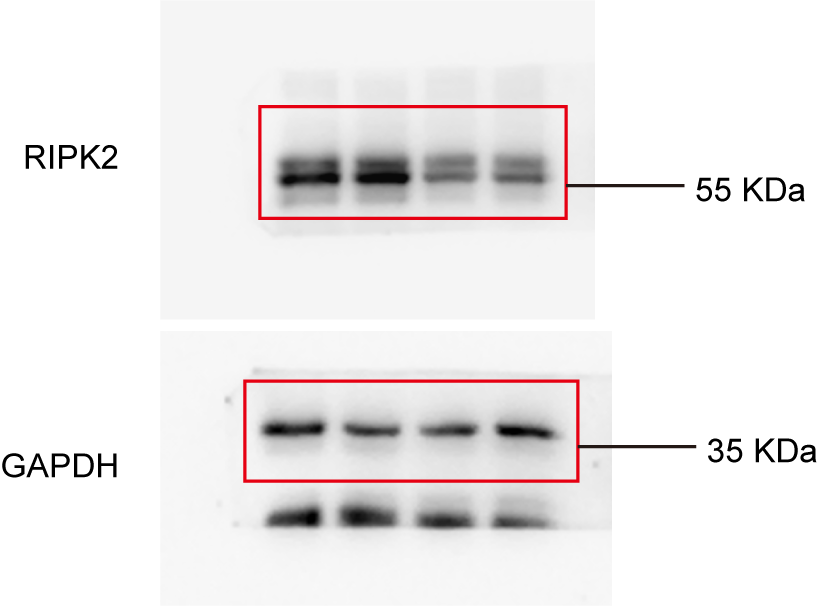

Supplement: Supplementary file 13 — Source data Fig. 8 [file 44319_2024_276_MOESM13_ESM.zip › Fig 8/8B/8B.tif]

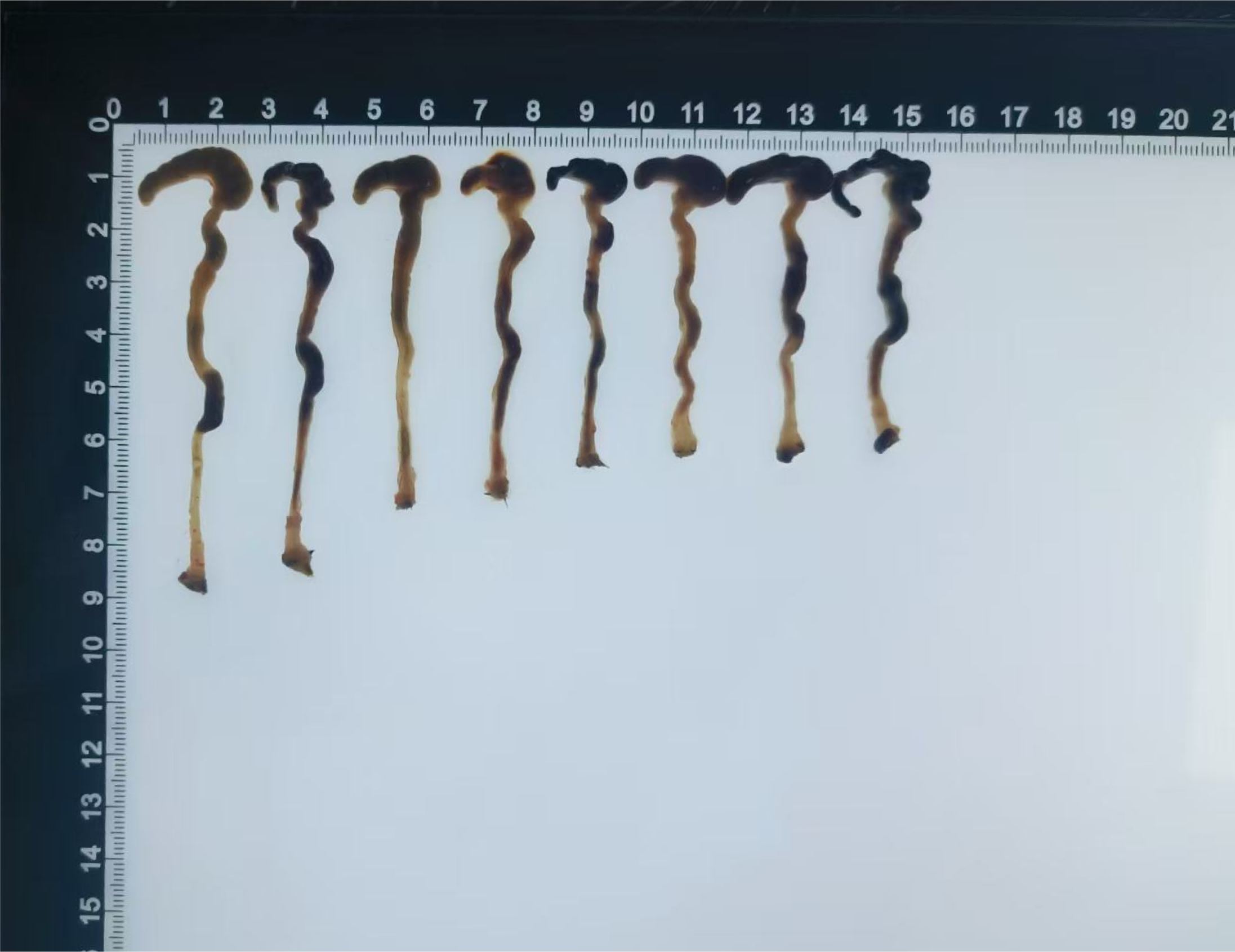

Supplement: Supplementary file 13 — Source data Fig. 8 [file 44319_2024_276_MOESM13_ESM.zip › Fig 8/8F/8F.png]

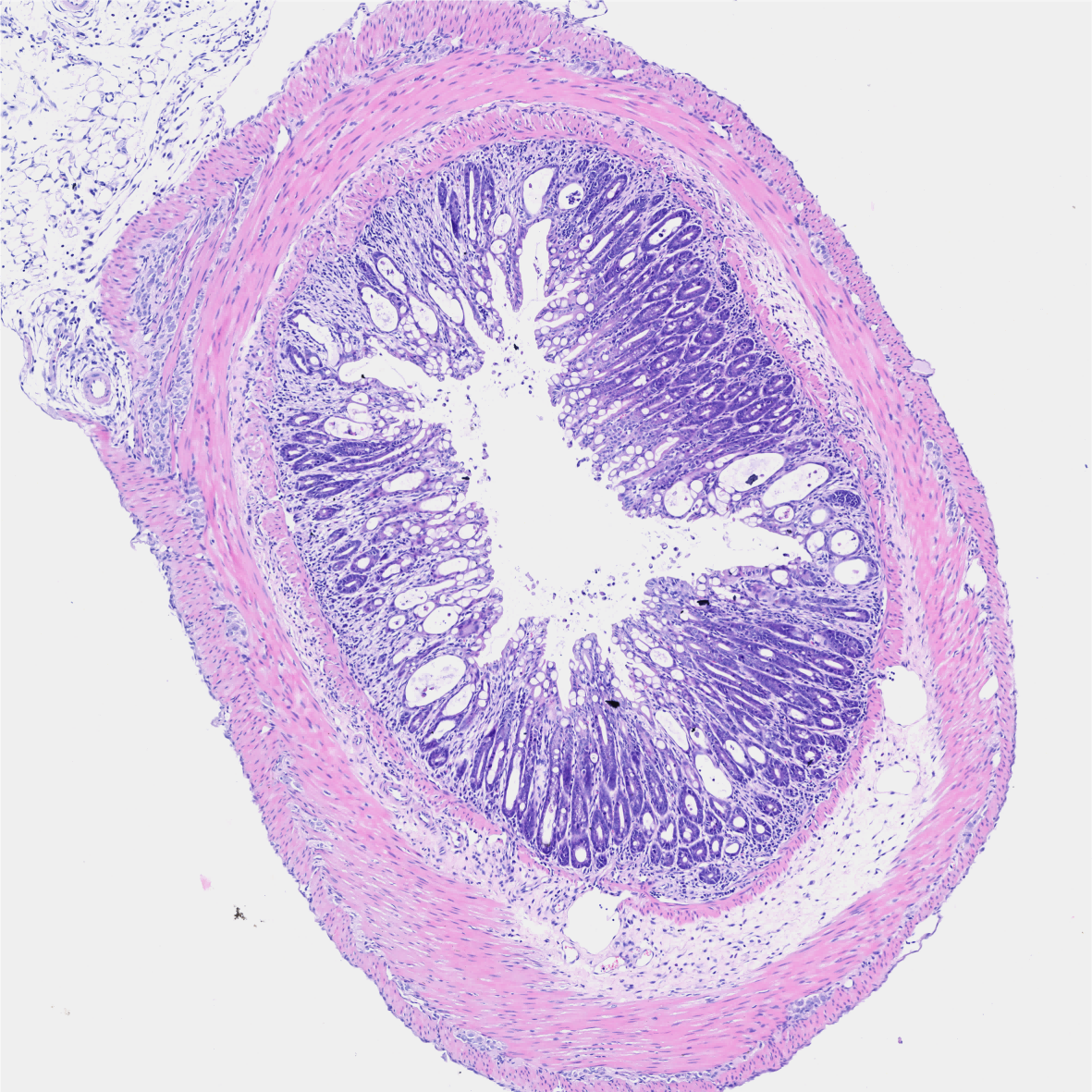

Supplement: Supplementary file 13 — Source data Fig. 8 [file 44319_2024_276_MOESM13_ESM.zip › Fig 8/8J/HE staining/AAV9-CON/Yod1++_overall image.png]

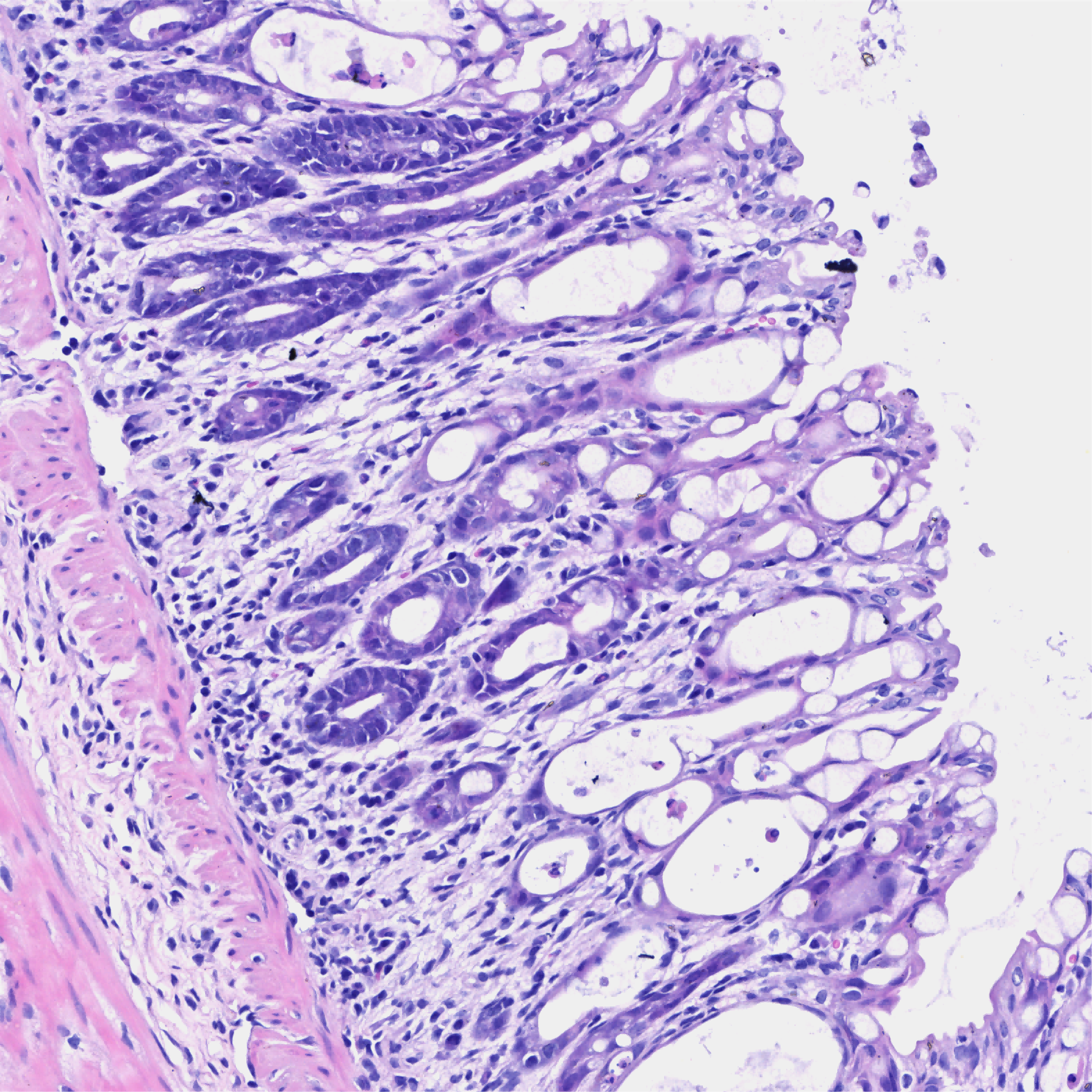

Supplement: Supplementary file 13 — Source data Fig. 8 [file 44319_2024_276_MOESM13_ESM.zip › Fig 8/8J/HE staining/AAV9-CON/Yod1++_partial image.png]

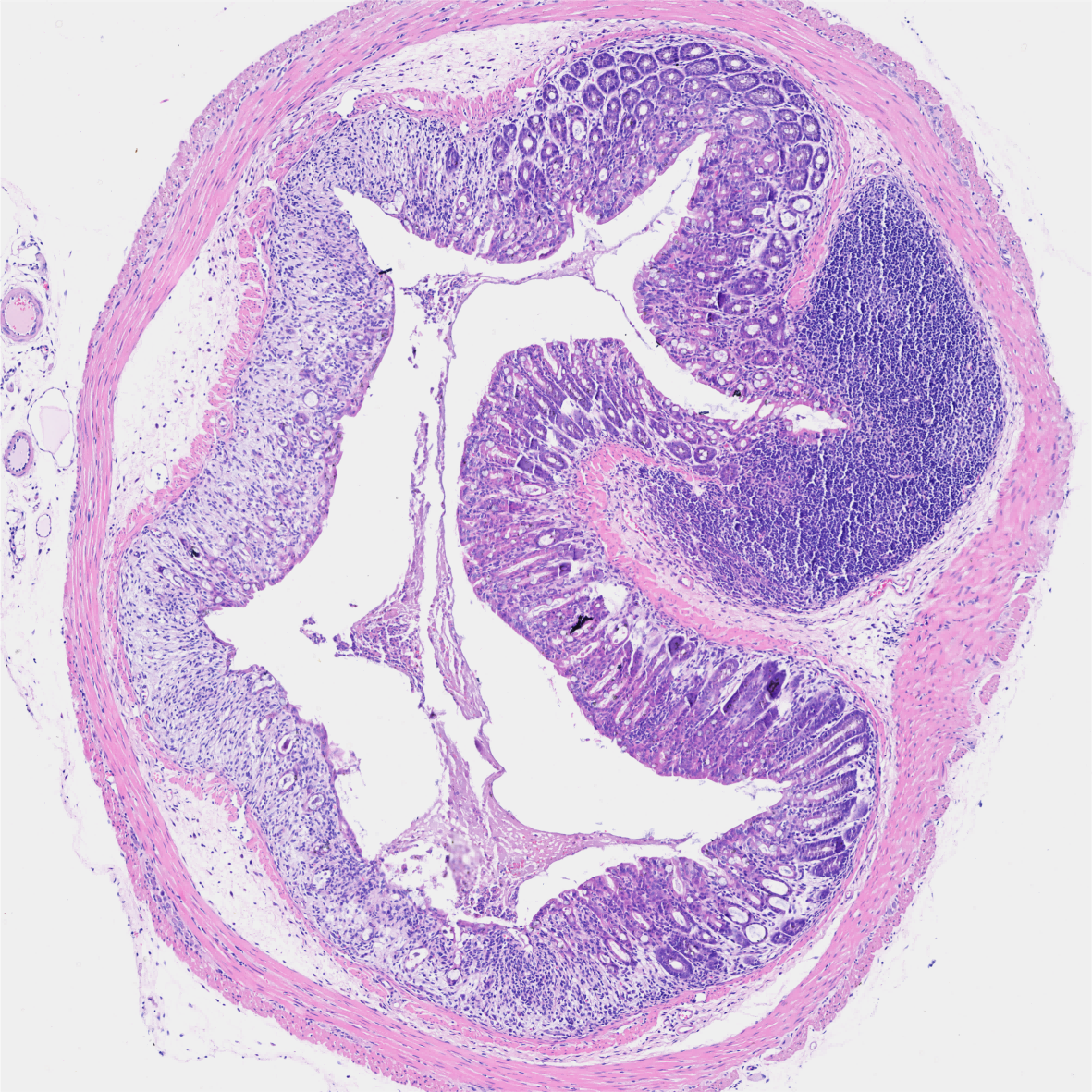

Supplement: Supplementary file 13 — Source data Fig. 8 [file 44319_2024_276_MOESM13_ESM.zip › Fig 8/8J/HE staining/AAV9-CON/Yod1--_overall image.png]

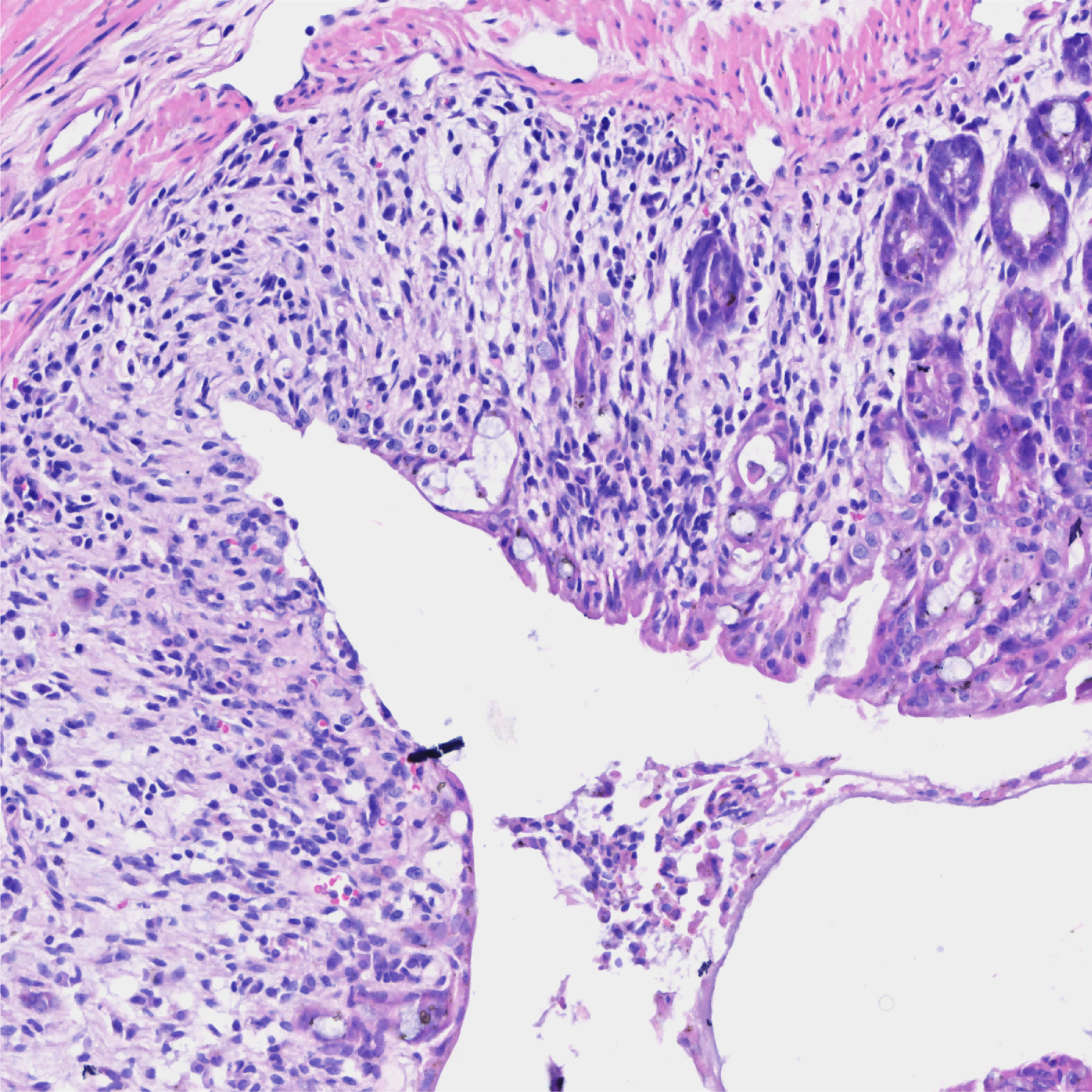

Supplement: Supplementary file 13 — Source data Fig. 8 [file 44319_2024_276_MOESM13_ESM.zip › Fig 8/8J/HE staining/AAV9-CON/Yod1--partial image.png]

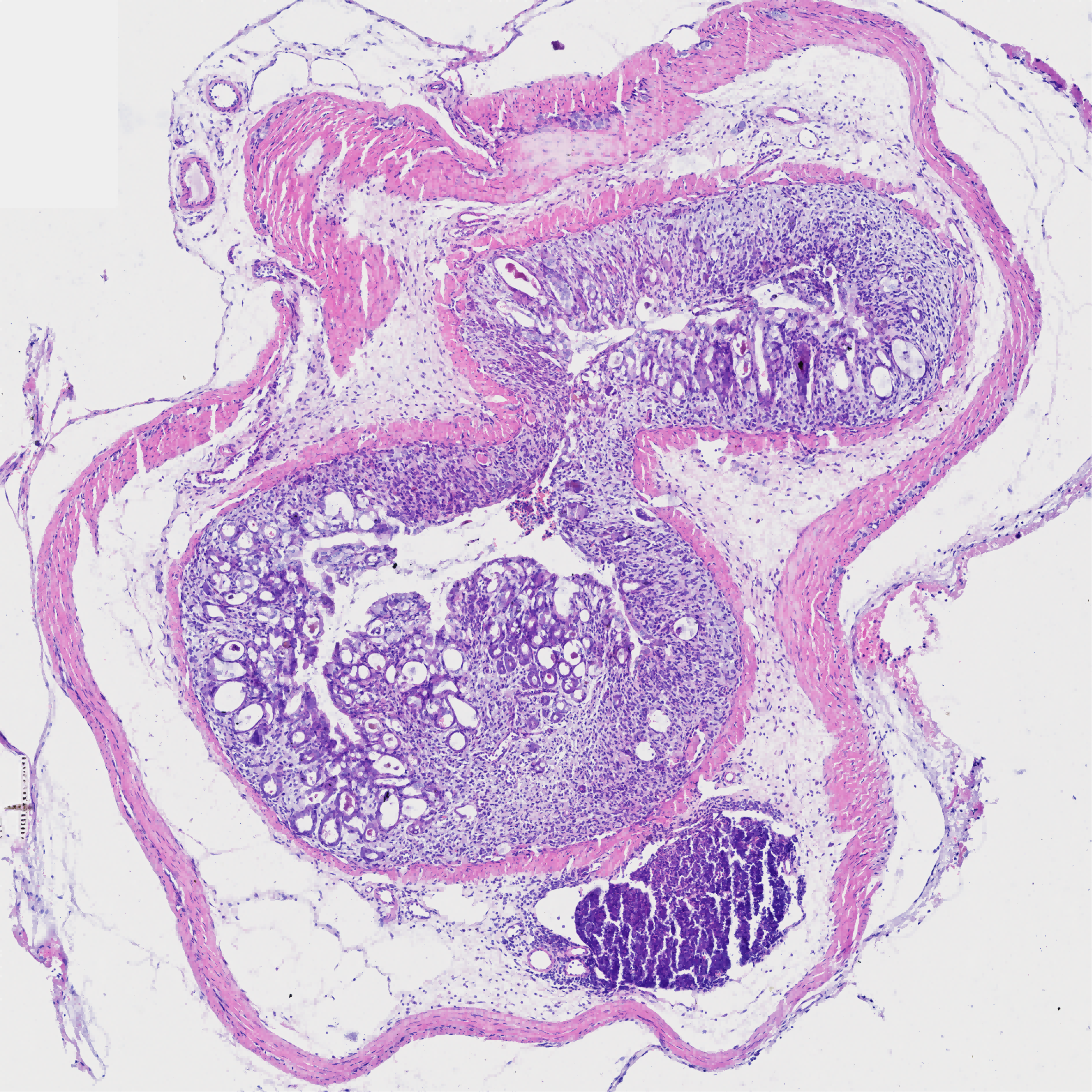

Supplement: Supplementary file 13 — Source data Fig. 8 [file 44319_2024_276_MOESM13_ESM.zip › Fig 8/8J/HE staining/AAV9-Ripk2-shRNA/Yod1++_overall image(1).png]

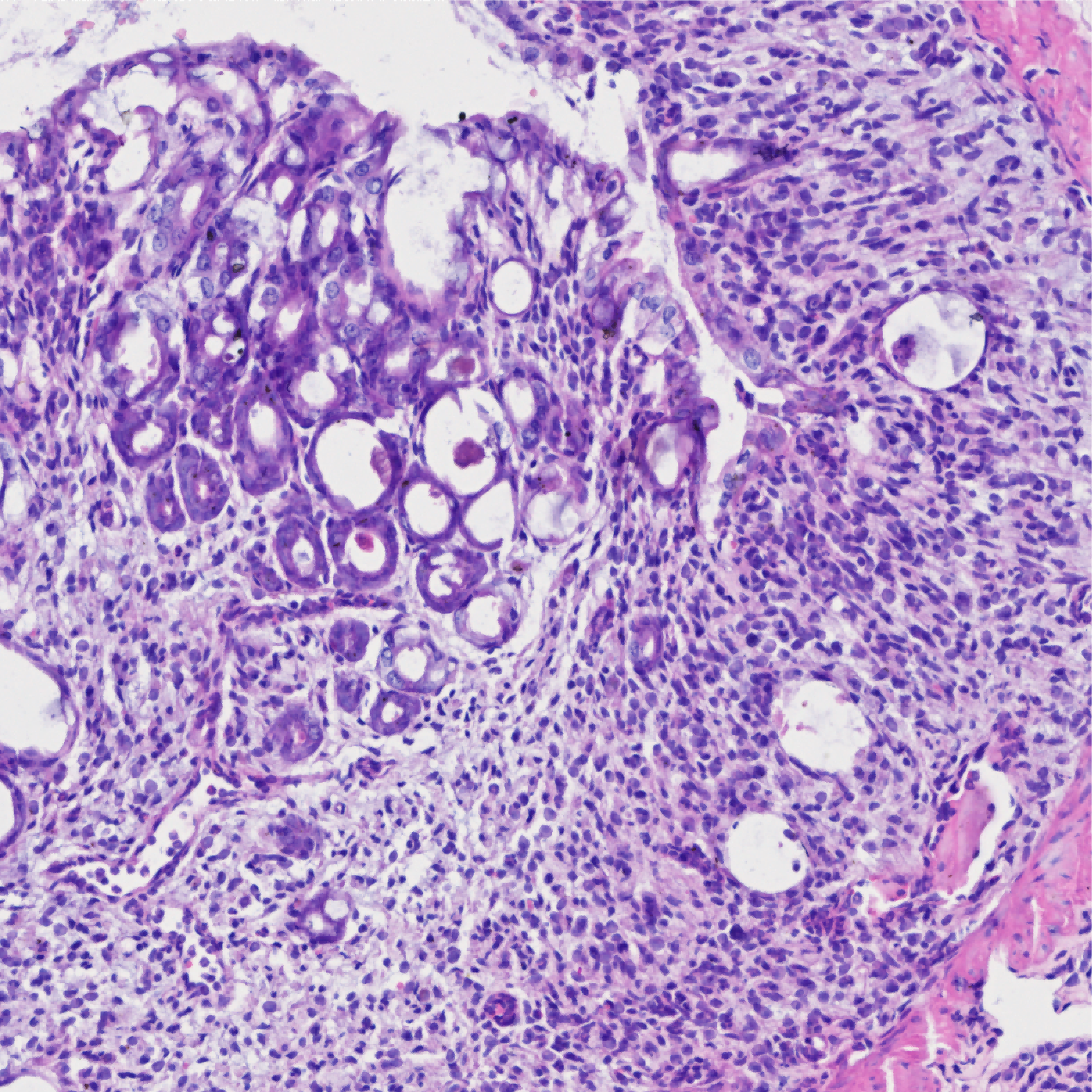

Supplement: Supplementary file 13 — Source data Fig. 8 [file 44319_2024_276_MOESM13_ESM.zip › Fig 8/8J/HE staining/AAV9-Ripk2-shRNA/Yod1++_partial image(1).png]

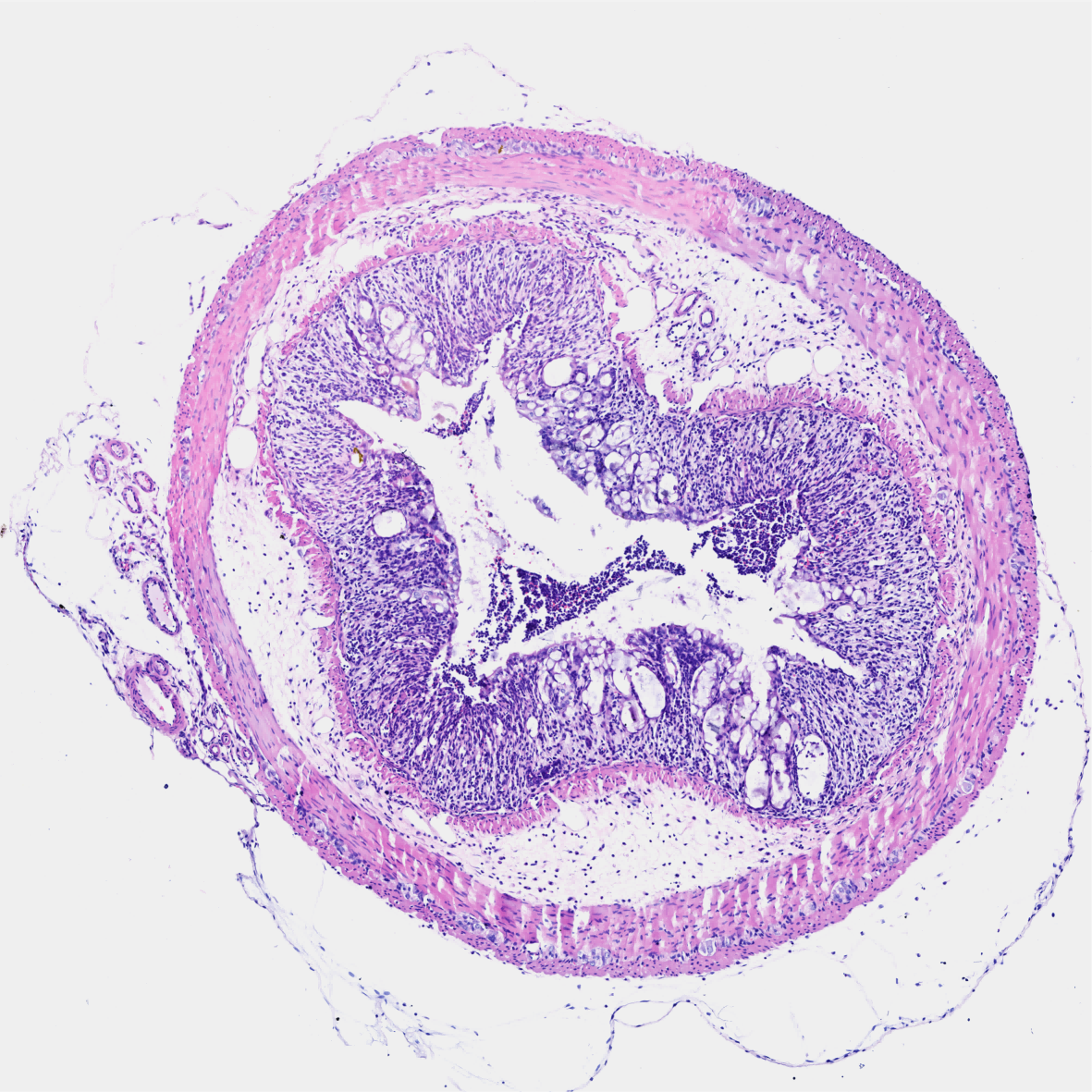

Supplement: Supplementary file 13 — Source data Fig. 8 [file 44319_2024_276_MOESM13_ESM.zip › Fig 8/8J/HE staining/AAV9-Ripk2-shRNA/Yod1--_overall image(1).png]

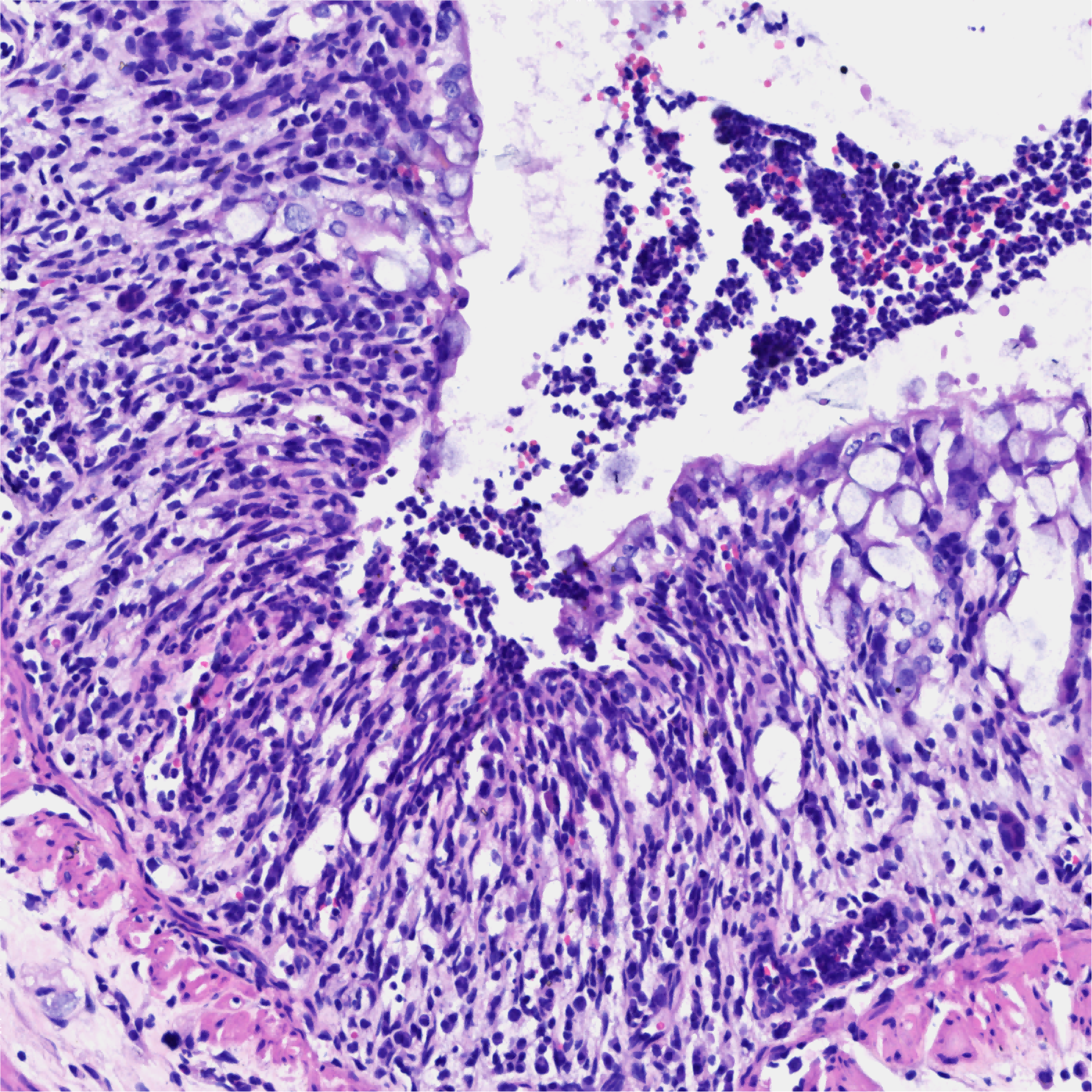

Supplement: Supplementary file 13 — Source data Fig. 8 [file 44319_2024_276_MOESM13_ESM.zip › Fig 8/8J/HE staining/AAV9-Ripk2-shRNA/Yod1--_partial image.png]

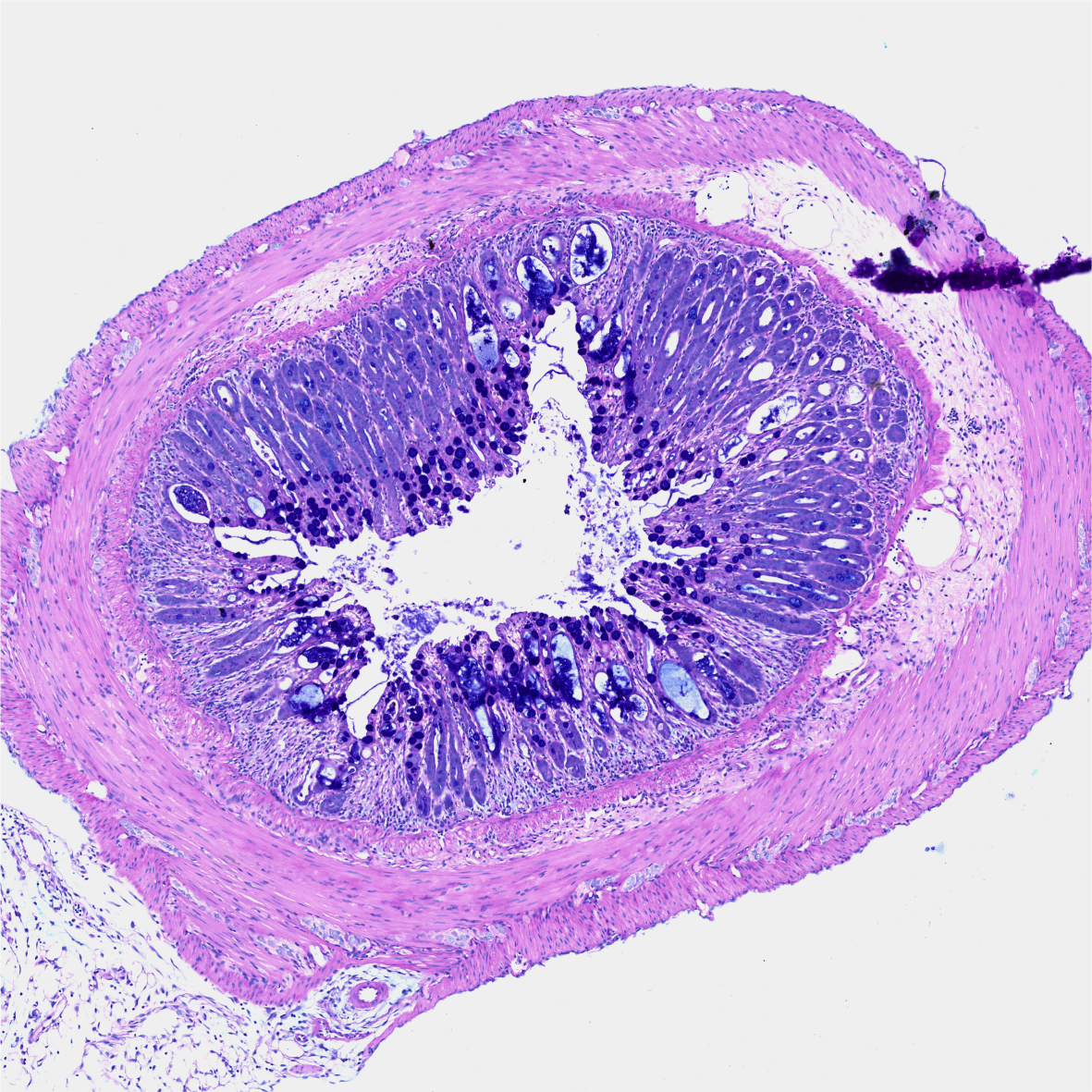

Supplement: Supplementary file 13 — Source data Fig. 8 [file 44319_2024_276_MOESM13_ESM.zip › Fig 8/8J/PAS_AB staining/AAV9-CON/Yod1++_overall image(2).png]

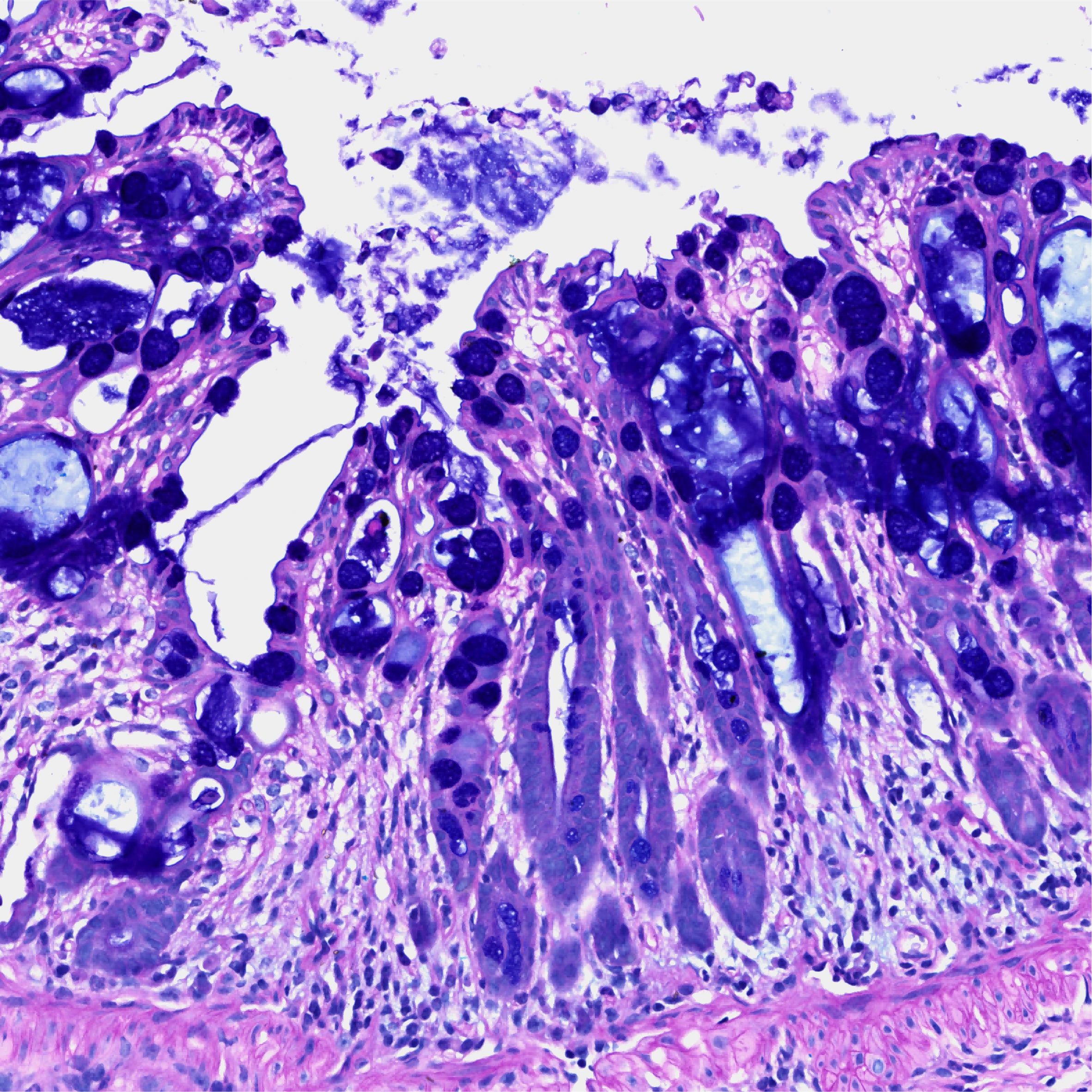

Supplement: Supplementary file 13 — Source data Fig. 8 [file 44319_2024_276_MOESM13_ESM.zip › Fig 8/8J/PAS_AB staining/AAV9-CON/Yod1++_partial image(2).png]

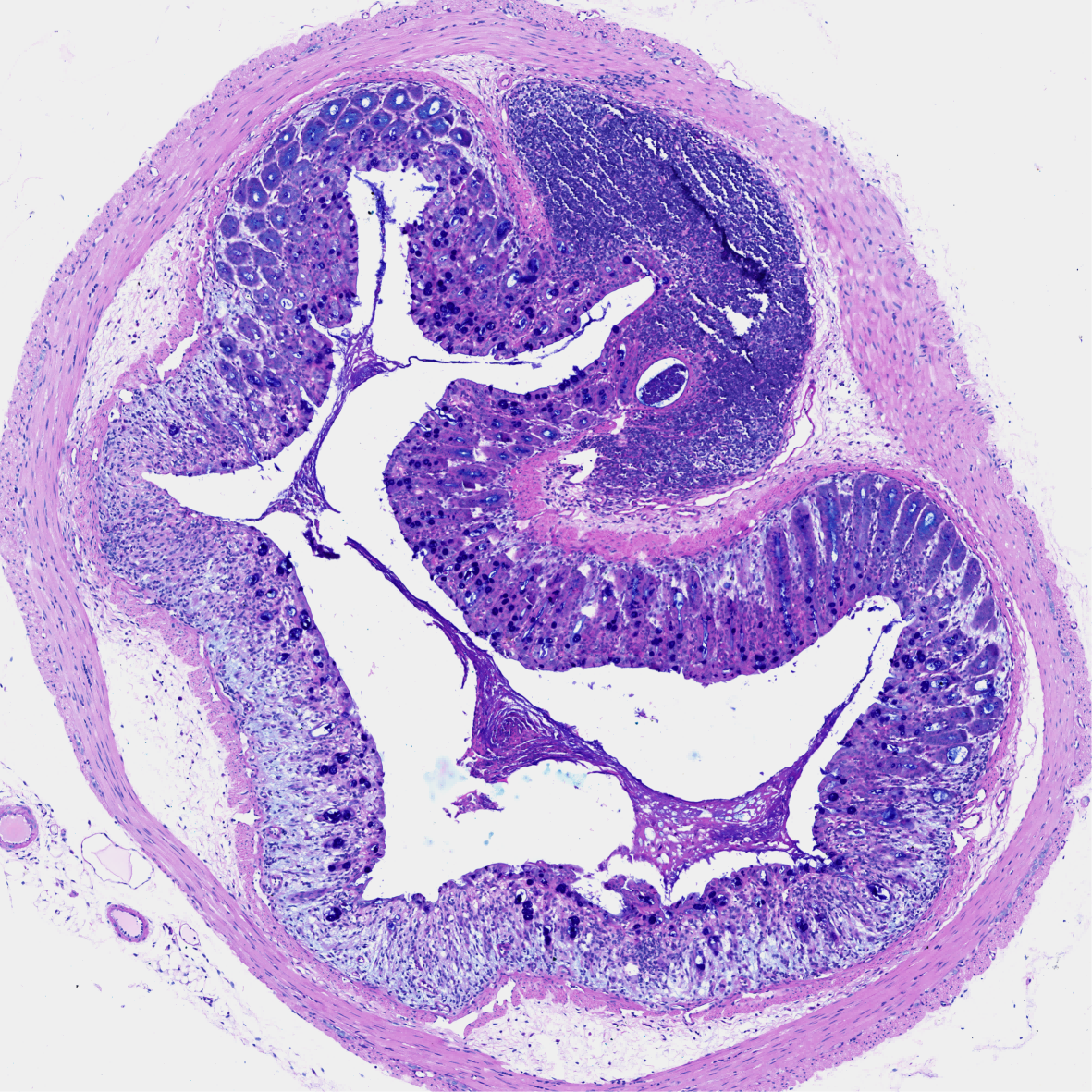

Supplement: Supplementary file 13 — Source data Fig. 8 [file 44319_2024_276_MOESM13_ESM.zip › Fig 8/8J/PAS_AB staining/AAV9-CON/Yod1--_overall image(2).png]

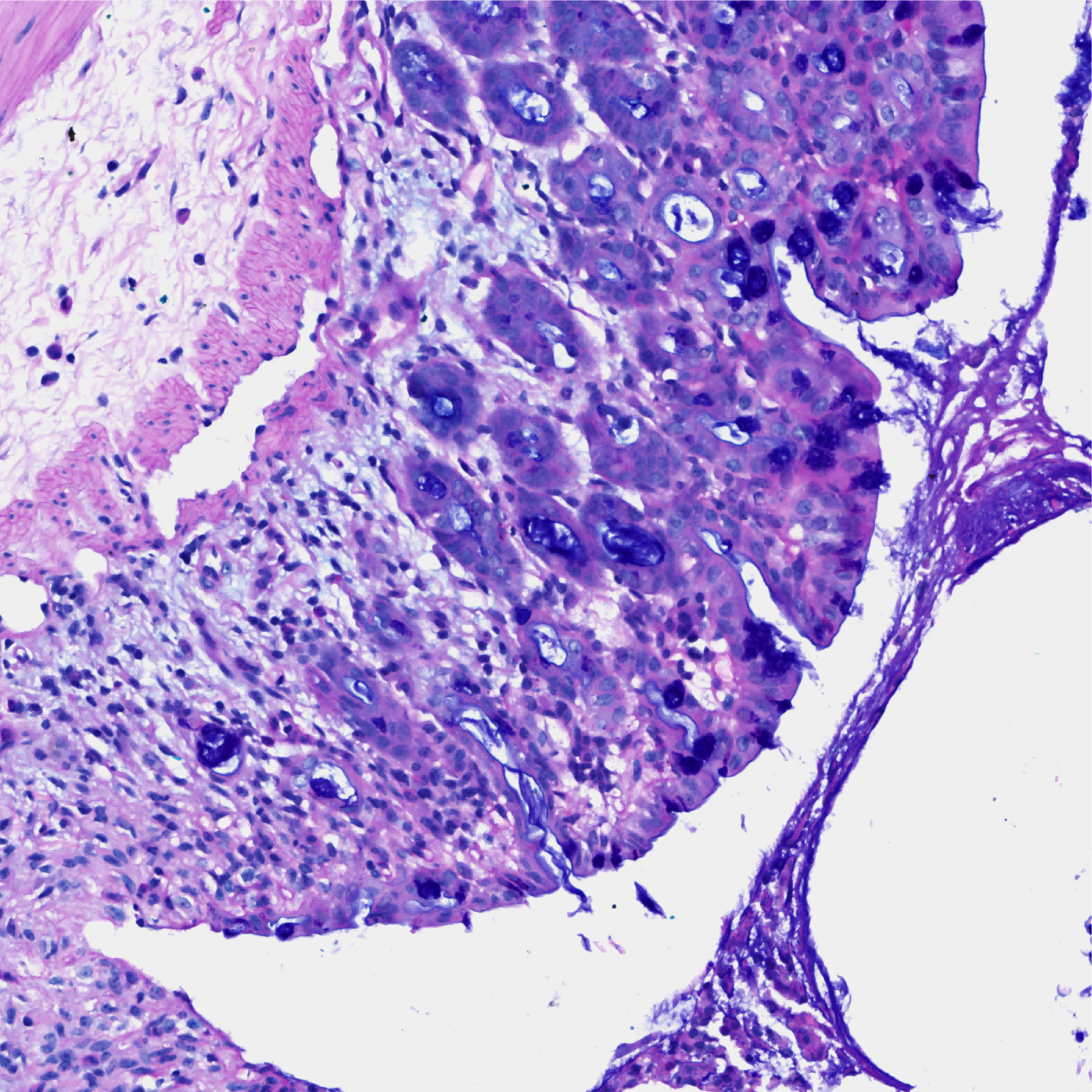

Supplement: Supplementary file 13 — Source data Fig. 8 [file 44319_2024_276_MOESM13_ESM.zip › Fig 8/8J/PAS_AB staining/AAV9-CON/Yod1--partial image(1).png]

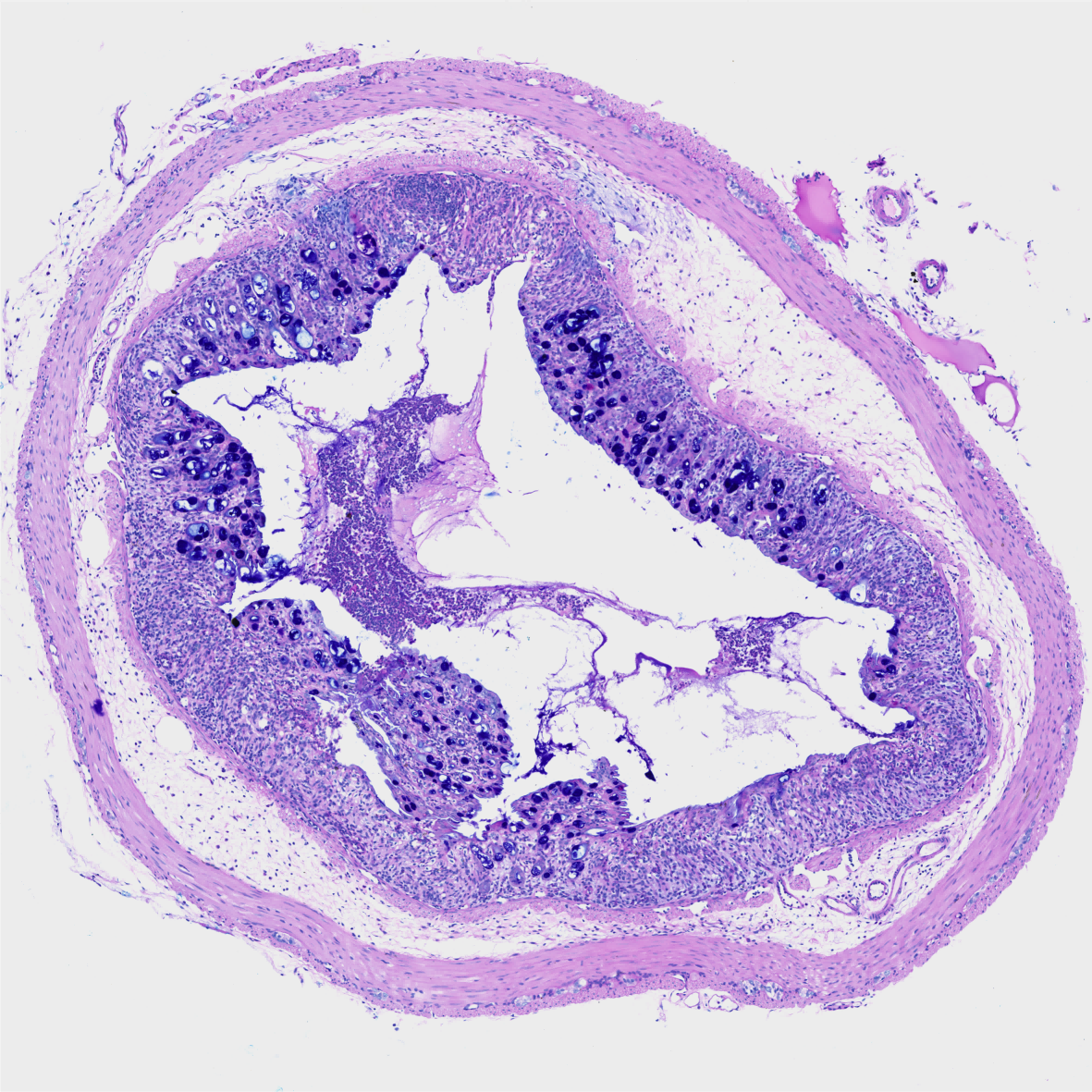

Supplement: Supplementary file 13 — Source data Fig. 8 [file 44319_2024_276_MOESM13_ESM.zip › Fig 8/8J/PAS_AB staining/AAV9-Ripk2-shRNA/Yod1++_overall image(3).png]

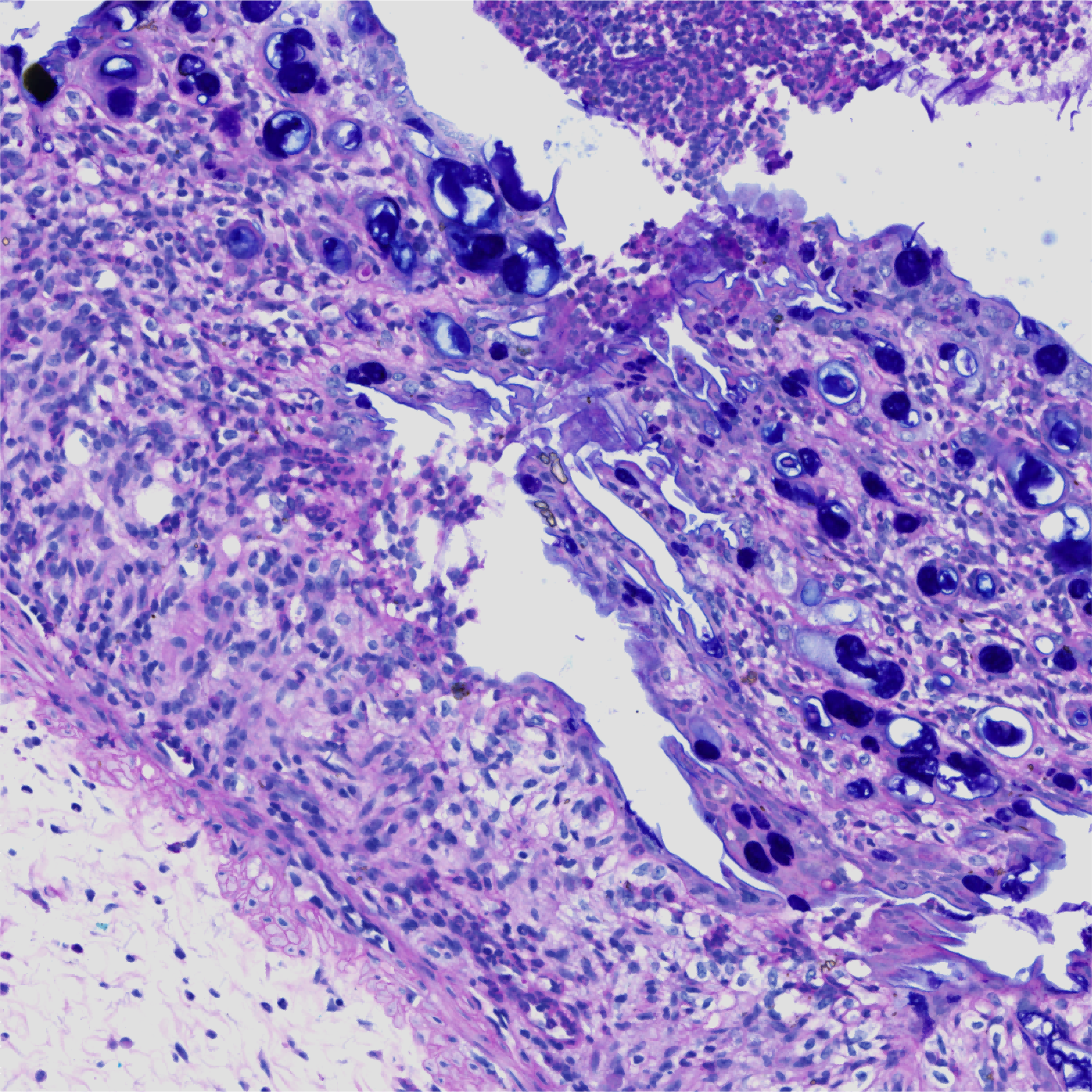

Supplement: Supplementary file 13 — Source data Fig. 8 [file 44319_2024_276_MOESM13_ESM.zip › Fig 8/8J/PAS_AB staining/AAV9-Ripk2-shRNA/Yod1++_partial image(3).png]

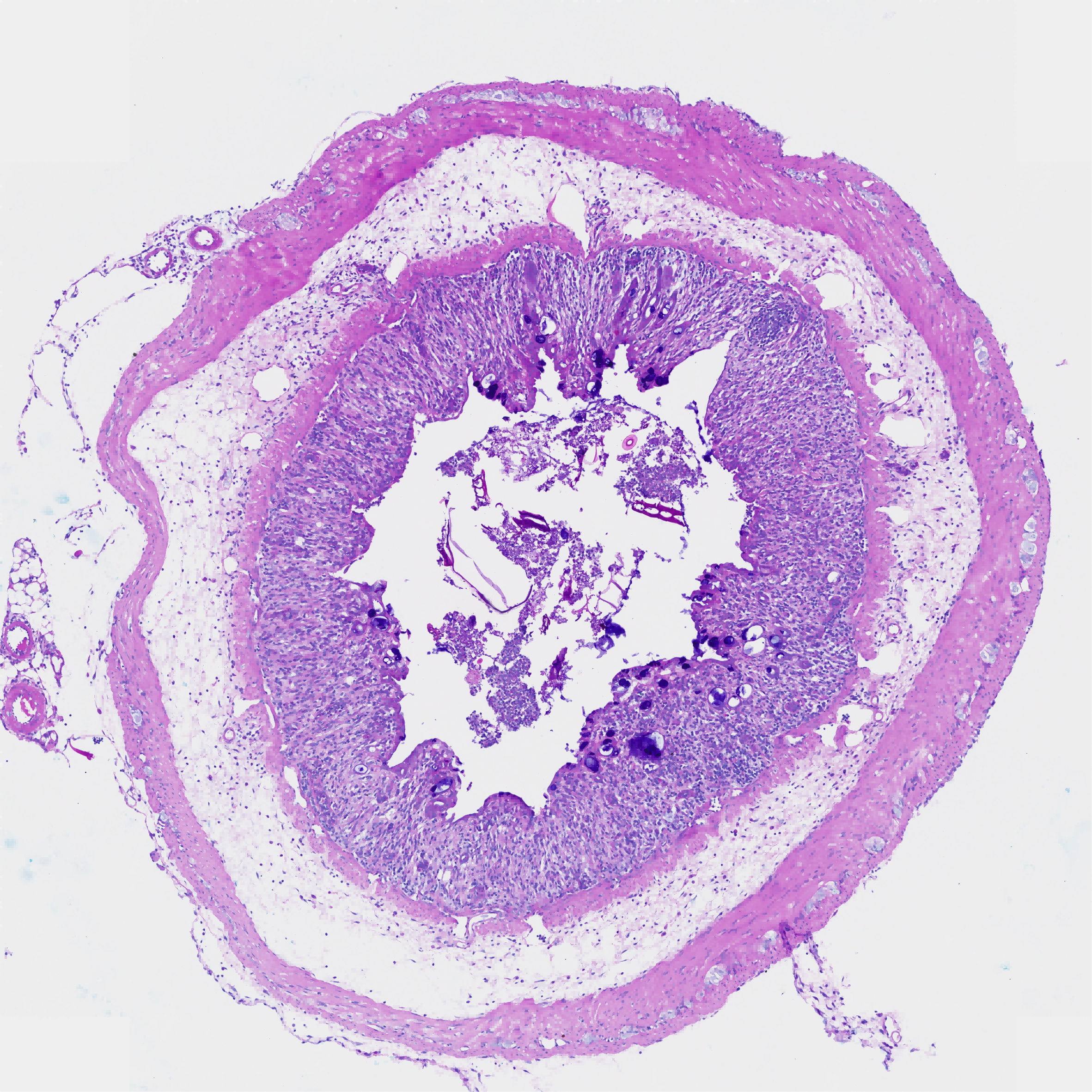

Supplement: Supplementary file 13 — Source data Fig. 8 [file 44319_2024_276_MOESM13_ESM.zip › Fig 8/8J/PAS_AB staining/AAV9-Ripk2-shRNA/Yod1--_overall image(3).png]

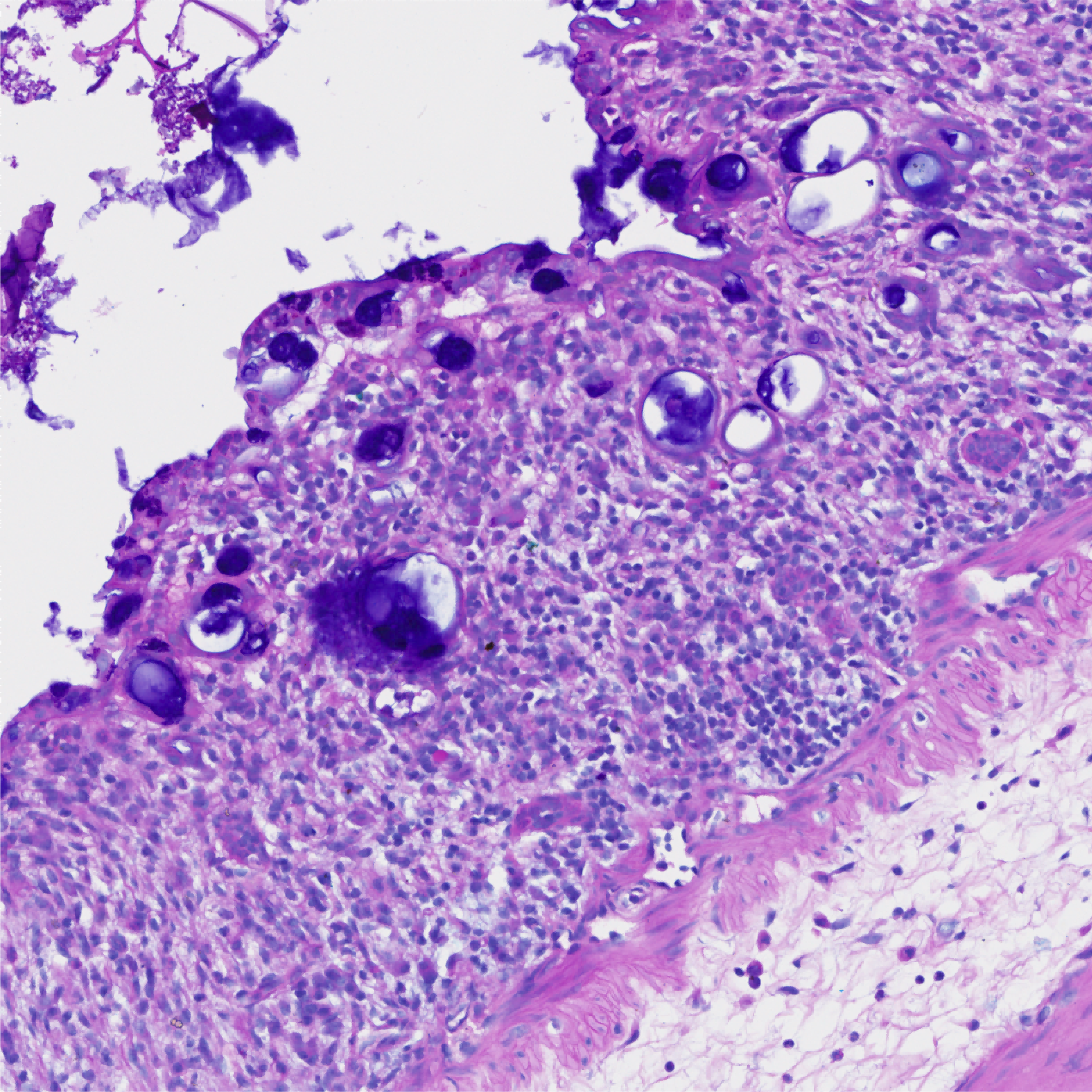

Supplement: Supplementary file 13 — Source data Fig. 8 [file 44319_2024_276_MOESM13_ESM.zip › Fig 8/8J/PAS_AB staining/AAV9-Ripk2-shRNA/Yod1--partial image(2).png]

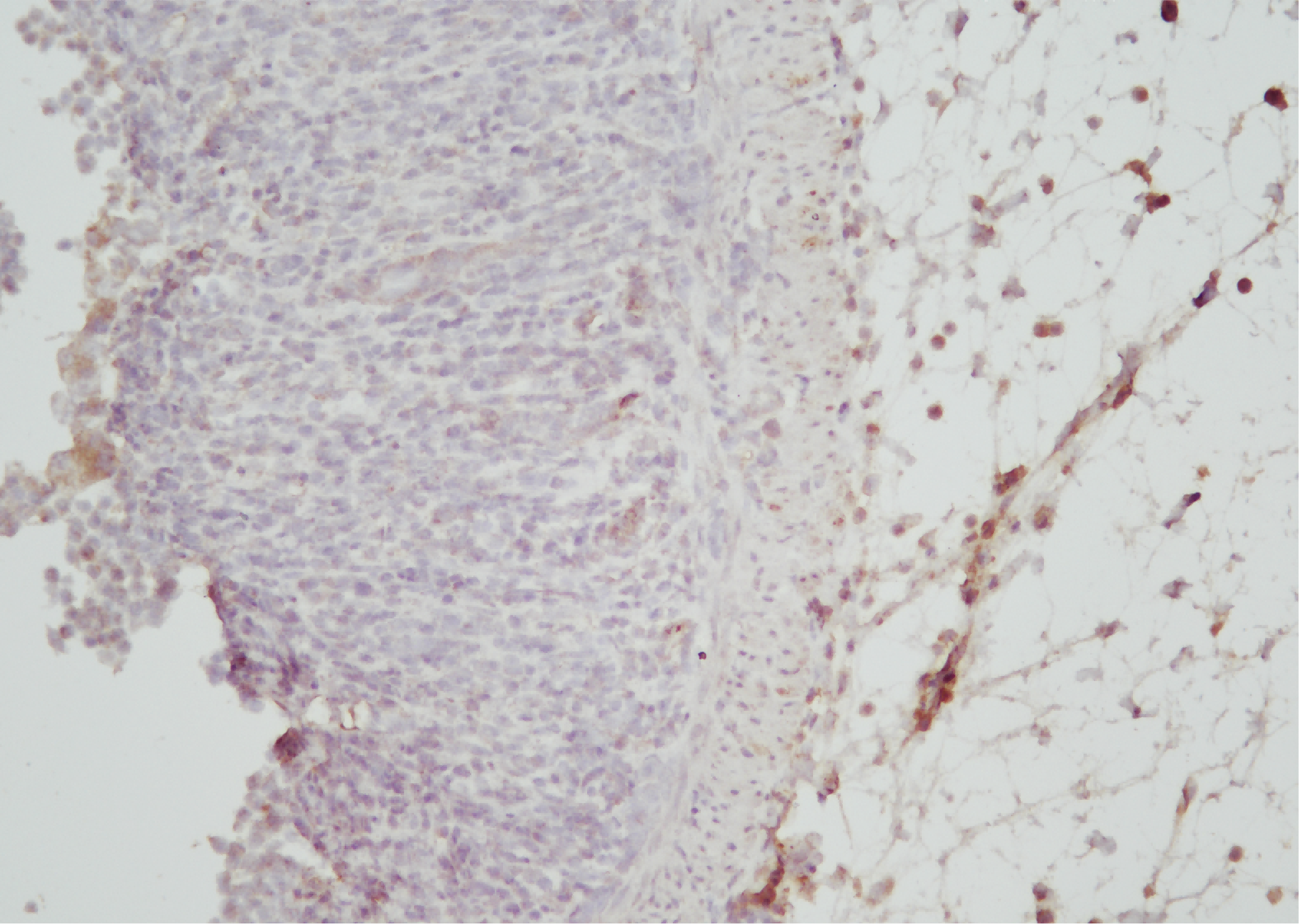

Supplement: Supplementary file 14 — EV and Appendix Figures Source Data [file 44319_2024_276_MOESM14_ESM.zip › Appendix Fig. S1/DSS_200x.png]

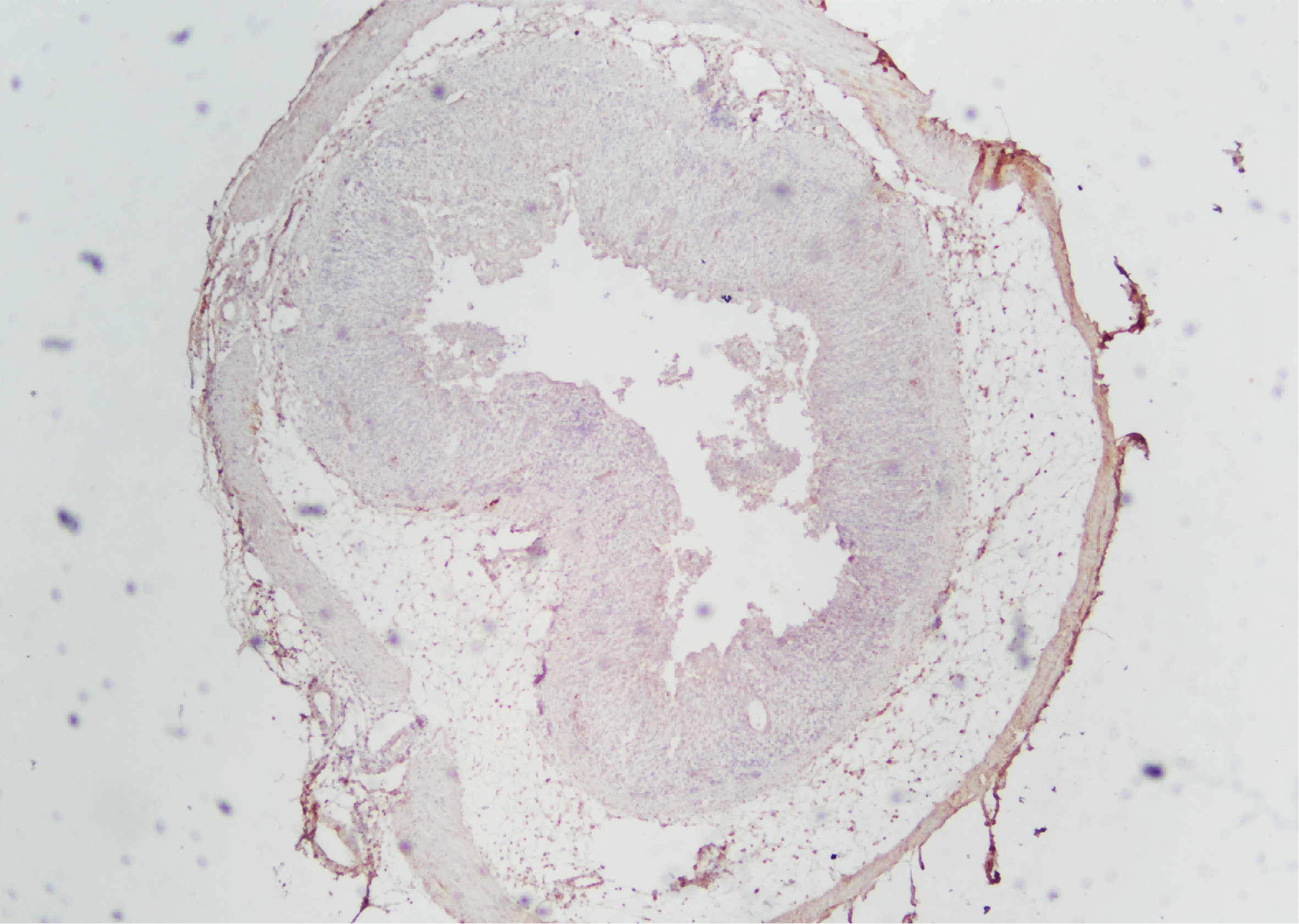

Supplement: Supplementary file 14 — EV and Appendix Figures Source Data [file 44319_2024_276_MOESM14_ESM.zip › Appendix Fig. S1/DSS_40x.png]

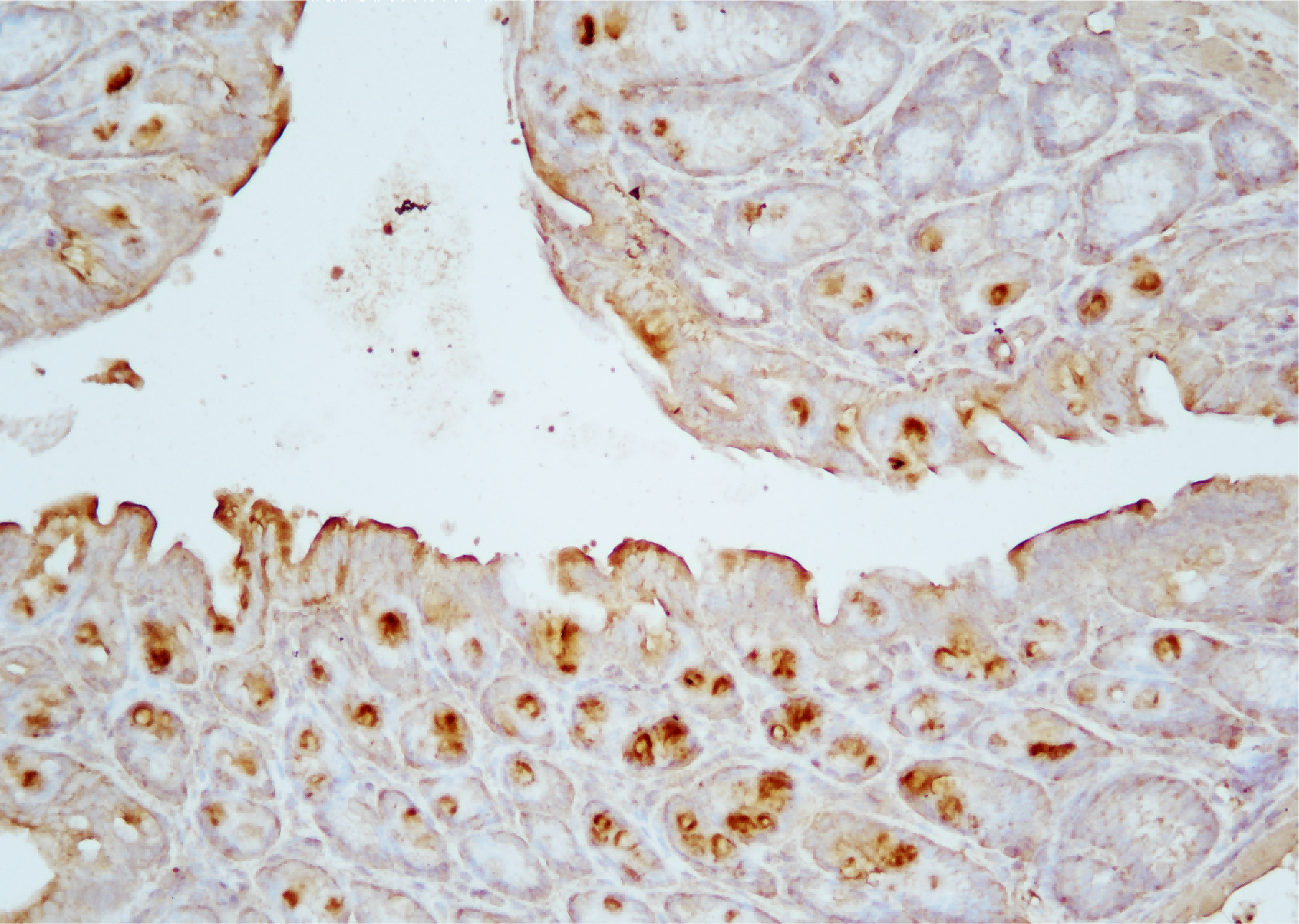

Supplement: Supplementary file 14 — EV and Appendix Figures Source Data [file 44319_2024_276_MOESM14_ESM.zip › Appendix Fig. S1/Water_200x.png]

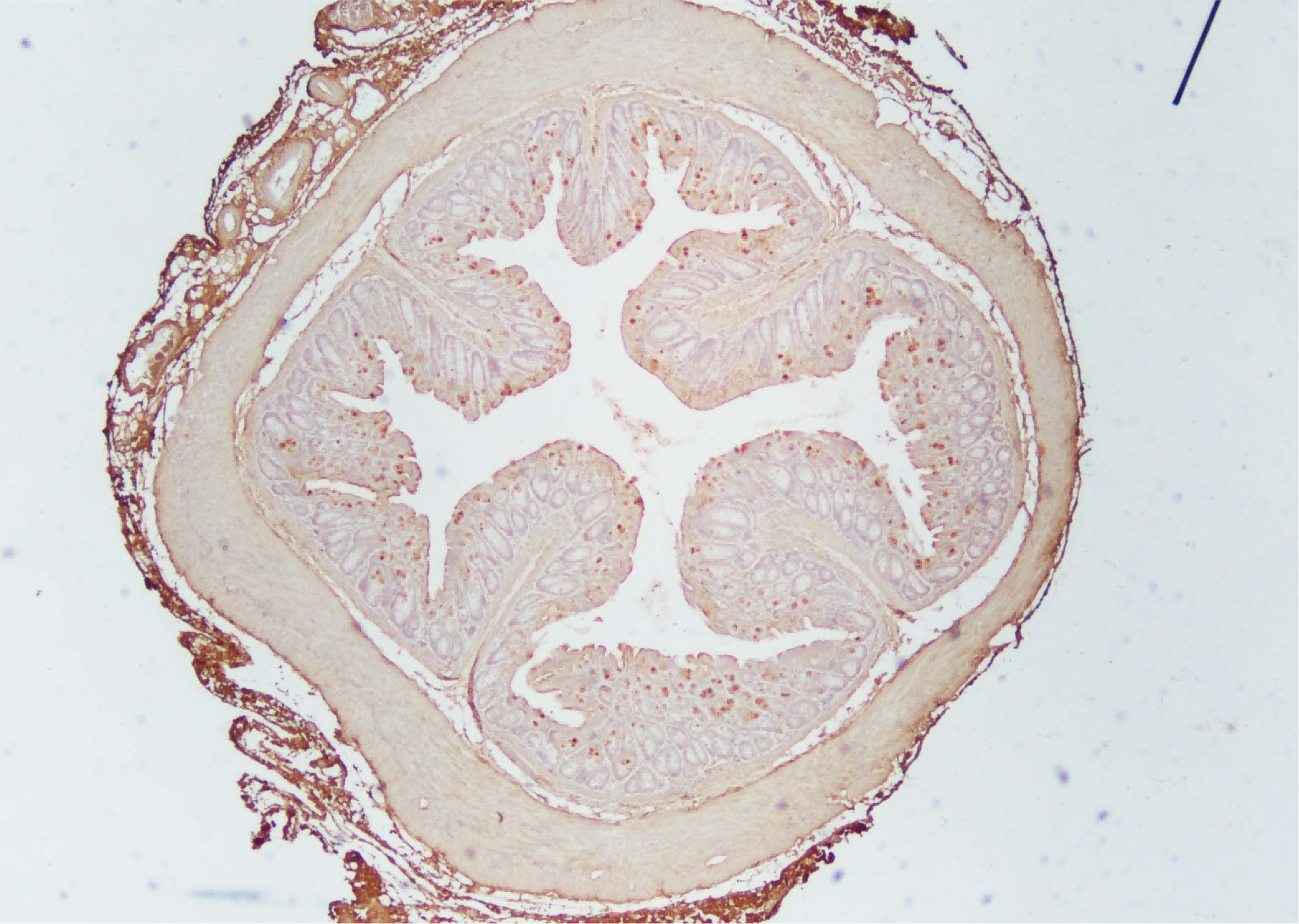

Supplement: Supplementary file 14 — EV and Appendix Figures Source Data [file 44319_2024_276_MOESM14_ESM.zip › Appendix Fig. S1/Water_40x.png]

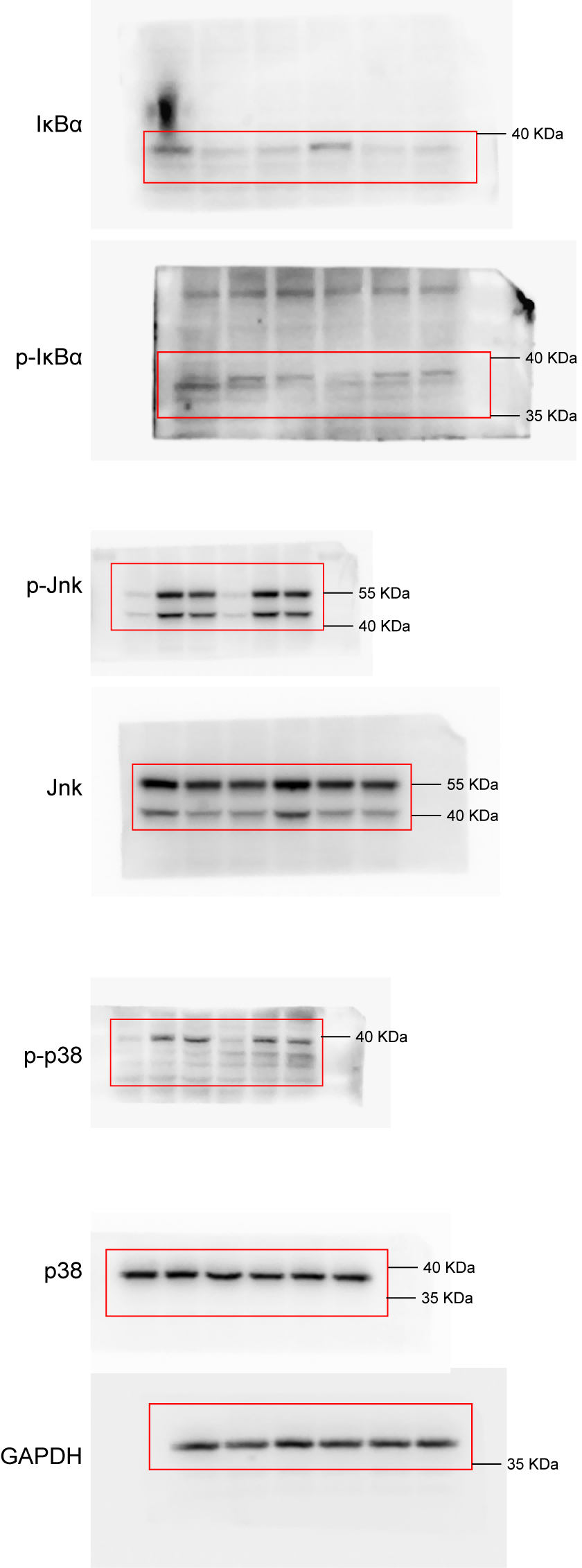

Supplement: Supplementary file 14 — EV and Appendix Figures Source Data [file 44319_2024_276_MOESM14_ESM.zip › Appendix Fig. S10/AFS10F.png]

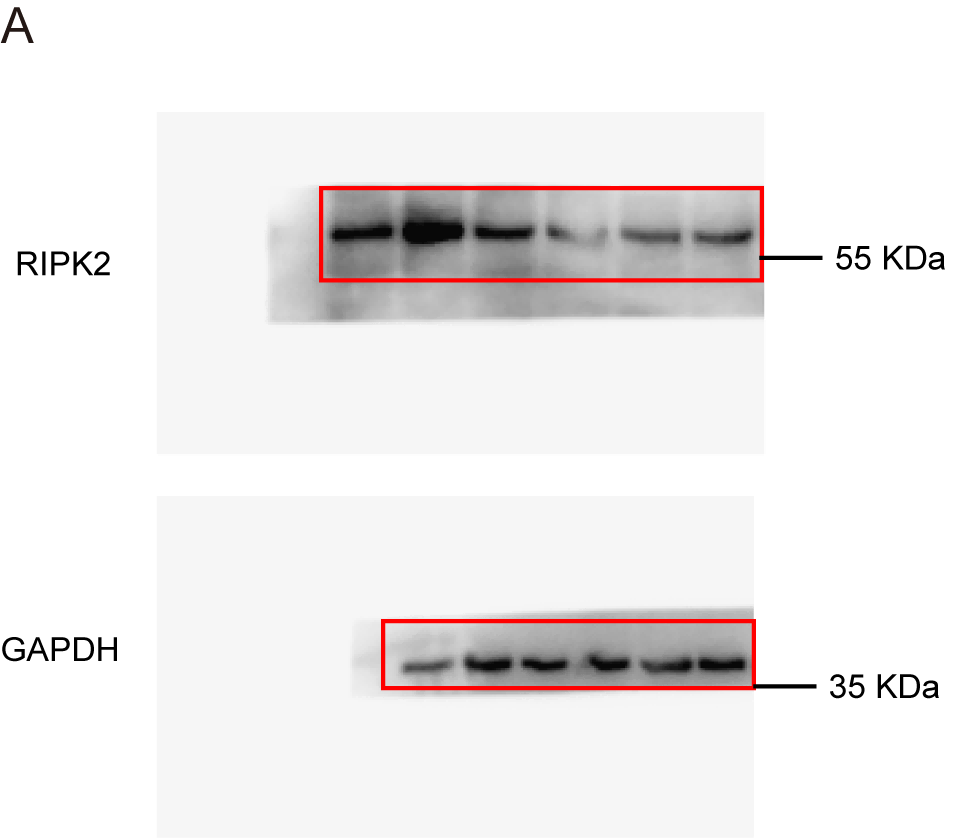

Supplement: Supplementary file 14 — EV and Appendix Figures Source Data [file 44319_2024_276_MOESM14_ESM.zip › Appendix Fig. S11/AFS11A.png]

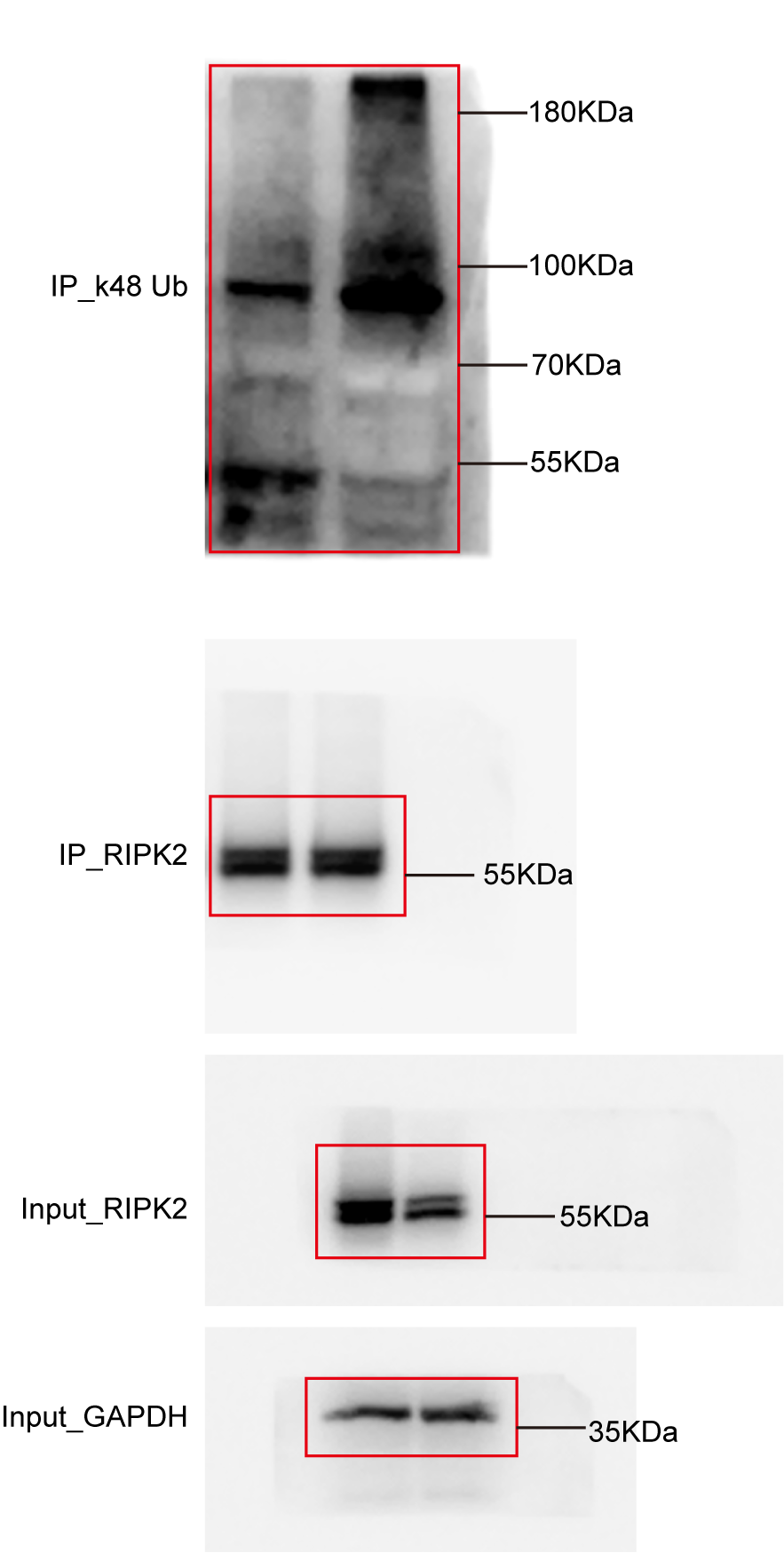

Supplement: Supplementary file 14 — EV and Appendix Figures Source Data [file 44319_2024_276_MOESM14_ESM.zip › Appendix Fig. S11/AFS11B.tif]

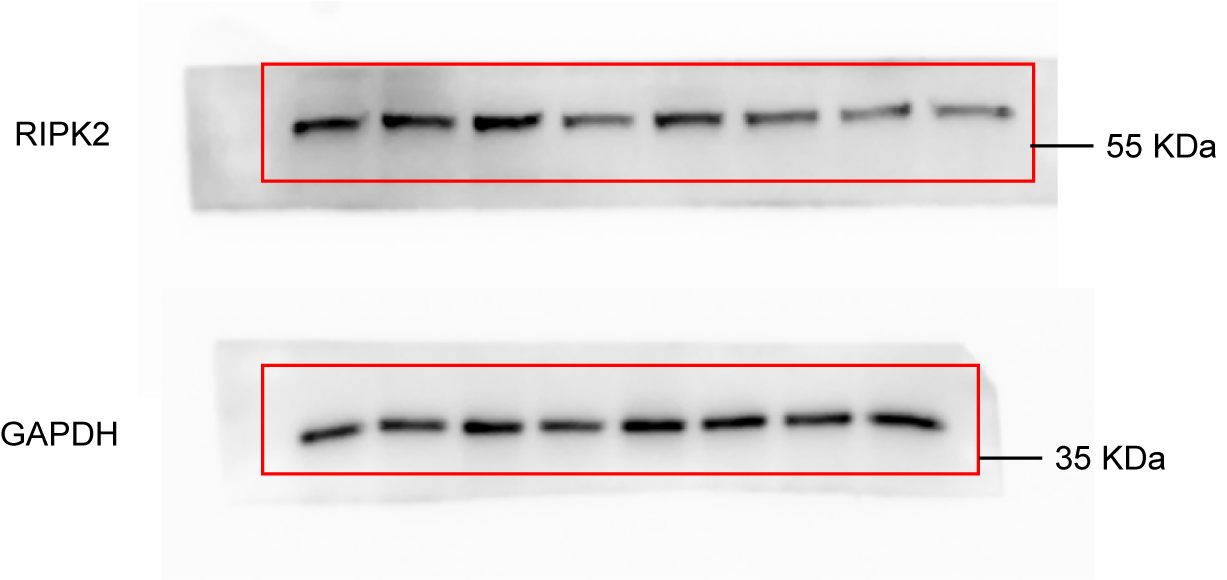

Supplement: Supplementary file 14 — EV and Appendix Figures Source Data [file 44319_2024_276_MOESM14_ESM.zip › Appendix Fig. S12/AFS12A.tif]

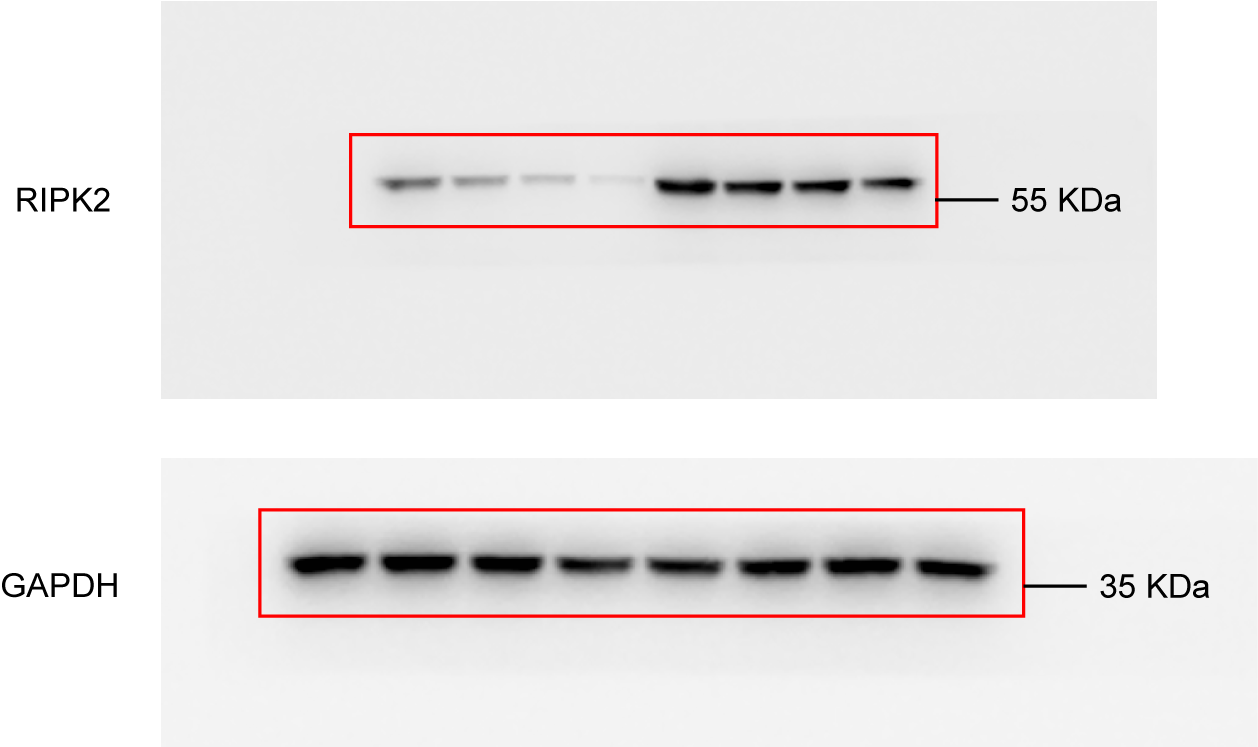

Supplement: Supplementary file 14 — EV and Appendix Figures Source Data [file 44319_2024_276_MOESM14_ESM.zip › Appendix Fig. S12/AFS12B.tif]

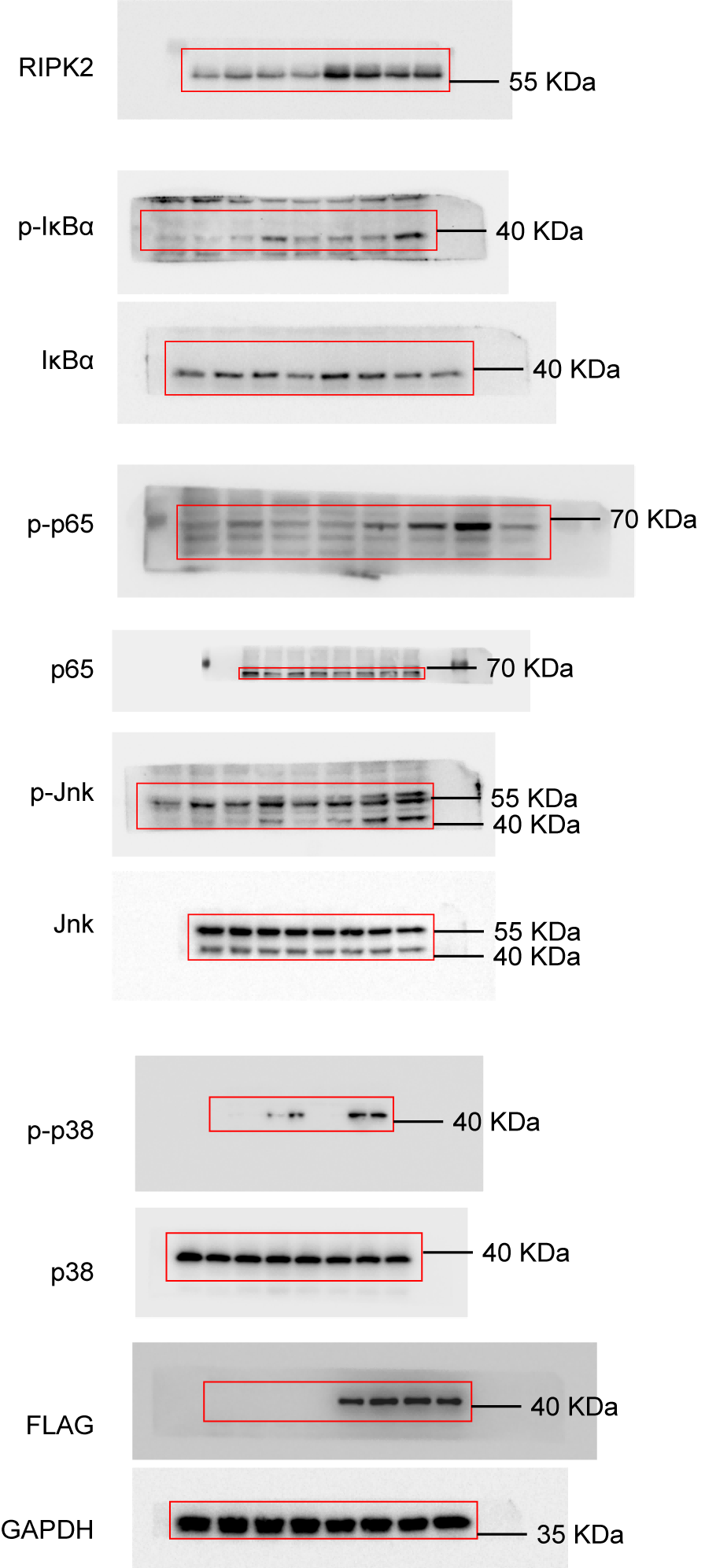

Supplement: Supplementary file 14 — EV and Appendix Figures Source Data [file 44319_2024_276_MOESM14_ESM.zip › Appendix Fig. S13/AFS13A.png]

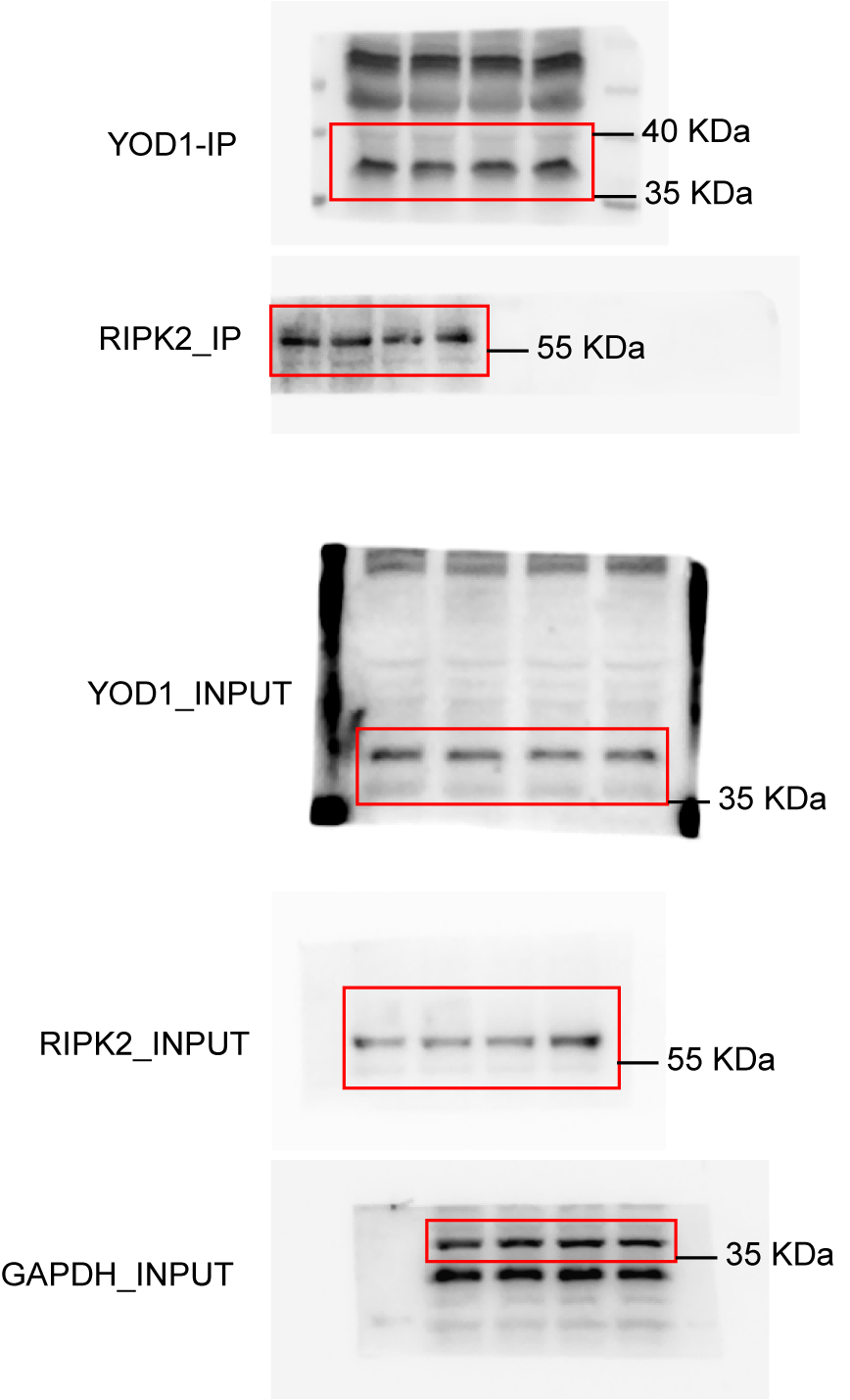

Supplement: Supplementary file 14 — EV and Appendix Figures Source Data [file 44319_2024_276_MOESM14_ESM.zip › Appendix Fig. S14/AFS14A.png]

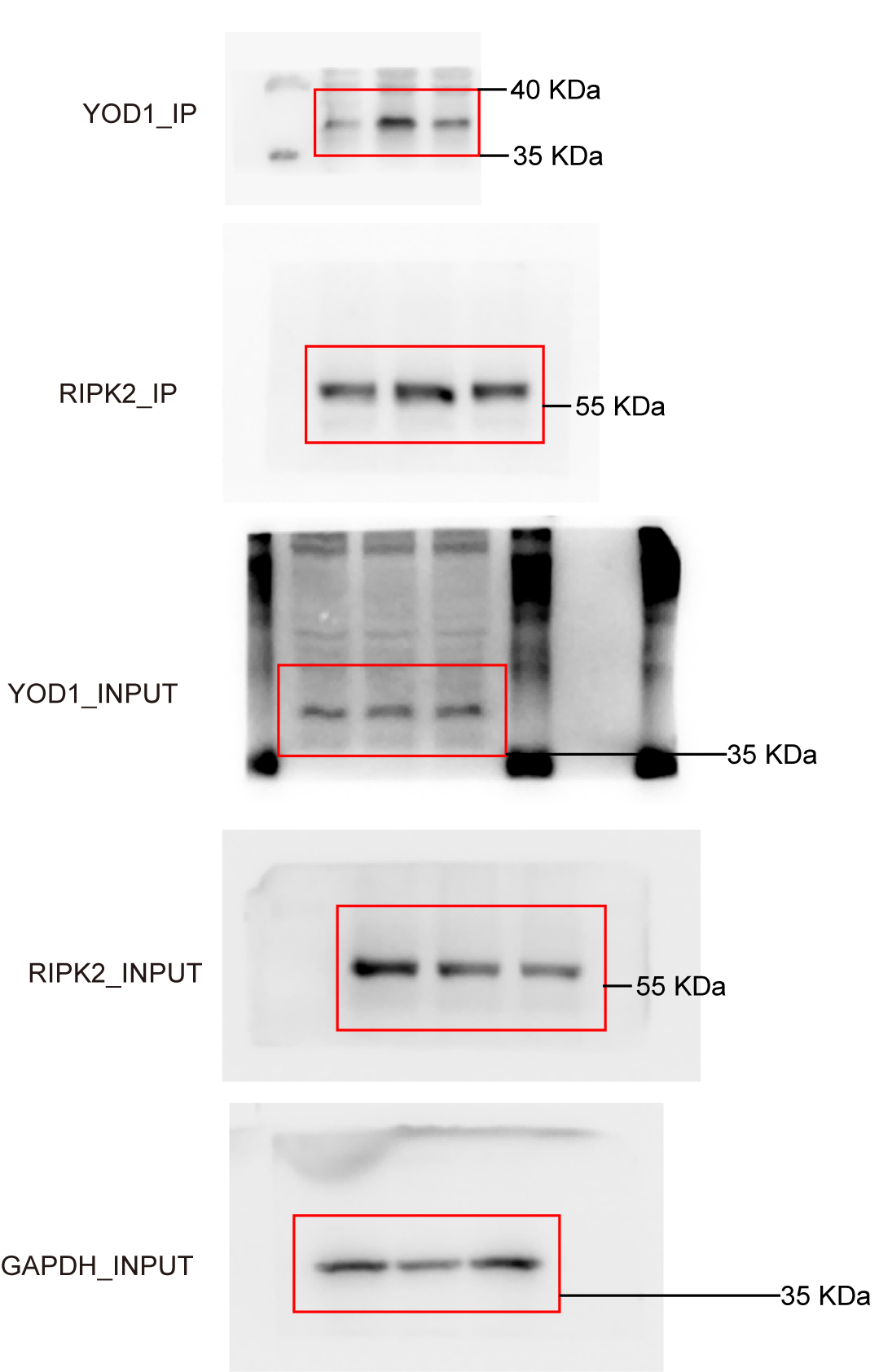

Supplement: Supplementary file 14 — EV and Appendix Figures Source Data [file 44319_2024_276_MOESM14_ESM.zip › Appendix Fig. S14/AFS14B.png]

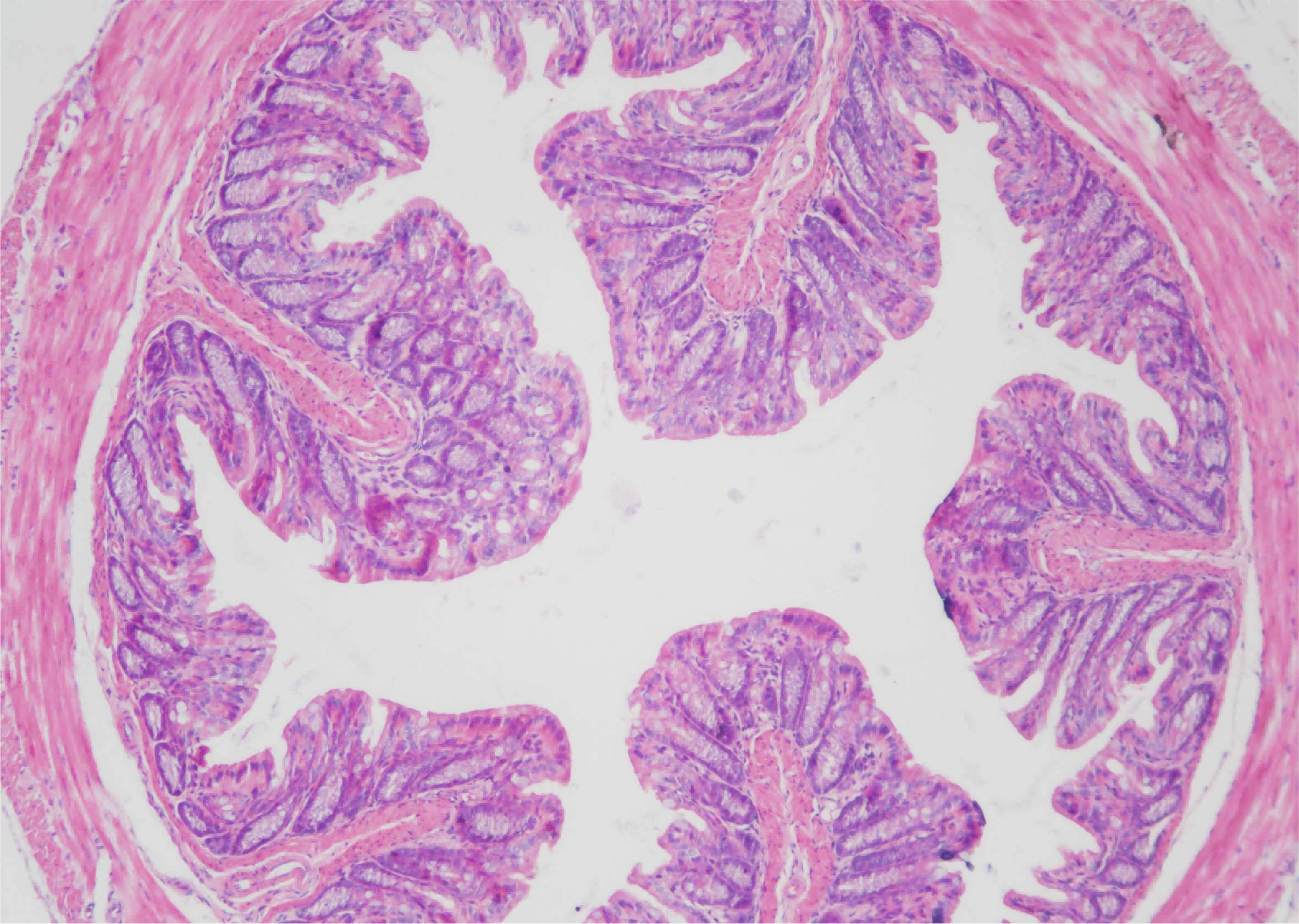

Supplement: Supplementary file 14 — EV and Appendix Figures Source Data [file 44319_2024_276_MOESM14_ESM.zip › Appendix Fig. S2/AFS2B/100×/Yod1++/1.png]

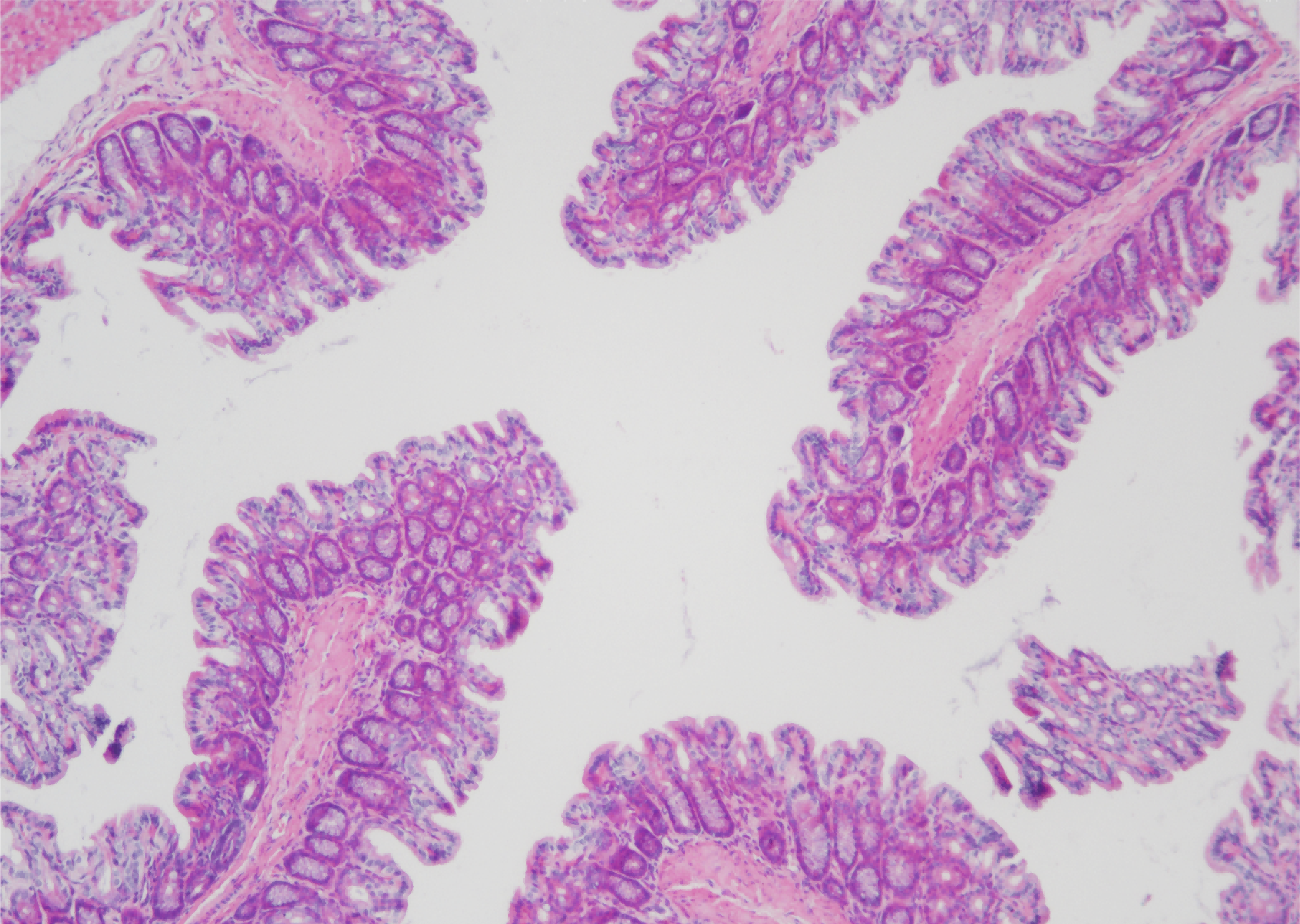

Supplement: Supplementary file 14 — EV and Appendix Figures Source Data [file 44319_2024_276_MOESM14_ESM.zip › Appendix Fig. S2/AFS2B/100×/Yod1++/2.png]

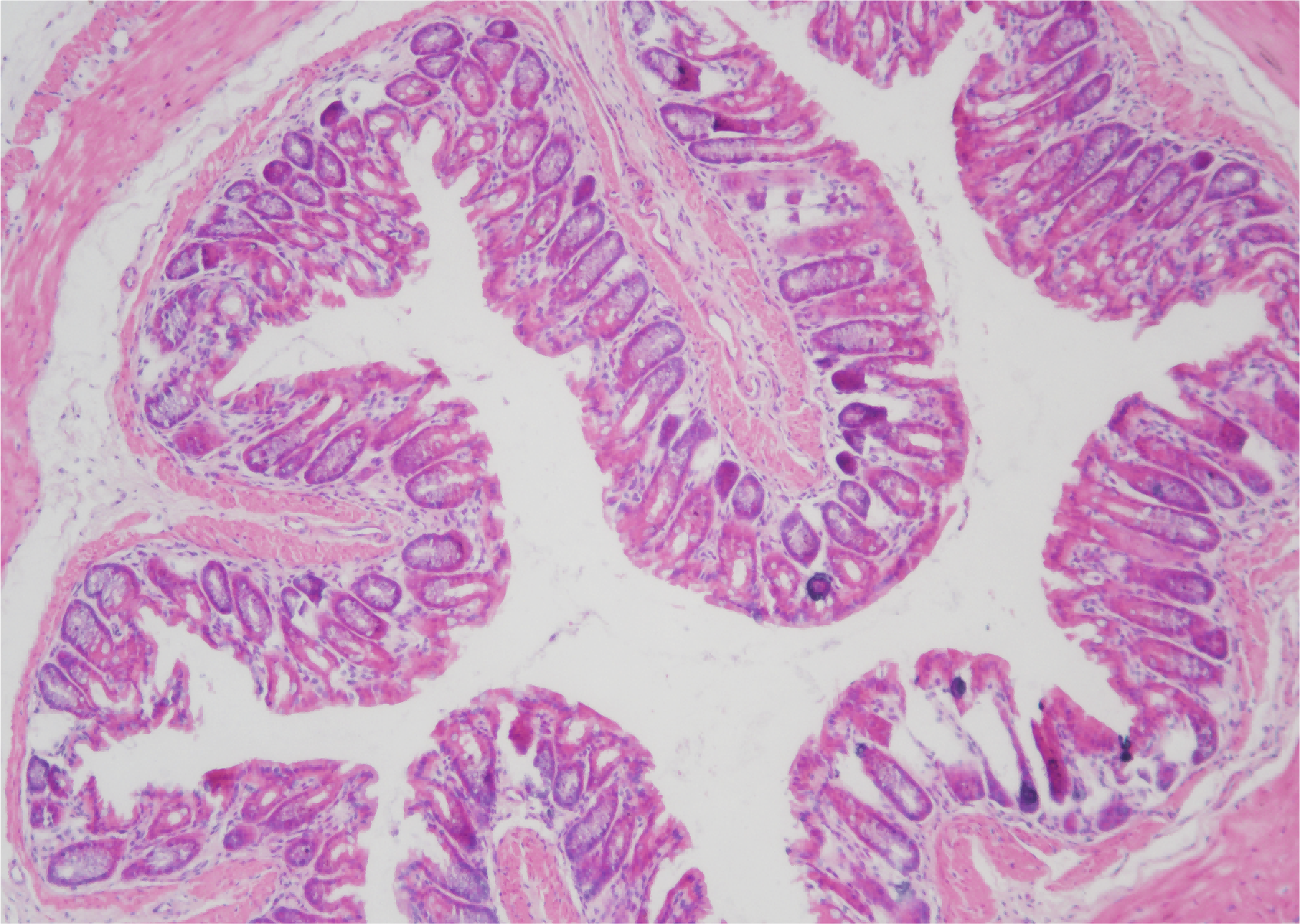

Supplement: Supplementary file 14 — EV and Appendix Figures Source Data [file 44319_2024_276_MOESM14_ESM.zip › Appendix Fig. S2/AFS2B/100×/Yod1++/3.png]

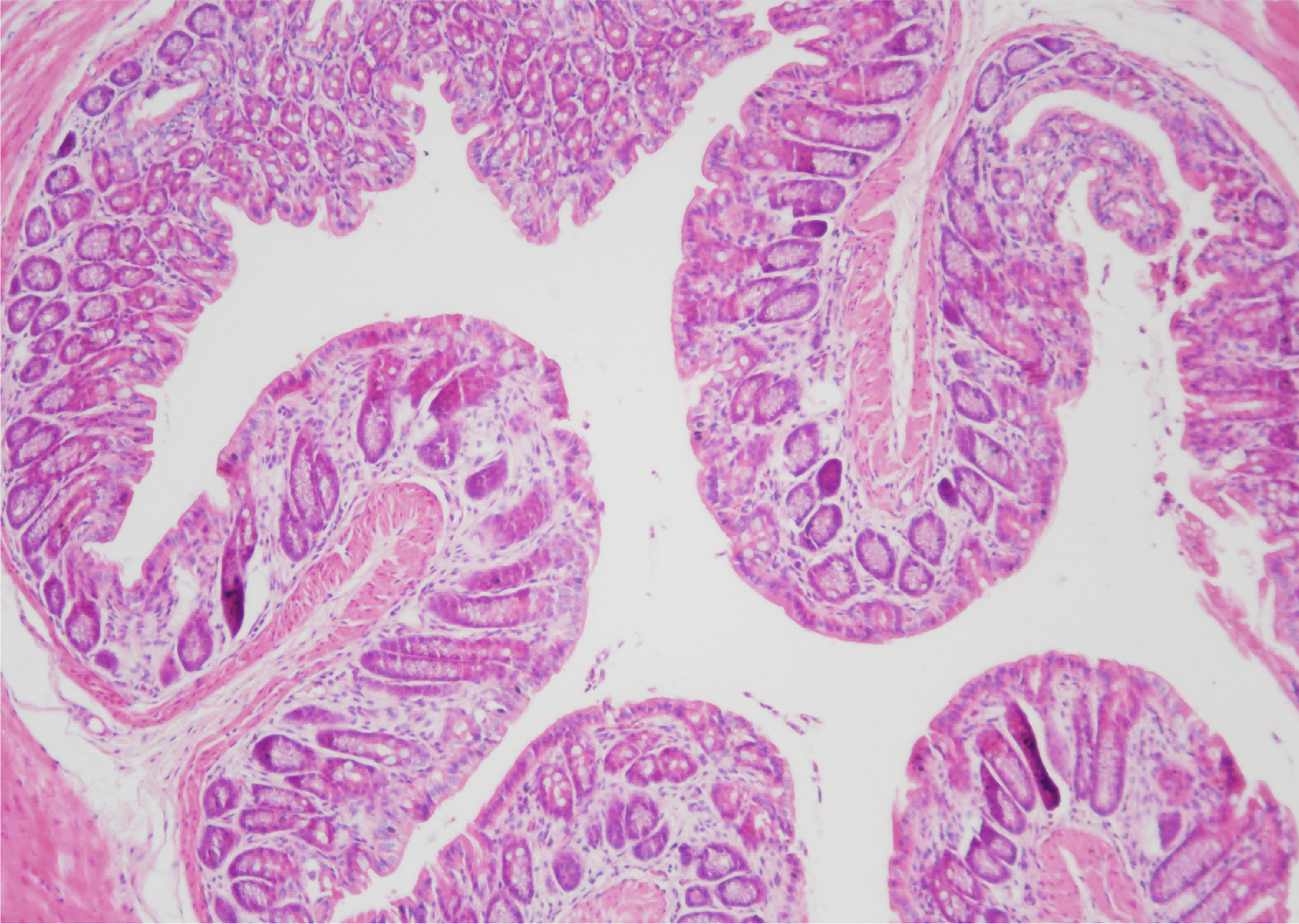

Supplement: Supplementary file 14 — EV and Appendix Figures Source Data [file 44319_2024_276_MOESM14_ESM.zip › Appendix Fig. S2/AFS2B/100×/Yod1++/4.png]

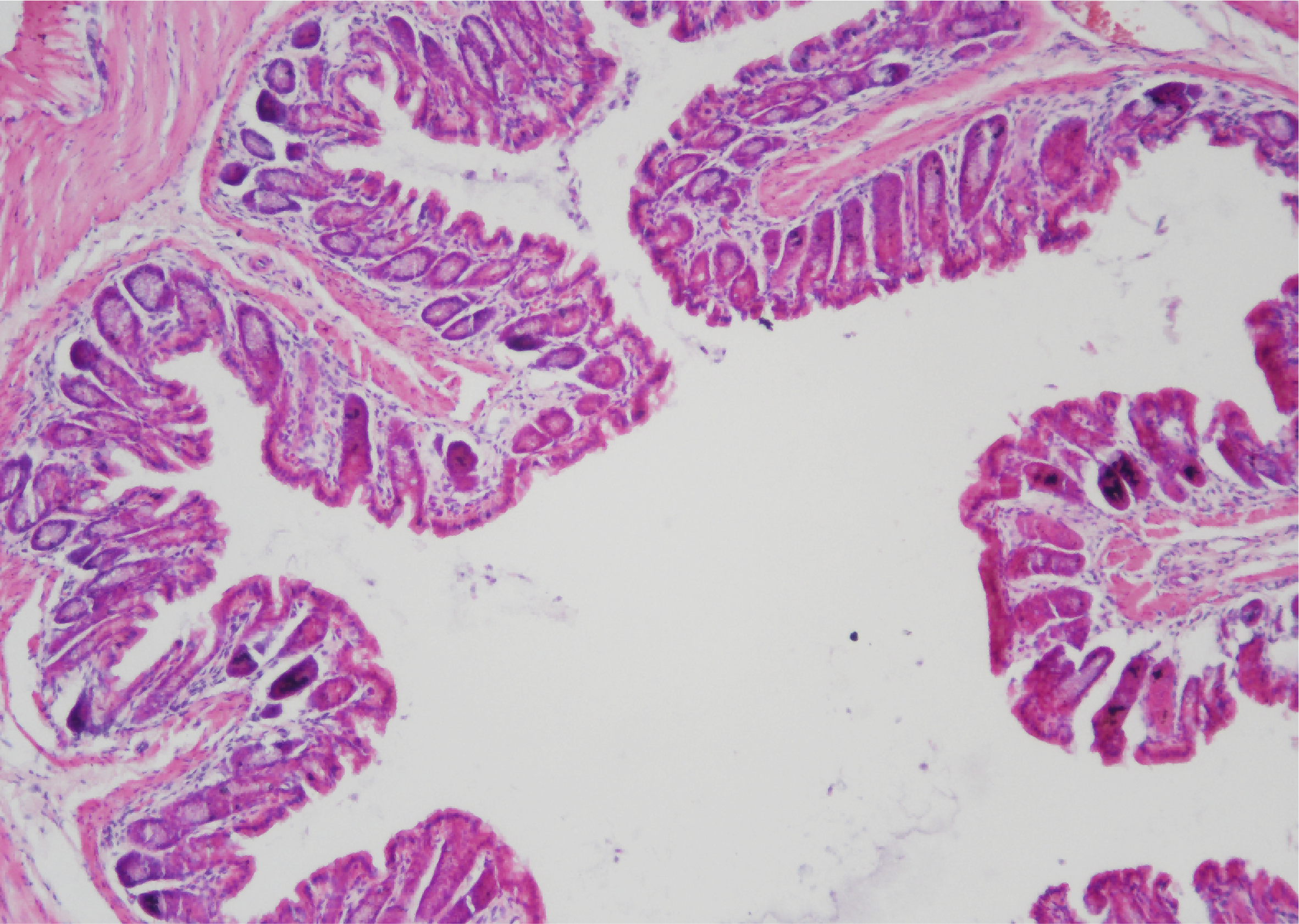

Supplement: Supplementary file 14 — EV and Appendix Figures Source Data [file 44319_2024_276_MOESM14_ESM.zip › Appendix Fig. S2/AFS2B/100×/Yod1++/5.png]

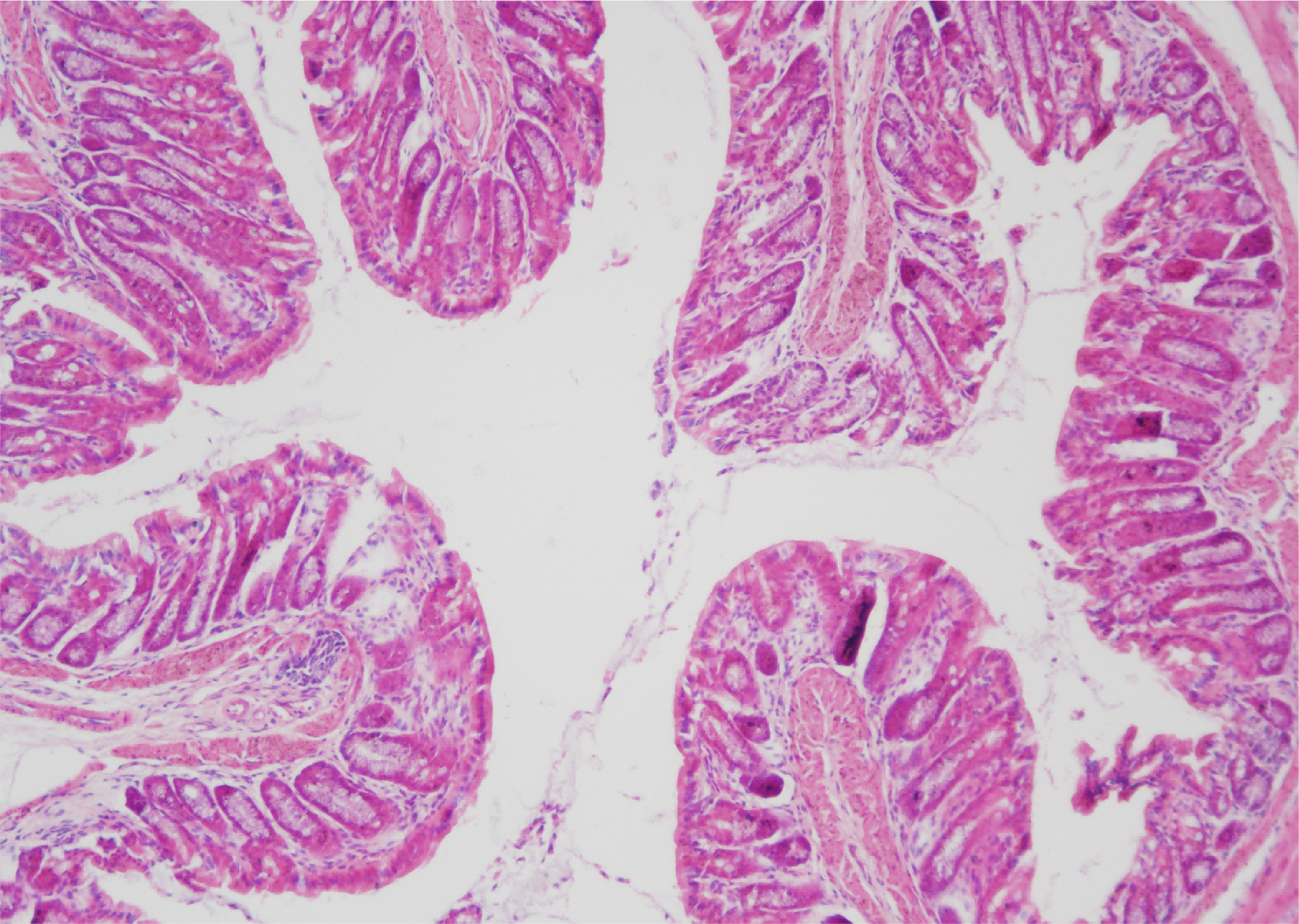

Supplement: Supplementary file 14 — EV and Appendix Figures Source Data [file 44319_2024_276_MOESM14_ESM.zip › Appendix Fig. S2/AFS2B/100×/Yod1++/6.png]

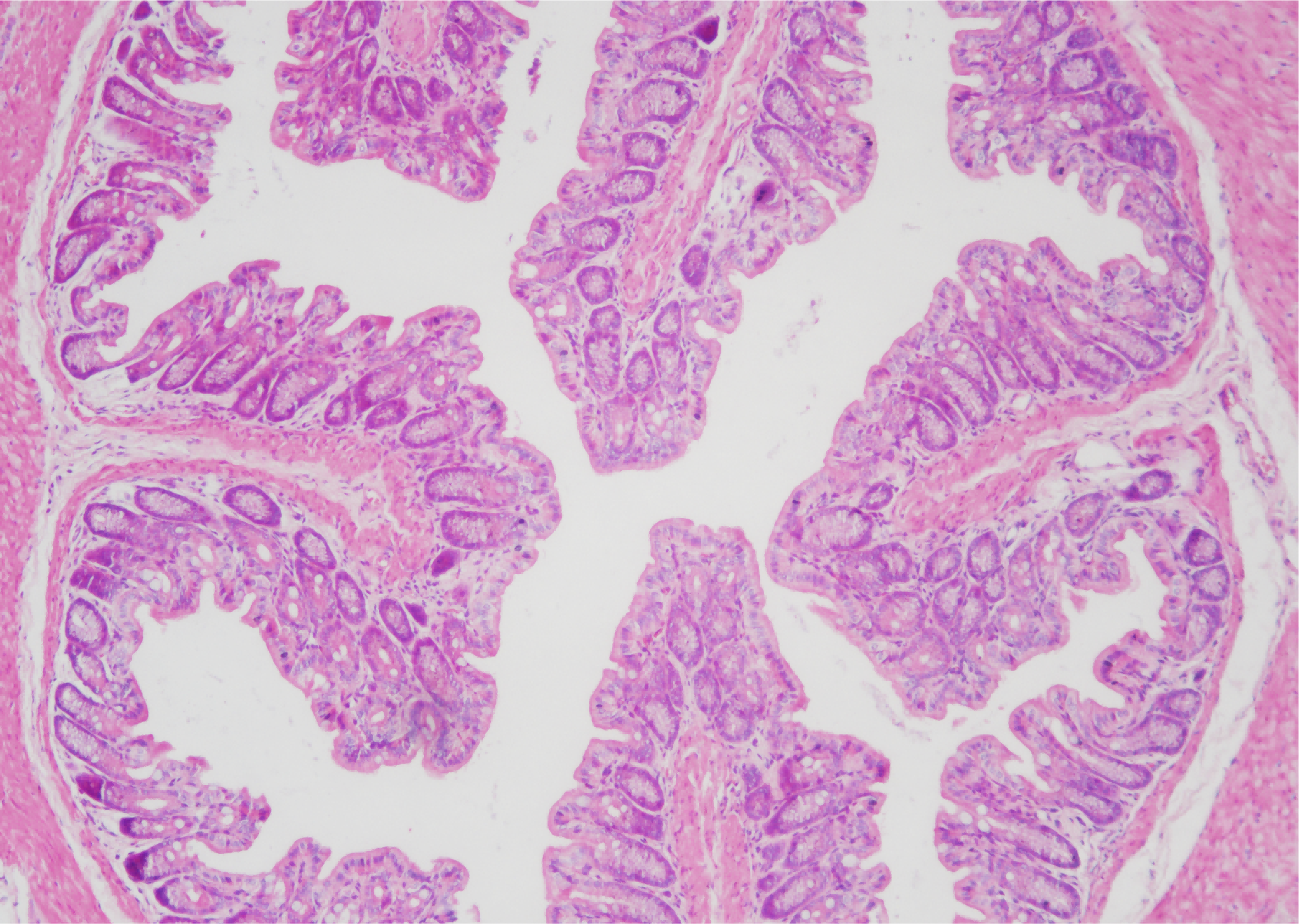

Supplement: Supplementary file 14 — EV and Appendix Figures Source Data [file 44319_2024_276_MOESM14_ESM.zip › Appendix Fig. S2/AFS2B/100×/Yod1++/7.png]

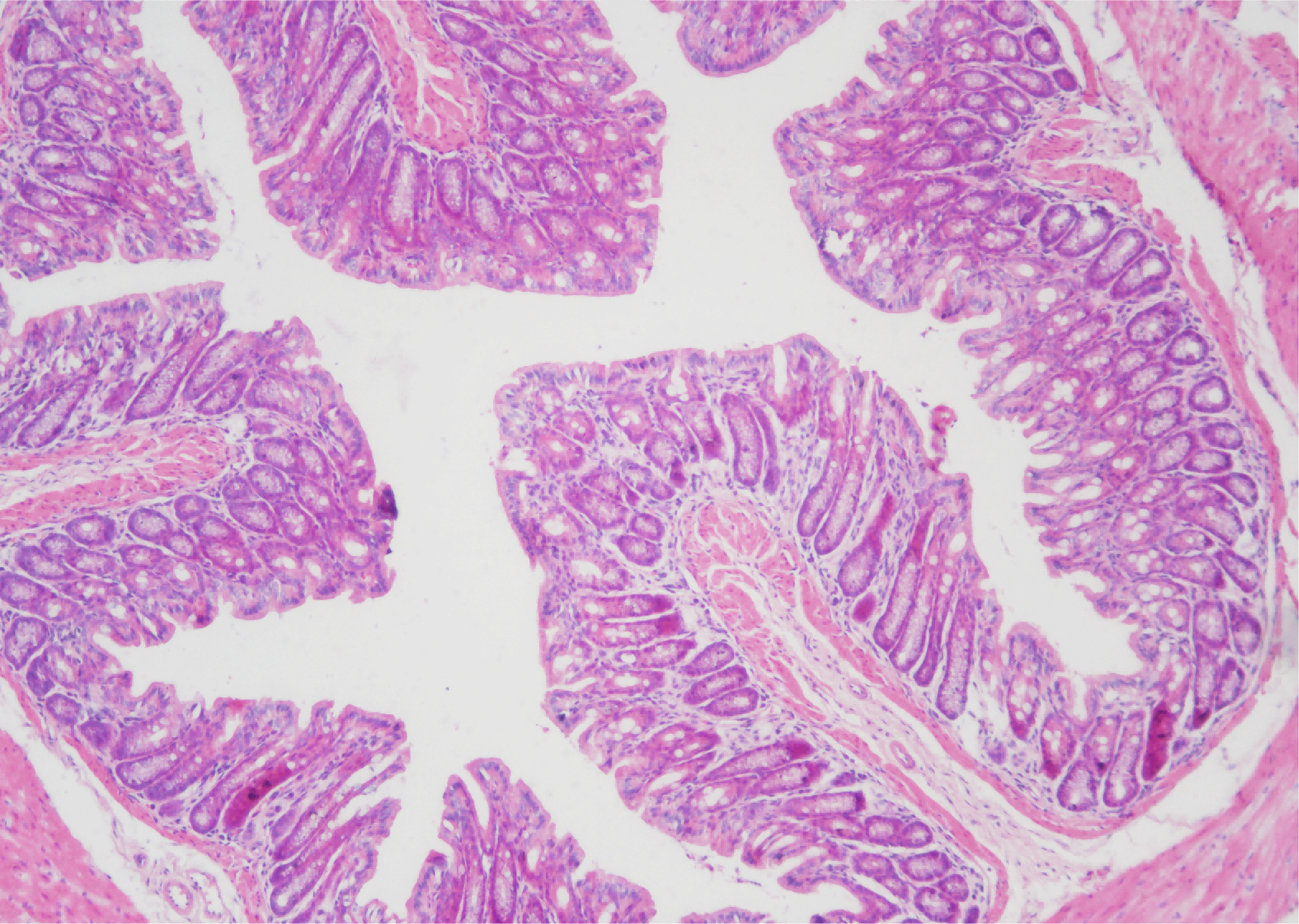

Supplement: Supplementary file 14 — EV and Appendix Figures Source Data [file 44319_2024_276_MOESM14_ESM.zip › Appendix Fig. S2/AFS2B/100×/Yod1--/1.png]

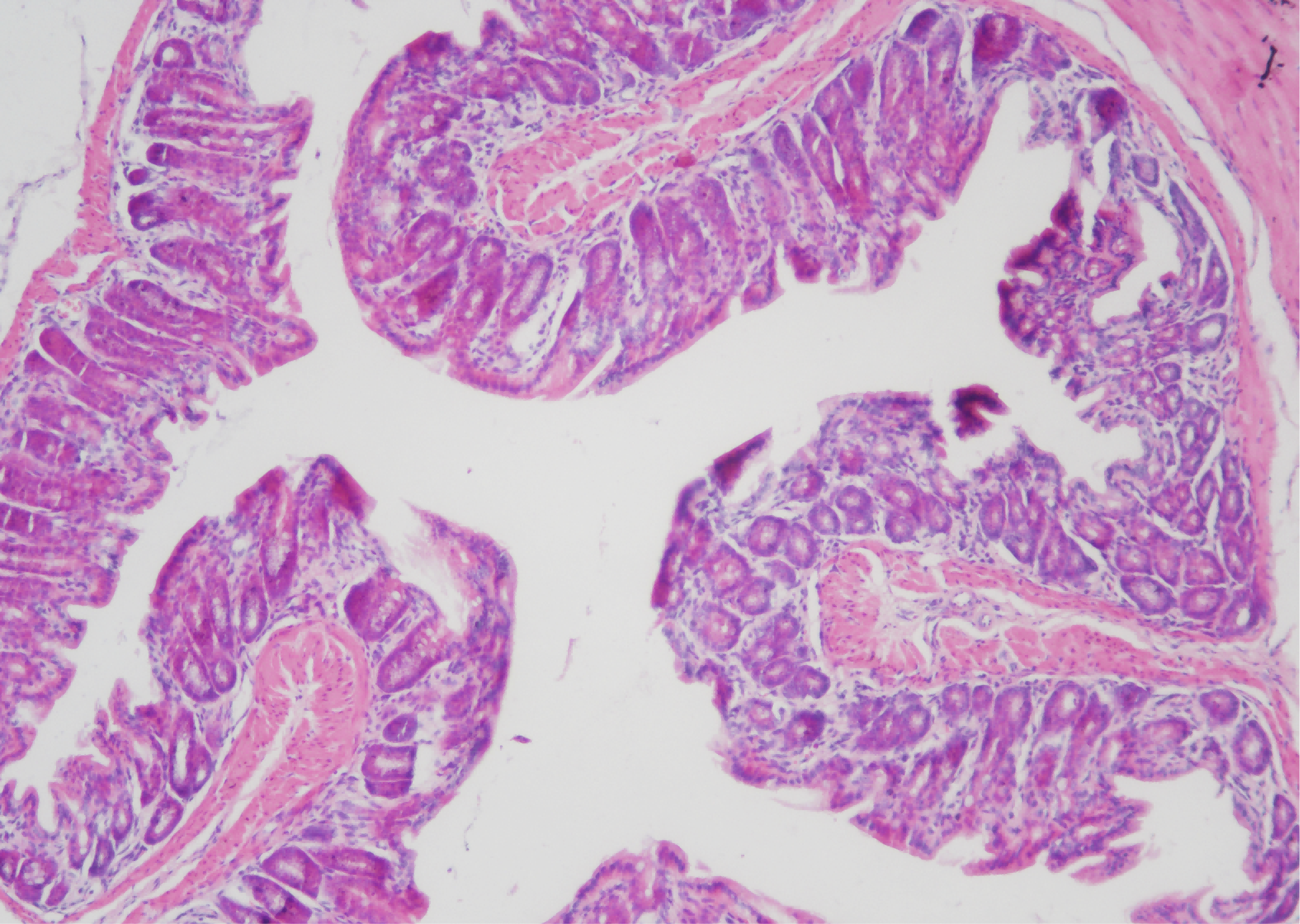

Supplement: Supplementary file 14 — EV and Appendix Figures Source Data [file 44319_2024_276_MOESM14_ESM.zip › Appendix Fig. S2/AFS2B/100×/Yod1--/2.png]

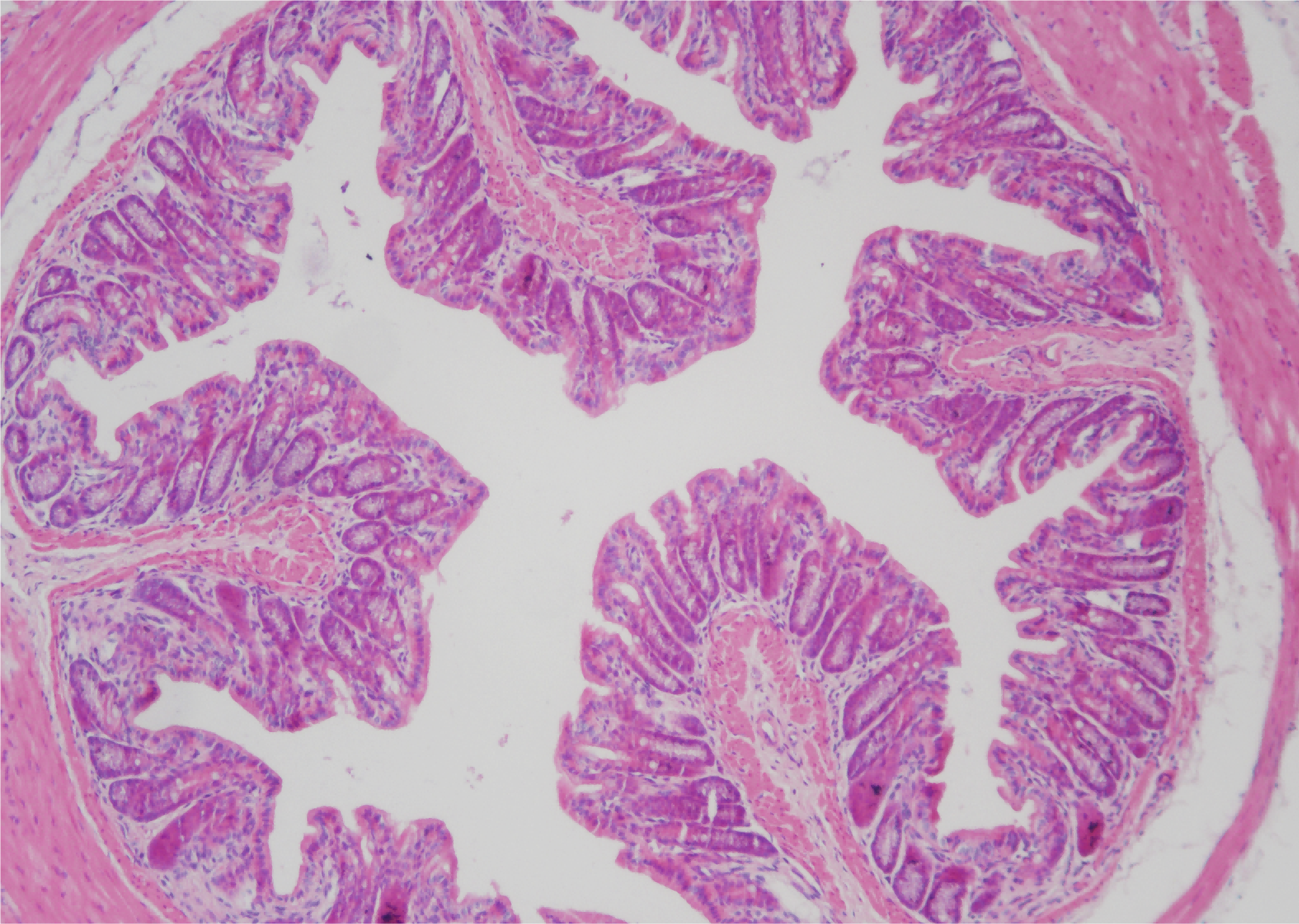

Supplement: Supplementary file 14 — EV and Appendix Figures Source Data [file 44319_2024_276_MOESM14_ESM.zip › Appendix Fig. S2/AFS2B/100×/Yod1--/3.png]

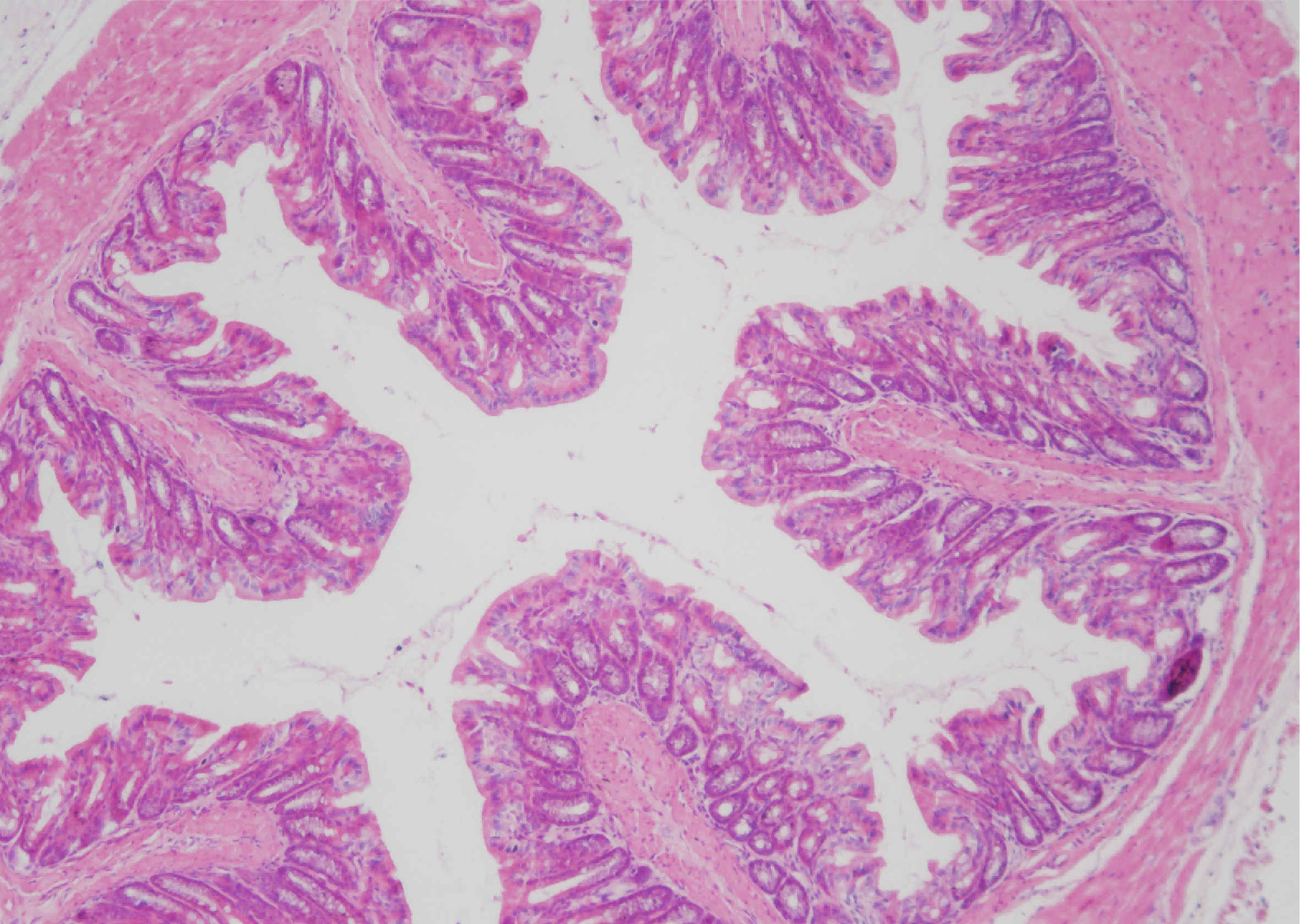

Supplement: Supplementary file 14 — EV and Appendix Figures Source Data [file 44319_2024_276_MOESM14_ESM.zip › Appendix Fig. S2/AFS2B/100×/Yod1--/4.png]

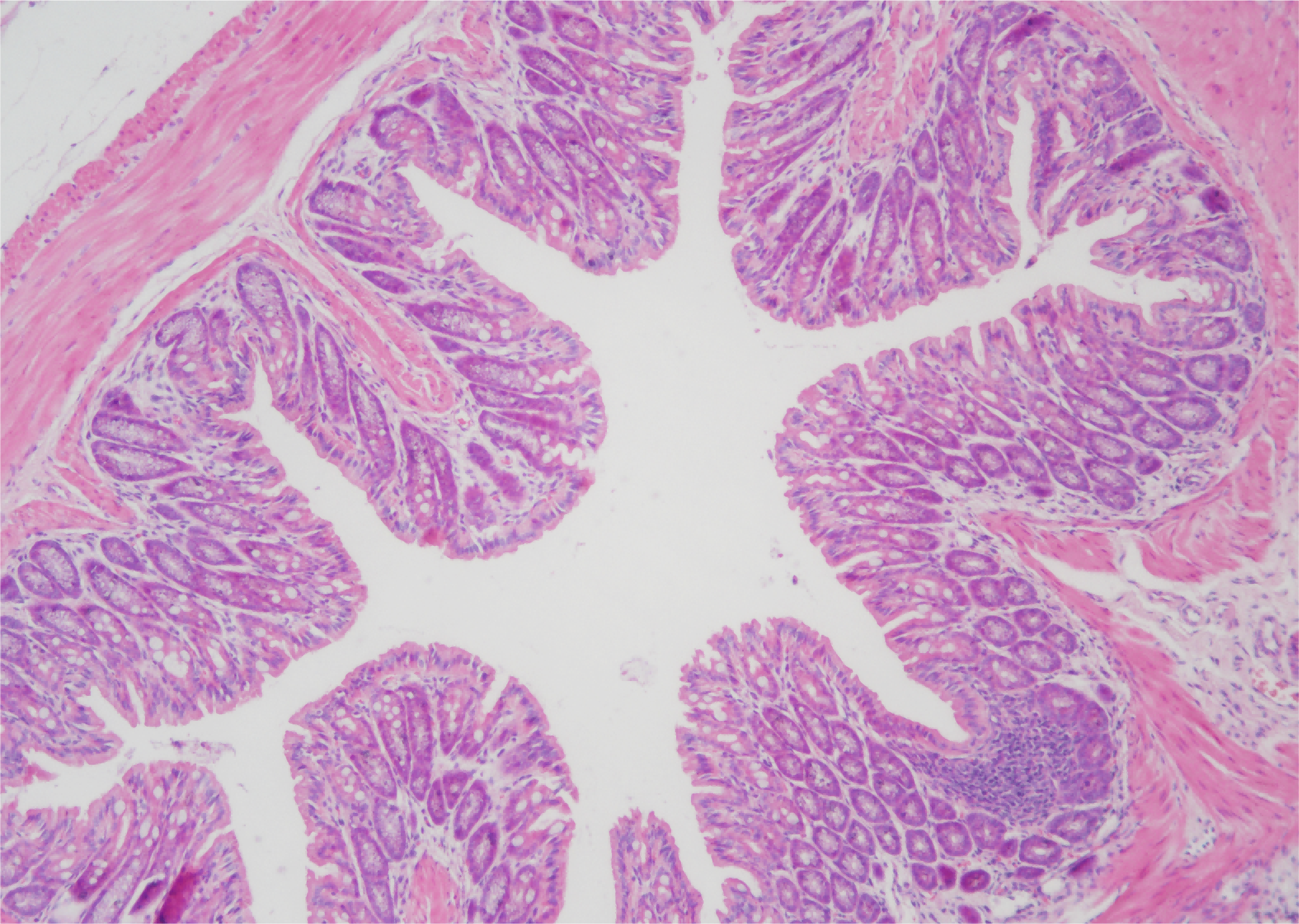

Supplement: Supplementary file 14 — EV and Appendix Figures Source Data [file 44319_2024_276_MOESM14_ESM.zip › Appendix Fig. S2/AFS2B/100×/Yod1--/5.png]

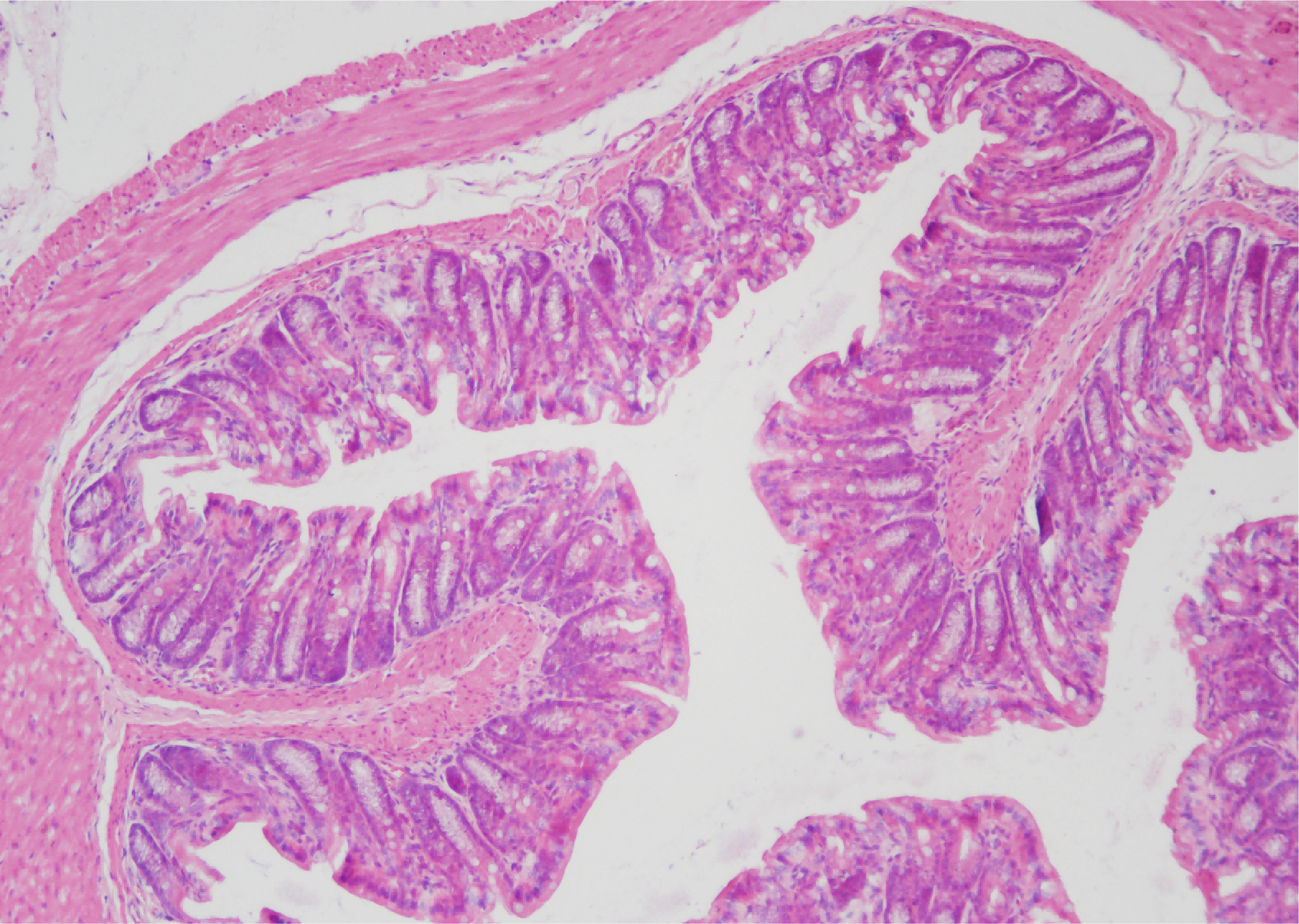

Supplement: Supplementary file 14 — EV and Appendix Figures Source Data [file 44319_2024_276_MOESM14_ESM.zip › Appendix Fig. S2/AFS2B/100×/Yod1--/6.png]

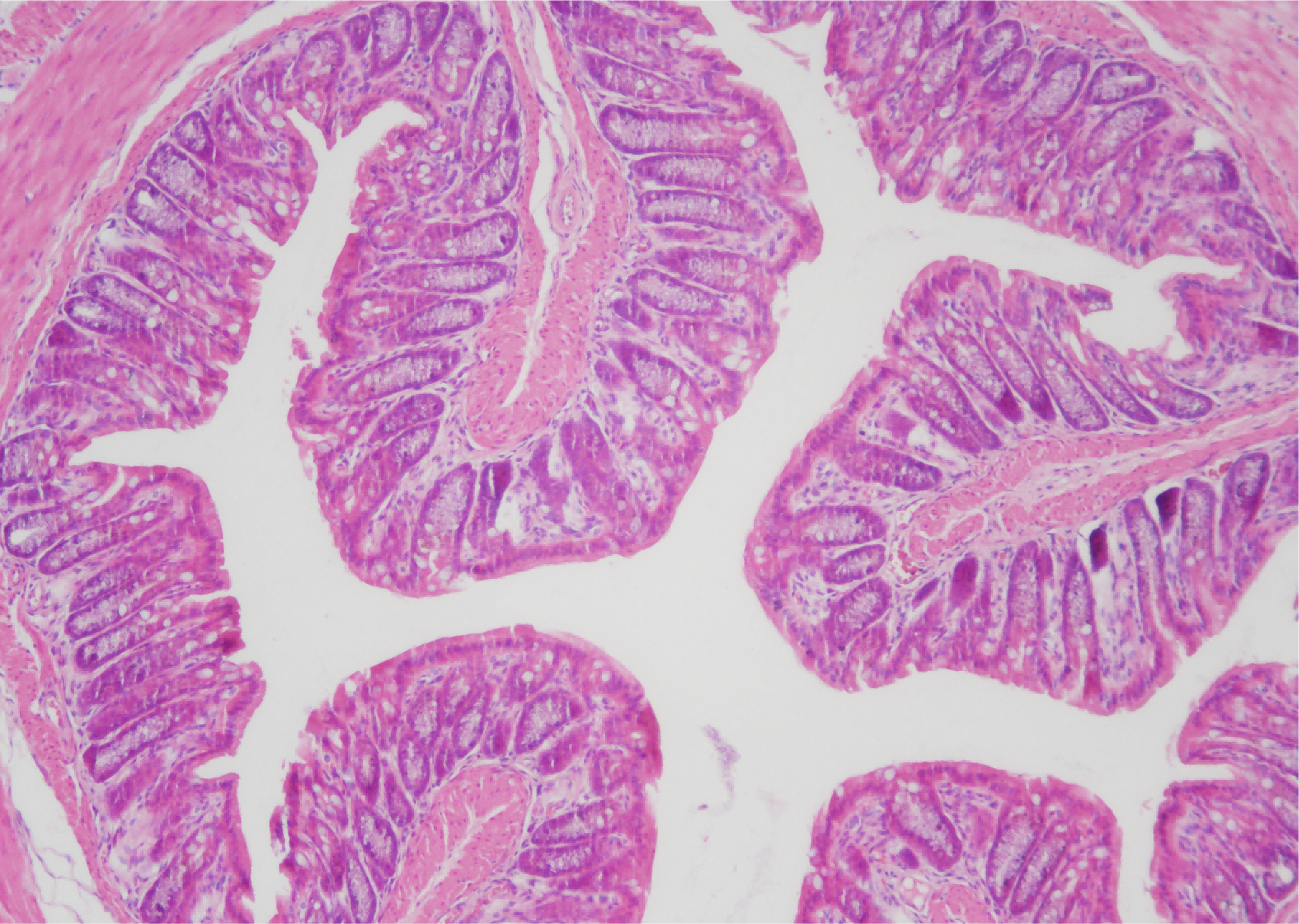

Supplement: Supplementary file 14 — EV and Appendix Figures Source Data [file 44319_2024_276_MOESM14_ESM.zip › Appendix Fig. S2/AFS2B/100×/Yod1--/7.png]

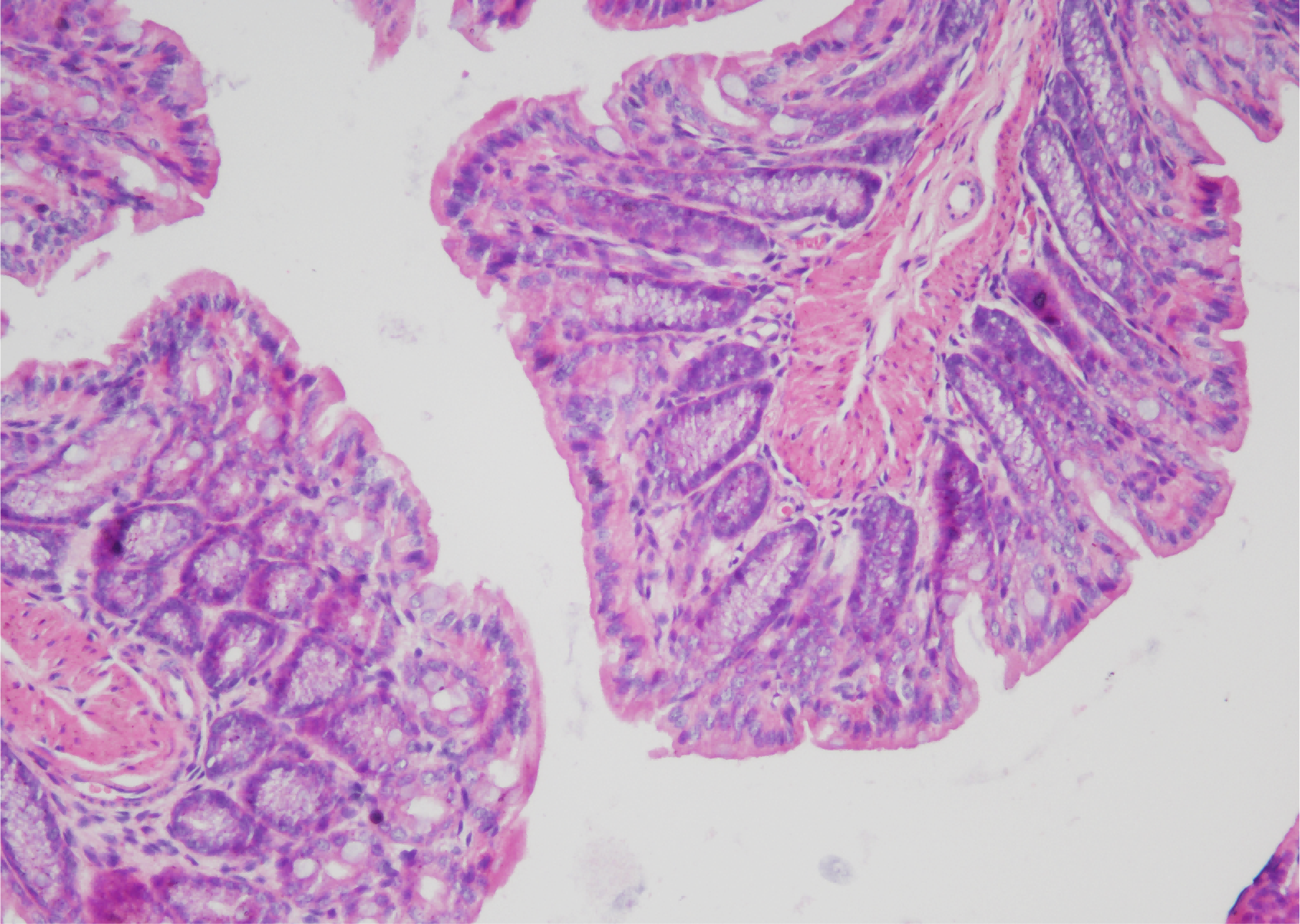

Supplement: Supplementary file 14 — EV and Appendix Figures Source Data [file 44319_2024_276_MOESM14_ESM.zip › Appendix Fig. S2/AFS2B/200×/Yod1++/1.png]

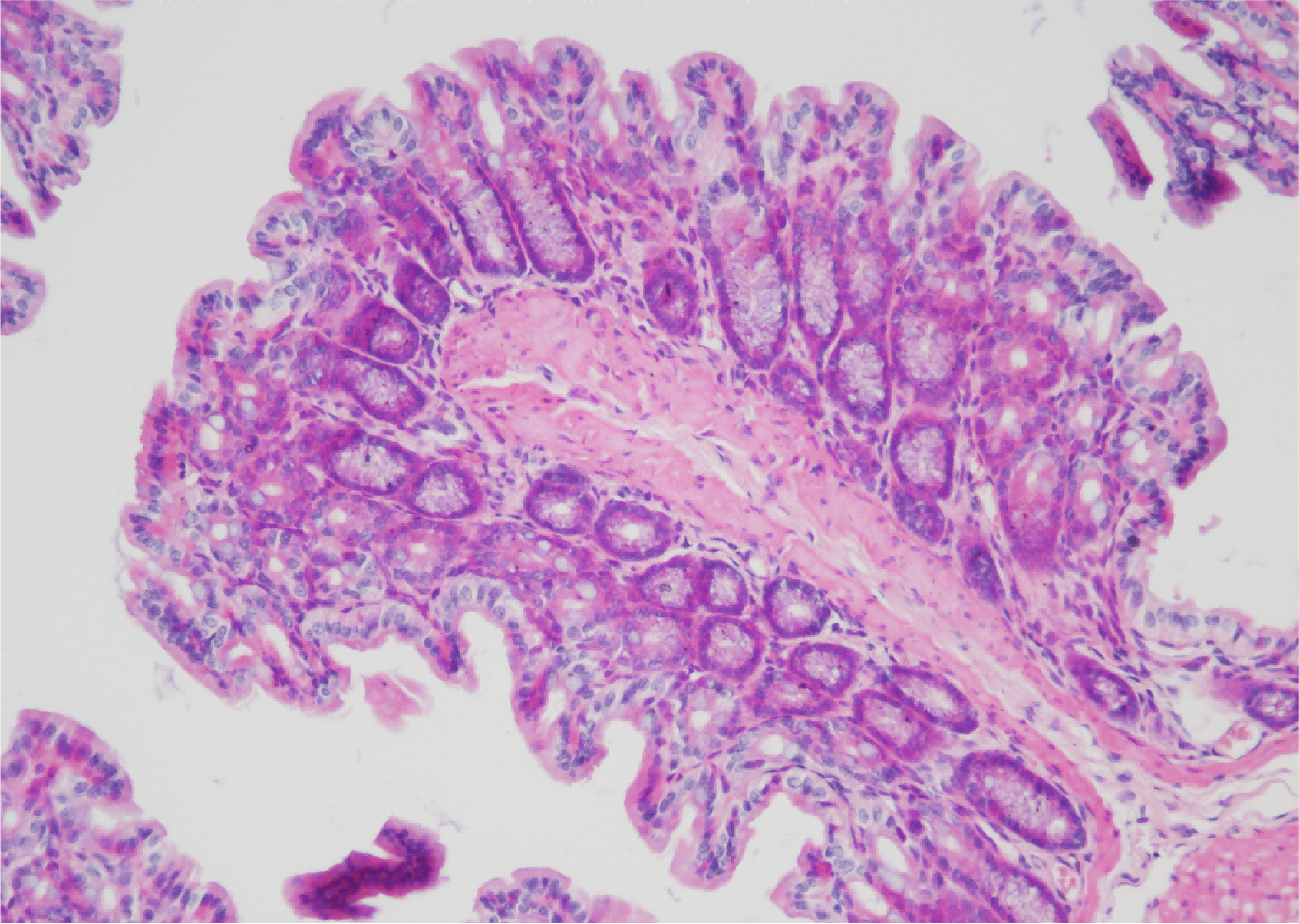

Supplement: Supplementary file 14 — EV and Appendix Figures Source Data [file 44319_2024_276_MOESM14_ESM.zip › Appendix Fig. S2/AFS2B/200×/Yod1++/2.png]

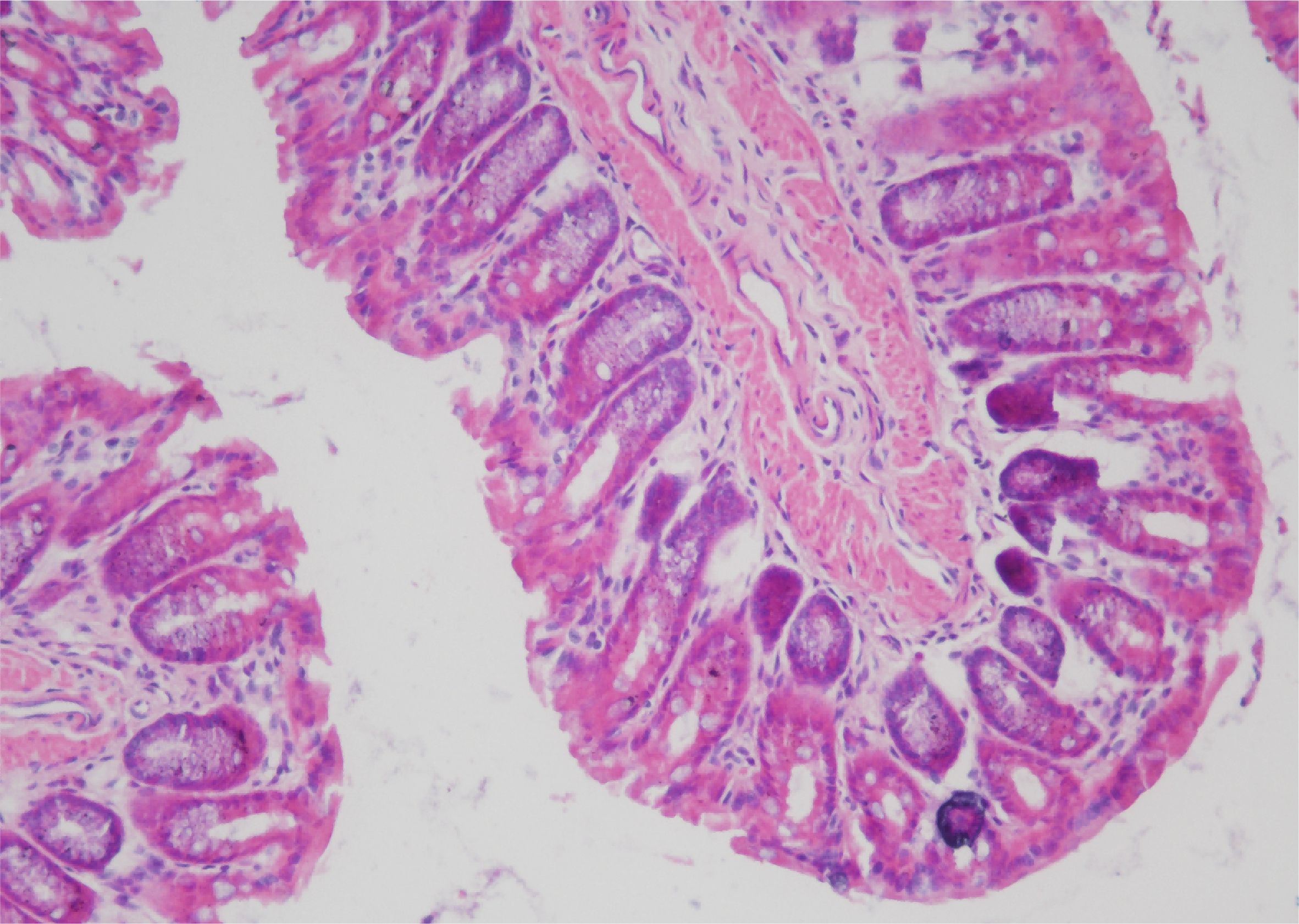

Supplement: Supplementary file 14 — EV and Appendix Figures Source Data [file 44319_2024_276_MOESM14_ESM.zip › Appendix Fig. S2/AFS2B/200×/Yod1++/3.png]

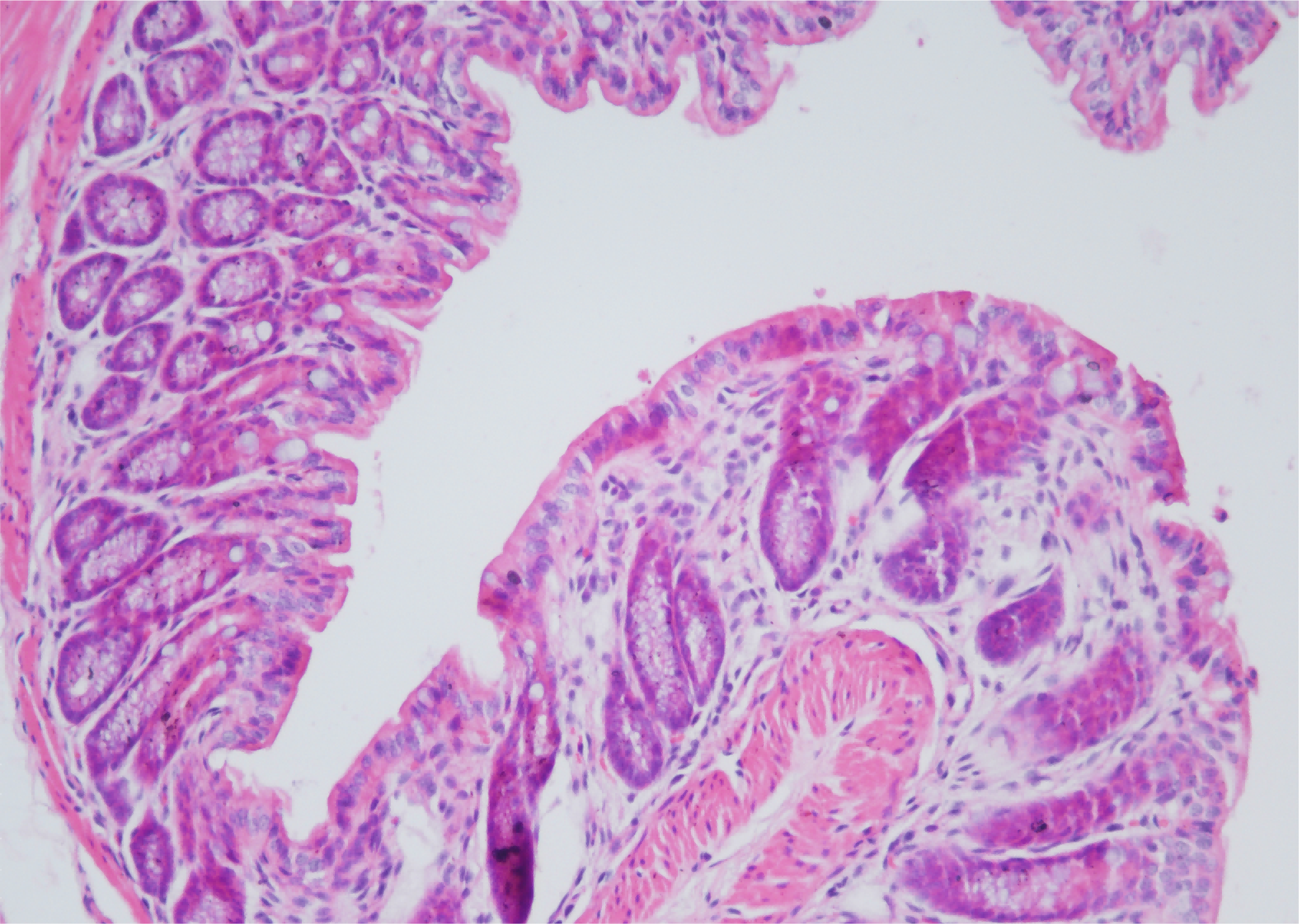

Supplement: Supplementary file 14 — EV and Appendix Figures Source Data [file 44319_2024_276_MOESM14_ESM.zip › Appendix Fig. S2/AFS2B/200×/Yod1++/4.png]

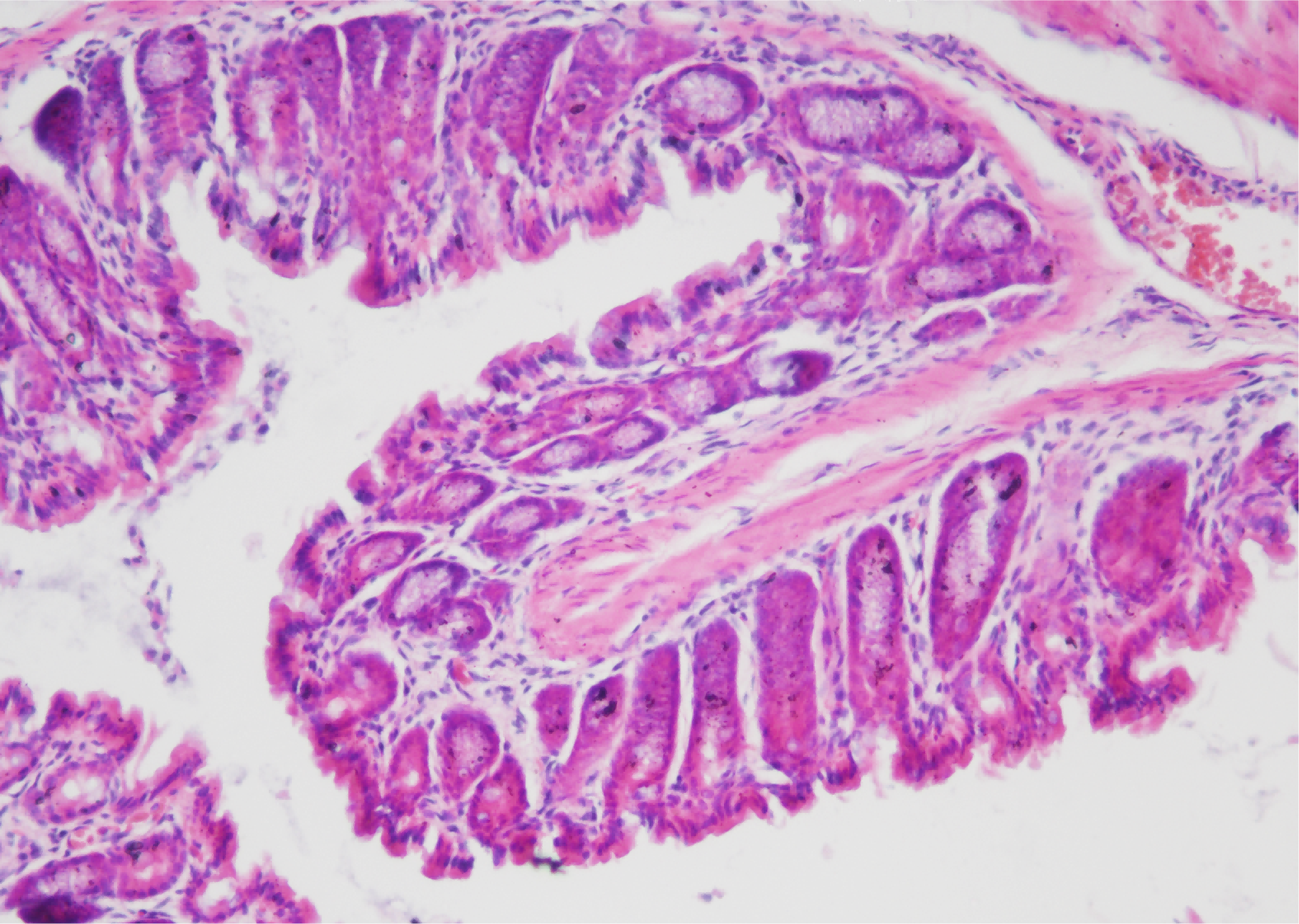

Supplement: Supplementary file 14 — EV and Appendix Figures Source Data [file 44319_2024_276_MOESM14_ESM.zip › Appendix Fig. S2/AFS2B/200×/Yod1++/5.png]

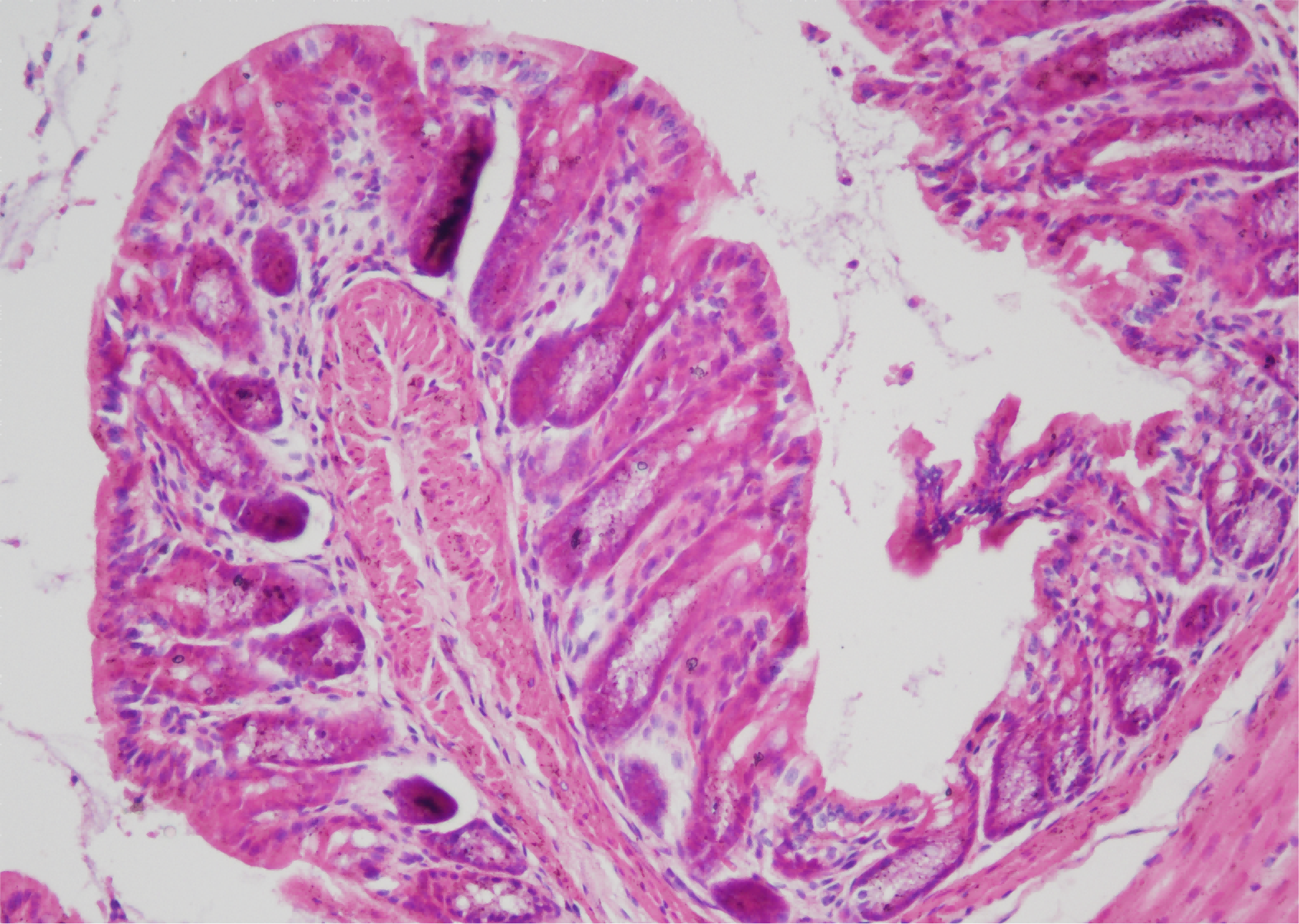

Supplement: Supplementary file 14 — EV and Appendix Figures Source Data [file 44319_2024_276_MOESM14_ESM.zip › Appendix Fig. S2/AFS2B/200×/Yod1++/6.png]

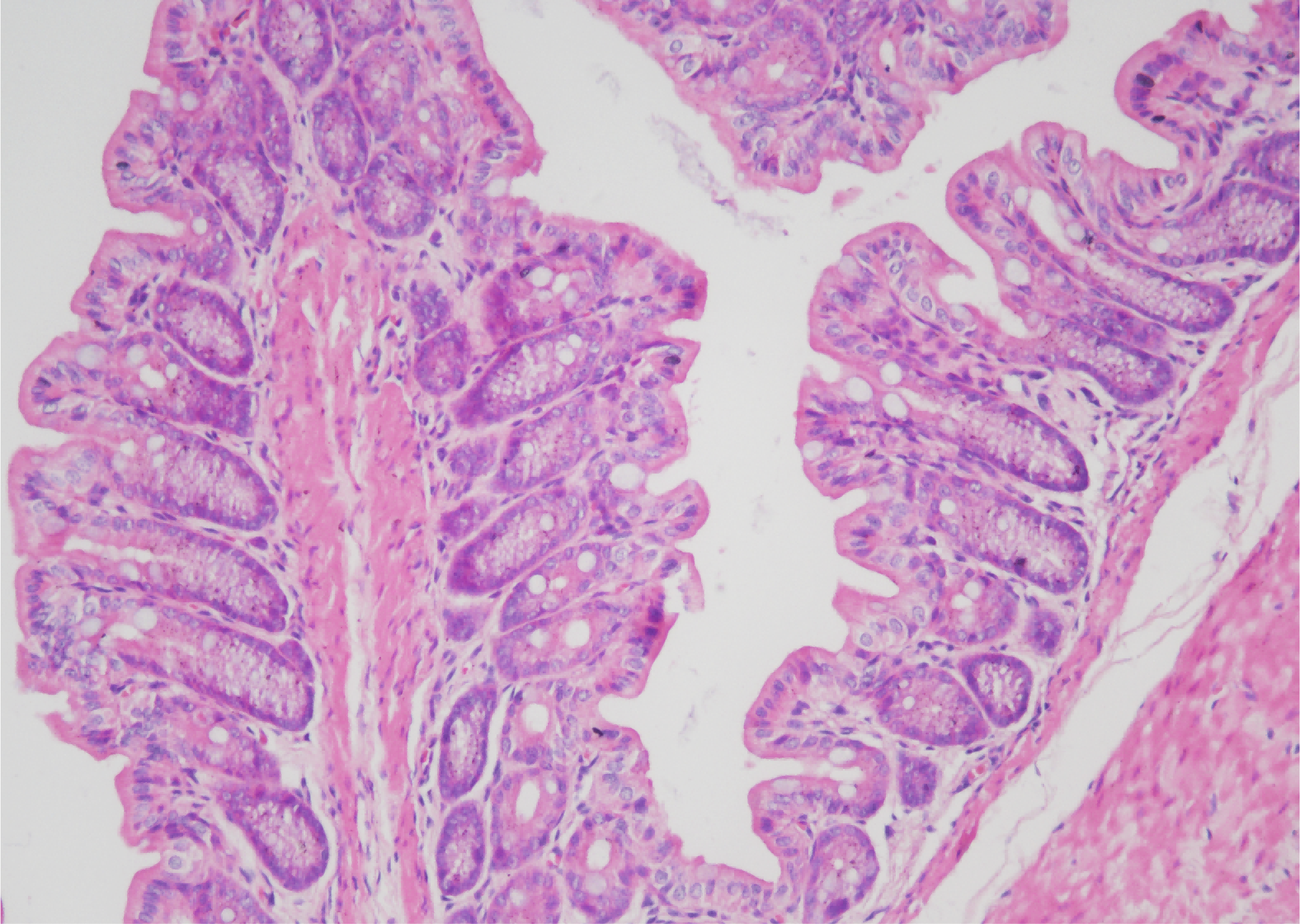

Supplement: Supplementary file 14 — EV and Appendix Figures Source Data [file 44319_2024_276_MOESM14_ESM.zip › Appendix Fig. S2/AFS2B/200×/Yod1++/7.png]

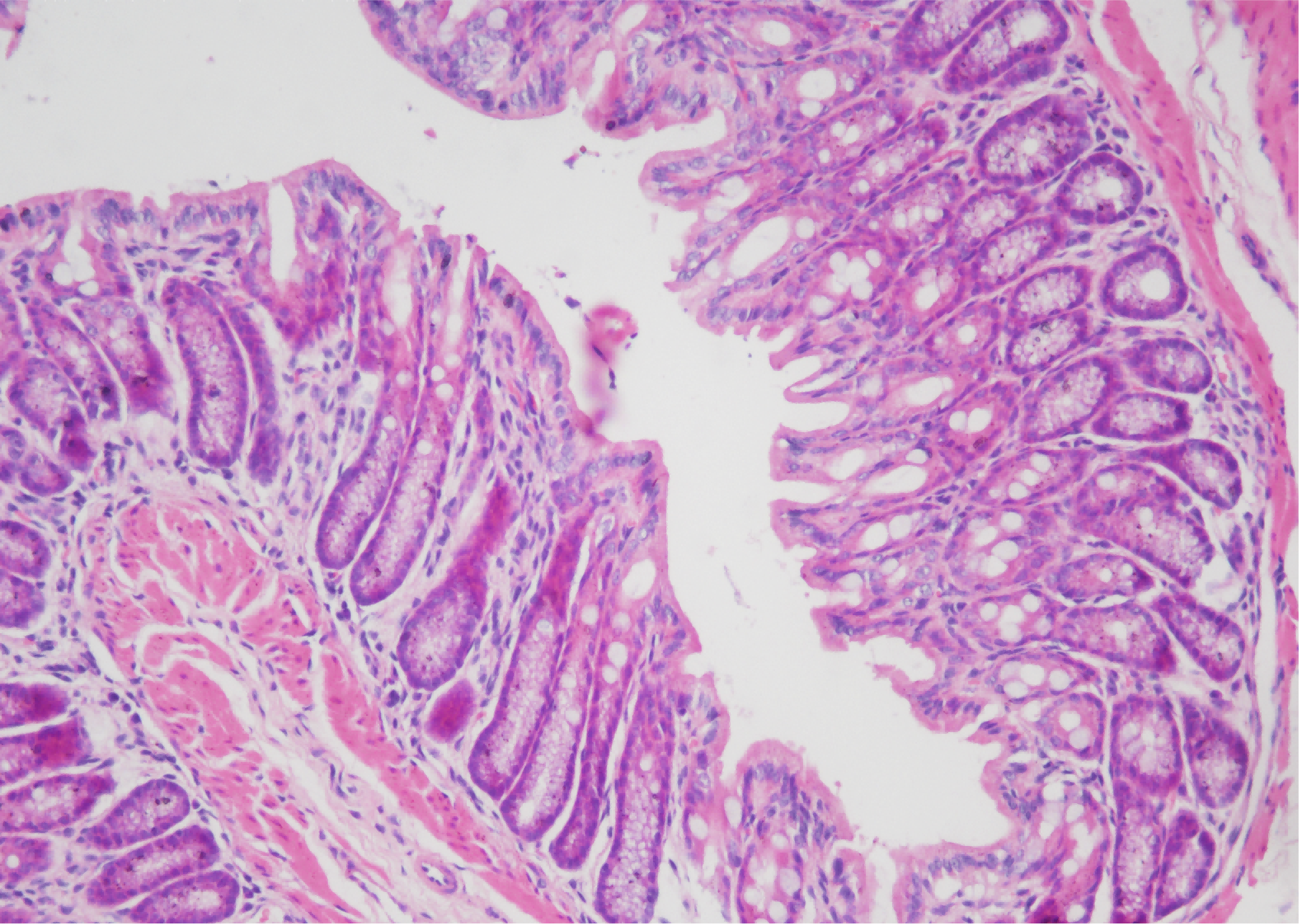

Supplement: Supplementary file 14 — EV and Appendix Figures Source Data [file 44319_2024_276_MOESM14_ESM.zip › Appendix Fig. S2/AFS2B/200×/Yod1--/1.png]

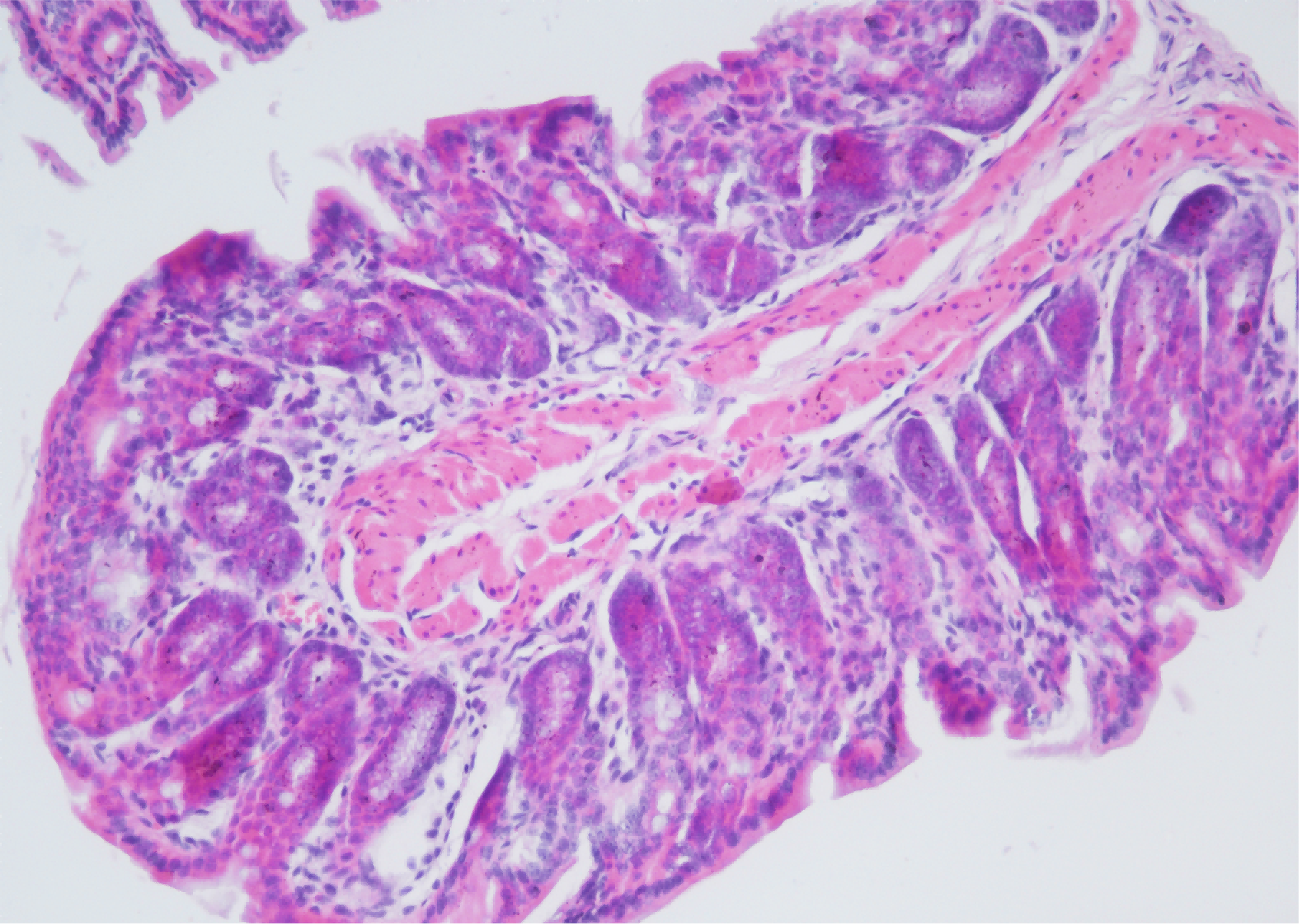

Supplement: Supplementary file 14 — EV and Appendix Figures Source Data [file 44319_2024_276_MOESM14_ESM.zip › Appendix Fig. S2/AFS2B/200×/Yod1--/2.png]

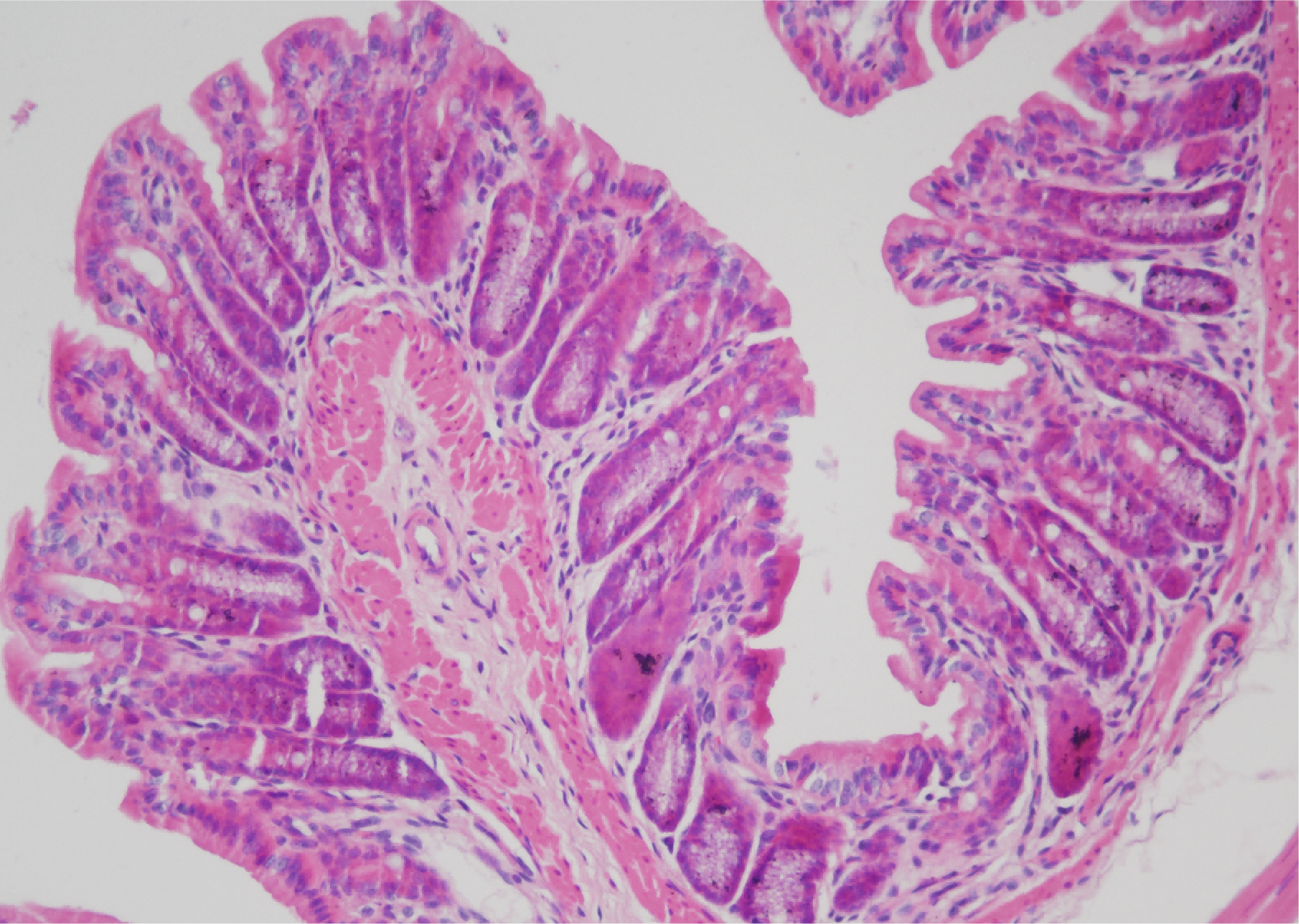

Supplement: Supplementary file 14 — EV and Appendix Figures Source Data [file 44319_2024_276_MOESM14_ESM.zip › Appendix Fig. S2/AFS2B/200×/Yod1--/3.png]

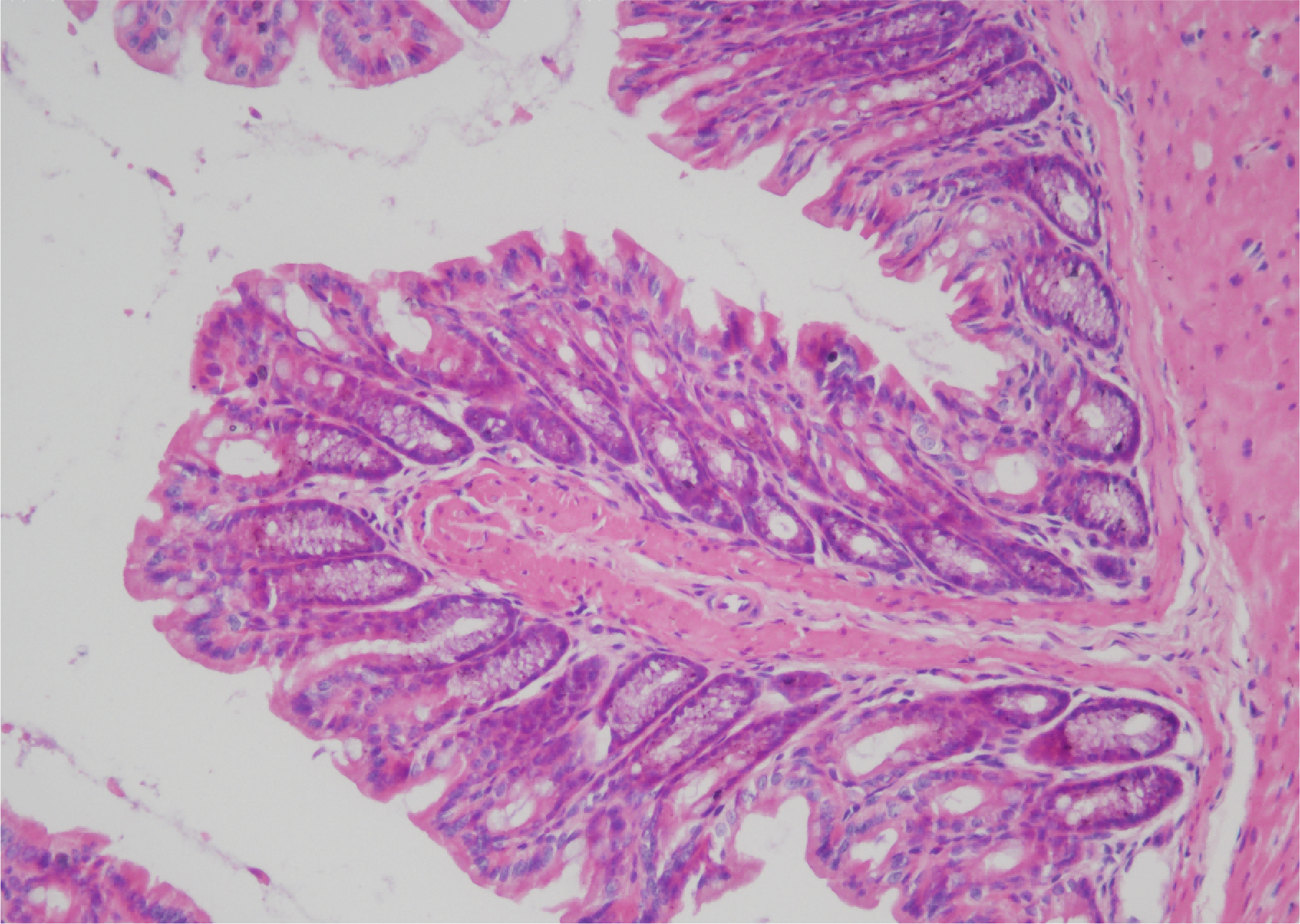

Supplement: Supplementary file 14 — EV and Appendix Figures Source Data [file 44319_2024_276_MOESM14_ESM.zip › Appendix Fig. S2/AFS2B/200×/Yod1--/4.png]

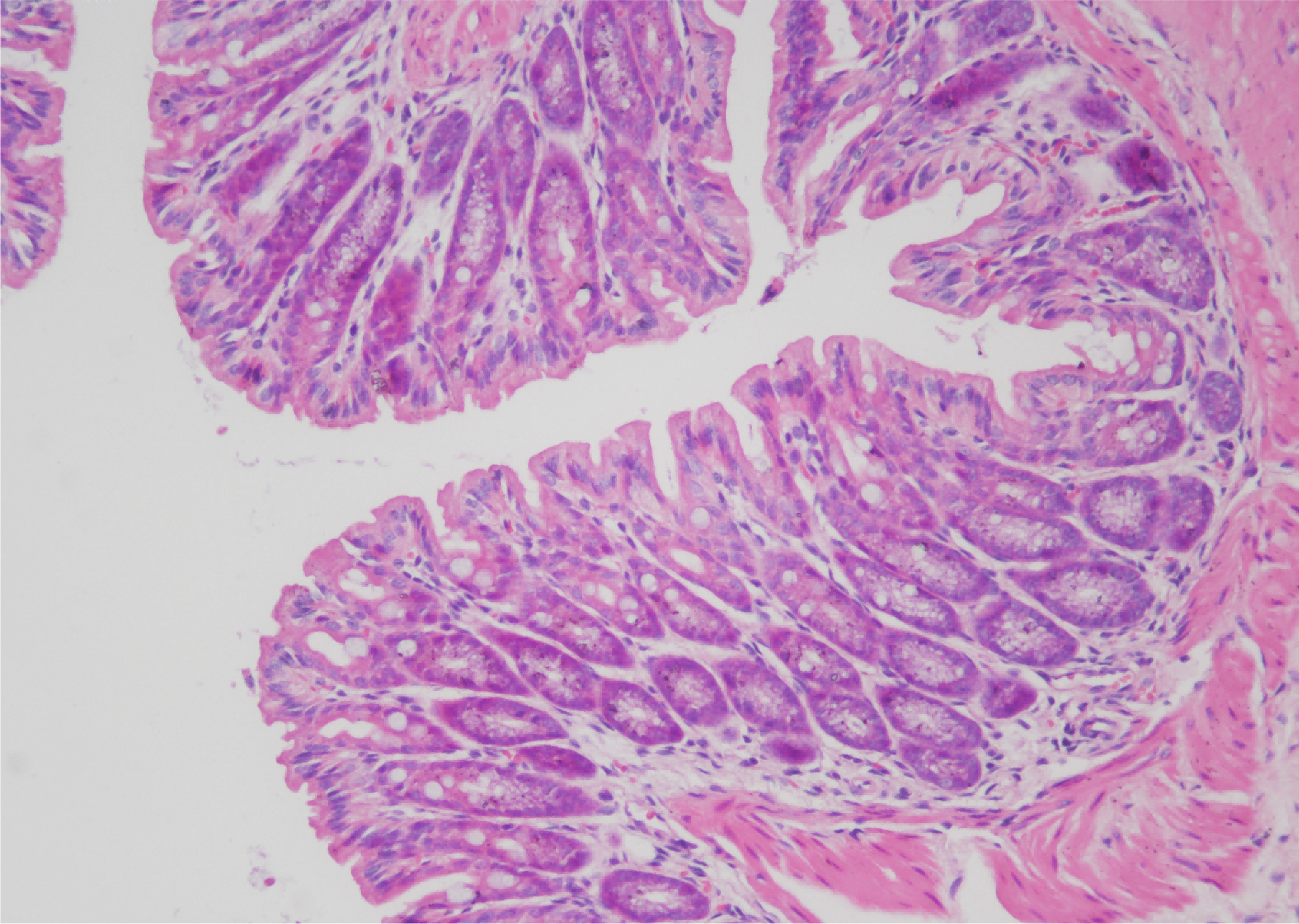

Supplement: Supplementary file 14 — EV and Appendix Figures Source Data [file 44319_2024_276_MOESM14_ESM.zip › Appendix Fig. S2/AFS2B/200×/Yod1--/5.png]

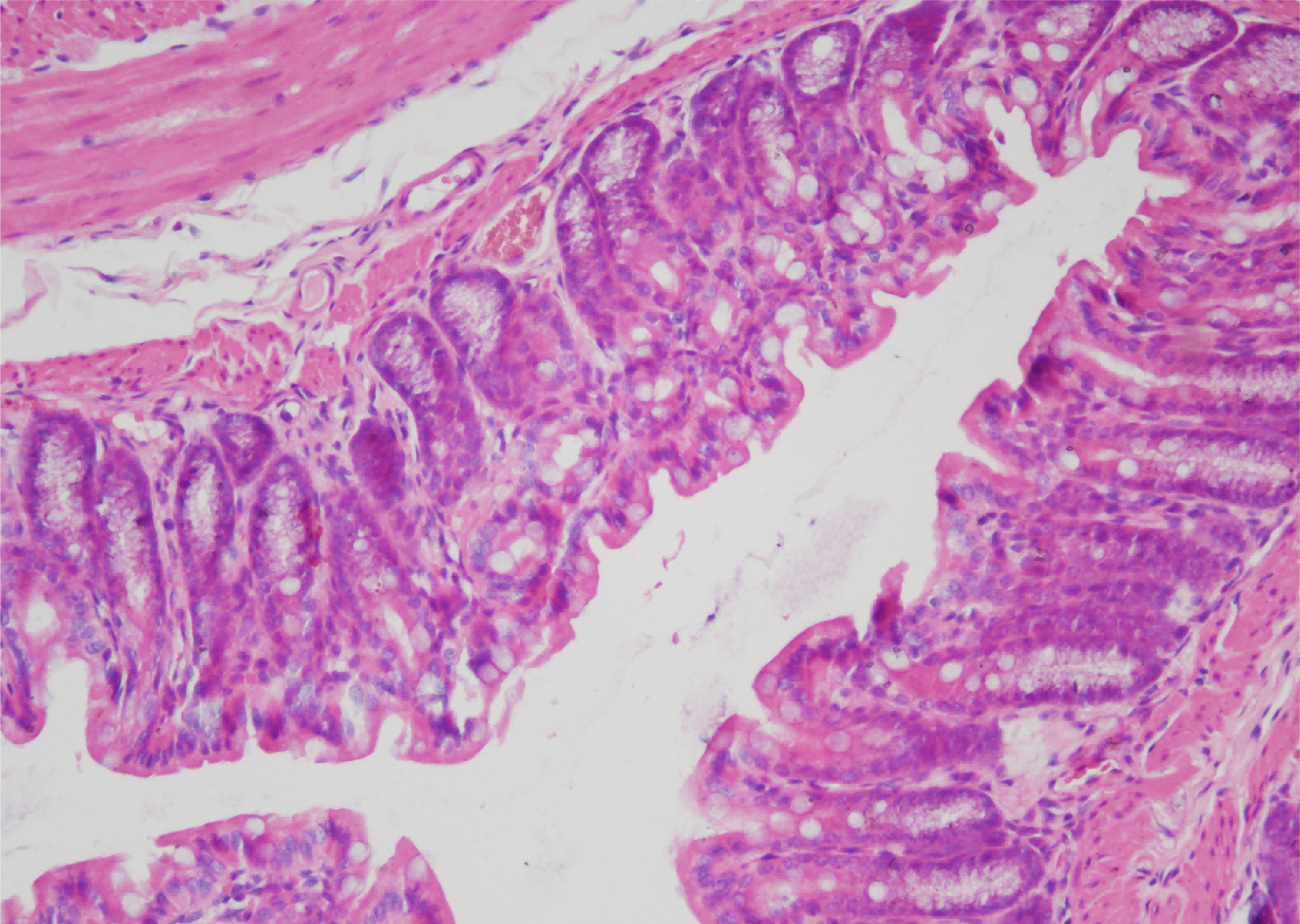

Supplement: Supplementary file 14 — EV and Appendix Figures Source Data [file 44319_2024_276_MOESM14_ESM.zip › Appendix Fig. S2/AFS2B/200×/Yod1--/6.png]

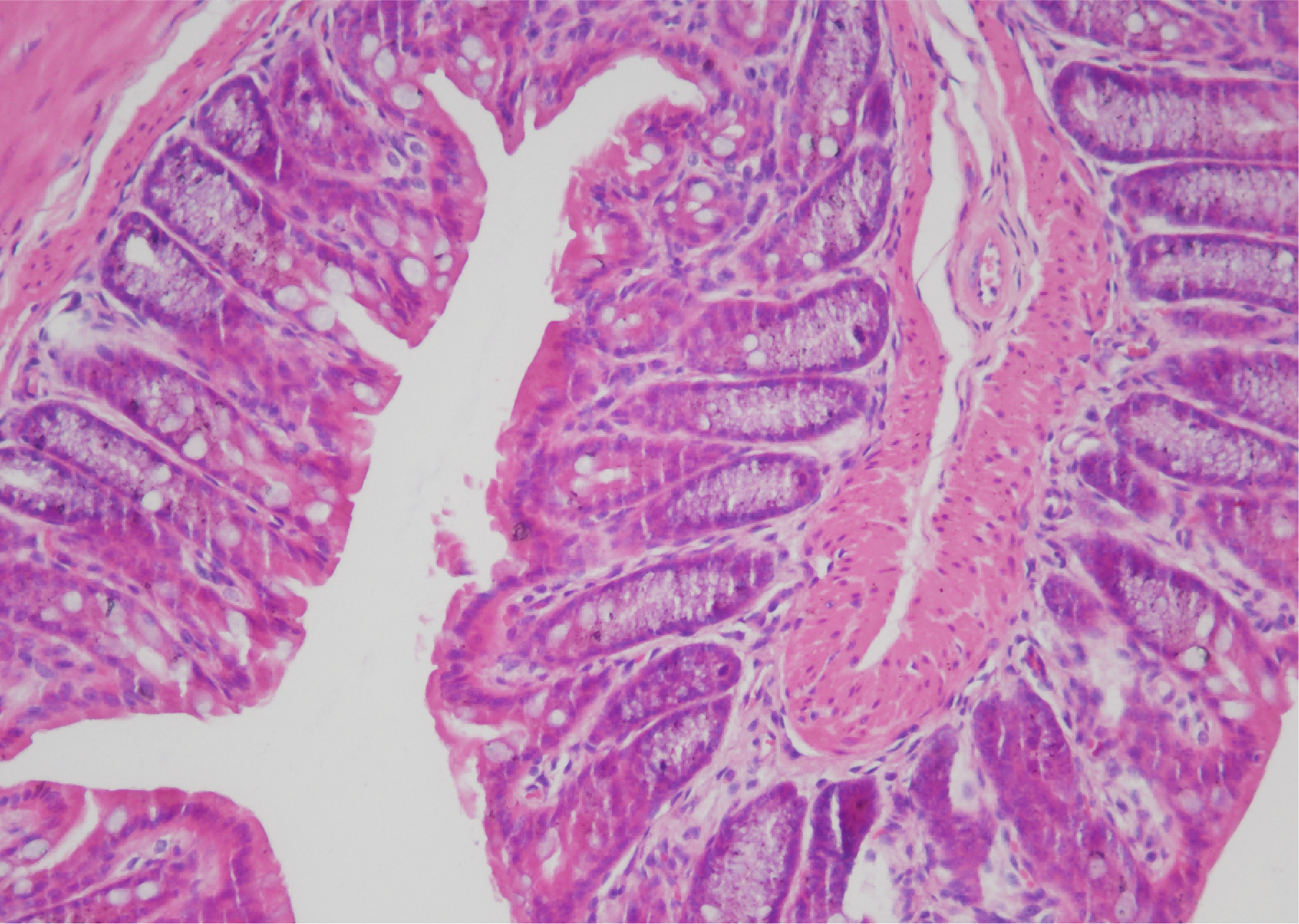

Supplement: Supplementary file 14 — EV and Appendix Figures Source Data [file 44319_2024_276_MOESM14_ESM.zip › Appendix Fig. S2/AFS2B/200×/Yod1--/7.png]

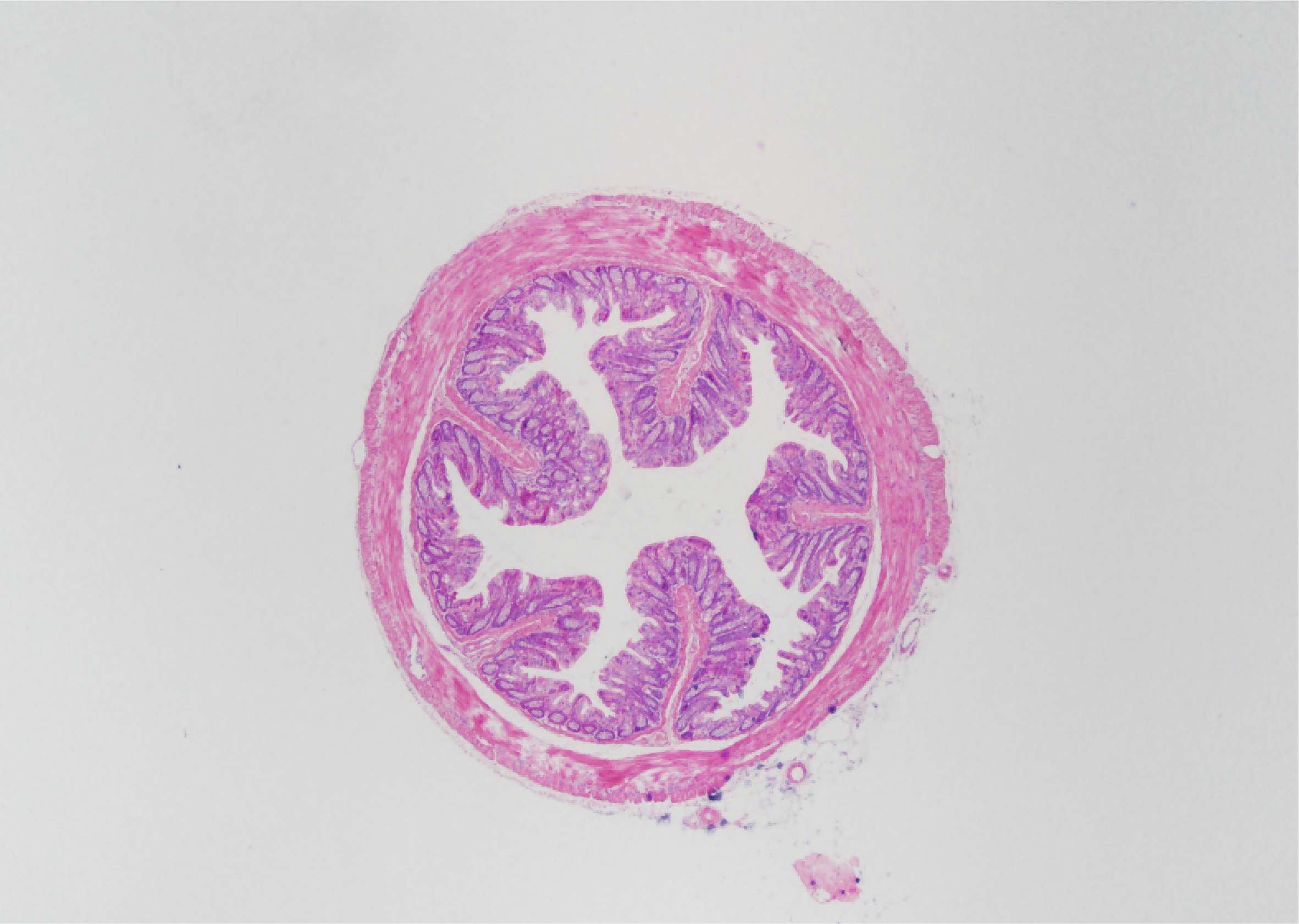

Supplement: Supplementary file 14 — EV and Appendix Figures Source Data [file 44319_2024_276_MOESM14_ESM.zip › Appendix Fig. S2/AFS2B/40×/Yod1++/1.png]

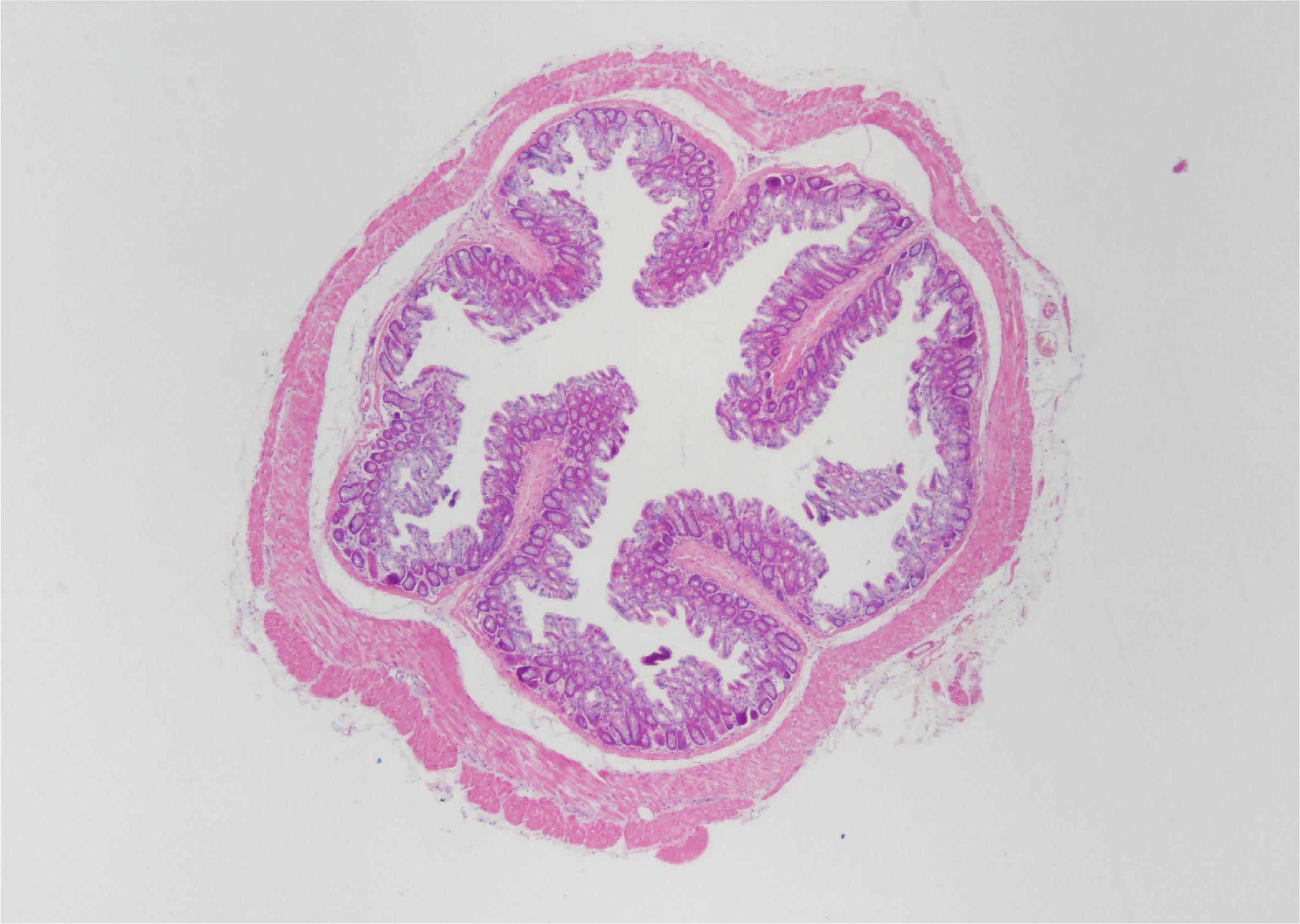

Supplement: Supplementary file 14 — EV and Appendix Figures Source Data [file 44319_2024_276_MOESM14_ESM.zip › Appendix Fig. S2/AFS2B/40×/Yod1++/2.png]

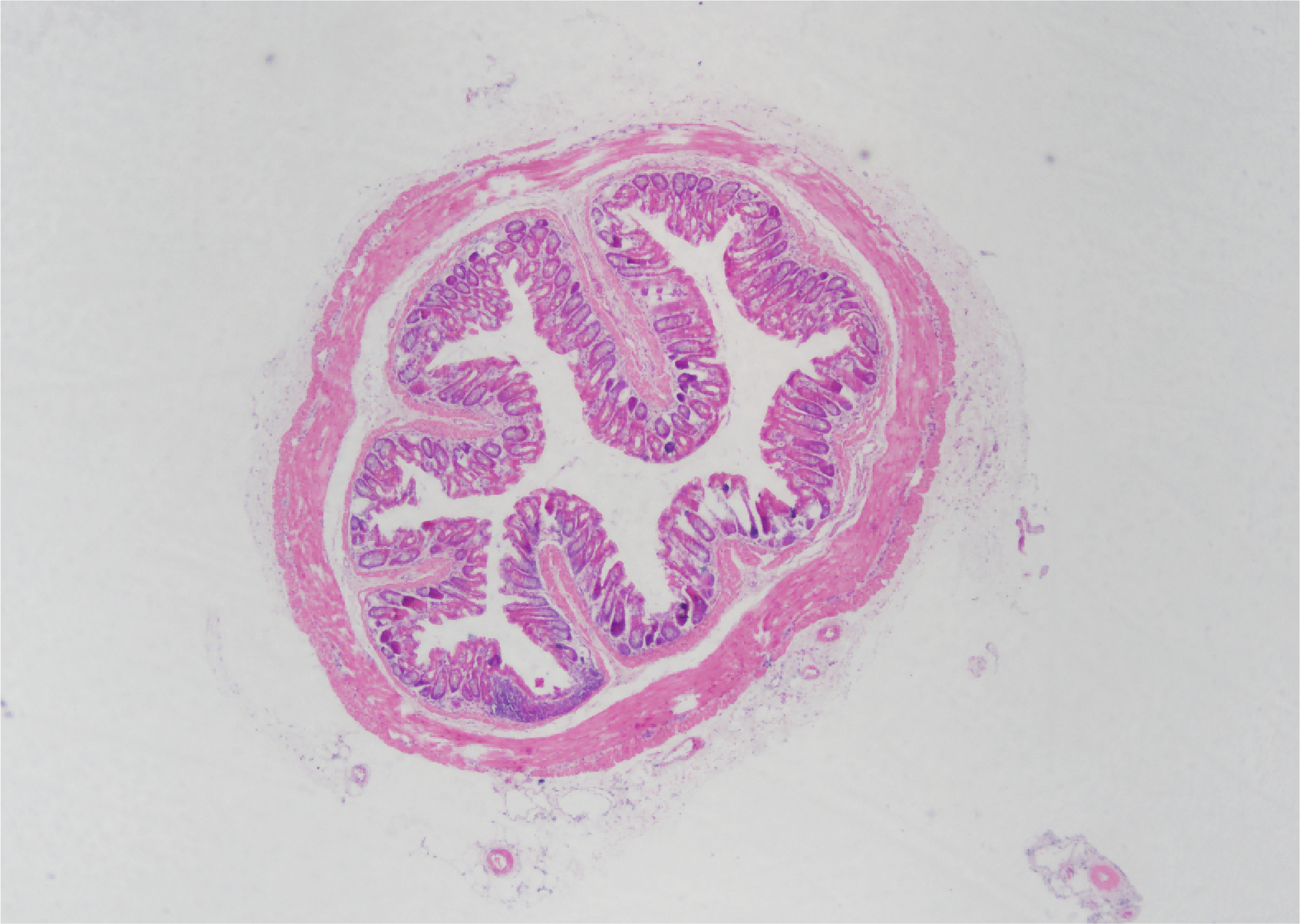

Supplement: Supplementary file 14 — EV and Appendix Figures Source Data [file 44319_2024_276_MOESM14_ESM.zip › Appendix Fig. S2/AFS2B/40×/Yod1++/3.png]

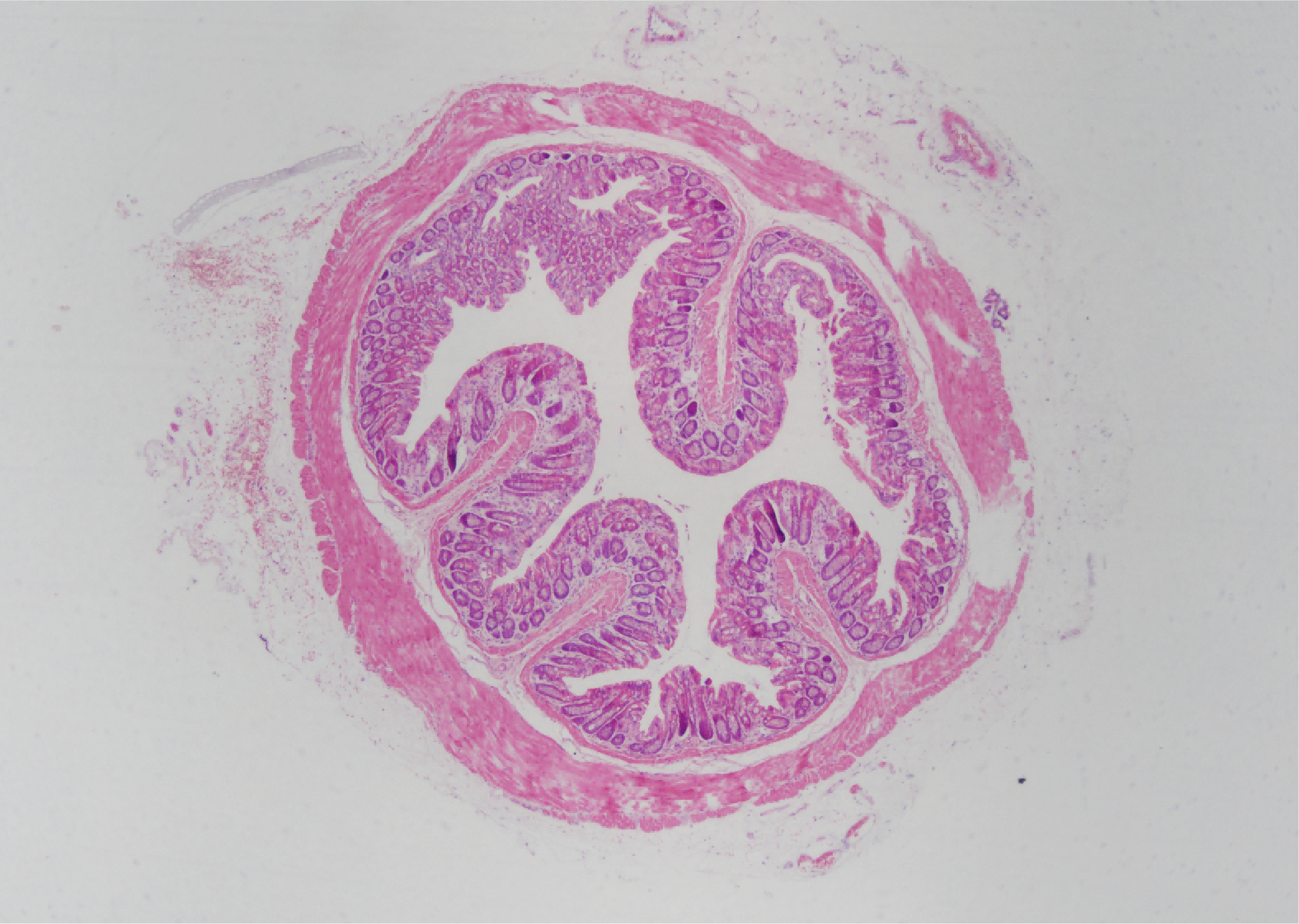

Supplement: Supplementary file 14 — EV and Appendix Figures Source Data [file 44319_2024_276_MOESM14_ESM.zip › Appendix Fig. S2/AFS2B/40×/Yod1++/4.png]

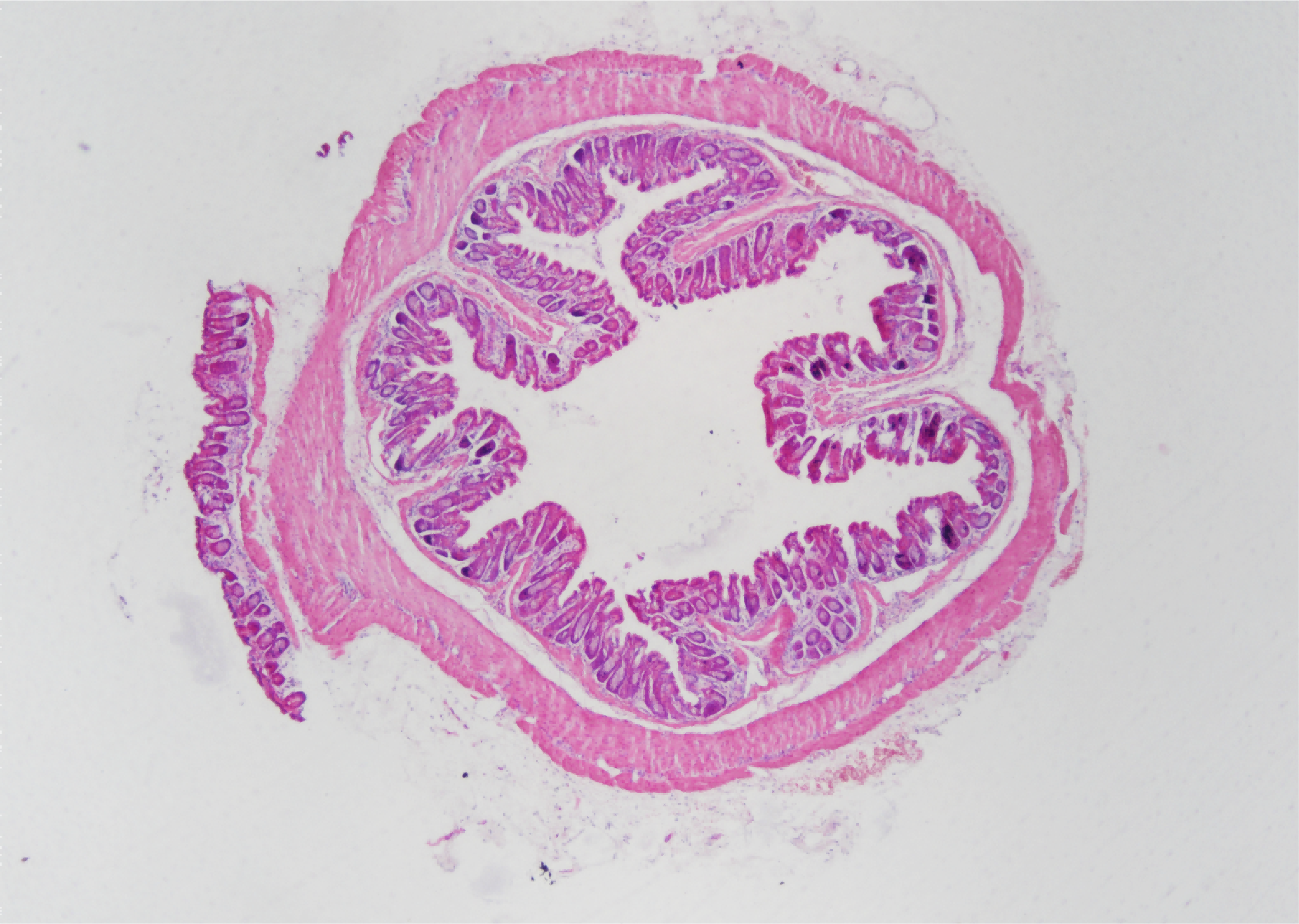

Supplement: Supplementary file 14 — EV and Appendix Figures Source Data [file 44319_2024_276_MOESM14_ESM.zip › Appendix Fig. S2/AFS2B/40×/Yod1++/5.png]

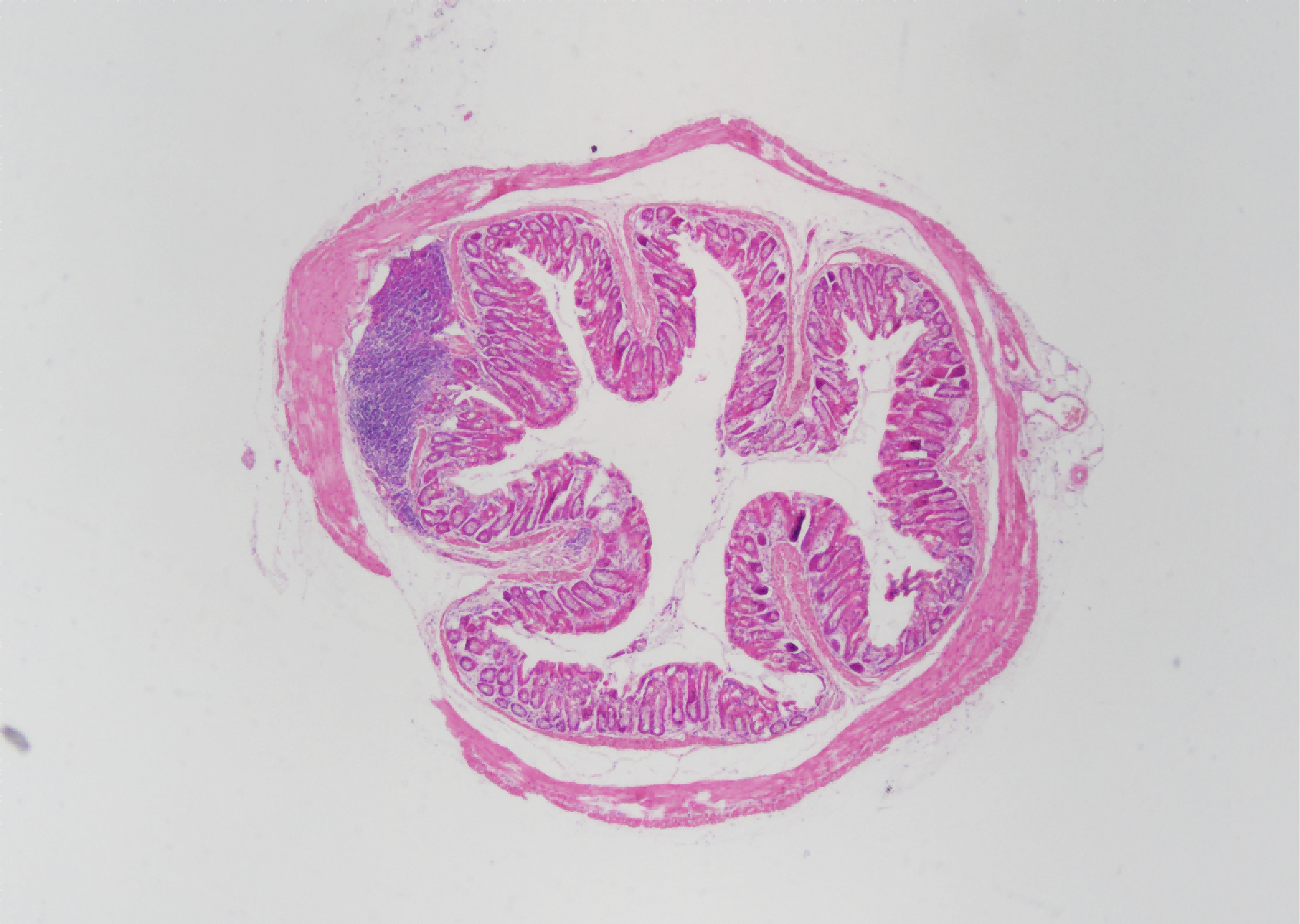

Supplement: Supplementary file 14 — EV and Appendix Figures Source Data [file 44319_2024_276_MOESM14_ESM.zip › Appendix Fig. S2/AFS2B/40×/Yod1++/6.png]

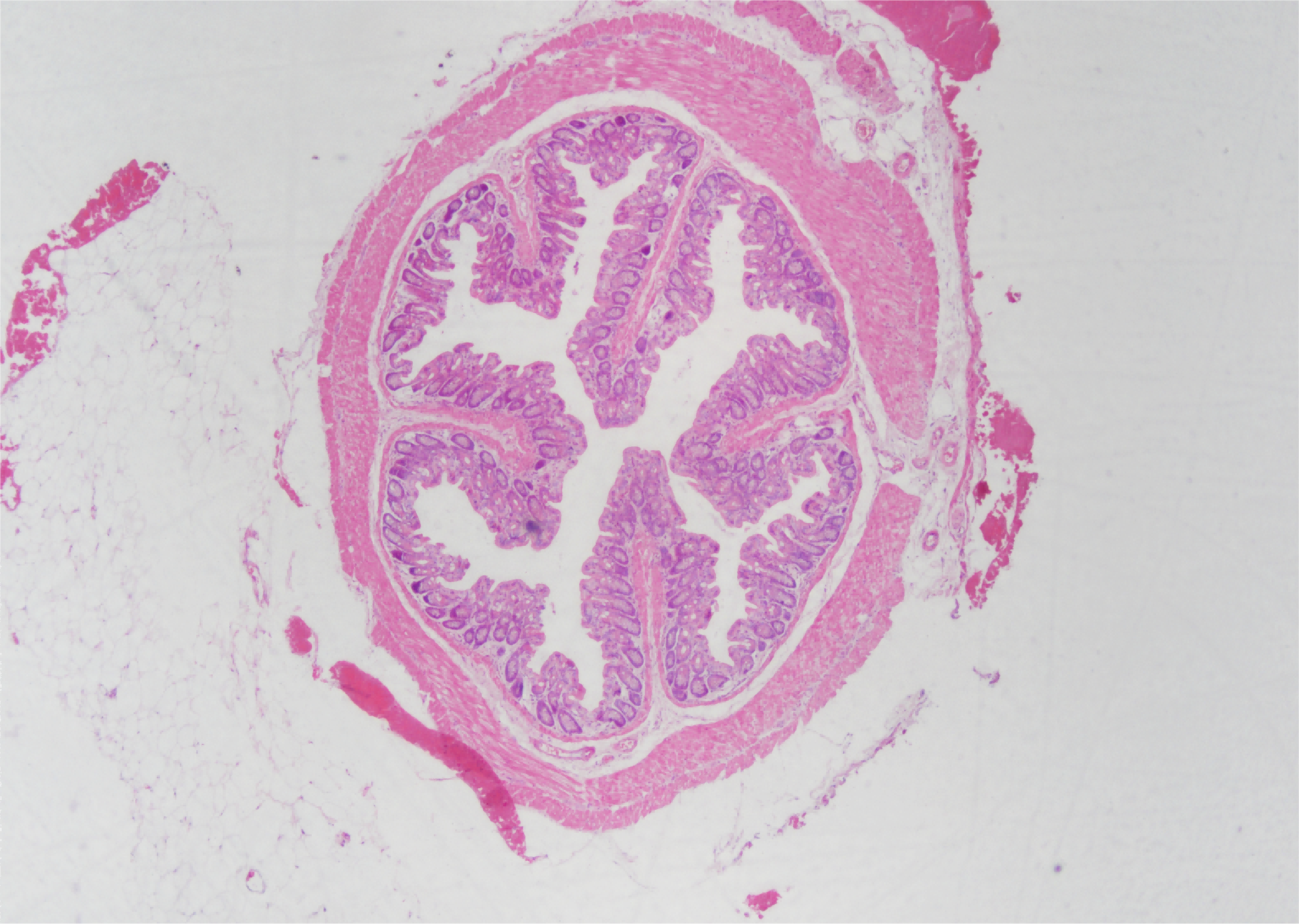

Supplement: Supplementary file 14 — EV and Appendix Figures Source Data [file 44319_2024_276_MOESM14_ESM.zip › Appendix Fig. S2/AFS2B/40×/Yod1++/7.png]

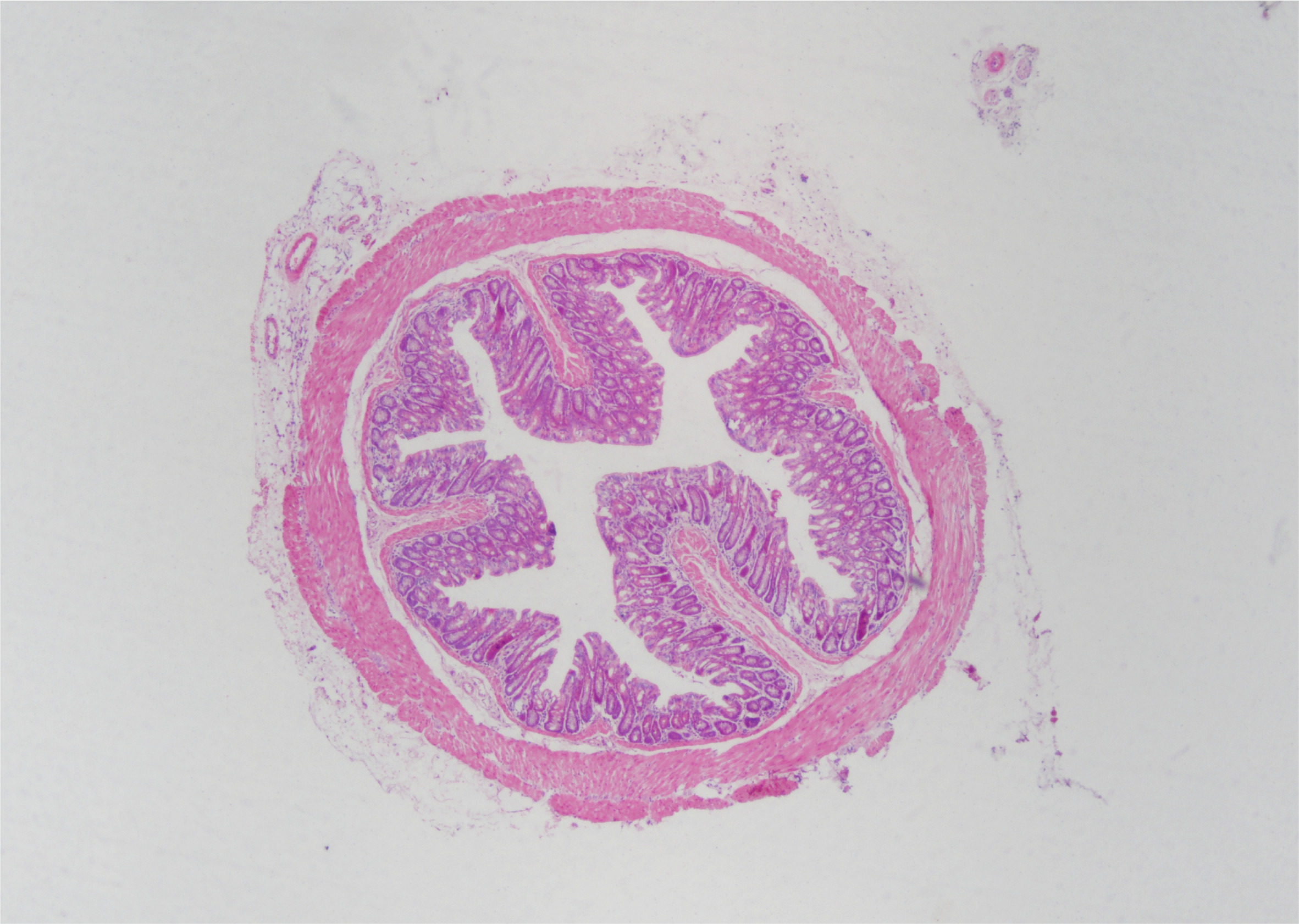

Supplement: Supplementary file 14 — EV and Appendix Figures Source Data [file 44319_2024_276_MOESM14_ESM.zip › Appendix Fig. S2/AFS2B/40×/Yod1--/1.png]

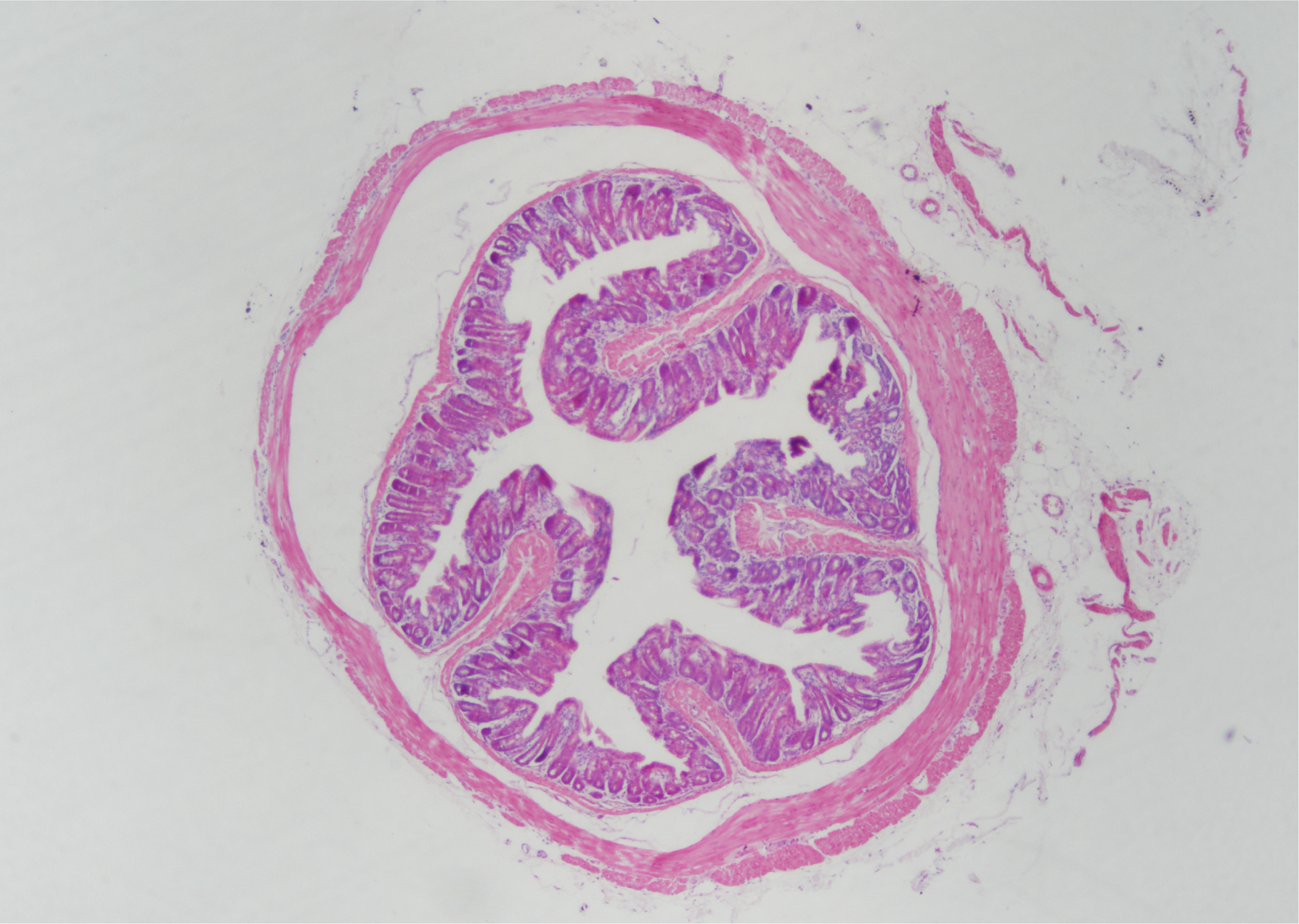

Supplement: Supplementary file 14 — EV and Appendix Figures Source Data [file 44319_2024_276_MOESM14_ESM.zip › Appendix Fig. S2/AFS2B/40×/Yod1--/2.png]

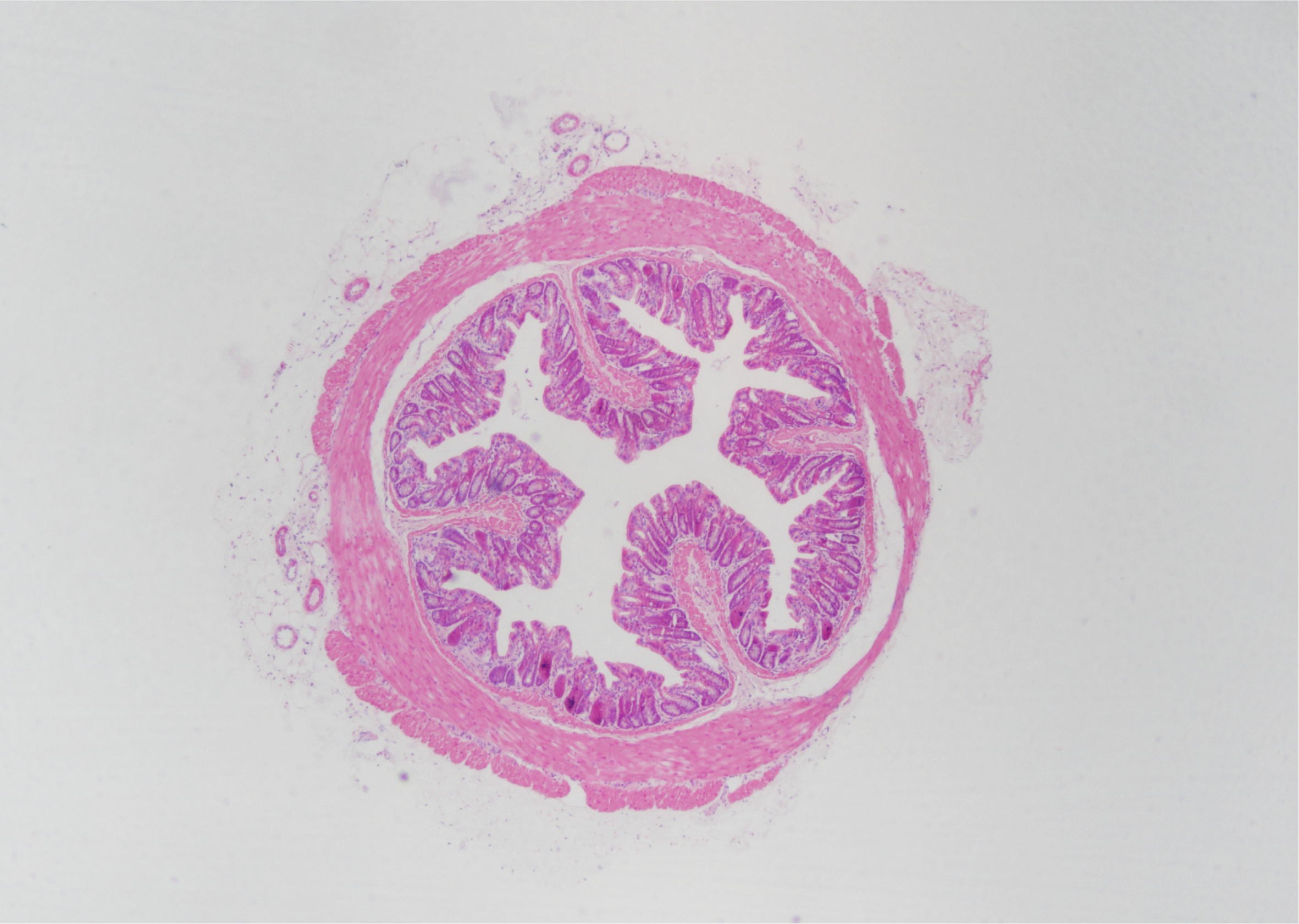

Supplement: Supplementary file 14 — EV and Appendix Figures Source Data [file 44319_2024_276_MOESM14_ESM.zip › Appendix Fig. S2/AFS2B/40×/Yod1--/3.png]

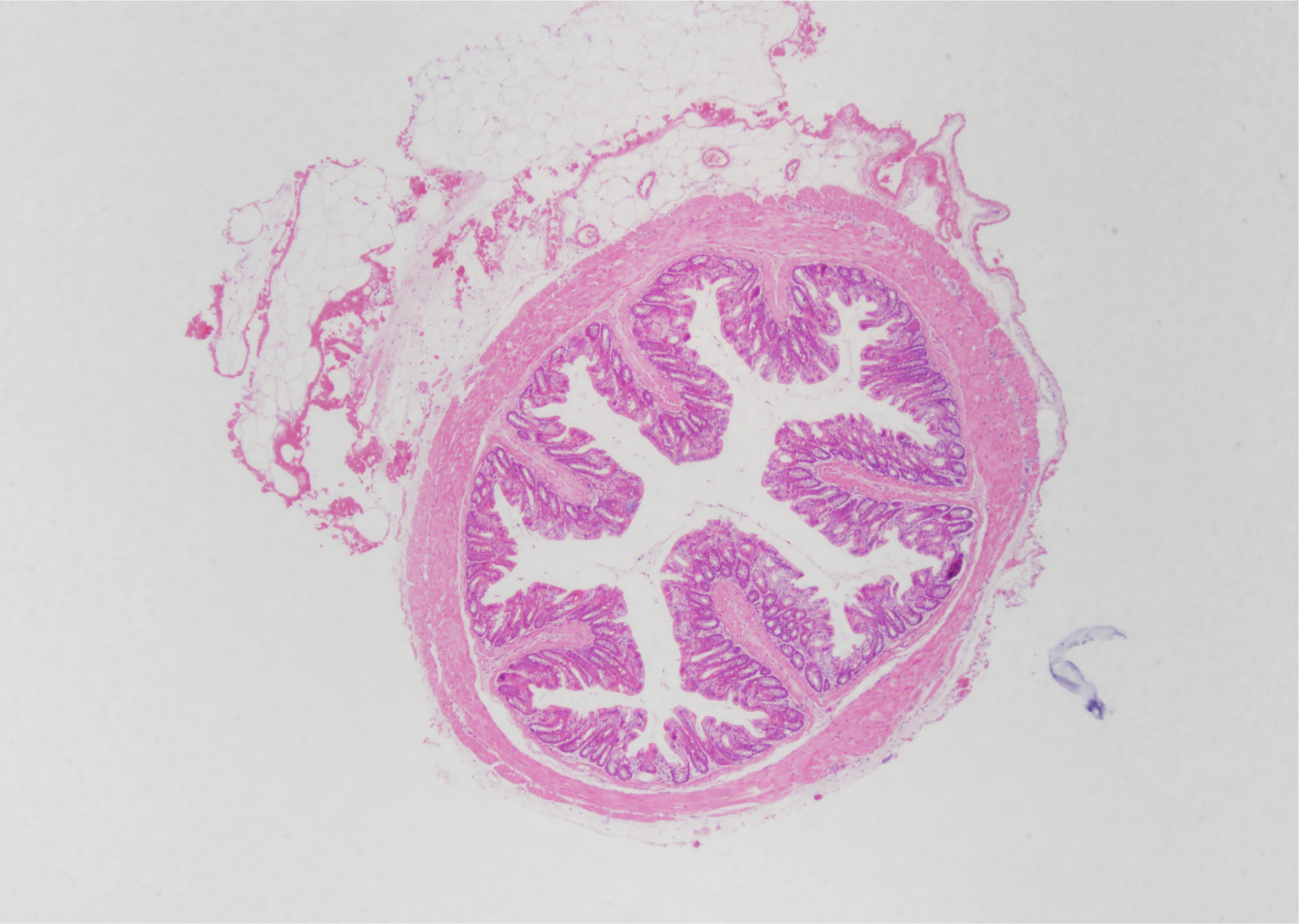

Supplement: Supplementary file 14 — EV and Appendix Figures Source Data [file 44319_2024_276_MOESM14_ESM.zip › Appendix Fig. S2/AFS2B/40×/Yod1--/4.png]

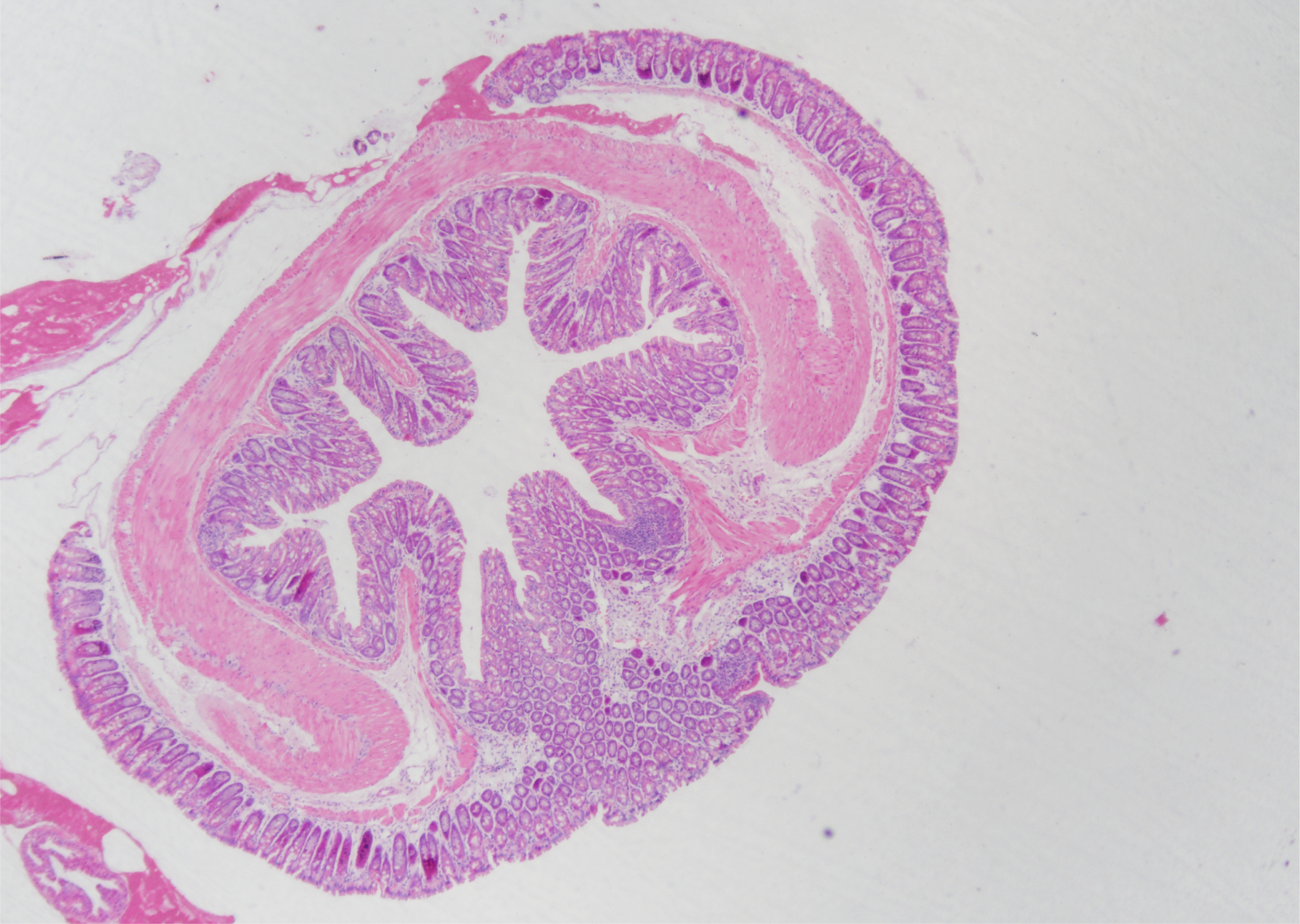

Supplement: Supplementary file 14 — EV and Appendix Figures Source Data [file 44319_2024_276_MOESM14_ESM.zip › Appendix Fig. S2/AFS2B/40×/Yod1--/5.png]

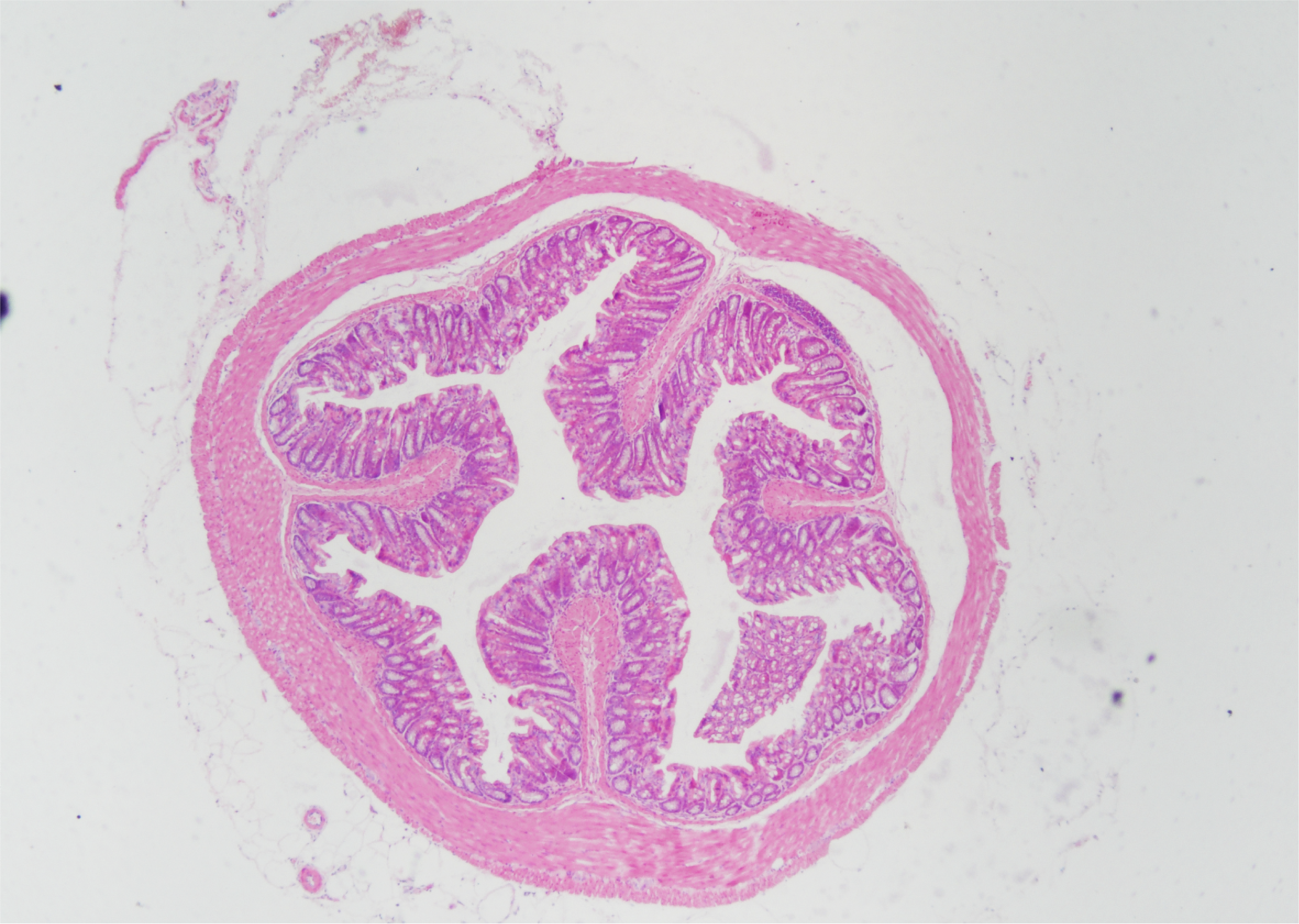

Supplement: Supplementary file 14 — EV and Appendix Figures Source Data [file 44319_2024_276_MOESM14_ESM.zip › Appendix Fig. S2/AFS2B/40×/Yod1--/6.png]

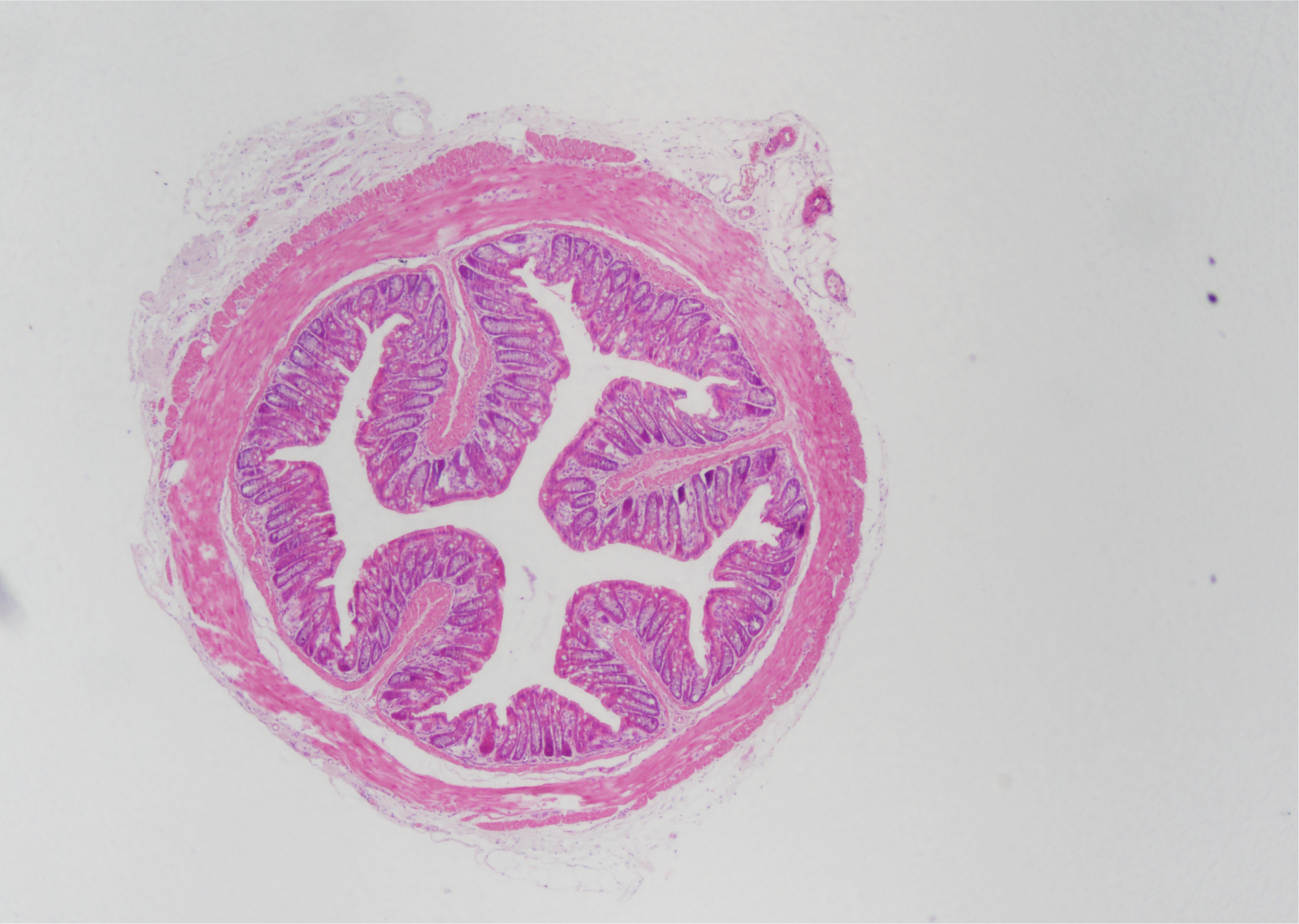

Supplement: Supplementary file 14 — EV and Appendix Figures Source Data [file 44319_2024_276_MOESM14_ESM.zip › Appendix Fig. S2/AFS2B/40×/Yod1--/7.png]

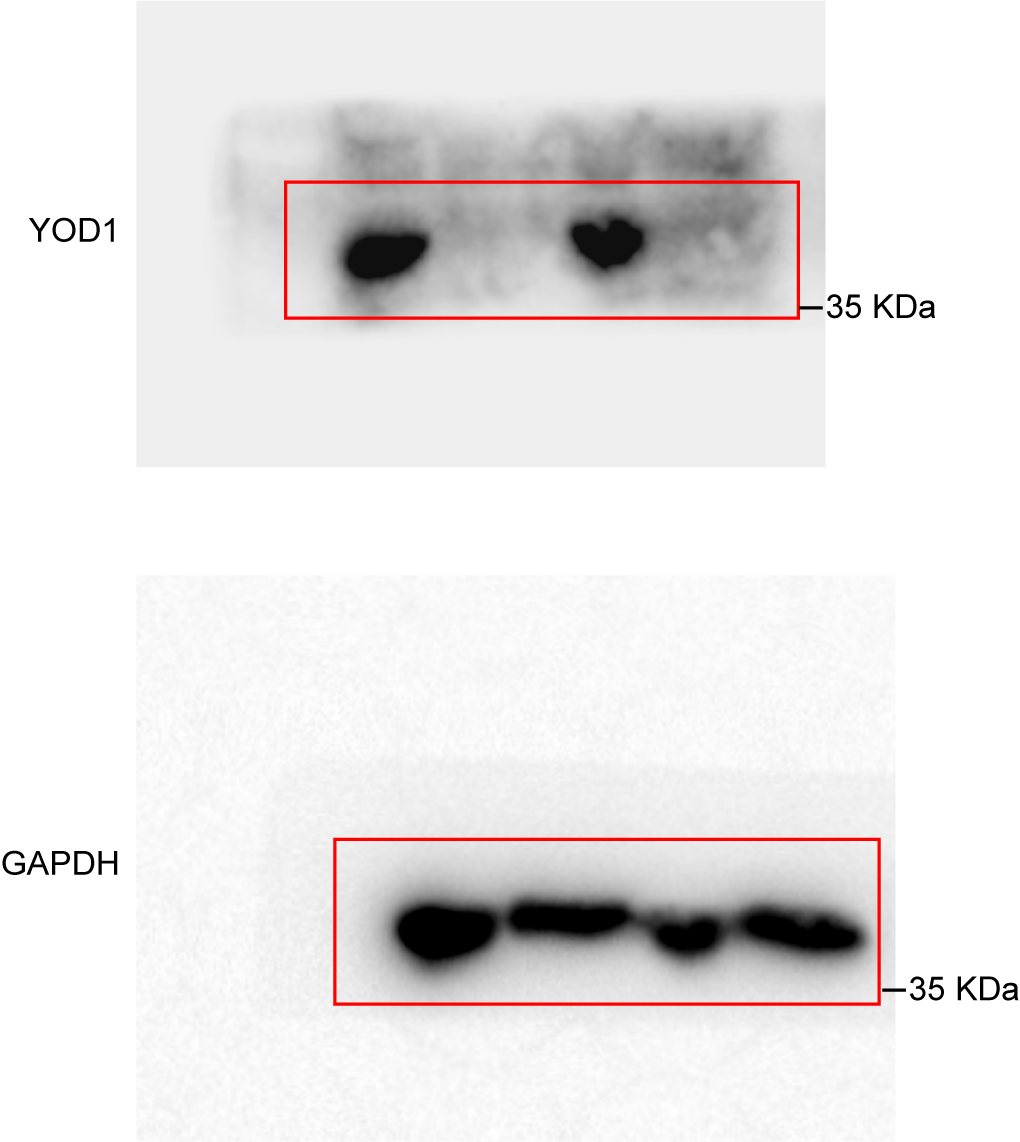

Supplement: Supplementary file 14 — EV and Appendix Figures Source Data [file 44319_2024_276_MOESM14_ESM.zip › Appendix Fig. S9/AFS9A.tif]

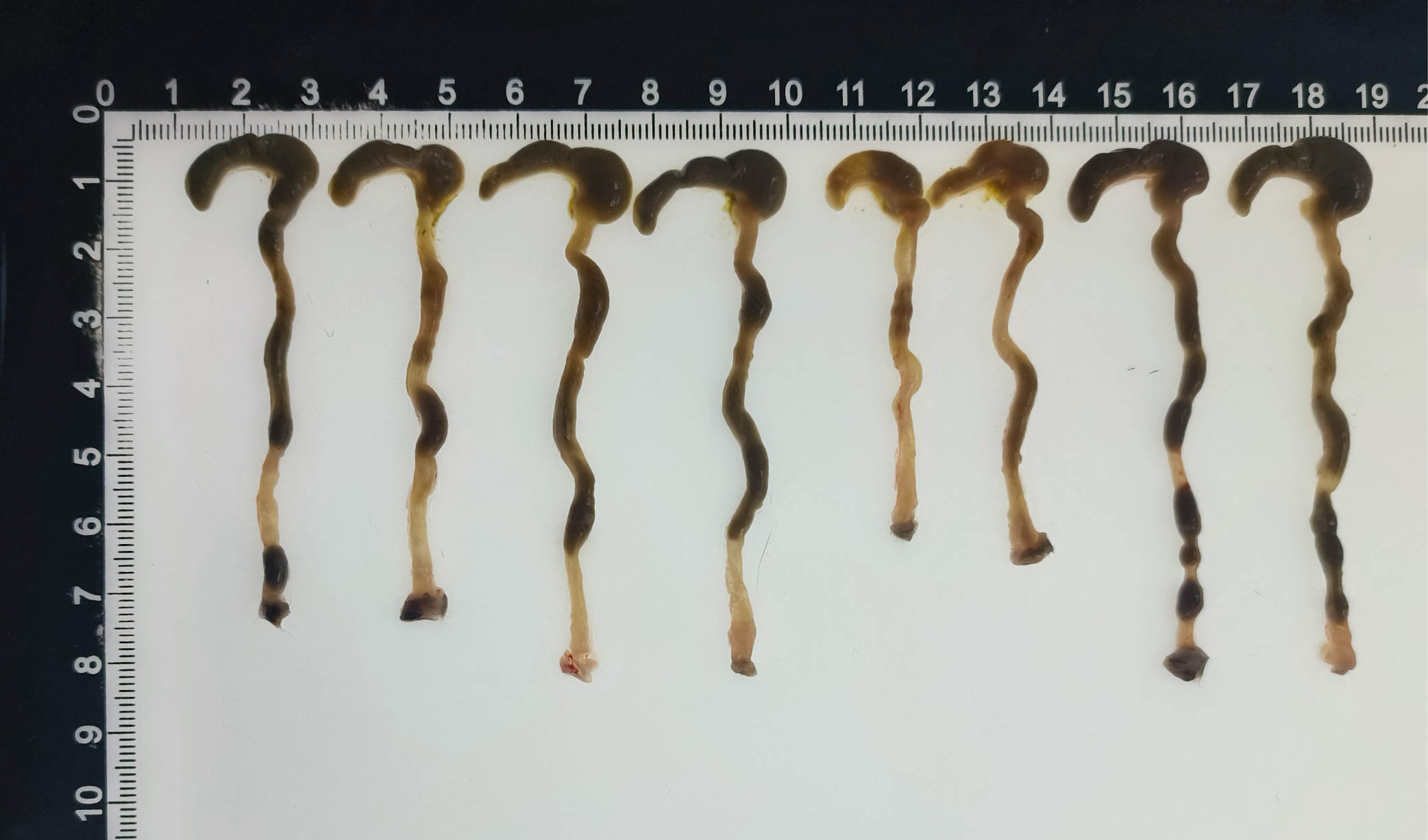

Supplement: Supplementary file 14 — EV and Appendix Figures Source Data [file 44319_2024_276_MOESM14_ESM.zip › Fig EV1/EV1-E/EV1-E.png]

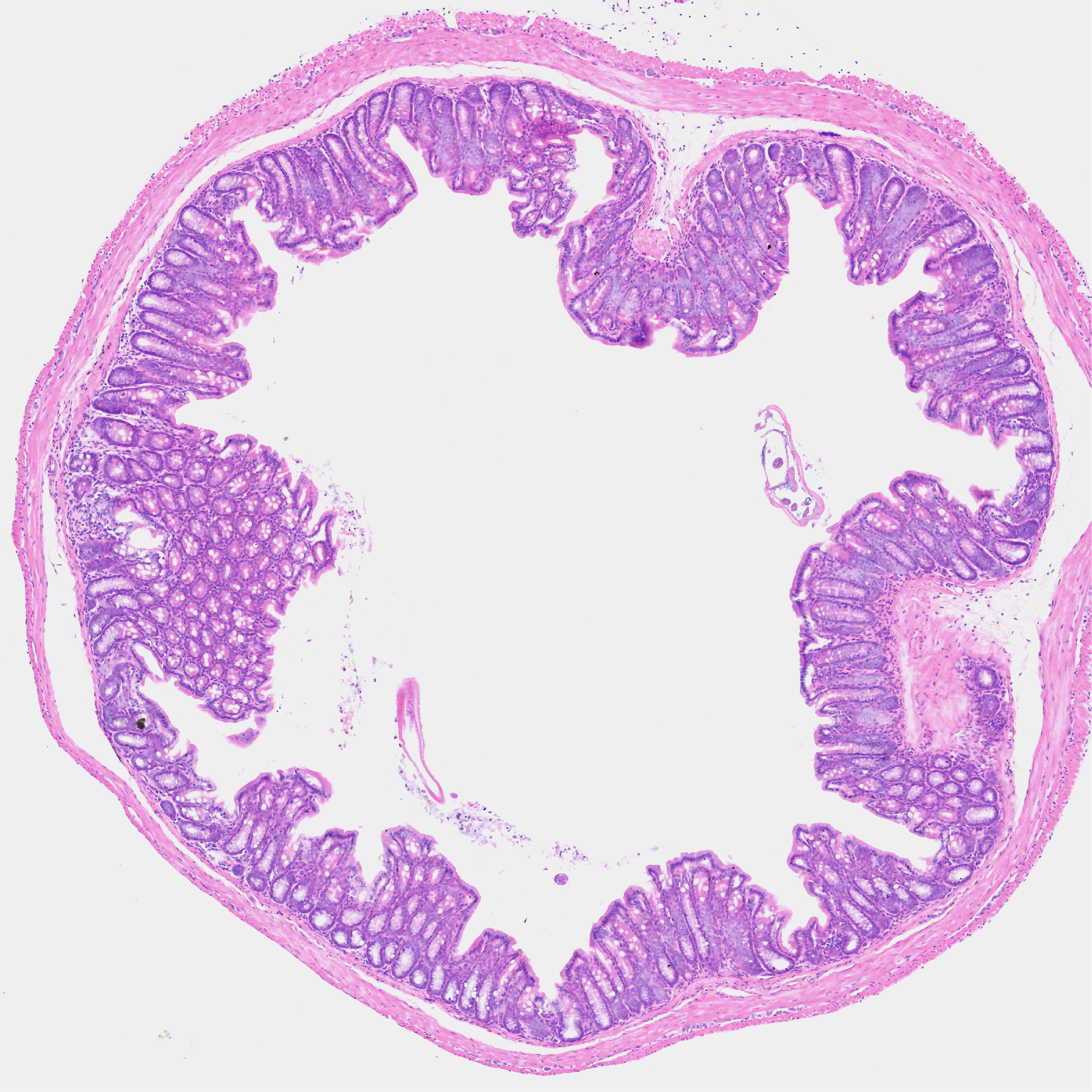

Supplement: Supplementary file 14 — EV and Appendix Figures Source Data [file 44319_2024_276_MOESM14_ESM.zip › Fig EV1/EV1-I/HE staining/Yod1++_LPS_overall image.png]

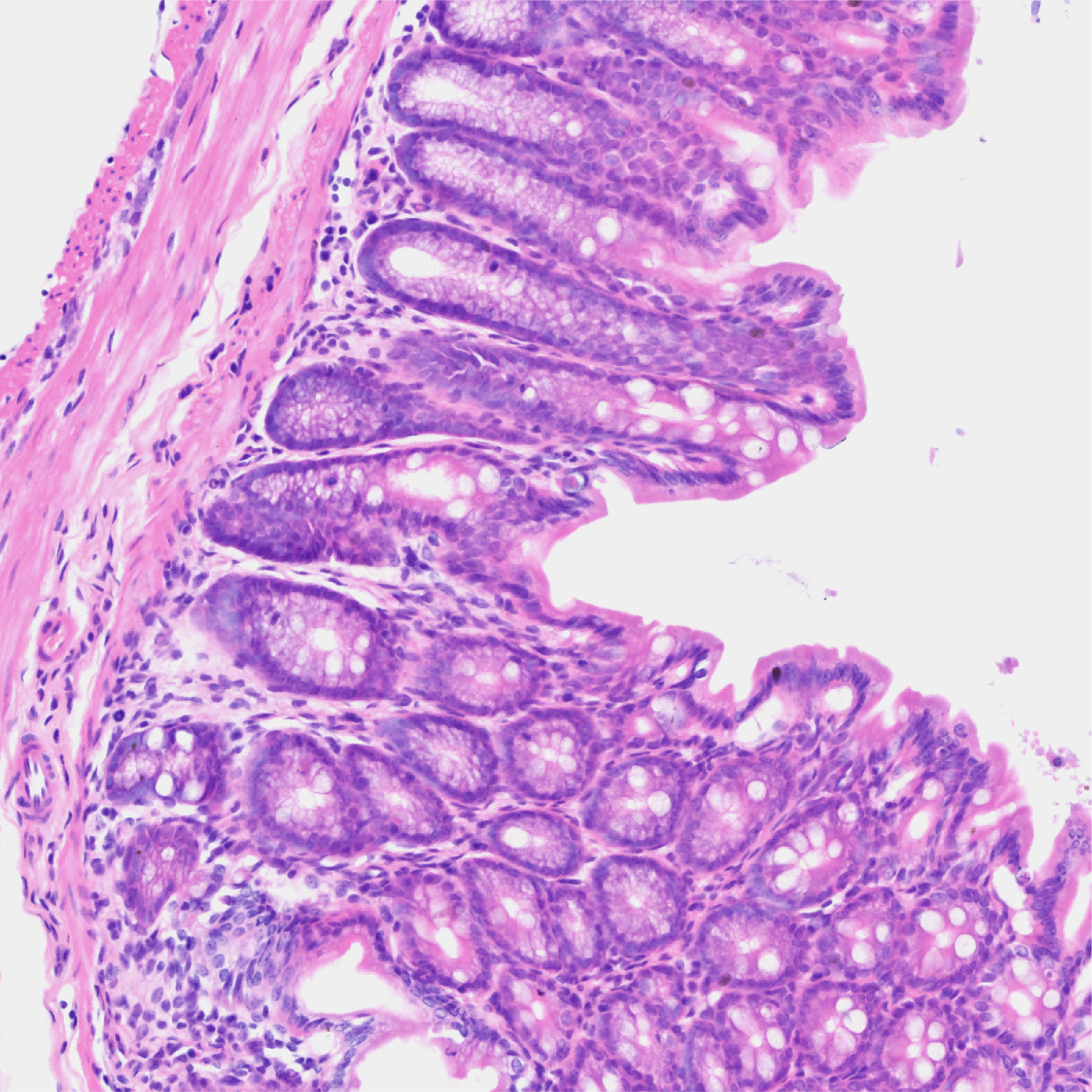

Supplement: Supplementary file 14 — EV and Appendix Figures Source Data [file 44319_2024_276_MOESM14_ESM.zip › Fig EV1/EV1-I/HE staining/Yod1++_LPS_partial image.png]

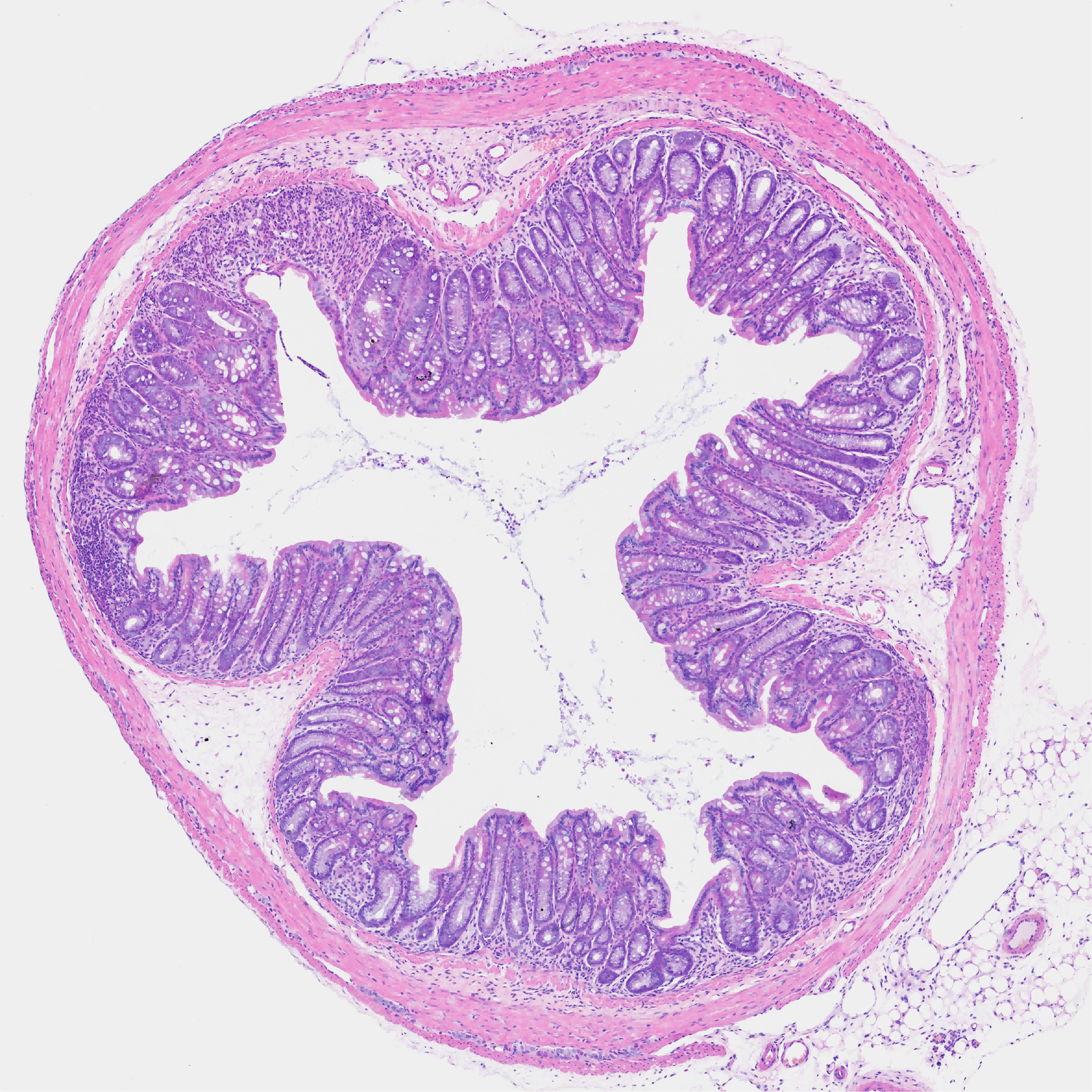

Supplement: Supplementary file 14 — EV and Appendix Figures Source Data [file 44319_2024_276_MOESM14_ESM.zip › Fig EV1/EV1-I/HE staining/Yod1++_PBS_overall image.png]

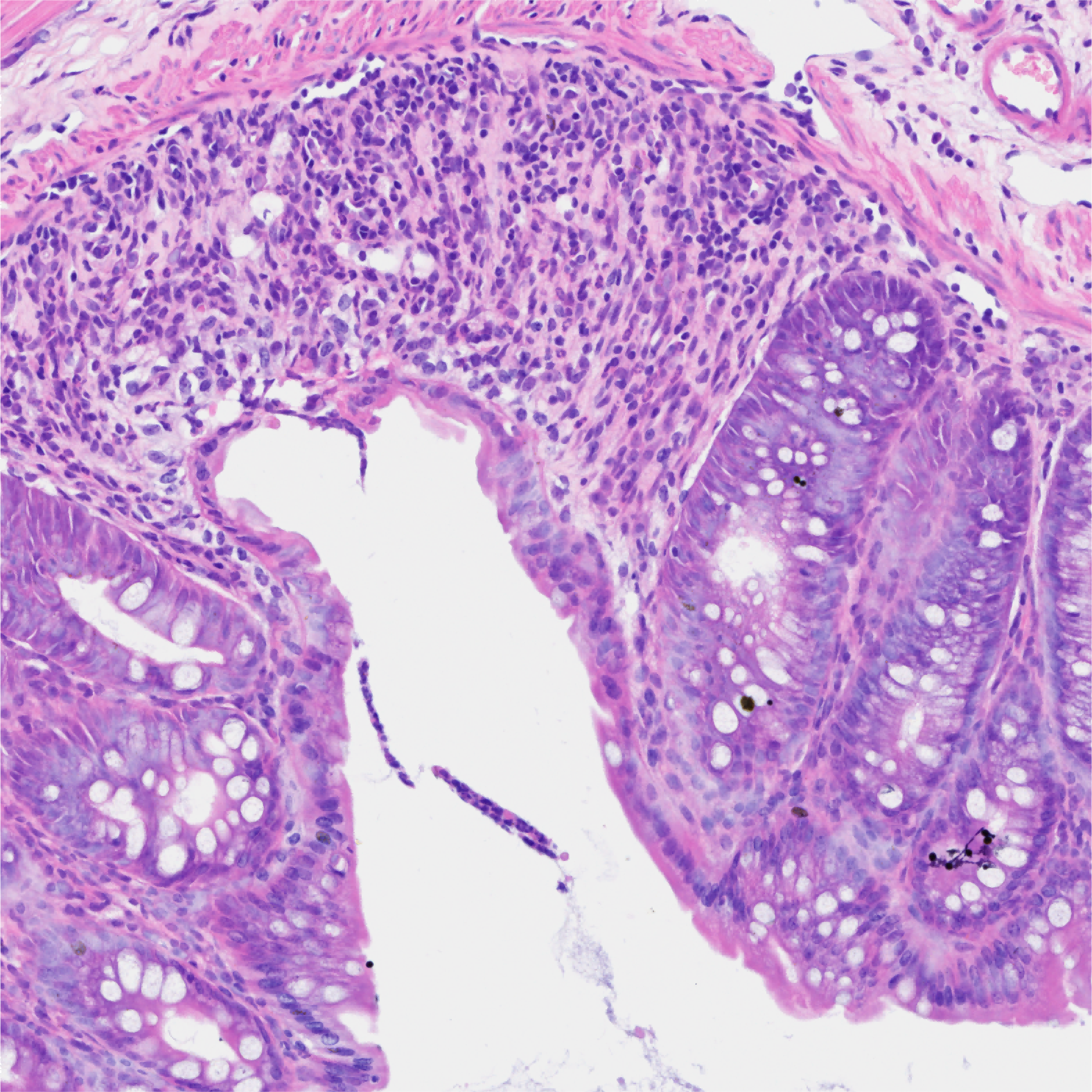

Supplement: Supplementary file 14 — EV and Appendix Figures Source Data [file 44319_2024_276_MOESM14_ESM.zip › Fig EV1/EV1-I/HE staining/Yod1++_PBS_partial image.png]

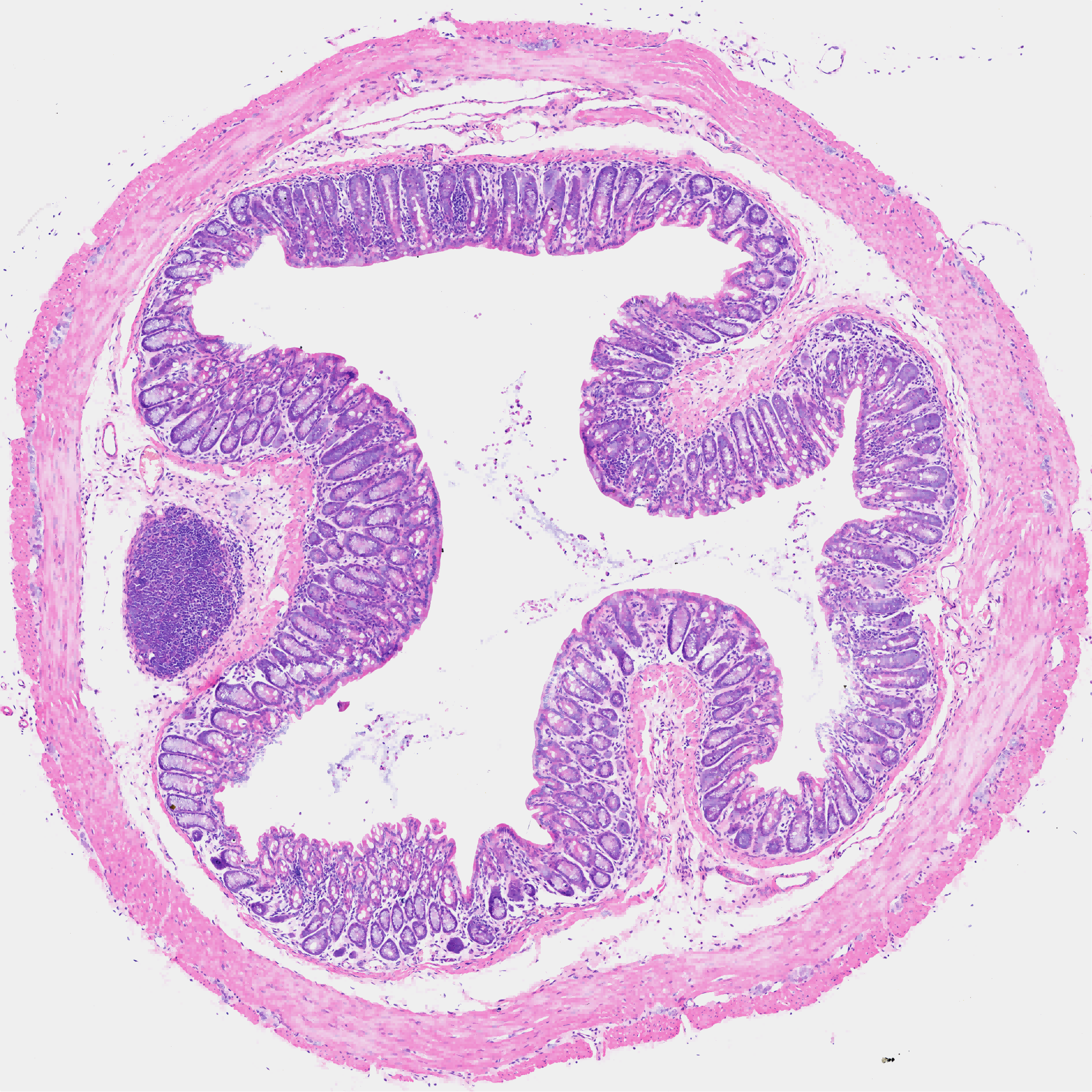

Supplement: Supplementary file 14 — EV and Appendix Figures Source Data [file 44319_2024_276_MOESM14_ESM.zip › Fig EV1/EV1-I/HE staining/Yod1--_LPS_overall image.png]

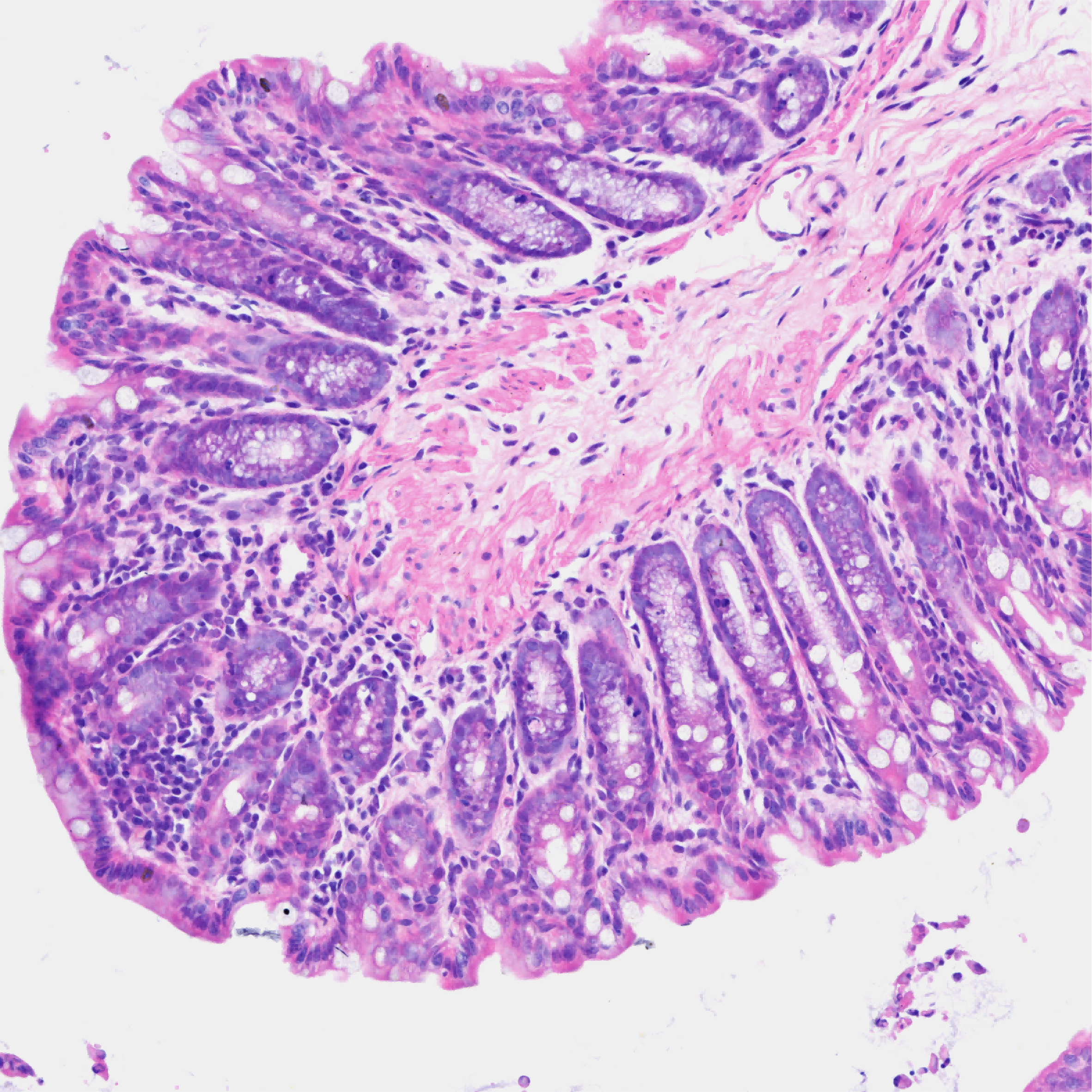

Supplement: Supplementary file 14 — EV and Appendix Figures Source Data [file 44319_2024_276_MOESM14_ESM.zip › Fig EV1/EV1-I/HE staining/Yod1--_LPS_partial image.png]
