# Supplementary figures and images for: YOD1 sustains NOD2-mediated protective signaling in colitis by stabilizing RIPK2 (part 3 of 3)
Source: EMBO Rep. 2024 Sep 27;25(11):4827–45. doi: 10.1038/s44319-024-00276-6 (PMC11549337; doi:10.1038/s44319-024-00276-6)

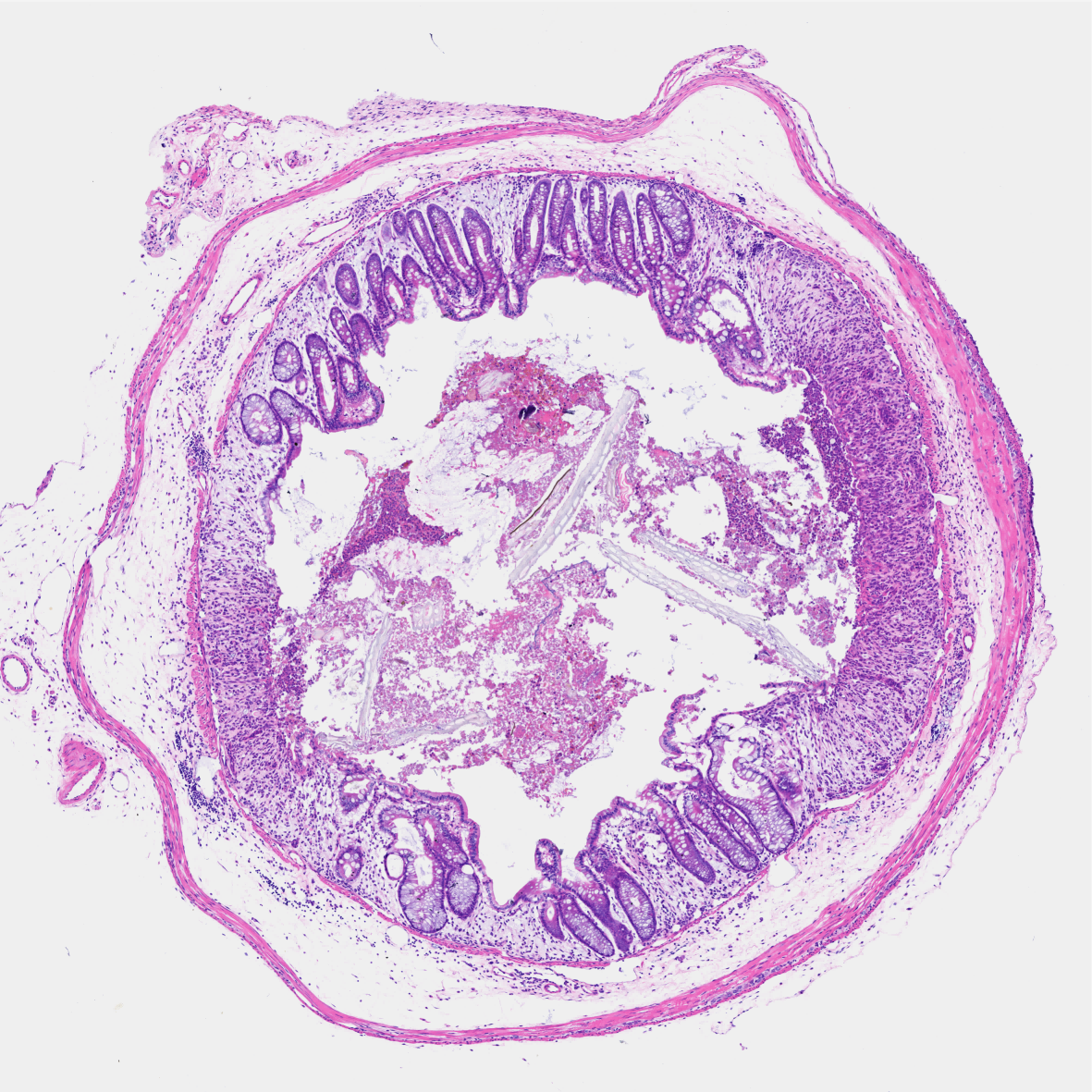

Supplement: Supplementary file 14 — EV and Appendix Figures Source Data [file 44319_2024_276_MOESM14_ESM.zip › Fig EV1/EV1-I/HE staining/Yod1--_PBS_overall image.png]

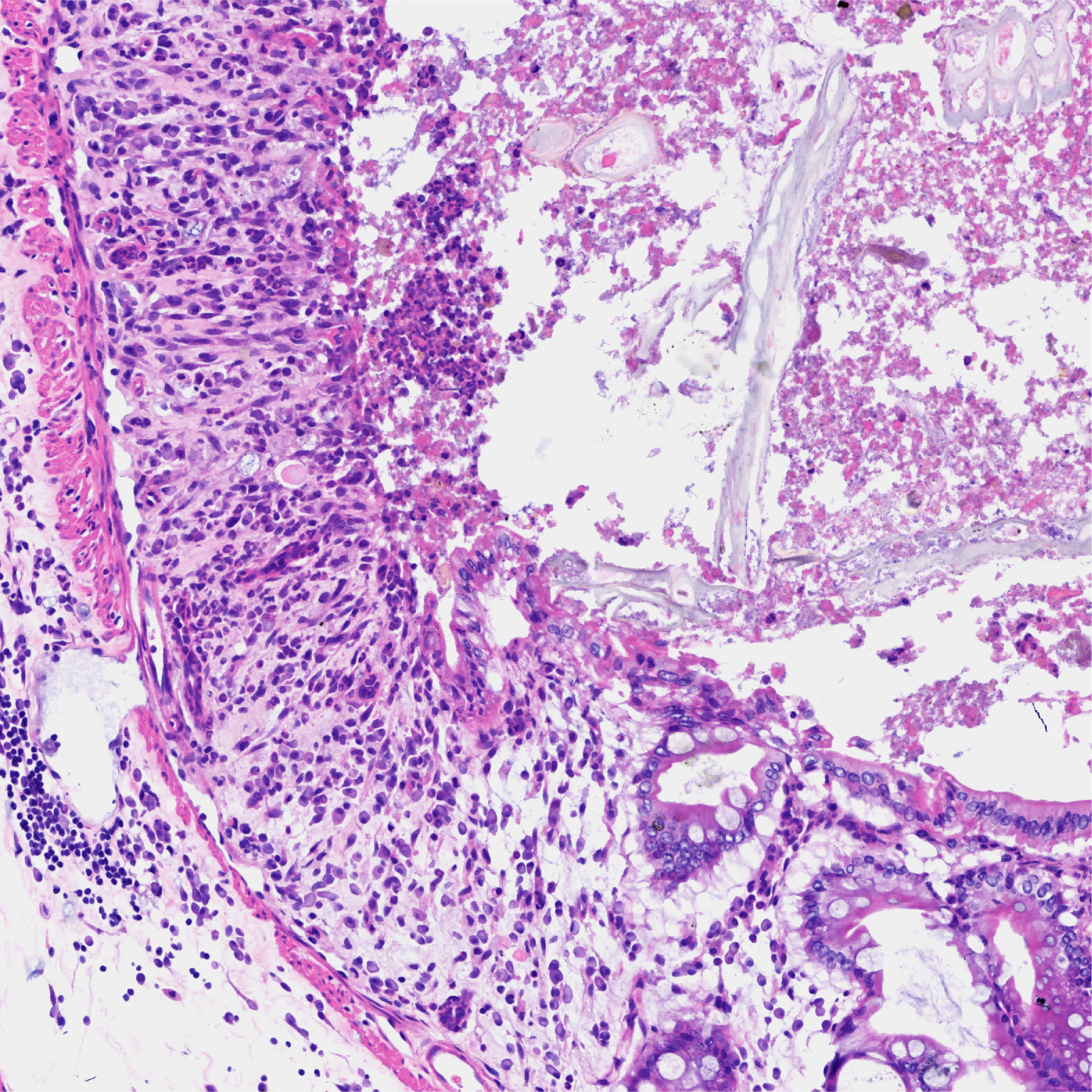

Supplement: Supplementary file 14 — EV and Appendix Figures Source Data [file 44319_2024_276_MOESM14_ESM.zip › Fig EV1/EV1-I/HE staining/Yod1--_PBS_partial image.png]

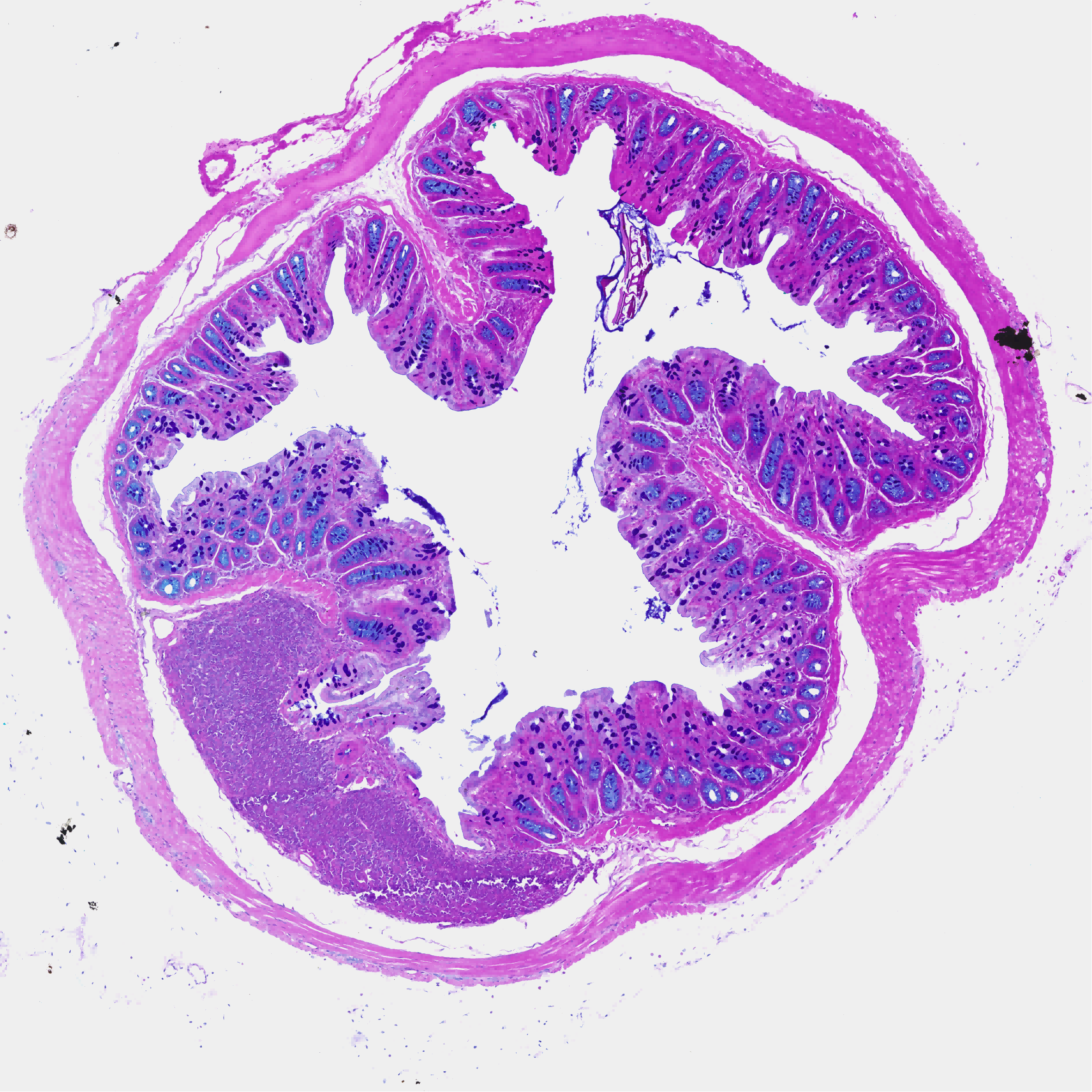

Supplement: Supplementary file 14 — EV and Appendix Figures Source Data [file 44319_2024_276_MOESM14_ESM.zip › Fig EV1/EV1-I/PAS_AB staining/Yod1++_LPS_overall view.png]

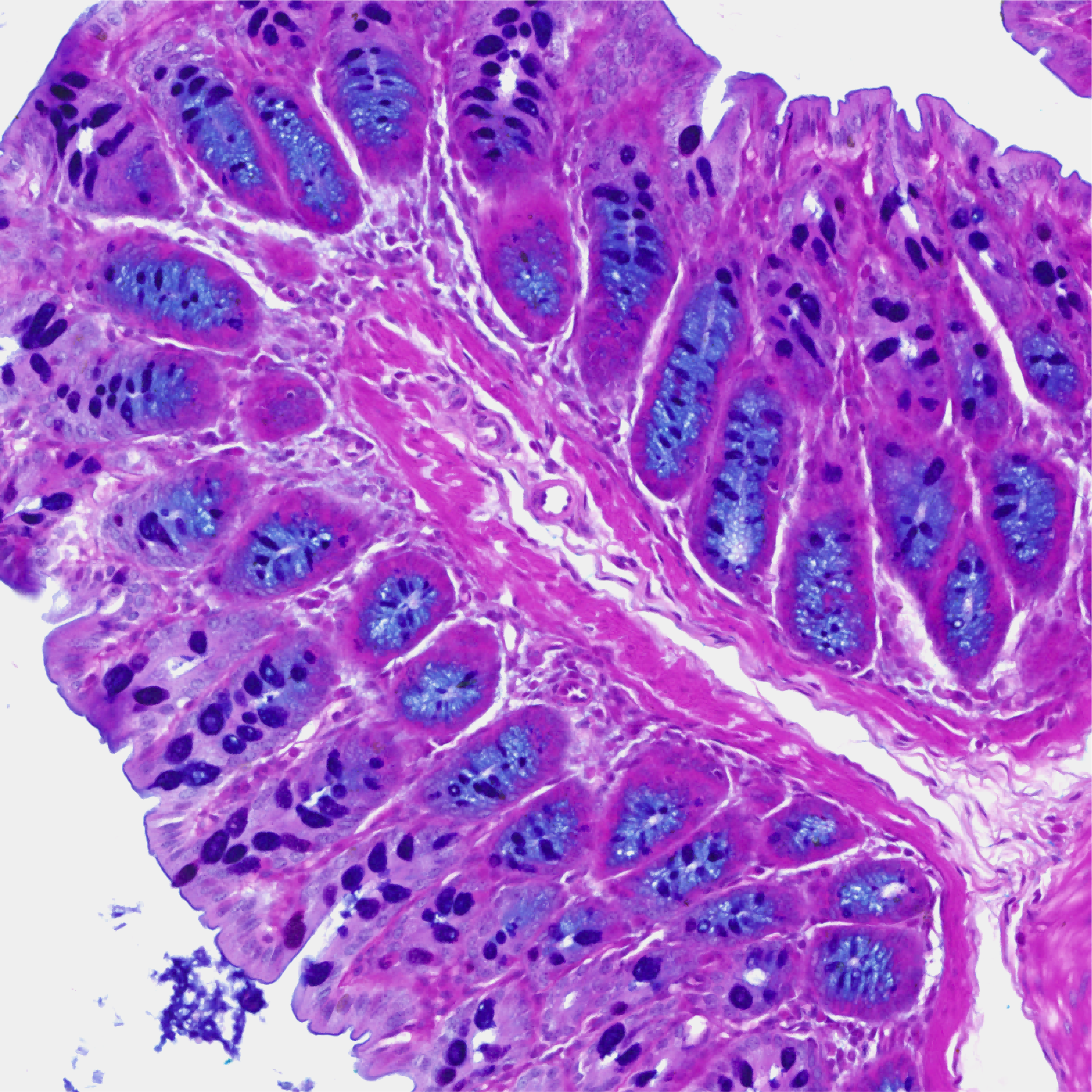

Supplement: Supplementary file 14 — EV and Appendix Figures Source Data [file 44319_2024_276_MOESM14_ESM.zip › Fig EV1/EV1-I/PAS_AB staining/Yod1++_LPS_partial view.png]

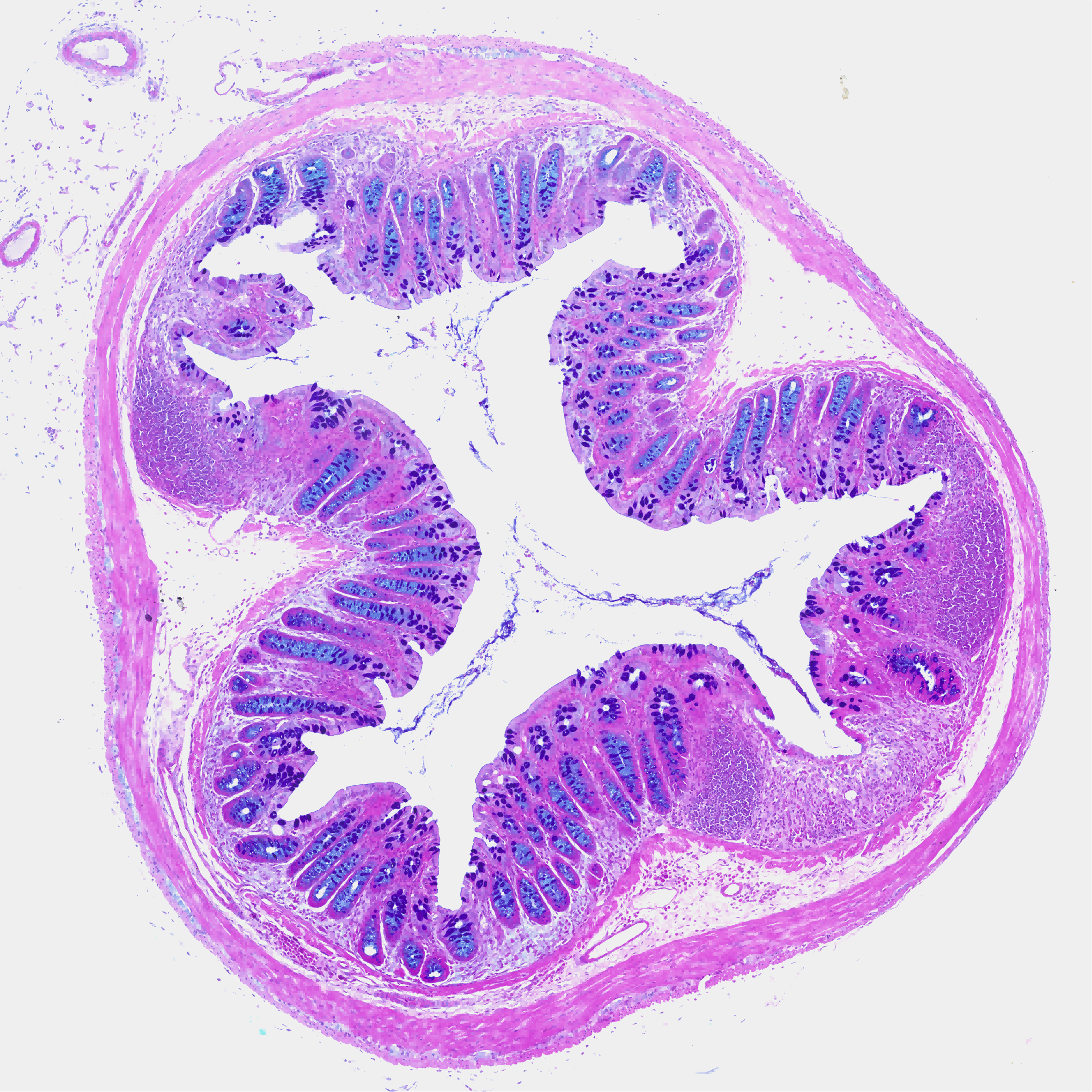

Supplement: Supplementary file 14 — EV and Appendix Figures Source Data [file 44319_2024_276_MOESM14_ESM.zip › Fig EV1/EV1-I/PAS_AB staining/Yod1++_PBS_overall view.png]

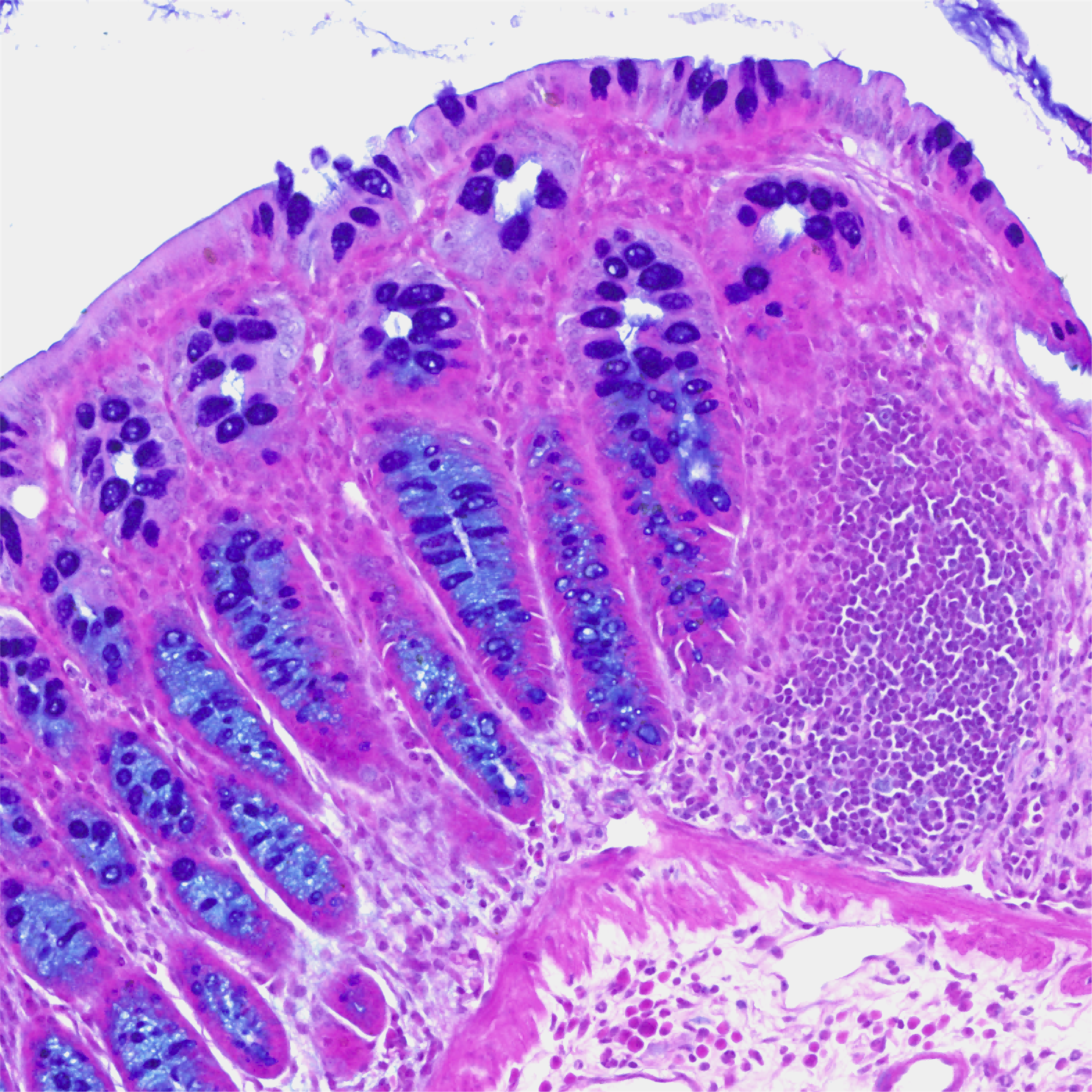

Supplement: Supplementary file 14 — EV and Appendix Figures Source Data [file 44319_2024_276_MOESM14_ESM.zip › Fig EV1/EV1-I/PAS_AB staining/Yod1++_PBS_partial view.png]

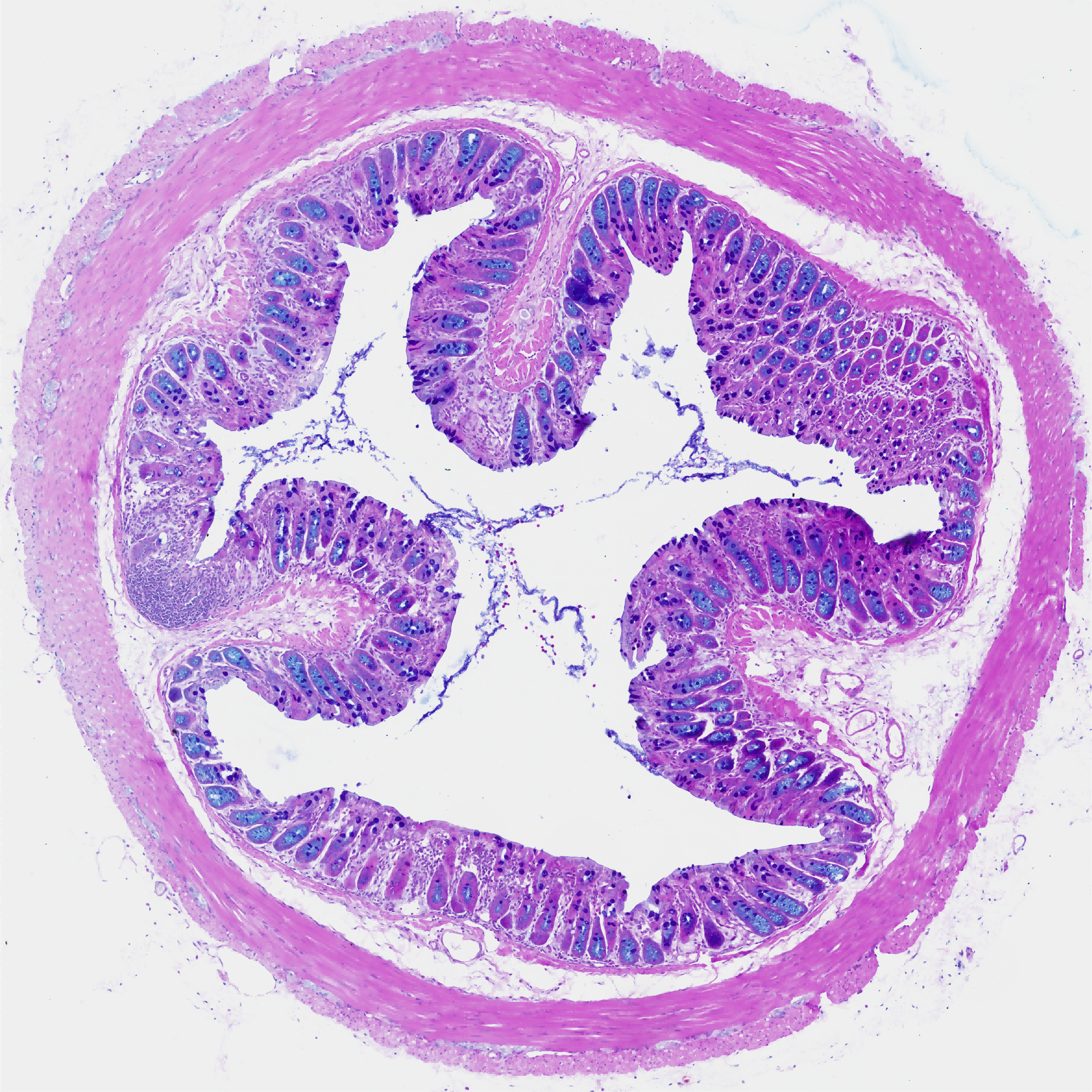

Supplement: Supplementary file 14 — EV and Appendix Figures Source Data [file 44319_2024_276_MOESM14_ESM.zip › Fig EV1/EV1-I/PAS_AB staining/Yod1--_LPS_overall view.png]

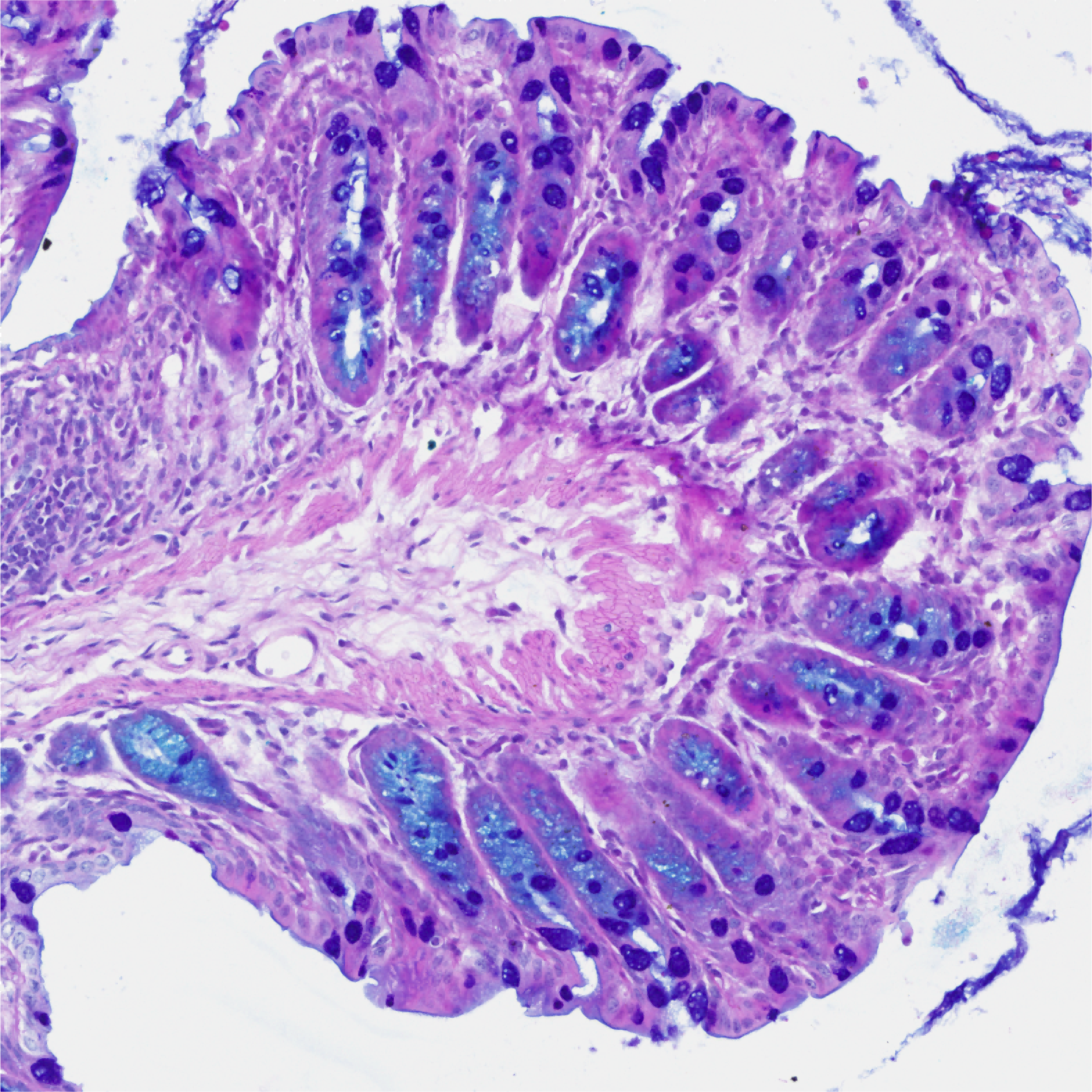

Supplement: Supplementary file 14 — EV and Appendix Figures Source Data [file 44319_2024_276_MOESM14_ESM.zip › Fig EV1/EV1-I/PAS_AB staining/Yod1--_LPS_partial view.png]

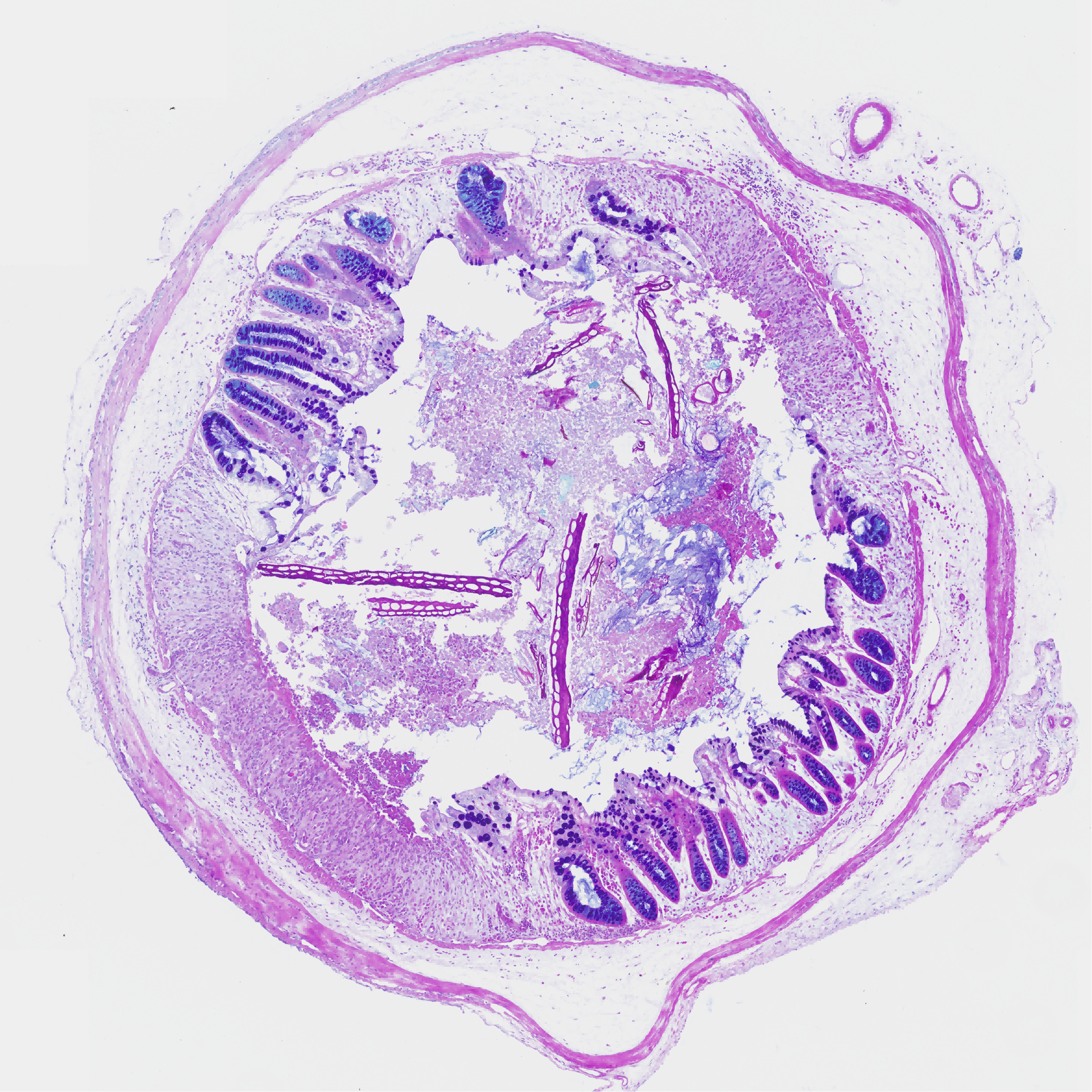

Supplement: Supplementary file 14 — EV and Appendix Figures Source Data [file 44319_2024_276_MOESM14_ESM.zip › Fig EV1/EV1-I/PAS_AB staining/Yod1--_PBS_overall view.png]

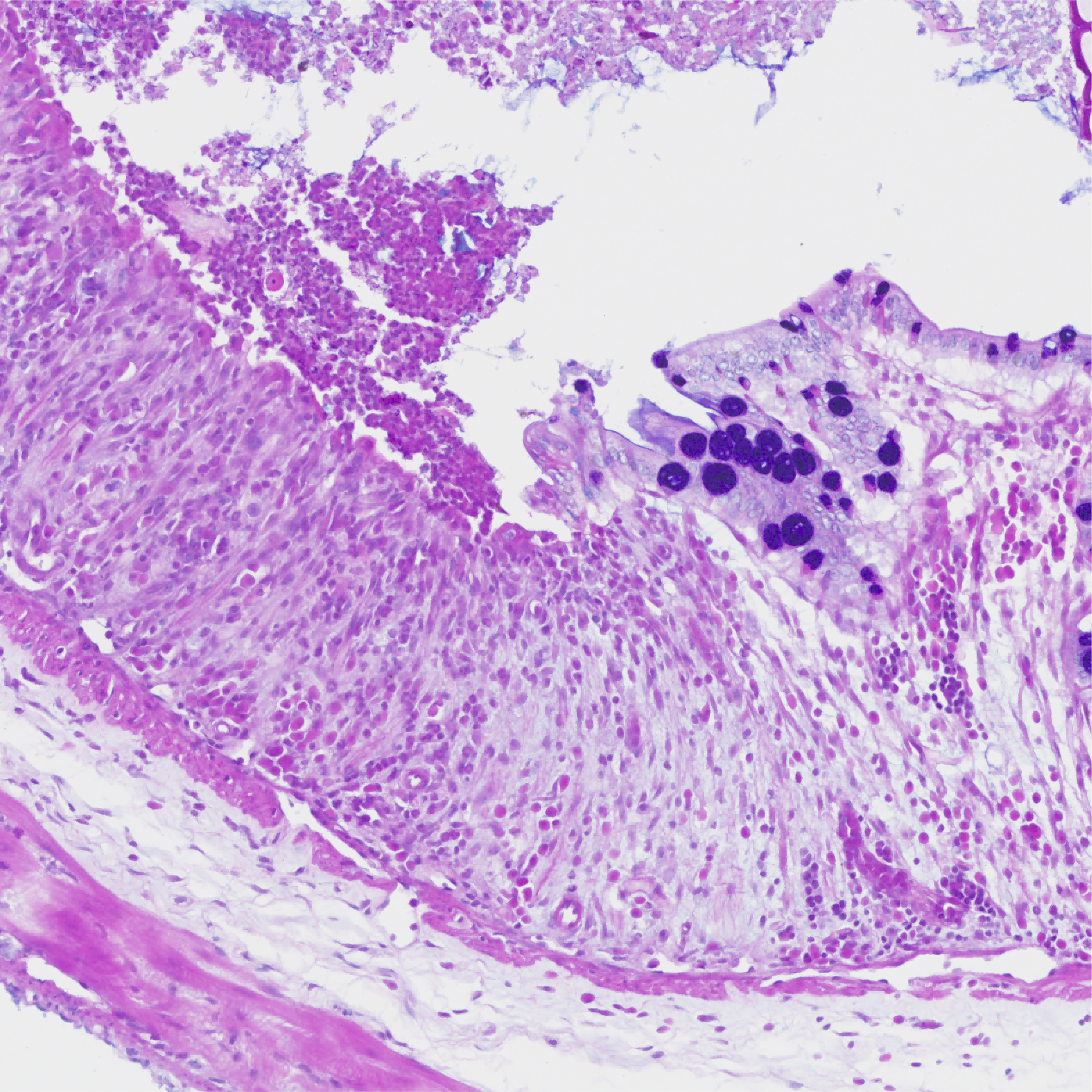

Supplement: Supplementary file 14 — EV and Appendix Figures Source Data [file 44319_2024_276_MOESM14_ESM.zip › Fig EV1/EV1-I/PAS_AB staining/Yod1--_PBS_partial view.png]
