# Supplementary material for: YOD1 sustains NOD2-mediated protective signaling in colitis by stabilizing RIPK2
Source: EMBO Rep. 2024 Sep 27;25(11):4827–45. doi: 10.1038/s44319-024-00276-6 (PMC11549337; doi:10.1038/s44319-024-00276-6)
Supplement: Supplementary file 15 — Expanded View Figures [file 44319_2024_276_MOESM15_ESM.pdf]

Expanded View Figure

Figure EV1. LPS treatment ameliorates DSS colitis in both *Yod1*$^{+/+}$ and *Yod1*$^{-/-}$ mice.

(**A**) Experimental flowchart for the DSS experiment with LPS treatment. (**B**) Body weight change of *Yod1*$^{+/+}$ and *Yod1*$^{-/-}$ mice ($n = 6$, biological replicates). (**C**, **D**) Rectal bleeding (**C**) and stool consistency (**D**) of *Yod1*$^{+/+}$ and *Yod1*$^{-/-}$ mice on day 7 after DSS treatment ($n = 6$, biological replicates). (**E–H**) Representative image (**E**), length (**F**), histology score (**G**), and PAS/AB positive area (**H**) of colons from *Yod1*$^{+/+}$ and *Yod1*$^{-/-}$ mice on day 7 after DSS treatment ($n = 6$, biological replicates). (**I**) Representative H&E and PAS/AB staining of colons from *Yod1*$^{+/+}$ and *Yod1*$^{-/-}$ mice on day 7 after DSS treatment. Scale bar = 100 μm. Data information: Data are representative of two replicates. Data in (**B–D**, **F–H**) show the mean ± SEM. Statistical analyses were performed using two-way ANOVA followed by the Sidak post-test (**B–D**, **F–H**). Source data are available online for this figure.

 © The Author(s)

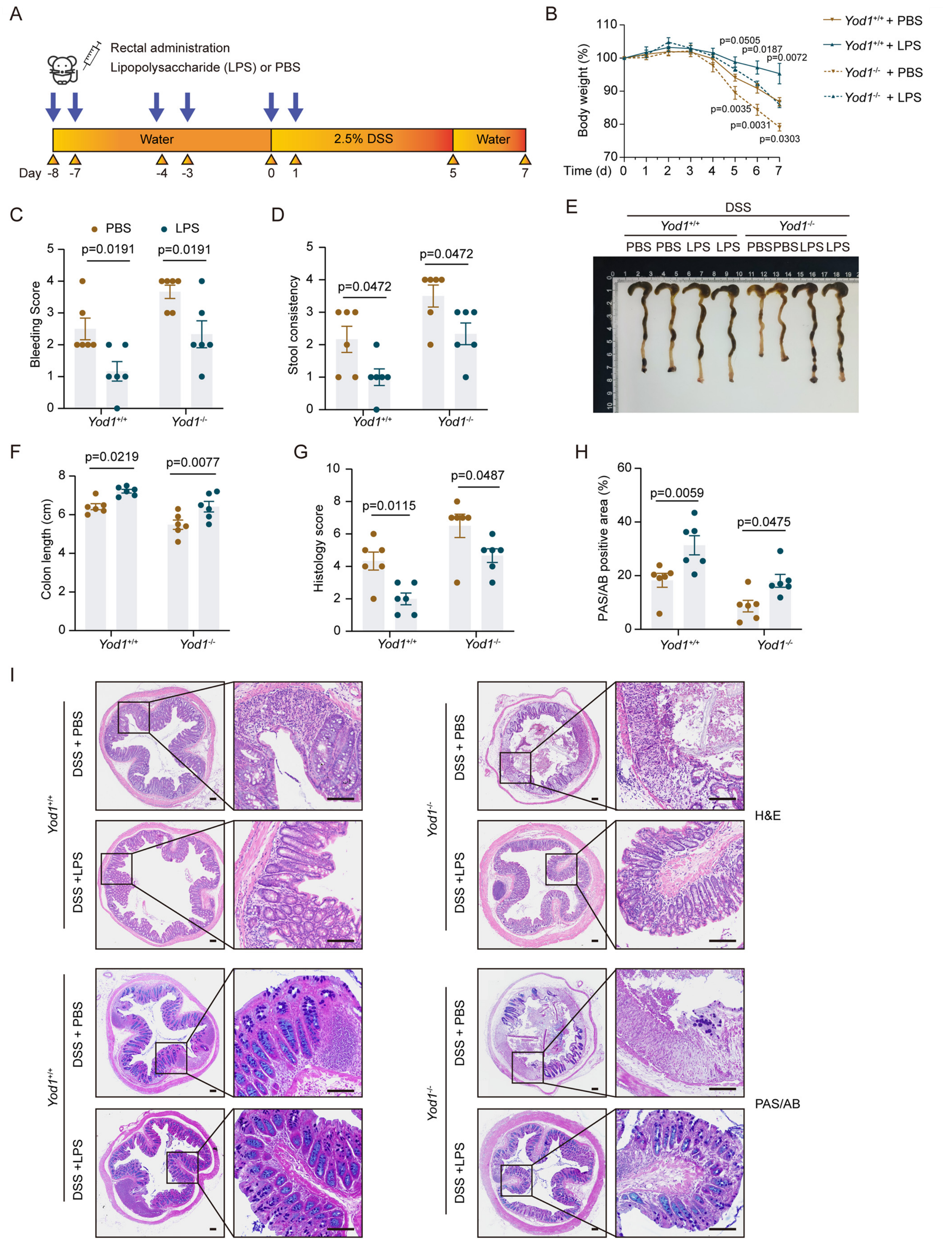
A
Rectal administration
Lipopolysaccharide (LPS) or PBS
Water
2.5% DSS
Water
Day -8 -7 -4 -3 0 1 5 7
B
Body weight (%)
Time (d)
Yod1+/+ + PBS
Yod1+/+ + LPS
Yod1-/- + PBS
Yod1-/- + LPS
p=0.0505
p=0.0187
p=0.0072
p=0.0035
p=0.0031
p=0.0303
C
PBS
LPS
Bleeding Score
p=0.0191
p=0.0191
Yod1+/+
Yod1-/-
D
Stool consistency
p=0.0472
p=0.0472
Yod1+/+
Yod1-/-
E
DSS
Yod1+/+
Yod1-/-
PBS PBS LPS LPS PBS PBS LPS LPS
F
Colon length (cm)
p=0.0219
p=0.0077
Yod1+/+
Yod1-/-
G
Histology score
p=0.0115
p=0.0487
Yod1+/+
Yod1-/-
H
PAS/AB positive area (%)
p=0.0059
p=0.0475
Yod1+/+
Yod1-/-
I
Yod1+/+
Yod1-/-
DSS + PBS
DSS +LPS
H&E
PAS/AB

© The Author(s)
